# Synthesis, Antiparasitic Activity, and ADME Profile of Morpholinephenylbenzamides: A Pharmacological Study Against Chagas Disease and Visceral Leishmaniasis

**DOI:** 10.1002/cmdc.70518

**Published:** 2026-10-08

**Authors:** Danilo Nascimento Farago, Daniela de Melo Resende, Mariza Gabriela Faleiro de Moura Lodi Cruz, Rafaela Marçal Sousa, Rafael Consolin Chelucci, Simone Michelan‐Duarte, Felipe da Silva Mendonça de Melo, Douglas Davison da Silva Oliveira, Paula Derksen Macruz, Alexandra Maria dos Santos Carvalho, Eduardo Jorge Pilau, Izabela Marques Dourado Bastos, Adriano D. Andricopulo, Leonardo L. G. Ferreira, Silvane Maria Fonseca Murta, Celso de Oliveira Rezende Júnior

**Affiliations:** ^1^ Laboratório de Síntese de Candidatos a Fármacos Instituto de Química Universidade Federal de Uberlândia (UFU) Uberlândia Minas Gerais Brazil; ^2^ Grupo Genômica Funcional de Parasitos Instituto René Rachou Fundação Oswaldo Cruz (FIOCRUZ Minas) Belo Horizonte Minas Gerais Brazil; ^3^ Laboratório de Química Medicinal e Computacional (LQMC) Instituto de Física de São Carlos (IFSC) Universidade de São Paulo (USP) São Carlos São Paulo Brazil; ^4^ Pathogen‐Host Interface Laboratory Department of Cell Biology University of Brasilia (UnB) Brasília Distrito Federal Brazil; ^5^ Laboratório de Biomoléculas e Espectrometria de Massas (LaBioMass) Universidade Estadual de Maringá (UEM) Maringá Paraná Brazil; ^6^ Departamento de Química Universidade Federal de Juiz de Fora (UFJF) Juiz de Fora Minas Gerais Brazil

**Keywords:** drug discovery, hit‐to‐lead, neglected tropical diseases

## Abstract

Chagas disease and leishmaniasis are neglected tropical diseases with limited treatment options. This study reports a hit‐to‐lead optimization of morpholinephenylbenzamides, starting from hit **1**, which showed potent activity against *Trypanosoma cruzi* (IC_50_ 0.29–0.76 μM) and *Leishmania infantum* (IC_50_ 10.7 μM), despite a suboptimal ADME profile. A structure–activity relationship study of twenty‐four novel derivatives provided key pharmacophoric insights. While *Trypanosoma cruzi* was less susceptible to chemical modifications, compound **20** exhibited potency comparable to benznidazole (IC_50_ 3.58 μM) with significantly improved selectivity, lipophilicity, and solubility relative to compound **1**. Against *Leishmania infantum*, six derivatives maintained activity, with compound **24** showing a 1.5‐fold potency increase IC_50_ 7.6 μM). Additionally, compound **16** stood out for its superior ADME profile, including enhanced permeability and metabolic stability. These findings validate the morpholinephenylbenzamide scaffold as a robust class with dual antiparasitic activity.

## Graphical abstract

Hit‐to‐lead optimization of morpholinephenylbenzamides reveals key structure–activity relationships against *Trypanosoma cruzi* and *Leishmania infantum*. Potency, selectivity, and ADME properties are differentially improved across the series, identifying compounds **20**, **24**, and **16** as complementary templates for the future design of dual antiparasitic leads.

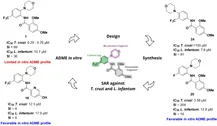

## 1 Introduction

Neglected tropical diseases (NTDs) affect over one billion people worldwide and include 20 conditions that predominantly impact impoverished communities in tropical regions. They are caused by diverse pathogens such as viruses, bacteria, parasites, fungi, and toxins; among them are *Trypanosomatidae*‐related infections such as Chagas disease and leishmaniasis [1, 2, 3]. Chagas disease affects more than seven million people globally, with fewer than 10% diagnosed. Although endemic to Latin America, it has been increasingly reported in Europe and North America [4, 5]. Similarly, visceral leishmaniasis, the most severe form of leishmaniasis and the second deadliest parasitic disease, causes over one million new cases and 20,000–40,000 deaths annually [6, 7, 8]. Although both diseases are caused by parasites from the same family, their etiological agents and transmission vectors differ. In Chagas disease, the causative agent is *Trypanosoma cruzi* (*T. cruzi*), transmitted through the feces of infected triatomine bugs [9, 10]. Visceral leishmaniasis, in contrast, is caused by various *Leishmania* (*L*) species, mainly *L. donovani* in India and East Africa and *L. infantum* in Europe and Latin America, which are transmitted primarily by female sandflies [11, 12].

The only two available treatments for Chagas disease are nifurtimox and benznidazole (Figure 1), nitroheterocyclic drugs developed over 50 years ago. Both have several limitations, including parasite resistance, limited efficacy (particularly in the chronic phase), long treatment duration (60–90 days), and serious side effects such as anorexia, paresthesia, weight loss, and nausea, often leading to treatment interruption [13, 14]. Currently, several drugs with different mechanisms are available for the treatment of visceral leishmaniasis, including pentavalent antimonials (Pentostam and Glucantime), miltefosine, paromomycin, pentamidine, and amphotericin B (Figure 1). However, their clinical use is limited by high toxicity, severe side effects, parasite resistance, high cost, and prolonged treatment duration. Additionally, the pain and discomfort associated with parenteral administration represent further challenges [14, 15].

**FIGURE 1:**
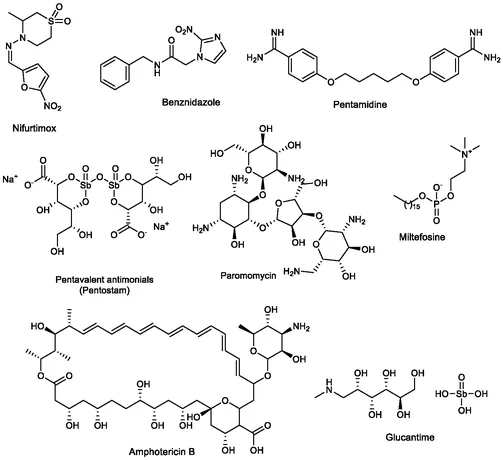
Chemical structures of the current therapeutics for Chagas disease and main drugs used to treat visceral leishmaniasis.

Altogether, these factors underscore the urgent need for the development of new effective drugs to treat these infections. Therefore, there is currently strong collaboration between the pharmaceutical industry and academia to develop new compounds with potential activity against these diseases, resulting in a growing number of studies in this field, but there is still a lack of efficient treatment [7, 14, 15, 16]. In this work, we conducted a hit‐to‐lead optimization study focused on the biological activity of a series of morpholinephenylbenzamides compounds against *T. cruzi* and *L. infantum*. The initial hit, compound **1** (Figure 2), was selected from the high‐throughput screening work due to its high activity against amastigote forms of *T. cruzi* (Tulahuen strain) and *L. donovani* and low cytotoxicity against HepG2 cells [17]. This compound contains morpholine and two aryl rings in its structure, two very well‐known groups of fragments that are present in a high number of bioactive compounds and have their biological activity and metabolism extensively studied [18, 19]. We focused our studies on the evaluation of pharmacophore groups by modifying the morpholine, the central aryl ring, and the benzamide moieties. The compounds exhibiting the best antiparasitic activities were subsequently subjected to in vitro ADME assays to identify those with a suitable profile for in vivo studies (lead candidates).

**FIGURE 2:**
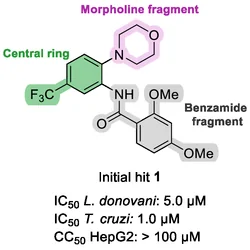
Structure of the selected initial hit **1** with its biological activity reported in [17].

## 2 Results and Discussion

### 2.1 Synthesis and In Vitro Pharmacological Evaluation of the Initial Hit 1

The initial hit **1** was obtained following the synthetic route depicted in Scheme 1. The synthesis started with the aromatic nucleophilic substitution of fluoride compound **2a** with morpholine **3a**, affording intermediate arylmorpholine **4a** in 97% yield, which was subsequently reduced to the corresponding aniline **5a** in 99% yield via catalytic hydrogenation with palladium (0). Hit **1** was then synthesized from aniline **5a** using two distinct amide bond–forming reactions. The first reaction involved carboxylic acid **6a** mediated by *O*(benzotriazol‐1‐yl)‐*N*,*N*,*N′*,*N′*‐tetramethyluronium hexafluorophosphate (HBTU), which provided **1** in a 29% yield. The second method used an acyl chloride (prepared from carboxylic acid **6a** with oxalyl chloride), providing **1** in a 53% yield. The reaction employing the acyl chloride was proved to be more efficient as it afforded a higher yield and proceeded under milder temperature conditions than the HBTU‐mediated coupling. In both procedures, complete consumption of aniline **5a** was not achieved, even with an excess of the coupling partner (carboxylic acid or acyl chloride). This highlights the poor nucleophilicity of aniline **5a**, which is likely caused by steric hindrance from the morpholine fragment located at the ortho position relative to the amino group (NH_2_).

**SCHEME 1:**
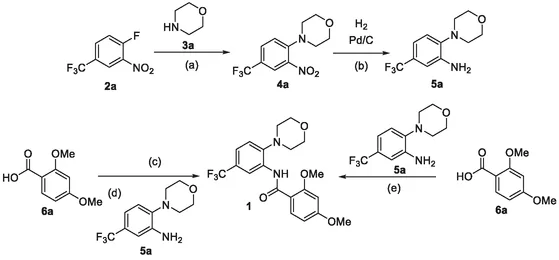
Synthesis of initial hit **1**. Reagents and conditions: (a) K_2_CO_3_, DMF, 60 °C, 24 h, 97%; (b) MeOH, r.t. 12 h, 99%; (c) oxalyl chloride, DMF (cat.), DCM, r.t., Ar atm. 5 h; (d) Et_3_N, DCM, r.t., Ar atm., 114 h, 53%; (e) HBTU, Et_3_N, DMF, r.t. −80 °C, 139 h, 29%.

Biological activity of **1** was evaluated against the amastigote forms of *T. cruzi* (Tulahuen strain) and *L. infantum* (MHOM/BR/1974/PP75) infecting L929 and THP‐1 cells, respectively, to determine its potency (IC_50_) and cytotoxicity (CC_50_) and confirm the antiparasitic activities reported in the high‐throughput screen study that discovered the initial hit. Compound **1** showed high activity against *T. cruzi* (IC_50_ 0.76 µM), coupled with a high selectivity index (SI = CC_50_/IC_50_ = 109.5) (Table 1). However, its activity against *L. infantum* was moderate, as reflected by a higher IC_50_ (10.7 µM) and a lower SI (36.3). These findings confirm the potential of this chemical class to combat both Chagas disease and visceral leishmaniasis, establishing compound **1** as a promising initial hit for subsequent hit‐to‐lead optimization. The in vitro ADME profile of **1** was also screened, including lipophilicity (logD), kinetic solubility (pH 2.0 and pH 7.4), PAMPA permeability, and microsomal metabolic stability (Table 1). This comprehensive analysis was performed to evaluate its potential for adequate bioavailability and to identify shortcomings that could be improved by subsequent chemical modifications. A favorable pharmacokinetic profile is essential for oral drug candidates, especially those intended for chronic diseases where patient adherence is critical. Therefore, addressing potential ADME liabilities early is crucial in the drug discovery process [20]. The evaluated ADME parameters suggest a limited pharmacokinetic profile for **1**, primarily due to its low aqueous solubility (KS = 4. 10 µM in pH 2.0 and 6.14 µM in pH 7.4) [21, 22], poor permeability (PAMPA = 0.78 × 10^−6^ cm/s), and low microsomal metabolic stability (*t*_1/2_ = 15.61 min and Clint = 177.6 µL/min/mg) [23]. This poor aqueous solubility and metabolic stability may be related to its high lipophilicity (elogD = 4.60), which falls outside the optimal range (between 1 and 3) [24].

**TABLE 1:** Hit **1** biological and ADME in vitro properties.

Cp	*T. cruzi* (Tulahuen)		*L. infantum*		**logD** e	**KS, µM** f		**PAMPA** ***P***_**e**_ **,** **10** ^**−6**^ **cm/s** g	***t***_**1/2**_ **MLM, min** h	**MLM Cl**_**int**_, **µL/min/mg** i
	**IC**_**50**_, **µM** **(SI)** a	**CC**_**50**_ **L929** b, **µM**	**IC**_**50**_, **µM** **(SI)** c	**CC**_**50**_ **THP‐1** d, **µM**		pH 2.0	pH 7.4			
**1**	0.76 ± 0.02 (109.5)	82.7	10.7 ± 1.2 (36.3)	390.4	4.60	4.10	6.15	0.78	15.61	177.6

a IC_50_ value for the inhibition of the growth of *T. cruzi* in L929 cells (mean of at least two independent determinations, *p* < 0.05), SI, selectivity index = CC_50_/IC_50_ in L929 cells.

b CC_50_ value measured in L929 cells (mean of at least two independent determinations, *p* < 0.05).

c IC_50_ value for the inhibition of the growth of *L. infantum* in THP‐1 macrophages (mean of at least two independent determinations, *p* < 0.05), SI, selectivity index = CC_50_/IC_50_ in THP‐1 cells.

d CC_50_ value measured in THP‐1 macrophages (mean of at least two independent determinations, *p* < 0.05).

e Experimental lipophilicity, determined at pH, 7.4.

f Kinetic solubility (µM).

g PAMPA permeability. *P*_e_ < 1.5.10^−6^ cm/s: low permeability, *P*_e_ > 1.5.10^−6^ cm/s: high permeability.

h Microsomal half‐life determined in Mouse Liver Microsomes (MLM), in presence of NADPH cofactor.

i Intrinsic clearance determined in MLM, in presence of NADPH cofactor. Amphotericin B (reference drug) was used as positive control against *L. infantum* (IC_50_ of 0.25 μM; CC_50_ of 364 μM); Benznidazole (reference drug) was used as positive control against *T. cruzi* (IC_50_ of 3.8 μM; CC_50_ of 2381 μM).

### 2.2 Structure–Activity Relationship Against *T. cruzi* and *L. infantum*

Although **1** exhibited satisfactory potency against both parasites, meeting the IC_50_ < 10 µM criteria to hit compounds [25], its poor ADME profile represented the main limitation. To overcome this issue, structural modifications were designed and implemented on the benzamide, morpholine, and phenyl fragments of **1** (Figure 2). The goal was to explore key pharmacophoric groups, establish a structure–activity relationship (SAR) against *T. cruzi* (Tulahuen) and *L. infantum*, and reduce lipophilicity, thereby improving kinetic solubility and metabolic stability. Lipophilicity of the designed compounds was estimated using the theoretical partition coefficient (clogP) via the swissADME platform [26], which served as a predictive tool for reducing this parameter. Furthermore, to enhance metabolic stability, functional groups potentially involved in oxidative metabolism, such as methoxy (OMe), were either removed or replaced with less susceptible moieties. All synthesized compounds were subsequently tested against the amastigote forms of *T. cruzi* and *L. infantum*, and the CC_50_ was determined for the most active derivatives.

### 2.3 Chemical Modifications on the Benzamide Fragment

The first series of modifications was carried out on the benzamide fragment using aniline **5a** previously synthesized as shown in Scheme 1. Synthesis of compounds **7**–**12**, **14,** and **16**–**19** was achieved via amide bond formation using three different sets of reaction (Scheme 2). In the first condition, the corresponding acyl chloride was prepared *in situ* from the carboxylic acid analog using oxalyl chloride and subsequently coupled with aniline **5a**. The other two conditions involved direct amide coupling of the free carboxylic acid using a coupling agent: one employing HBTU and triethylamine (Et_3_N) as the base and the other with 1‐ethyl‐3‐(3‐dimethylaminopropyl)carbodiimide (EDC) in the presence of 4‐(dimethylamino)pyridine (DMAP) as the catalyst.

**SCHEME 2:**
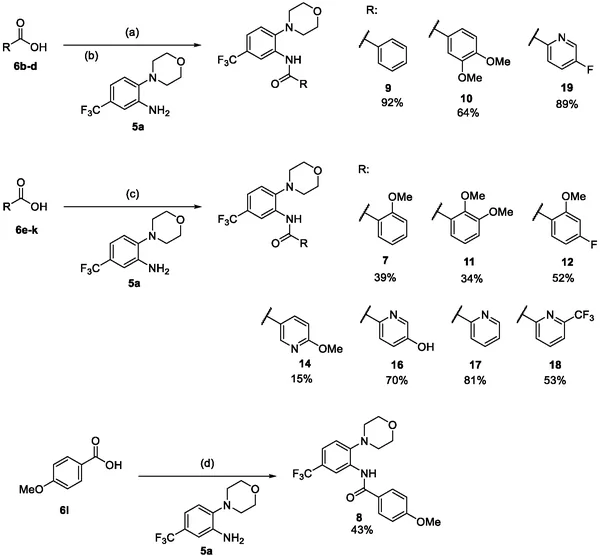
Synthesis of compound **7**–**12**, **14**, and **16**–**19**. Reagents and conditions: (a) oxalyl chloride, DMF (cat.), DCM, Ar atm, 0 °C → r.t.; (b) Et_3_N, DCM, Ar atm, r.t., 64%–92%; (c) HBTU, Et_3_N, DMF, 60°C–95 °C, 15%–81%; (d) EDC, DMAP, DCM/ACN, r.t. →70 °C, 92 h, 43%.

Compound **13** was synthesized by demethylation of **1** using a BBr_3_ solution in DCM. Conversely, compound **15** was obtained via methylation of **16** using dimethyl sulfate in the presence of sodium *tert*‐butoxide as a base (Scheme 3).

**SCHEME 3:**
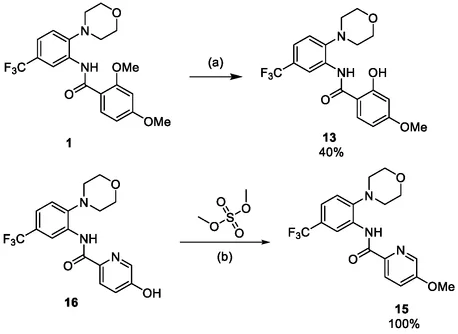
Synthesis of compound **13** and **15**. Reagents and conditions: (a) BBr_3_ (1 M in DCM), 0 °C → r.t., Ar atm, 49 h, 40%; (b) *t*‐BuONa, DMF, Ar atm, 21 h, 100%.

The biological activity of these compounds with modifications on the benzamide fragment, against the amastigote forms of both parasites, is summarized in Table 2, along with their theoretical lipophilicity (clogP). The first set of modifications on the benzamide fragment focused on the methoxy groups, given their well‐known influence on biological activity and their susceptibility to metabolic oxidation [27]. The removal of one of the methoxy groups (**7** and **8**) or both (**9**) resulted in a complete loss of activity at the maximum concentration tested (IC_50_ > 100 µM) against *T. cruzi*. In contrast, the removal of the *para*‐methoxy group (compound **7**) or both methoxy groups (compound **9**) resulted in a complete loss of activity against *L. infantum*. However, the removal of the *ortho*‐methoxy group (compound **8**) was partially tolerated, causing a 2.5‐fold decrease in potency (IC_50_ = 27.2 µM for **8** vs. IC_50_ = 10.7 µM for **1**). Crucially, the selectivity index (SI = 37.3) remained comparable to that of **1** (SI = 36.3). Repositioning the methoxy groups of **1** from the *ortho* to the *meta* position (**10**) and from the *para* to the *meta* position (**11**) was then evaluated. Both rearrangements resulted in a loss of potency against *T. cruzi* (IC_50_ > 100 µM). Against *L. infantum*, compound **10** was inactive, while the *para*‐to‐*meta* modification (**11**) was less detrimental (IC_50_ = 27.6 µM, SI = 14.4). Furthermore, modification of the methoxy group at the *ortho* or *para* position was assessed. Substitution with a fluorine atom (**12**) led to a complete loss of activity against both parasites (IC_50_ > 100 µM). In contrast, replacement with a hydroxyl group (**13**) abolished activity against *T. cruzi* (IC_50_ > 100 µM) but retained similar potency to **1** against *L. infantum* (IC_50_ = 10.0 µM), albeit with a marked reduction in selectivity (SI = 9.2).

**TABLE 2:** In vitro anti‐*T. cruzi* and anti‐*L. infantum* activities and clogP values of compounds **7–13**.

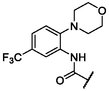						
Compound	clogP	**IC**_**50**_ ***T. cruzi,*** **µM** **(SI)** a	**CC**_**50**_ **L929, µM** b	**IC**_**50**_ ***L. infantum*** **,** **µM** **(SI)** c	**CC**_**50**_ **THP‐1, µM** d	
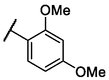 **1**	3.32	0.76 ± 0.02 (109.5)	82.7	10.7 ± 1.2 (36.3)	390.4	
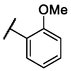 **7**	3.35	>100	N.T.	>100	N.T.	
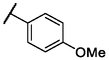 **8**	3.36	>100	N.T.	27.2 ± 1.8(37.3)	1012.2	
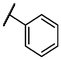 **9**	3.46	>100	N.T.	>100	N.T.	
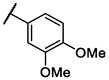 **10**	3.35	>100	N.T.	>100	N.T.	
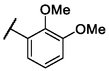 **11**	3.35	>100	N.T.	27.6 ± 4.1(14.4)	397.4	
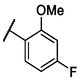 **12**	3.73	>100	N.T.	>100	N.T.	
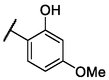 **13**	3.12	>100	N.T.	10.0 ± 0.4 (9.2)	91.8	
**Bzn**	N.D.	3.8 (625)	2381	N.T.	N.T.	
**AmB**	N.D.	N.T.	N.T.	0.25 ± 0.04 (1455)	363.8	

*Source* *:* clogP data were obtained from the SwissADME website.

Abbreviations: N.T.,  not tested; N.D., not determined; Bzn, benznidazole, used as a reference drug (positive control) in the assays against *T.* *cruzi*. AmB, amphotericin B, used as a reference drug (positive control) in the assays against *L.* *infantum*.

a IC_50_ value for the inhibition of the growth of *T. cruzi* in L929 cells (mean of at least two independent determinations, *p* < 0.05), SI, selectivity index = CC_50_/IC_50_ in L929 cells.

b CC_50_ value measured in L929 cells (mean of at least two independent determinations, *p* < 0.05).

c IC_50_ value for the inhibition of the growth of *L. infantum* in THP‐1 macrophages (mean of at least two independent determinations, *p* < 0.05), SI, selectivity index =  CC_50_/IC_50_ in THP‐1 cells.

d CC_50_ value measured in THP‐1 macrophages (mean of at least two independent determinations, *p* < 0.05).

From a structure–activity perspective, the removal or positional alteration of methoxy groups can modify several physicochemical and molecular properties, including the electronic distribution of the molecule, steric environment, lipophilicity, hydrogen‐bonding capacity, and potentially its preferred conformation. Such changes may consequently influence molecular recognition and interactions with the biological target, as well as other processes relevant to antiparasitic activity, such as cellular uptake, membrane permeability, or intracellular distribution. Although we cannot determine which of these factors is primarily responsible for the observed loss of potency in the absence of mechanistic and biophysical studies, these effects provide a plausible explanation for the sensitivity of antiparasitic activity to the position and presence of the methoxy substituents.

The present results evidence the critical role played by the methoxyl groups and their substituent positions on the benzene ring regarding antiparasitic activities, suggesting their significance as a pharmacophoric group for this chemical series. Specifically, the 2,4‐dimethoxy pattern proved essential for potent anti‐*T. cruzi* activity. However, removal of the *ortho*‐methoxy group was tolerated against *L. infantum* and could potentially improve metabolic stability, given that a methoxy group at the *ortho* position relative to a carbonyl is generally more susceptible to O─Me bond cleavage [28]. Therefore, this modification was retained for further evaluation, incorporating a pyridinyl moiety onto the phenyl ring to potentially enhance overall metabolic stability [27]. These findings are summarized in Table 3.

**TABLE 3:** In vitro anti‐*T. cruzi* and anti‐*L. infantum* activities and clogP values of pyridinyl derivatives **14–19**.

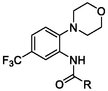						
Compound	clogP	**IC**_**50**_ ***T. cruzi,*** **µM** **(SI)** a	**CC**_**50**_ **L929, µM** b	**IC**_**50**_ ***L. infantum,*** **µM** **(SI)** c	**CC**_**50**_ **THP‐1, µM** d	
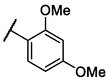 **1**	3.32	0.76 ± 0.02 (109.5)	82.7	10.7 ± 1.2 (36.3)	390.4	
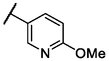 **14**	2.85	82.1 ± 23.9 (1.9)	157.3	10.8 ± 1.7(100.7)	1090.3	
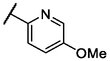 **15**	2.83	>100	N.T.	17.1 ± 0.6 (7.3)	124.8	
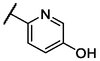 **16**	2.26	12.5 ± 3.3(4.3)	53.1	17.9 ± 0.1 (4.4)	78.1	
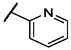 **17**	2.87	>100	N.T.	>100	N.T.	
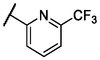 **18**	3.81	>100	N.T.	54.9 ± 6.0	N.T.	
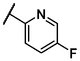 **19**	2.97	>100	N.T.	>100	N.T.	
**Bzn**	N.D.	3.8 (625)	2381	N.T.	N.T.	
**AmB**	N.D.	N.T.	N.T.	0.25 ± 0.04 (1455)	363.8	

*Source:* clogP data was obtained from the SwissADME website.

Abbreviations: N.T., not tested; N.D., not determined, Bzn, benznidazole, used as a reference drug (positive control) in the assays against *T.* *cruzi*. AmB, amphotericin B, used as a reference drug (positive control) in the assays against *L.* *infantum*.

a IC_50_ value for the inhibition of the growth of *T. cruzi* in L929 cells (mean of at least two independent determinations, *p* < 0.05), SI, selectivity index = CC_50_/IC_50_ in L929 cells.

b CC_50_ value measured in L929 cells (mean of at least two independent determinations, *p* < 0.05).

c IC_50_ value for the inhibition of the growth of *L. infantum* in THP‐1 macrophages (mean of at least two independent determinations, *p* < 0.05), SI, selectivity index = CC_50_/IC_50_ in THP‐1 cells.

d CC_50_ value measured in THP‐1 macrophages (mean of at least two independent determinations, *p* < 0.05).

Our initial investigation into the pyridinyl moiety focused on the position of the endocyclic nitrogen and its potential to reduce lipophilicity. We evaluated nitrogen insertion at positions 3 (compound **14**) and 2 (compound **15**) relative to the carbonyl group. Against *T. cruzi*, both modifications significantly decreased the potency (IC_50_ = 82.1 µM for **14** and IC_50_ > 100 µM for **15**). Conversely, for anti‐*L. infantum* activity, the endocyclic nitrogen at position 3 (compound **14**) was preferred (IC_50_ = 10.8 µM) over position 2 (**15**; IC_50_ = 17.1 µM). This preference resulted in a substantially higher SI than the initial hit **1** (SI = 100.7 for **14** vs. SI = 36.3 for **1**) while also achieving a favorable reduction in lipophilicity (clogP = 2.85 for **14** vs. clogP = 3.32 for **1**). The next step involved evaluating the replacement of the methoxy group with a more polar group, specifically the hydroxyl group (**16**). This modification improved biological activity against *T. cruzi* (IC_50_ = 12.5 µM), relative to its methoxy analog (**15**). In contrast, anti‐*L. infantum* activity remained comparable (IC_50_ = 17.9 µM for **16** vs. IC_50_ = 17.1 µM for **15**). Finally, further modifications on the pyridinyl fragment were explored, including the removal of the methoxy group (**17**) and substitution with other groups, such as CF_3_ (**18**) and F (**19**). Unfortunately, these modifications led to a loss of activity against both parasites, with all derivatives being inactive against *T. cruzi* at the maximum tested concentration (IC_50_ > 100 µM). Among these derivatives, **18** showed the highest residual potency against *L. infantum* (IC_50_ = 54.9 µM).

Overall, these results validate the hypothesis that 2,4‐dimethoxy groups on the benzamide fragment are essential and must be retained for potent anti‐*T. cruzi* activity. For anti‐*L. infantum* activity, however, the introduction of an endocyclic nitrogen (compound **14**) markedly increased selectivity while maintaining potency comparable to the initial hit **1**.

### 2.4 Chemical Modifications on the Central Phenyl Ring

The next set of modifications focused on the central phenyl ring, starting with the replacement of the trifluoromethyl group with other substituents and alterations to the nature of the phenyl ring. The same synthetic pathway used for compound **1** was employed to obtain these derivatives (Scheme 4). The synthesis began with an aromatic nucleophilic substitution on the aryl halide with morpholine, followed by nitro reduction under two different conditions: catalytic hydrogenation with Pd/C or treatment with SnCl_2_. The resulting aniline was then coupled with the corresponding carboxylic acid using HBTU to afford the desired compounds.

**SCHEME 4:**
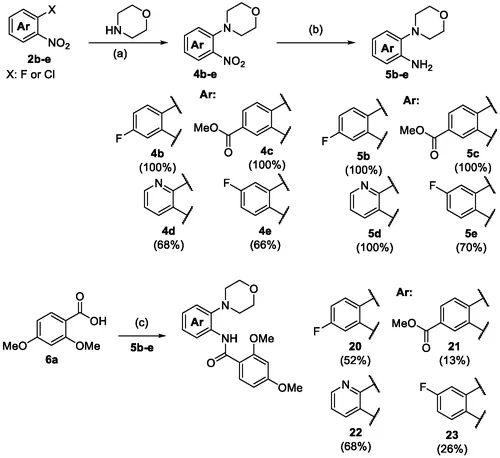
Synthesis of derivatives compounds **20**–**23**. Reagents and conditions: (a) Et_3_N, DMF, 70 °C, 68%–100% // For **4e**: Na_2_CO_3_, toluene, 70 °C, 65 h, 66%; (b) H_2_, Pd/C, AcOEt/MeOH (1:1), r.t., 100% // **5e**: SnCl_2_, AcOEt, reflux, 1 h, 70%; (c) HBTU, Et_3_N, DMF, 80 °C, 13%–68%.

The antiparasitic activity of the compounds with these modifications is presented in Table 4, along with their theoretical clogP values. The initial modifications on this fragment involved replacing the trifluoromethyl group with other less lipophilic electron‐withdrawing groups (EWG). This was done to reduce the lipophilicity and the electronic density of the phenyl ring, given its known susceptibility to oxidative metabolism. First, the trifluoromethyl group was replaced with a fluorine atom (compound **20**). This resulted in a 4.7‐fold loss of potency against *T. cruzi* compared to the initial hit **1** (IC_50_ = 3.58 µM) but showed increased selectivity (SI = 264.3). Despite the reduction in activity against *T. cruzi*, **20** still possesses biological activity equivalent to that of benznidazole (IC_50_ = 3.8 µM), the reference drug for Chagas disease. Against *L. infantum*, **20** showed similar potency to hit **1** (IC_50_ = 12.8 µM for **20** vs. IC_50_ = 10.7 µM for hit **1**) but with a significant decrease in selectivity (SI = 18.6 for **20** vs. SI = 36.3 for hit **1**). The trifluoromethyl group was then replaced by a methyl ester (compound **21**), a mesomeric EWG. This led to an 8‐fold decrease in potency against *L. infantum* (IC_50_ = 89.8 µM) and a complete loss of activity against *T. cruzi* (IC_50_ > 100 µM). Replacement of the phenyl ring with a pyridinyl ring was also evaluated, but this modification similarly resulted in a loss of activity against both parasites (IC_50_ > 100 µM). Since **20** showed relevant activity against *T. cruzi*, the position of the substituent was evaluated by moving the fluorine atom from position 4–5 (compound **23**). This abolished anti‐*T. cruzi* activity (IC_50_ > 100 µM) but maintained potency against *L. infantum* (IC_50_ = 10.4 µM) while increasing selectivity (SI = 103.2).

**TABLE 4:** In vitro anti‐*T. cruzi* and anti‐*L. infantum* activities and clogP values of compounds **20–23**.

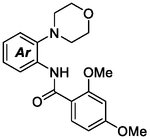					
Ar	clogP	**IC**_**50**_ ***T. cruzi,*** **µM** **(SI)** a	**CC**_**50**_ **L929, µM** b	**IC**_**50**_ ***L. infantum,*** **µM** **(SI)** c	**CC**_**50**_ **THP‐1, µM** d
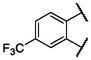 **1**	3.32	0.76 ± 0.02 (109.5)	82.7	10.7 ± 1.2 (36.3)	390.4
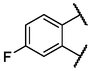 **20**	2.66	3.58 ± 1.30(264.3)	>555	12.8 ± 1.3 (18.6)	237.2
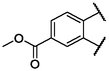 **21**	2.30	>100	N.T.	89.8 ± 1.3	N.T.
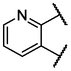 **22**	1.87	>100	N.T.	>100	N.T.
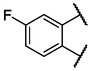 **23**	2.66	>100	N.T.	10.4 ± 2,8 (103.2)	1074.1
**Bzn**	N.D.	3.8 (625)	2381	N.T.	N.T.
**AmB**	N.D.	N.T.	N.T.	0.25 ± 0.04 (1455)	363.8

*Source:* clogP data was obtained from the SwissADME website.

Abbreviations: N.T., not tested; N.D., not determined; Bzn, benznidazole, used as a reference drug (positive control) in the assays against *T.* *cruzi*. AmB, amphotericin B, used as a reference drug (positive control) in the assays against *L.* *infantum*.

a IC_50_ value for the inhibition of the growth of *T. cruzi* in L929 cells (mean of at least two independent determinations, *p* < 0.05), SI, selectivity index = CC_50_/IC_50_ in L929 cells.

b CC_50_ value measured in L929 cells (mean of at least two independent determinations, *p* < 0.05).

c IC_50_ value for the inhibition of the growth of *L. infantum* in THP‐1 macrophages (mean of at least two independent determinations, *p* < 0.05), SI, selectivity index = CC_50_/IC_50_ in THP‐1 cells.

d CC_50_ value measured in THP‐1 macrophages (mean of at least two independent determinations, *p* < 0.05).

Compounds **24** and **25** were synthesized according to Scheme 5. The synthesis of **24** began with an aromatic nucleophilic substitution on fluoride **2f**, followed by a reduction using catalytic hydrogenation. The final product was then obtained by amidation using the corresponding acyl chloride. Compound **25**, in contrast, was synthesized by coupling aniline **5b** with carboxylic acid **6i** using HBTU‐mediated amide formation. Compound **24**, a constitutional isomer of **1**, was designed to evaluate the influence of varying the position of the morpholine moiety on the central phenyl ring. This modification abolished anti‐*T. cruzi* activity (IC_50_ > 100 µM) but increased anti‐*L. infantum* activity (IC_50_ = 7.6 µM) with a relatively high selectivity index (SI = 81.3) when compared to the initial hit **1** (Table 5). Compound **25** was designed by combining the central fragment of **20** (fluorine derivative), which was the most active derivative against *T. cruzi* (IC_50_ of 3.58 μM, Table 4), with the benzamide fragment of **16** (hydroxypyridine derivative), a derivative that showed a significant reduction in lipophilicity and moderate activity against *T. cruzi* and *L. infantum* (IC_50_ of 12.5 and 17.9 μM, respectively, Table 3). However, **25** exhibited low activity (IC_50_ > 100 µM) against both parasites (Table 5).

**SCHEME 5:**
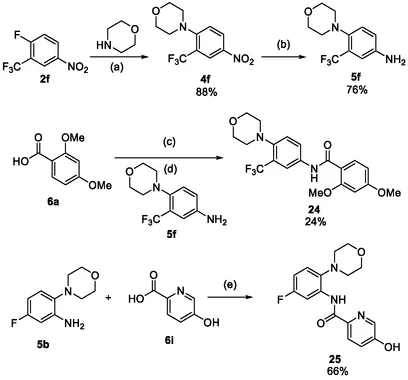
Synthesis of derivatives **24** and **25**. Reagents and conditions: (a) Et_3_N, DMF, 70 °C, 88%; (b) H_2_, Pd/C, AcOEt/MeOH (1:1), r.t., 24 h, 76%; (c) oxalyl chloride, DMF (cat.), DCM, r.t., Ar atm., 2 h; (d) Et_3_N, DCM, r.t., Ar atm., 116 h, 24%; (e) HBTU, Et_3_N, DMF, 70 °C, 22 h, 66%.

**TABLE 5:** In vitro anti‐*T. cruzi* and anti‐*L. infantum* activities and clogP values of **24** and **25**.

Compound	clogP	IC_50_ ***T. cruzi,*** **µM** (SI)^a^	CC_50_ L929, µM^b^	IC_50_ ***L.*** ***infantum*** **,** **µM** (SI)^c^	CC_50_ THP‐1, µM^d^
**24**	3.39	>100	N.T.	7.6 ± 0.2 (81.3)	614.2
**25**	1.61	>100	N.T.	>100	N.T.
**Bzn**	N.D.	3.8 (625)	2381	N.T.	N.T.
**AmB**	N.D.	N.T.	N.T.	0.25 ± 0.04 (1455)	363.8

*Source:* clogP data was obtained from the SwissADME website.

Abbreviations: N.T., not tested, N.D., not determined; Bzn, benznidazole, used as a reference drug (positive control) in the assays against *T.* *cruzi*. AmB, amphotericin B, used as a reference drug (positive control) in the assays against *L.* *infantum*.

a IC_50_ value for the inhibition of the growth of *T. cruzi* in L929 cells (mean of at least two independent determinations, *p* < 0.05), SI, selectivity index = CC_50_/IC_50_ in L929 cells.

b CC_50_ value measured in L929 cells (mean of at least two independent determinations, *p* < 0.05).

c IC_50_ value for the inhibition of the growth of *L. infantum* in THP‐1 macrophages (mean of at least two independent determinations, *p* < 0.05), SI, selectivity index = CC_50_/IC_50_ in THP‐1 cells.

d CC_50_ value measured in THP‐1 macrophages (mean of at least two independent determinations, *p* < 0.05).

This SAR reiterates the greater resistance of *T. cruzi* to chemical modifications on the central phenyl ring, while *L. infantum* was more sensitive, leading to derivatives with IC_50_ values equivalent to the initial hit **1**.

### 2.5 Chemical Modifications on the Morpholine Fragment

The final set of modifications targeted the morpholine fragment by either replacing it with other heterocyclic amines or removing the fragment entirely. The synthesis of these compounds is reported in Scheme 6. The synthetic route began with the aromatic nucleophilic substitution of the aryl fluoride **2a** with the chosen heterocyclic amine (thiomorpholine, *N*‐methylpiperazine, and piperazine) to afford **4g‐i**. The piperazine derivative **4i** was then protected using Boc_2_O. Subsequently, the nitro intermediates **4g‐i** were reduced via catalytic hydrogenation to afford the aniline intermediates **5g‐i**. The final target compounds were obtained through HBTU‐mediated amide coupling with the corresponding carboxylic acid or by reaction with the appropriate acyl halide. Compound **29** was then deprotected using trifluoroacetic acid (TFA).

**SCHEME 6:**
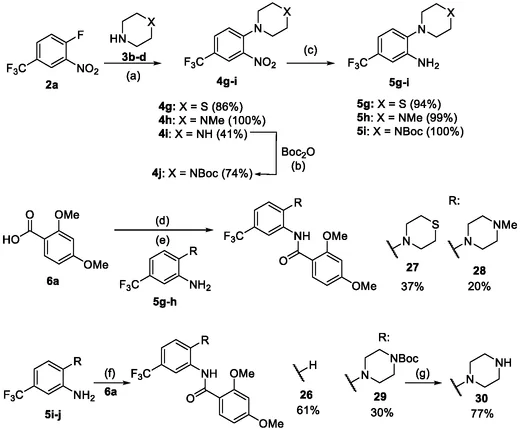
Synthesis of derivatives compounds **26**–**30**. Reagents and conditions: (a) Et_3_N, DMF, 70 °C, 41%–100%; (b) Et_3_N, DCM, r.t., 21 h, 74%; (c) H_2_, Pd/C, AcOEt/MeOH (1:1), r.t., 94%–100%; (d) oxalyl chloride, DMF (cat.), DCM, r.t., Ar atm.; (e) Et_3_N, DMF, DCM, r.t., 20%–37%; (f) HBTU, Et_3_N, DMF, 80 °C, 30%–61%; (g) TFA, DCM, r.t., 20 h, 77%.

The biological activities of compounds featuring modifications on the morpholine fragment are summarized in Table 6. To assess the influence of the heterocyclic ring, morpholine was removed (compound **26**). This resulted in a substantial decrease in potency, specifically a 5‐fold reduction against *L. infantum* (IC_50_ = 53.2 µM) and the complete loss of anti‐*T. cruzi* activity (IC_50_ > 100 µM). This clearly indicates that the heterocyclic ring is essential for the observed antiparasitic activity of **1**. Further investigation into steric and electronic effects involved replacing the oxygen atom with less electronegative groups to diminish the inductive electron‐withdrawing effect. Compounds were synthesized using a sulfur atom (**27**) and an *N*‐methyl group (**28**). Both modifications exhibited low activity against both parasites (IC_50_ > 83 µM). The effect of increasing ring polarity was explored by substituting the oxygen atom with an N‐H group (**30**), which potentially enhances interactions via hydrogen bonding. Both the final product (**30**) and its protected intermediate (**29**) reduced anti‐*T. cruzi* potency (IC_50_ = 82.8 µM for **29** and IC_50_ = 21.0 µM for **30**). Against *L. infantum*, **30** showed a 2.7‐fold decrease in potency (IC_50_ = 28.6 µM), whereas the *N*‐Boc‐protected intermediate (**29**) displayed activity equipotent to the hit compound **1** (IC_50_ = 10.4 µM) but with a substantially increased selectivity (SI > 70). Overall, these data suggest that morpholine plays an essential role in the anti‐*T. cruzi* activity. For anti‐*L. infantum* activity, while the heterocyclic ring remains essential, the *N*‐Boc‐piperazine derivative **29** proved to be the more favorable modification for achieving moderate biological potency and high selectivity**.**

**TABLE 6:** In vitro anti‐*T. cruzi* and anti‐*L. infantum* activities and clogP values of **26–30**.

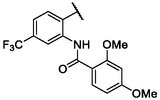					
Compound	clog P	**IC**_**50**_ ***T. cruzi,* µM** (**SI)** a	**CC**_**50**_ **L929, µM** b	**IC**_**50**_ ***L. infantum,*** **µM** (**SI)** c	**CC**_**50**_ **THP‐1, µM** d
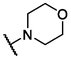 **1**	3.32	0.76 ± 0.02 (109.5)	82.7	10.7 ± 1.2 (36.3)	390.4
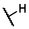 **26**	3.63	>100	N.T.	53.2 ± 5.8	N.T.
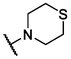 **27**	3.96	>100	N.T.	83.2 ± 3.1	N.T.
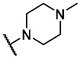 **28**	3.28	>100	N.T.	>100	N.T.
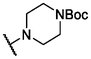 **29**	3.99	82.8	N.T.	10.4 ± 1.7 (>70)	>700
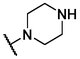 **30**	2.99	21.0 ± 0.7(2.2)	45.6	28.6 ± 2.0	N.T.
**Bzn**	N.D.	3.8 (625)	2381	N.T.	N.T.
**AmB**	N.D.	N.T.	N.T.	0.25 ± 0.04 (1455)	363.8

*Source:* clogP data was obtained from the SwissADME website.

Abbreviations: N.T., not tested; N.D., not determined; Bzn, benznidazole, used as a reference drug (positive control) in the assays against *T.* *cruzi*. AmB, amphotericin B, used as a reference drug (positive control) in the assays against *L.* *infantum*.

a IC_50_ value for the inhibition of the growth of *T. cruzi* in L929 cells (mean of at least two independent determinations, *p* < 0.05), SI, selectivity index = CC_50_/IC_50_ in L929 cells.

b CC_50_ value measured in L929 cells (mean of at least two independent determinations, *p* < 0.05).

c IC_50_ value for the inhibition of the growth of *L. infantum* in THP‐1 macrophages (mean of at least two independent determinations, *p* < 0.05), SI, selectivity index = CC_50_/IC_50_ in THP‐1 cells.

d CC_50_ value measured in THP‐1 macrophages (mean of at least two independent determinations, *p* < 0.05).

### 2.6 In Vitro ADME Profile of 16 and 20

To evaluate the impact of reducing lipophilicity and removing metabolic soft spots, compounds **16** and **20** were chosen for ADME profile assessment (Table 7). These compounds featured respective chemical modifications in the benzamide and central phenyl ring fragments of the initial hit **1** and showed moderate antiparasitic activities. Despite showing lower lipophilicity than **1**, both **16** and **20** remain highly lipophilic, evidenced by logD values exceeding 3.9. Both derivatives demonstrated improved aqueous solubility compared to **1** at pH 7.4. Furthermore, **16** was more soluble than **1** at pH 2.0, a result likely due to its lower overall lipophilicity and the presence of the pyridinic nitrogen, which is capable of protonation in acidic media. Gastrointestinal permeability was also enhanced for both **16** and **20**, as indicated by values significantly greater than **1**. However, their metabolic stability in the mouse model revealed contrasting trends: **16**, which is more lipophilic than **20** but lacks the methoxy groups in the benzamide fragment (potential soft spots), exhibited excellent metabolic stability, achieving an intrinsic clearance and microsomal half‐life in MLM 6.5 times better than hit **1**. Conversely, compound **20**, although less lipophilic than **16** but retaining the methoxy groups on the benzamide fragment, showed poor metabolic stability in mice, performing even worse than hit **1**. These data strongly suggest that the methoxy groups on the benzamide fragment act as critical metabolic soft spots for this chemical class, exerting a more significant impact on stability than the class's inherent high lipophilicity. Finally, **16** maintained the same favorable metabolic stability profile in human liver microsomes (HLM), whereas **20** showed better stability in human microsomes compared to mouse microsomes.

**TABLE 7:** ADME in vitro properties of **16** and **20**.

Cp	**logD** a	**KS, µM** b		**GI** ***P***_**e**_, **10** ^**−6**^ **cm/s** c	***t***_**1/2**_ **MLM, min** d	**MLM Cl**_**int,**_ **µL/min/mg** e	***t***_**1/2**_ **HLM, min** d	**HLM Cl**_**int**_, **µL/min/mg** e
		pH 2.0	pH 7.4					
**1**	4.60	4.10	6.15	0.78	15.61	177.60	N.T.	N.T.
**16**	4.29	5.13	23.3	15.7	101.93	27.20	108.30	25.60
**20**	3.94	20.36	18.97	15.1	6.24	444.40	14.91	186.00

Abbreviation: N.T., not tested.

a Experimental lipophilicity, determined at pH, 7.4.

b Kinetic solubility (µM).

c Gastrointestinal permeability. *P*_e_ < 1.5.10^−6^ cm/s: low permeability, *P*_e_ > 1.5.10^−6^ cm/s: high permeability.

d Microsomal half‐life determined in Mouse Liver Microsomes (MLM) or Human Liver Microsomes (HLM), in presence of NADPH cofactor.

e Intrinsic clearance determined in MLM or HLM, in presence of NADPH cofactor.

### 2.7 Antitrypanosomal Activity of 1 Against Different *T. cruzi* DTUs

*T. cruzi* exhibits significant genetic variability, which serves as the basis for its classification into seven distinct lineages: the six Discrete Typing Units (DTUs) (TcI–TcVI) and the genotype TcBat [29, 30]. It is believed that this genetic diversity is linked to the extensive range of clinical outcomes observed in patients with Chagas disease [31].

The initial hit **1** was the only compound in this class to demonstrate anti‐*T. cruzi* (Tulahuen, DTU TcVI) activity below 1 mM. It was five times more active than benznidazole and showed a potency level recommended for a lead candidate against Chagas disease [32, 33]. Considering the variable efficacy of anti‐Chagasic drugs across different *T. cruzi* DTUs and strains and in accordance with established recommendations for anti‐Chagasic drug discovery, compound **1** was subsequently evaluated in vitro *a*gainst the intracellular amastigote forms of two additional *T. cruzi* strains: the Y strain (DTU TcII) and the CL‐Brener strain (DTU TcVI). Compound **1** demonstrated approximately 2.5 times greater activity against the Y and CL‐Brener strains compared to the Tulahuen *T. cruzi* strain, with IC_50_ 0.31 and 0.29 μM, respectively (Table 8). This data clearly demonstrates its potent, broad‐spectrum activity against different *T. cruzi* DTUs, further highlighting the promising anti‐*T. cruzi* properties of **1**. This broad efficacy is particularly significant given that the clinical outcome and treatment response in Chagas disease often vary substantially depending on the infecting DTU. Therefore, the ability of **1** to maintain submicromolar activity across TcII (Y strain) and TcVI (CL‐Brener and Tulahuen strains) is a critical indicator of its potential as a universally effective therapeutic agent, addressing a major challenge in anti‐Chagas drug development.

**TABLE 8:** In vitro anti‐*T. cruzi* activity of **1** against Y and CL‐Brener strains.

Compound	***T. cruzi*** **Tulahuen** **IC**_**50**_ (**SI)** a	***T. cruzi*** **Y IC**_**50**_, (**SI)** b	***T. cruzi*** **CL‐Brener IC**_**50**_ (**SI)** c	**L6 CC**_**50**_, **µM** d
**1**	0.76 ± 0.02 (109.5)	0.31 ± 0.17 (>89)	0.29 ± 0.03 (>96)	>27.7
**Bzn**	3.8 (625)	6.10 ± 0.65 (>33)	2.08 ± 0.03 (>96)	>200

Abbreviation: Bzn, benznidazole, used as a reference drug (positive control) in the assays against *T.* *cruzi*.

a IC_50_ value for the inhibition of the growth of *T. cruzi* (Tulahuen strain) in L929 cells (mean of at least two independent determinations, *p* < 0.05), SI, selectivity index = CC_50_/IC_50_ in L929 cells.

b IC_50_ value for the inhibition of the growth of *T. cruzi* (Y strain) in L6 cells (mean of at least two independent determinations, *p* < 0.05), SI, selectivity index = CC_50_/IC_50_ in L6 cells.

c IC_50_ value for the inhibition of the growth of *T. cruzi* (CL Brener strain) in L6 cells (mean of at least two independent determinations, *p* < 0.05), SI, selectivity index = CC_50_/IC_50_ in L6 cells.

d CC_50_ value measured in L6 cells (mean of at least two independent determinations, *p* < 0.05).

## 3 Conclusion

This work successfully described the synthesis and systematic biological evaluation of twenty‐five novel compounds, with twenty‐four being derivatives of the initial hit **1**. Our studies showed that while hit **1** exhibited high potency (IC_50_ < 1 μM) against different *T. cruzi* strains belonging to two distinct DTUs and moderate activity (IC_50_ of 10 μM) against *L. infantum*, its utility as a lead candidate is critically limited by a poor ADME profile. Structural modifications targeting the benzamide, central phenyl ring, and morpholine fragments provided crucial SAR insights. While most derivatives showed diminished potency, key breakthroughs were achieved. Against *T. cruzi*, **20**, which incorporates modifications on the phenyl fragment, emerged as the most active derivative. Crucially, this compound demonstrated significant improvements in selectivity, lipophilicity, and aqueous solubility compared to the initial hit **1**. Despite a slight decrease in potency relative to hit **1**, compound **20** exhibited anti‐*T. cruzi* potency equivalent to that of benznidazole. Against *L. infantum*, optimization efforts yielded five equipotent analogs, with **24** notably displaying both increased potency and enhanced selectivity, establishing a new benchmark within the class for leishmaniasis. The central challenge underscored was that none of the synthesized analogs achieved the requisite balance of high antiparasitic activity (IC_50_ < 1 μM) and optimized ADME properties for a viable lead candidate for Chagas disease or visceral leishmaniasis. Nevertheless, the findings provide a clear path forward as **16** is highlighted as a critical structural template, possessing significantly optimized ADME characteristics, particularly microsomal metabolic stability and permeability. This systematic exploration of the morpholinephenylbenzamides class against Chagas disease and visceral leishmaniasis emphasizes the inherent difficulties in drug discovery, which is the simultaneous optimization of potency and pharmacokinetics. Future efforts should therefore be directed at rationally hybridizing the high‐potency features of **1**, **20**, and **24** with the ADME‐favorable scaffold elements identified in **16** to furnish a bona fide lead candidate.

## 4 Materials and Methods

### 4.1 Synthesis

#### 4.1.1 General Information

Unless specified, all reactions were performed under magnetic stirring, and commercial reagents and solvents were used without further purification. Dichloromethane (DCM), triethylamine (Et_3_N), and dimethylformamide (DMF) were dried with molecular sieves. Ethyl acetate (EtOAc) and hexane (Hex) were previously distilled. Flash column chromatography was performed using either Aldrich silica gel (35–70 mesh) or Macherey–Nagel silica gel (230–400 mesh ASTM). Analytical thin‐layer chromatography was performed on chromatography aluminum sheets impregnated with silica‐gel 60F_254_ (Sigma–Aldrich), and plate revelation was achieved using UV light (254 nm) and/or iodine atmosphere. ^1^H, proton‐decoupled ^13^C and ^13^C APT NMR spectra were acquired in CDCl_3_, CD_3_OD, or DMSO‐*d*_6_ at 400 MHz (^1^H) and 101 MHz (^13^C and ^13^C APT) (Bruker Ascend 400). Chemical shifts (*δ*) are reported in ppm using the residual solvent peak as an internal standard (CDCl_3_: 7.26 ppm, DMSO‐*d*_6_: 2.50 ppm, TMS: 0.00 ppm for ^1^H NMR spectra, and CDCl_3_: 77.16 ppm, DMSO‐*d*_6_: 39.52 ppm for ^13^C and ^13^C APT NMR spectra). Peak multiplicity was reported using the following abbreviations: s = singlet, d = doublet, t = triplet, q = quartet, dd = doublet of doublets, ddd = doublet of doublets of doublets, td = triplet of doublets, m = multiplet. The multiplicity is followed by the coupling constant(s) in Hz and integration. Exchangeable protons were not observable on some NMR spectra. For ^13^C APT NMR spectra, peaks pointing upwards correspond to primary and tertiary carbons, and peaks pointing downwards correspond to secondary and quaternary carbons. High‐resolution mass spectrometry (HRMS) was measured using electrospray ionization (ESI) (Bruker Daltonics Corporation, Q‐TOF geometry Impact II). High‐resolution mass spectrometry (HRMS) was performed on an Impact II Q‐TOF spectrometer (Bruker Daltonics, Germany) equipped with an electrospray ionization (ESI) source and operated in the positive ion mode. Instrument parameters were as follows: capillary voltage 4500 V, endplate offset 500 V, nebulizer pressure 0.4 bar, drying gas flow 4 L/min at 180 °C. Data were acquired at 1.0 Hz over the mass range of 50–700 m/z. External calibration was carried out by syringe infusion of sodium formate (10 mmol · L^−1^, isopropanol to water 1:1, v:v) at 10 µL/min. Samples were resuspended in 500 µL MeOH; an aliquot of 50 µL was diluted in 1000 µL MeOH and analyzed by direct infusion into the mass spectrometer.

#### 4.1.2 General Procedure A: Synthesis of Intermediates 4a–i

In a solution of amine **3a–i** in DMF, the base and aryl halide **2a–i** were added. The specific conditions, reagents, and concentrations used in each reaction are described in the subsections below. The reaction mixture was stirred under heating and monitored by TLC. Upon completion, the reaction was quenched by adding cold distilled water (25 mL) at room temperature. The resulting precipitate was filtered, and the aqueous phase was extracted with ethyl acetate (2 × 30 mL). The organic layers were combined with the solid, dried over anhydrous Na_2_SO_4_, filtered, concentrated, and dried under vacuum. The crude product was then recrystallized from hexane and DCM, affording the desired compound.

##### 4.1.2.1 Synthesis of Intermediate 4a

In a solution of fluoride **2a** (1.51 g, 7.22 mmol) in DMF (2 mL), morpholine **3a** (638.4 mg, 7.32 mmol) and potassium carbonate (1.98 g, 14.32 mmol) were added. The reaction mixture was stirred at 60 °C and followed the general procedure A, affording compound **4a** in 97% yield after 24 h of reaction.

**(4a)** Orange oil, 1.99 g, 97%. **^1^H NMR (400 MHz, CDCl_3_):** *δ* 8.06 (d, *J* = 2.5 Hz, 1H), 7.69 (dd, *J* = 8.7, 2.4 Hz, 1H), 7.17 (d, *J* = 8.7 Hz, 1H), 3.85 (t, *J* = 4.7 Hz, 4H), 3.14 (t, *J* = 4.7 Hz, 4H).

##### 4.1.2.2 Synthesis of Intermediate 4b

In a solution of morpholine **3a** (193.4 mg, 2.22 mmol) in DMF (2 mL), anhydrous Et_3_N (3.33 mmol, 337 mg) and aryl halide **2b** (177.2 mg, 1.11 mmol) were added. The reaction mixture was stirred at 70 °C and followed the general procedure A, affording compound **4b** in 100% yield after 4 h of reaction.

**(4b)** Orange oil, 252.7 mg, 100%. **^1^H NMR (400 MHz, CDCl_3_): *δ*** 7.52 (dd, *J* = 7.9, 2.9 Hz, 1H), 7.27 (ddd, *J* = 9.1, 7.7, 3.0 Hz, 1H), 7.21 (dd, *J* = 9.0, 4.9 Hz, 1H), 3.84 (t, *J* = 4.6 Hz, 4H), 3.02 (t, *J* = 4.6 Hz, 4H).

##### 4.1.2.3 Synthesis of Intermediate 4c

In a solution of morpholine **3a** (144.3 mg, 1.66 mmol) in DMF (2 mL), anhydrous Et_3_N (2.26 mmol, 228.6 mg) and aryl halide **2c** (150.0 mg, 0.753 mmol) were added. The reaction mixture was stirred at 70 °C and followed the general procedure A, affording compound **4c** in 100% yield after 24 h of reaction.

**(4c)** Reddish‐orange oil, 222 mg, 100%. **^1^H NMR (400 MHz, CDCl_3_): *δ*** 7.81 (d, *J* = 1.7 Hz, 1H), 7.75 (d, *J* = 8.4 Hz, 1H), 7.70 (dd, *J* = 8.4, 1.7 Hz, 1H), 3.95 (s, 3H), 3.83 (t, *J* = 4.6 Hz, 4H), 3.08 (t, *J* = 4.7 Hz, 4H).

##### 4.1.2.4 Synthesis of Intermediate 4d

In a solution of morpholine **3a** (48.0 mg, 0.551 mmol) in DMF (2 mL), anhydrous Et_3_N (0.909 mmol, 92.0 mg) and aryl halide **2d** (70.0 mg, 0.442 mmol) were added. The reaction mixture was stirred at 70 °C and followed the general procedure A, affording compound **4d** in 68% yield after 48 h of reaction.

**(4d)** Yellow solid, 62.8 mg, 68%. **^1^H NMR (400 MHz, CDCl_3_): *δ*** 8.34 (dd, *J* = 4.5, 1.7 Hz, 1H), 8.13 (dd, *J* = 8.0, 1.8 Hz, 1H), 6.78 (dd, *J* = 8.0, 4.6 Hz, 1H), 3.80 (t, *J* = 4.8 Hz, 4H), 3.46 (t, *J* = 4.8 Hz, 4H).

##### 4.1.2.5 Synthesis of Intermediate 4f

In a solution of morpholine **3a** (0.906 mg, 9.56 mmol) in DMF (2 mL), anhydrous Et_3_N (14.34 mmol, 1.45 g) and aryl halide **2f** (1.0 g, 4.78 mmol) were added. The reaction mixture was stirred at 76 °C and followed the general procedure A, affording compound **4f** in 88% yield after 25 h of reaction.

**(4f)** Brown solid, 1.16 g, 88%. **^1^H NMR (400 MHz, CDCl_3_): *δ*** 8.53 (d, *J* = 2.7 Hz, 1H), 8.35 (dd, *J* = 9.0, 2.7 Hz, 1H), 7.30 (d, *J* = 8.9 Hz, 1H), 3.86 (t, *J* = 4.6 Hz, 4H), 3.12 (t, *J* = 4.6 Hz, 4H).

##### 4.1.2.6 Synthesis of Intermediate 4g

In a solution of thiomorpholine **3b** (136.2 mg, 1.08 mmol) in DMF (2 mL), anhydrous Et_3_N (2.15 mmol, 217.6 mg) and aryl halide **2a** (150.0 mg, 0.717 mmol) were added. The reaction mixture was stirred at 100 °C and followed the general procedure A, affording compound **4g** in 86% yield after 18 h of reaction.

**(4g)** Solid, 180.6 mg, 86%. **^1^H NMR (400 MHz, CDCl_3_): *δ*** 8.05 (d, *J* = 2.9 Hz, 1H), 7.68 (dd, *J* = 8.7, 2.3 Hz, 1H), 7.19 (d, *J* = 8.5 Hz, 1H), 3.41 (t, *J* = 5.1 Hz, 4H), 2.79 (t, *J* = 5.1 Hz, 4H).

##### 4.1.2.7 Synthesis of Intermediate 4h

In a solution of N‐methylpiperazine **3c** (287.3 mg, 2.87 mmol) in DMF (2 mL), Na_2_CO_3_ (3.23 mmol, 342.3 mg) and aryl halide **2a** (150.0 mg, 0.717 mmol) were added. The reaction mixture was stirred at 100 °C and followed the general procedure A, affording compound **4h** in 100% yield after 23 h of reaction.

**(4h)** Orange oil, 218.0 mg, 100%. **^1^H NMR (400 MHz, CDCl_3_): *δ*** 8.03 (d, *J* = 1.6 Hz, 1H), 7.65 (dd, *J* = 8.8, 2.3 Hz, 1H), 7.16 (d, *J* = 8.7 Hz, 1H), 3.18 (t, *J* = 4.9 Hz, 4H), 2.57 (t, *J* = 4.9 Hz, 4H), 2.35 (s, 3H).

##### 4.1.2.8 Synthesis of Intermediate 4i

In a solution of aryl halide **2a** (275.0 mg, 1.32 mmol) in DMF (3.0 mL), piperazine **3d** (227.4 mg, 2.64 mmol) and Et_3_N (3.96 mmol, 400.7 mg) were added. The reaction mixture was stirred at 60 °C and followed the general procedure A, affording compound **4i** in 41% yield after 3 h of reaction.

**(4i)** Yellow oil, 150.0 mg, 41%. **^1^H NMR (400 MHz, CDCl_3_): *δ*** 8.04 (d, *J* = 2.5 Hz, 1H), 7.66 (dd, *J* = 8.7, 2.3 Hz, 1H), 7.16 (d, *J* = 8.7 Hz, 1H), 3.16–3.12 (m, 4H), 3.05–3.01 (m, 4H).

#### 4.1.3 Procedure for the Synthesis of Intermediate 4e

In a solution of fluoride **2e** (170.1 mg, 1.07 mmol) in toluene (2 mL)**,** morpholine **3a** (92.5 mg, 1.07 mmol) was added. After 10 min, Na_2_CO_3_ (0.535 mmol, 56.7 mg) was added in an ice bath. After 10 min, the reaction mixture was heated to 75 °C and stirred for 45 h, monitoring the reaction by TLC (7:3 Hex/AcOEt). The reaction was quenched by cooling to room temperature, adding distilled water (25 mL), and extracting with toluene (20 mL). The organic phase was dried over anhydrous Na_2_SO_4_, filtered, concentrated, and dried. The obtained material was dissolved in toluene (2 mL) and reacted again with morpholine **3a** (0.214 mmol, 18.6 mg) and Na_2_CO_3_ (0.535 mmol, 56.7 mg). The reaction mixture was stirred at 65 °C for 20 h and quenched by cooling to room temperature, adding distilled water, and extracting first with toluene (25 mL) and then with DCM (2 × 20 mL). The three organic layers were combined, dried over anhydrous Na_2_SO_4_, filtered, and evaporated under reduced pressure. The crude material was purified by flash column chromatograph**y** on silica using 9:1 Hex/AcOEt as the eluent, affording compound **4e** in 66% yield.

**(4e)** Yellow oil, 158.7 mg, 66%. **^1^H NMR (400 MHz, CDCl_3_):** *δ* 7.90 (dd, *J* = 9.0, 6.0 Hz, 1H), 7.41–7.36 (m, 1H), 6.77 (dd, *J* = 10.6, 2.6 Hz, 1H), 6.72 (ddd, *J* = 8.9, 7.0, 2.6 Hz, 1H), 3.85 (t, *J* = 4.6 Hz, 4H), 3.06 (t, *J* = 4.7 Hz, 4H).

#### 4.1.4 Procedure for the Synthesis of Intermediate 4j

In a solution of intermediate **4i** (150.0 mg, 0.545 mmol) in DCM (2 mL), Et_3_N (0.600 mmol, 60.7 mg) and Boc_2_O (0.545 mmol, 119.0 mg) were added. The reaction mixture was stirred at room temperature and monitored by TLC (9:1 DCM/MeOH). After 21 h, the reaction was quenched by evaporating the solvent and adding distilled water (50 mL), yielding an insoluble oil that was washed with 50 mL of distilled water, concentrated, and dried. The supernatant was transferred to a separatory funnel and extracted with ethyl acetate (30 mL), and the organic phase was combined with the concentrated oil and washed with 2 M NaOH solution (2 × 25 mL). The organic layer was dried over anhydrous Na_2_SO_4_, filtered, concentrated, and dried, affording compound **4j** in 74% yield.

**(4j)** Red oil, 150.0 mg, 74%. **^1^H NMR (400 MHz, CDCl_3_): *δ*** 8.08 (d, *J* = 1.8 Hz, 1H), 7.69 (dd, *J* = 9.1, 1.9 Hz, 1H), 7.17 (d, *J* = 8.0 Hz, 1H), 3.61 (t, *J* = 5.1 Hz, 4H), 3.12 (s, 4H), 1.48 (s, 9H).

#### 4.1.5 General Procedure B for Nitro Group Reduction

In a solution of the nitro compound in AcOEt/MeOH (1:1), Pd/C was added, and H_2_ gas was bubbled through the solution until complete consumption of the starting nitro compound. The masses of reagents and volumes of solvents used for the preparation of each compound are described in the subsections below. The reaction was monitored by TLC and quenched by filtering off the Pd over Celite. The solid was then concentrated using a rotary evaporator and dried under vacuum, affording the desired product.

##### 4.1.5.1 Synthesis of intermediate 5a

In a solution of nitro compound **4a** (2.0836 g, 7.54 mmol) in MeOH (50 mL), Pd/C (208 mg) was added. The reaction mixture was stirred at room temperature and followed general procedure B, affording compound **5a** in 99% yield after 12 h.

**(5a)** Orange solid, 1.851 g, 99%. **^1^H NMR (400 MHz, DMSO‐d_6_): *δ*** 7.01 (dd, *J* = 8.2, 0.8 Hz, 1H), 6.97 (d, *J* = 2.5 Hz, 1H), 6.84 (ddd, *J* = 8.1, 2.3, 0.8 Hz, 1H), 5.18 (s, 2H), 3.76 (t, *J* = 4.6 Hz, 4H), 2.83 (t, *J* = 4.6 Hz, 4H).

##### 4.1.5.2 Synthesis of Intermediate 5b

In a solution of nitro compound **4b** (198.8 mg, 0.879 mmol) in AcOEt/MeOH (20 mL), Pd/C (19.8 mg) was added. The reaction mixture was stirred at room temperature and followed general procedure B, affording compound **5b** in 100% yield after 2.5 h.

**(5b)** Red solid, 172 mg, 100%. **^1^H NMR (400 MHz, CDCl_3_): *δ*** 6.93 (dd, *J* = 8.5, 5.7 Hz, 1H), 6.46–6.38 (m, 2H), 4.14 (s, 2H), 3.83 (t, *J* = 4.6 Hz, 4H), 2.86 (t, *J* = 4.6 Hz, 4H).

##### 4.1.5.3 Synthesis of Intermediate 5c

In a solution of nitro compound **4c** (295.2 mg, 1.109 mmol) in AcOEt/MeOH (14 mL), Pd/C (29.5 mg) was added. The reaction mixture was stirred at room temperature and followed general procedure B, affording compound **5c** in 100% yield after 1.5 h.

**(5c)** Pale pink solid, 263.9 mg, 100%. **^1^H NMR (400 MHz, CDCl_3_): *δ*** 7.70 (d, *J* = 1.9 Hz, 1H), 7.67 (dd, *J* = 8.2, 1.9 Hz, 2H), 6.70 (d, *J* = 8.2 Hz, 1H), 4.41 (s, 2H), 3.88–3.82 (m, 7H), 2.92 (t, *J* = 4.7 Hz, 4H).

##### 4.1.5.4 Synthesis of Intermediate 5d

In a solution of nitro compound **4d** (58.3 mg, 0.279 mmol) in AcOEt/MeOH (10 mL), Pd/C (5.8 mg) was added. The reaction mixture was stirred at room temperature and followed general procedure B, affording compound **5d** in 100% yield after 45 min.

**(5d)** Solid, 52.3 mg, 100%. **^1^H NMR (400 MHz, CDCl_3_):** *δ* 7.81 (dd, *J* = 4.8, 1.5 Hz, 1H), 6.95 (dd, *J* = 7.7, 1.5 Hz, 1H), 6.85 (dd, *J* = 7.7, 4.8 Hz, 1H), 3.86 (t, *J* = 4.6 Hz, 4H), 3.78 (*s*, 2H), 3.13 (t, *J* = 4.6 Hz, 4H).

##### 4.1.5.5 Synthesis of Intermediate 5f

In a solution of nitro compound **4f** (103.0 mg, 0.373 mmol) in ACN (3 mL) and H_2_O (0.3 mL), NiCl_2_ · H_2_O (0.187 mmol, 44.3 mg) was added and stirred for 10 min, followed by the slow addition of NaBH_4_ (1.12 mmol, 42.3 mg). The reaction mixture was stirred at room temperature for 19 h, after which additional NiCl_2_ · H_2_O (0.0373 mmol, 8.9 mg) and NaBH_4_ (0.124 mmol, 14.1 mg) were added. After 2 more hours, the reaction was quenched by filtration through filter paper, diluting the filtrate with AcOEt (30 mL) and washing with water (20 mL). The organic phase was dried over anhydrous Na_2_SO_4_, filtered, concentrated, and dried, yielding an impure material containing intermediates and the starting material. The mixture was then subjected to catalytic hydrogenation following general procedure B, affording compound **5f** in 76% yield after 24 h of reaction.

**(5f)** Dark brown solid, 70.0 mg, 76%. **^1^H NMR (400 MHz, CDCl_3_): *δ*** 7.19 (d, *J* = 8.4 Hz, 1H), 6.91 (d, *J* = 2.8 Hz, 1H), 6.81 (dd, *J* = 8.4, 3.0 Hz, 1H), 3.79 (t, *J* = 4.6 Hz, 4H), 3.73 (*s*, 2H), 2.83 (t, *J* = 4.6 Hz, 4H).

##### 4.1.5.6 Synthesis of Intermediate 5g

In a solution of nitro compound **4g** (173.8 mg, 0.595 mmol) in AcOEt/MeOH (20 mL), Pd/C (17.4 mg) was added. The reaction mixture was stirred at room temperature and followed general procedure B, affording compound **5g** in 94% yield after 24 h.

**(5g)** Orange solid, 147.2 mg, 94%. **^1^H NMR (400 MHz, CDCl_3_):** *δ* 7.02 (d, *J* = 8.1 Hz, 1H), 6.98 (dd, *J* = 8.4, 2.4 Hz, 1H), 6.94 (d, *J* = 2.2 Hz, 1H), 4.07 (s, 2H), 3.18–3.12 (m, 4H), 2.83–2.78 (m, 4H).

##### 4.1.5.7 Synthesis of Intermediate 5h

In a solution of nitro compound **4h** (192.8 mg, 0.667 mmol) in AcOEt/MeOH (22 mL), Pd/C (19.3 mg) was added. The reaction mixture was stirred at room temperature and followed general procedure B, affording compound **5h** in 99% yield after 24 h.

**(5h)** Red solid, 172.9 mg, 99%. **^1^H NMR (400 MHz, CDCl_3_): *δ*** 7.04 (d, *J* = 8.1 Hz, 1H), 6.98 (dd, *J* = 8.3, 2.6 Hz, 1H), 6.93 (d, *J* = 2.2 Hz, 1H), 4.06 (*s*, 2H), 2.97 (t, *J* = 4.8 Hz, 4H), 2.65–2.54 (m, 4H), 2.37 (*s*, 3H).

##### 4.1.5.8 Synthesis of Intermediate 5i

In a solution of nitro compound **4j** (135.0 mg, 0.360 mmol) in AcOEt/MeOH (7.2 mL), Pd/C (13.5 mg) was added. The reaction mixture was stirred at room temperature and followed general procedure B, affording compound **5i** in 100% yield after 29 h.

**(5i)** Red oil, 116.1 mg, 100%. **^1^H NMR (400 MHz, CDCl_3_): *δ*** 6.99 (d, *J* = 1.7 Hz, 2H), 6.95 (s, 1H), 4.08 (s, 2H), 3.57 (s, 4H), 2.87 (t, *J* = 5.2 Hz, 4H), 1.49 (s, 9H).

#### 4.1.6 Procedure for the Synthesis of Intermediate 5e

In a solution of nitro compound **4e** (158.7 mg, 0.702 mmol) in AcOEt (5 mL), tin(II) chloride (1.1 g, 5.62 mmol) was added. The reaction mixture was stirred under reflux for 1 h and monitored by TLC (9:1 AcOEt/Hex). The reaction was quenched by cooling to room temperature and adding 10 mL of potassium bicarbonate (KHCO_3_) solution. The formed precipitate was filtered, and the supernatant was diluted with 15 mL of distilled water and extracted with AcOEt (2 × 20 mL). The combined organic layers were dried over anhydrous Na_2_SO_4_, filtered, concentrated, and dried, affording compound **5e** in 70% yield.

**(5e)** Dark red oil, 96.0 mg, 70%. **^1^H NMR (400 MHz, CDCl_3_): *δ*** 6.73 (ddd, *J* = 10.1, 2.0, 1.1 Hz, 1H), 6.68–6.66 (m, 1H), 6.65–6.64 (m, 1H), 3.89–3.72 (m, 6H), 2.90 (t, *J* = 4.6 Hz, 4H).

#### 4.1.7 General Procedure C for Amide Formation Using HBTU

In a solution of aniline in DMF (2 mL), carboxylic acid, HBTU, and triethylamine were added. The masses of reagents used for each compound are detailed in the subsections below. The reaction mixture was stirred at 60 °C–90 °C, monitored by TLC, and quenched by adding 25 mL of cold distilled water at room temperature, forming a precipitate that was filtered. The solid was dissolved in DCM (25 mL) and extracted with 2 M NaOH solution (2 × 20 mL). The organic phase was dried over anhydrous Na_2_SO_4_, filtered, concentrated, and dried under reduced pressure. The crude product was recrystallized from methanol/water, filtered, dried, and further purified by flash column chromatography on silica using 70% hexane/30% AcOEt as eluent. Finally, grease was removed with hexane, and the compound was dried under high vacuum, affording the final product.

##### 4.1.7.1 Synthesis of Hit Compound 1

In a solution of aniline 5a (30.0 mg, 0.122 mmol) in DMF (2 mL), carboxylic acid 6a (0.244 mmol, 44.5 mg), HBTU (0.244 mmol, 92.0 mg), and triethylamine (0.366 mmol, 37.0 mg, 51 μL) were added. The reaction mixture was stirred at 80 °C and followed general procedure C, affording hit **1** in 29% yield after 139 h.

**(1)** White solid, 14.6 mg, 29%. **^1^H NMR (400 MHz, CDCl_3_): *δ*** 10.36 (s, 1H), 8.86 (d, *J* = 2.1 Hz, 1H), 8.28 (d, *J* = 8.8 Hz, 1H), 7.34 (dd, *J* = 8.3, 2.1 Hz, 1H), 7.20 (d, *J* = 8.3 Hz, 1H), 6.68 (dd, *J* = 8.8, 2.3 Hz, 1H), 6.58 (d, *J* = 2.3 Hz, 1H), 4.10 (s, 3H), 3.91–3.85 (m, 7H), 2.96 (t, *J* = 4.6 Hz, 4H). **^13^C NMR (101 MHz, CDCl_3_): *δ*** 163.5, 162.7, 158.2, 144.3, 134.0, 133.5, 126.5 (q, *J* = 32.4 Hz), 123.6 (q, *J* = 272.0 Hz), 120.2 (q, *J* = 4.0 Hz), 119.0, 118.2 (q, *J* = 4.1 Hz), 114.2, 105.4, 98.5, 66.7, 56.0, 55.1, 51.5. **HRMS (ESI +):** calcd for C_20_H_22_F_3_N_2_O_4_^+^ [M + H]^+^ = 411.1526 *m/z*, found [M + H]^+^ = 411.1516 *m/z*, error = 2.43 ppm.

##### 4.1.7.2 Synthesis of Compound 7

In a solution of aniline **5a** (50.0 mg, 0.20 mmol) in DMF (2 mL), carboxylic acid **6e** (0.4 mmol, 60.9 mg), HBTU (0.40 mmol, 151.7 mg), and triethylamine (0.6 mmol, 60.7 mg, 84 μL) were added. The reaction mixture was stirred at 60 °C and followed general procedure C, affording compound 8 in 39% yield after 125 h.

**(7)** White solid, 30.1 mg, 39%. **^1^H NMR (400 MHz, CDCl_3_)*:*** *δ* 10.49 (s, 1H), 8.89 (d, *J* = 2.1 Hz, 1H), 8.31 (dd, *J* = 7.8, 1.9 Hz, 1H), 7.56–7.51 (m, 1H), 7.36 (dd, *J* = 8.3, 2.1 Hz, 1H), 7.22 (d, *J* = 8.3 Hz, 1H), 7.17 (td, *J* = 7.6, 1.1 Hz, 1H), 7.08 (d, *J* = 7.5 Hz, 1H), 4.12 (s, 3H), 3.89 (t, *J* = 4.6 Hz, 4H), 2.97 (t, *J* = 4.6 Hz, 4H). **^13^C NMR APT (101 MHz, CDCl_3_): *δ*** 163.4, 157.4, 144.9, 133.9, 133.6, 132.8, 127.1 (q, *J* = 32.8 Hz), 124.2 (q, *J* = 272.4 Hz), 121.9, 121.9, 121.0 (q, *J* = 4.0 Hz), 119.7, 118.6 (q, *J* = 3.9 Hz), 67.2, 56.6, 52.1. **HRMS (ESI +):** calcd for C_19_H_20_F_3_N_2_O_3_^+^ [M + H]^+^ = 381.1420 *m/z*, found [M + H]^+^ = 381.1411 *m/z*, error = 2.36 ppm.

##### 4.1.7.3 Synthesis of Compound 11

In a solution of aniline **5a** (30.0 mg, 0.122 mmol) in DMF (2 mL), carboxylic acid **6f** (0.183 mmol, 33.3 mg), HBTU (0.183 mmol, 69.4 mg), and triethylamine (0.366 mmol, 37.0 mg, 51 μL) were added. The reaction mixture was stirred at 95 °C and followed general procedure C, affording compound **11** in 34% yield after 120 h.

**(11)** White solid, 17.0 mg, 34%. **^1^H NMR (400 MHz, CDCl_3_): *δ*** 10.99 (s, 1H), 9.05 (d, *J* = 2.2 Hz, 1H), 7.83 (dd, *J* = 8.0, 1.7 Hz, 1H), 7.38 (dd, *J* = 7.9, 1.8 Hz, 1H), 7.27 (d, *J* = 2.7 Hz, 1H), 7.25–7.21 (m, 1H), 7.13 (dd, *J* = 8.1, 1.7 Hz, 1H), 4.01 (*s*, 3H), 3.97–3.93 (m, 7H), 2.97 (t, *J* = 4.6 Hz, 4H). **^13^C NMR APT (101 MHz, CDCl_3_): *δ*** 163.3, 152.8, 147.7, 144.4, 134.5, 127.5, 127.2, 126.8, 124.6, 123.1, 120.8 (d, *J* = 4.0 Hz), 120.1, 117.7–117.5 (m), 116.1, 67.0, 61.4, 56.2, 52.3. **HRMS (ESI +):** calcd for C_20_H_22_F_3_N_2_O_4_^+^ [M + H]^+^ = 411.1526 *m/z*, found [M + H]^+^ = 411.1510 *m/z*, error = 3.89 ppm.

##### 4.1.7.4 Synthesis of Compound 12

In a solution of aniline **5a** (30.0 mg, 0.122 mmol) in DMF (2 mL), carboxylic acid **6g** (0.244 mmol, 41.5 mg), HBTU (0.244 mmol, 92.5 mg), and triethylamine (0.488 mmol, 49.4 mg, 68 μL) were added. The reaction mixture was stirred at 95 °C and followed general procedure C, affording compound **12** in 52% yield after 51 h.

**(12)** Yellow solid, 25.4 mg, 52%. **^1^H NMR (400 MHz, CDCl_3_): *δ*** 10.34 (s, 1H), 8.85 (d, *J* = 2.5 Hz, 1H), 8.33 (dd, *J* = 8.8, 7.0 Hz, 1H), 7.37 (dd, *J* = 8.3, 2.8 Hz, 1H), 7.23 (d, *J* = 8.3 Hz, 1H), 6.87 (ddd, *J* = 8.7, 7.5, 2.3 Hz, 1H), 6.79 (dd, *J* = 10.4, 2.4 Hz, 1H), 4.12 (*s*, 3H), 3.90–3.87 (m, 4H), 2.98–2.94 (m, 4H). **^13^C NMR APT (101 MHz, CDCl_3_): *δ*** 164.8 (d, *J* = 465.8 Hz), 164.6, 158.7, 144.8, 134.9 (d, *J* = 10.6 Hz), 133.8, 127.2 (d, *J* = 32.6 Hz), 121.1 (t, *J* = 4.0 Hz), 119.8, 118.7 (d, *J* = 3.7 Hz), 108.9 (d, *J* = 21.3 Hz), 100.0 (d, *J* = 26.4 Hz), 67.2, 57.0, 52.1. **HRMS (ESI +):** calcd for C_19_H_19_F_4_N_2_O_3_^+^ [M + H]^+^ = 399.1326 *m/z*, found [M + H]^+^ = 399.1317 *m/z*, error = 2.25 ppm.

##### 4.1.7.5 Synthesis of Compound 14

In a solution of aniline **5a** (50.0 mg, 0.20 mmol) in DMF (2 mL), carboxylic acid **6i** (0.30 mmol, 45.9 mg), HBTU (0.30 mmol, 113.8 mg), and triethylamine (0.6 mmol, 60.7 mg, 85 μL) were added. The reaction mixture was stirred at 95 °C and followed general procedure C, affording compound **14** in 15% yield after 147 h.

**(14)** White solid, 11.3 mg, 15%. **^1^H NMR (400 MHz, CDCl_3_): *δ*** 9.29 (*s*, 1H), 8.88 (d, *J* = 2.1 Hz, 1H), 8.73 (d, *J* = 2.5 Hz, 1H), 8.16 (dd, *J* = 8.7, 2.6 Hz, 1H), 7.39 (dd, *J* = 8.4, 2.3 Hz, 1H), 7.31 (d, *J* = 8.3 Hz, 1H), 6.89 (d, *J* = 8.7 Hz, 1H), 4.04 (*s*, 3H), 3.95–3.91 (m, 4H), 2.98–2.93 (m, 4H). **^13^C NMR APT (101 MHz, CDCl_3_): *δ*** 166.7, 163.3, 146.3, 144.0, 138.0, 134.1, 123.9, 121.1 (d, *J* = 4.6 Hz), 117.0, 111.7, 67.7, 54.3, 52.6. **HRMS (ESI +):** calcd for C_18_H_19_F_3_N_3_O_3_^+^ [M + H]^+^ = 382.1373 *m/z*, found [M + H]^+^ = 382.1367 *m/z*, error = 1.57 ppm.

##### 4.1.7.6 Synthesis of Compound 16

In a solution of aniline **5a** (50.0 mg, 0.20 mmol) in DMF (2 mL), carboxylic acid **6j** (0.579 mmol, 80.5 mg), HBTU (0.609 mmol, 231.0 mg), and triethylamine (0.812 mmol, 82.2 mg, 110 μL) were added. The reaction mixture was stirred at 95 °C and followed general procedure C, affording compound **16** in 70% yield after 94 h.

**(16)** Dark brown solid, 52.4 mg, 70%. **^1^H NMR (400 MHz, CDCl_3_): *δ*** 10.87 (s, 1H), 8.89 (d, *J* = 2.0 Hz, 1H), 8.27 (*s*, 1H), 8.19 (d, *J* = 8.5 Hz, 1H), 7.35 (t, *J* = 7.7 Hz, 2H), 7.22 (d, *J* = 8.2 Hz, 1H), 4.03–3.99 (m, 4H), 3.03–2.97 (m, 4H). **^13^C NMR APT (101 MHz, CDCl_3_): *δ*** 162.2, 155.4, 144.5, 142.7, 136.8, 133.3, 124.0, 123.4, 120.8 (d, *J* = 3.7 Hz), 119.9, 116.6 (d, *J* = 4.0 Hz), 67.4, 52.1. **HRMS (ESI +):** calcd for C_17_H_17_F_3_N_3_O_3_^+^ [M + H]^+^ = 368.1216 *m/z*, found [M + H]^+^ = 368.1212 *m/z*, error = 1.09 ppm.

##### 4.1.7.7 Synthesis of Compound 17

In a solution of aniline **5a** (32.7 mg, 0.133 mmol) in DMF (2 mL), carboxylic acid **6k** (0.400 mmol, 49.0 mg), HBTU (0.400 mmol, 151.7 mg), and triethylamine (0.532 mmol, 53.8 mg, 75 μL) were added. The reaction mixture was stirred at 95 °C and followed general procedure C, affording compound **17** in 81% yield after 68 h.

**(17)** Brown solid, 37.8 mg, 81%. **^1^H NMR (400 MHz, CDCl_3_): *δ*** 11.09 (*s*, 1H), 8.94 (*s*, 1H), 8.69 (d, *J* = 4.9 Hz, 1H), 8.33 (d, *J* = 7.8 Hz, 1H), 7.96 (td, *J* = 7.7, 1.7 Hz, 1H), 7.54 (dd, *J* = 7.7, 4.7 Hz, 1H), 7.40 (d, *J* = 8.2 Hz, 1H), 7.26 (d, *J* = 15.7 Hz, 1H), 4.03 (t, *J* = 4.7 Hz, 4H), 3.02 (t, *J* = 4.6 Hz, 4H). **^13^C NMR APT (101 MHz, CDCl_3_): *δ*** 162.1, 150.1, 148.3, 144.6, 137.8, 133.2, 127.2 (d, *J* = 33.0 Hz), 126.6, 124.1 (d, *J* = 272.2 Hz), 122.5, 121.0 (q, *J* = 3.9 Hz), 120.0, 116.7 (q, *J* = 4.0 Hz), 67.4, 52.1. **HRMS (ESI +):** calcd for C_17_H_17_F_3_N_3_O_2_^+^ [M + H]^+^ = 352.1267 *m/z*, found [M + H]^+^ = 352.1254 *m/z*, error = 3.69 ppm.

##### 4.1.7.8 Synthesis of Compound 18

In a solution of aniline **5a** (34.3 mg, 0.139 mmol) in DMF (2 mL), carboxylic acid **6l** (0.556 mmol, 106.3 mg), HBTU (0.556 mmol, 210.8 mg), and triethylamine (0.695 mmol, 70.5 mg, 97 μL) were added. The reaction mixture was stirred at 95 °C and followed general procedure C, affording compound **18** in 55% yield after 42 h.

**(18)** Dark brown solid, 32.3 mg, 55%. **^1^H NMR (400 MHz, CDCl_3_):** *δ* 11.03 (s, 1H), 8.96 (s, 1H), 8.53 (d, *J* = 7.7 Hz, 1H), 8.16 (t, *J* = 7.8 Hz, 1H), 7.92 (d, *J* = 7.9 Hz, 1H), 7.42 (d, *J* = 9.5 Hz, 1H), 7.30 (d, *J* = 8.4 Hz, 1H), 4.02–3.96 (m, 4H), 3.00–2.94 (m, 4H). **^13^C NMR APT (101 MHz, CDCl_3_): *δ*** 160.9, 150.7, 147.3 (d, *J* = 35.9 Hz), 145.0, 139.8, 133.4, 127.8 (d, *J* = 32.6 Hz), 125.5, 123.3 (d, *J* = 2.6 Hz), 122.8, 121.6 (q, *J* = 4.0 Hz), 120.9, 117.0–116.6 (m), 67.3, 52.6. **HRMS (ESI +):** calcd for C_18_H_16_F_6_N_3_O_2_^+^ [M + H]^+^ = 420.1141 *m/z*, found [M + H]^+^ = 420.1126 *m/z*, error = 3.57 ppm.

##### 4.1.7.9 Synthesis of Compound 20

In a solution of aniline **5b** (29.6 mg, 0.150 mmol) in DMF (2 mL), carboxylic acid **6a** (0.300 mmol, 54.7 mg), HBTU (0.300 mmol, 113.8 mg), and triethylamine (0.45 mmol, 45.5 mg, 63 μL) were added. The reaction mixture was stirred at 75 °C and followed general procedure C, affording compound **20** in 52% yield after 26 h.

**(20)** Beige solid, 28 mg, 52%. **^1^H NMR (400 MHz, CDCl_3_): *δ*** 10.45 (s, 1H), 8.40 (dd, *J* = 11.3, 3.2 Hz, 1H), 8.25 (d, *J* = 8.8 Hz, 1H), 7.10 (dd, *J* = 8.9, 5.6 Hz, 1H), 6.76 (td, *J* = 8.3, 3.0 Hz, 1H), 6.67 (dd, *J* = 8.8, 2.4 Hz, 1H), 6.57 (d, *J* = 2.5 Hz, 1H), 4.09 (s, 3H), 3.90–3.84 (m, 7H), 2.89 (t, *J* = 4.5 Hz, 4H). **^13^C NMR APT (101 MHz, CDCl_3_): *δ*** 163.33, 162.59 (d, *J* = 271.0 Hz), 158.80 (d, *J* = 8.5 Hz), 137.70 (d, *J* = 2.9 Hz), 135.50, 135.38, 134.44, 120.77 (d, *J* = 9.6 Hz), 115.16, 109.77 (d, *J* = 22.7 Hz), 108.66 (d, *J* = 28.7 Hz), 105.89, 99.04, 67.44, 56.65, 55.62, 52.78. **HRMS (ESI +):** calcd for C_19_H_22_FN_2_O_4_^+^ [M + H]^+^ = 361.1558 *m/z*, found [M + H]^+^ = 361.1548 *m/z*, error = 2.77 ppm.

##### 4.1.7.10 Synthesis of Compound 21

In a solution of aniline **5c** (50.0 mg, 0.212 mmol) in DMF (2 mL), carboxylic acid **6a** (0.424 mmol, 77.2 mg), HBTU (0.424 mmol, 160.8 mg), and triethylamine (0.636 mmol, 160.8 mg, 221 μL) were added. The reaction mixture was stirred at 75 °C and followed general procedure C, affording compound **21** in 13% yield after 23 h.

**(21)** Yellow solid, 11.1 mg, 13%. **^1^H NMR (400 MHz, CDCl_3_): *δ*** 10.53 (*s*, 1H), 8.64 (d, *J* = 8.4 Hz, 1H), 8.26 (d, *J* = 8.7 Hz, 1H), 7.89–7.83 (m, 2H), 6.68 (dd, *J* = 8.8, 2.3 Hz, 1H), 6.57 (d, *J* = 2.3 Hz, 1H), 4.10 (*s*, 3H), 3.93–3.86 (m, 10H), 2.96 (t, *J* = 4.7 Hz, 4H). **^13^C NMR APT (101 MHz, CDCl_3_): *δ*** 166.8, 164.1, 163.4, 158.8, 141.6, 138.3, 134.5, 127.2, 125.1, 121.3, 120.5, 115.0, 106.0, 99.0, 67.3, 56.7, 55.6, 52.4, 52.0. **HRMS (ESI +):** calcd for C_21_H_25_N_2_O_6_^+^ [M + H]^+^ = 401.1707 *m/z*, found [M + H]^+^ = 401.1698 *m/z*, error = 2.24 ppm.

##### 4.1.7.11 Synthesis of Compound 22

In a solution of aniline **5d** (48.5 mg, 0.271 mmol) in DMF (2 mL), carboxylic acid **6a** (0.813 mmol, 148.1 mg), HBTU (0.813 mmol, 316.0 mg), and triethylamine (1.36 mmol, 137.1 mg, 189 μL) were added. The reaction mixture was stirred at 85 °C and followed general procedure C, affording compound **22** in 68% yield after 75 h.

**(22)** Dark red solid, 63.6 mg, 68%. **^1^H NMR (400 MHz, CDCl_3_): *δ*** 10.18 (s, 1H), 8.73 (dd, *J* = 8.0, 1.8 Hz, 1H), 8.27 (d, *J* = 8.8 Hz, 1H), 8.09 (dd, *J* = 4.8, 1.7 Hz, 1H), 7.06 (dd, *J* = 8.1, 4.8 Hz, 1H), 6.68 (dd, *J* = 8.8, 2.3 Hz, 1H), 6.57 (d, *J* = 2.3 Hz, 1H), 4.11 (s, 3H), 3.91–3.86 (m, 7H), 3.16–3.12 (m, 4H).**^13^C NMR APT (101 MHz, CDCl_3_): *δ*** 164.1, 163.4, 158.8, 153.4, 142.4, 134.4, 128.9, 128.3, 119.9, 114.5, 105.9, 98.9, 67.2, 56.5, 55.6, 50.3. **HRMS (ESI +):** calcd for C_18_H_22_N_3_O_4_^+^ [M + H]^+^ = 344.1604 *m/z*, found [M + H]^+^ = 344.1601 *m/z*, error = 0.87 ppm.

##### 4.1.7.12 Synthesis of Compound 23

In a solution of aniline **5e** (33.1 mg, 0.169 mmol) in DMF (2 mL), carboxylic acid **6a** (0.507 mmol, 92.4 mg), HBTU (0.507 mmol, 192.0 mg), and triethylamine (0.845 mmol, 85.5 mg, 118 μL) were added. The reaction mixture was stirred at 113 °C and followed general procedure C, affording compound **23** after 46 h (material still impure).

**(23)** Yellow solid, 16.0 mg, 26%. **^1^H NMR (400 MHz, CDCl_3_): *δ*** 10.19 (*s*, 1H), 8.45–8.40 (m, 1H), 8.28 (d, *J* = 8.8 Hz, 1H), 6.89–6.83 (m, 2H), 6.67 (dd, *J* = 8.8, 2.4 Hz, 1H), 6.57 (d, *J* = 2.4 Hz, 1H), 4.09 (s, 3H), 3.89 (*s*, 3H), 3.88–3.84 (m, 4H), 2.93–2.90 (m, 4H). **^13^C NMR APT (101 MHz, CDCl_3_): *δ*** 164.0, 163.1, 159.4 (d, *J* = 243.9 Hz), 158.8, 143.9 (d, *J* = 7.3 Hz), 134.6, 129.8 (d, *J* = 2.9 Hz), 123.1 (d, *J* = 8.8 Hz), 115.1, 111.2 (d, *J* = 21.6 Hz), 107.2 (d, *J* = 23.5 Hz), 105.9, 99.1, 67.4, 56.6, 55.8, 52.3.**HRMS (ESI +):** calcd for C_19_H_22_FN_2_O_4_^+^ [M + H]^+^ = 361.1558 *m/z*, found [M + H]^+^ = 361.1548 *m/z*, error = 2.77 ppm.

##### 4.1.7.13 Synthesis of Compound 25

In a solution of aniline **5b** (32,4 mg, 0,165 mmol) in DMF (2 mL), carboxylic acid **6i** (0,495 mmol, 68,9 mg), HBTU (0,495 mmol, 187,7 mg), and triethylamine (0,660 mmol, 66,8 mg, 92 µL) were added. The reaction mixture was stirred at 80 °C and followed general procedure C, affording compound **25** in 66% yield after 71 h.

**(25)** Brown solid, 34,4 mg, 66%. **^1^H NMR (400 MHz, CDCl_3_): *δ*** 10.97 (s, 1H), 8.30–8.20 (m, 2H), 8.04 (d, *J* = 8.5 Hz, 1H), 7.38 (dd, *J* = 8.6, 2.7 Hz, 1H), 7.32 (dd, *J* = 8.8, 5.8 Hz, 1H), 6.91 (td, *J* = 8.5, 3.0 Hz, 1H), 3.84 (t, *J* = 4.6 Hz, 4H), 2.84 (t, *J* = 4.6 Hz, 4H). **^13^C NMR APT (101 MHz, CDCl_3_): *δ*** 161.8, 159.1 (d, *J* = 238.8 Hz), 157.1, 140.5, 137.8 (d, *J* = 2.9 Hz), 137.2, 134.3 (d, *J* = 12.5 Hz), 123.8, 123.2, 122.3 (d, *J* = 9.9 Hz), 109.5 (d, *J* = 22.4 Hz), 105.3 (d, *J* = 28.6 Hz), 66.9, 52.2.**HRMS (ESI +):** calcd for C_16_H_17_FN_3_O_3_^+^ [M + H]^+^ = 318.1248 *m/z*, found [M + H]^+^ = 318.1242 *m/z*, error = 1.89 ppm.

##### 4.1.7.14 Synthesis of Compound 26

In a solution of aniline **5j** (60.4 mg, 0.375 mmol) in DMF (2 mL), carboxylic acid **6a** (1.46 mmol, 265.3 mg), HBTU (1.46 mmol, 567.0 mg), and triethylamine (1.88 mmol, 189.8 mg, 262 μL) were added. The reaction mixture was stirred at 80 °C and followed general procedure C, affording compound **26** in 61% yield after 89 h.

**(26)** Red solid, 74.3 mg, 61%. **^1^H NMR (400 MHz, CDCl_3_): *δ*** 9.83 (s, 1H), 8.26 (d, *J* = 8.8 Hz, 1H), 7.94 (s, 1H), 7.87 (d, *J* = 8.0 Hz, 1H), 7.46 (t, *J* = 8.2 Hz, 1H), 7.35 (dd, *J* = 7.8, 0.7 Hz, 1H), 6.67 (dd, *J* = 8.8, 2.4 Hz, 1H), 6.55 (d, *J* = 2.4 Hz, 1H), 4.06 (s, 3H), 3.88 (s, 3H). **^13^C NMR APT (101 MHz, CDCl_3_): *δ*** 164.2, 163.5, 158.8, 139.3, 134.5, 129.6, 123.6, 120.8, 120.5 (d, *J* = 3.7 Hz), 117.1 (d, *J* = 4.0 Hz), 114.4, 106.0, 99.0, 56.5, 55.8. **HRMS (ESI +):** calcd for C_16_H_15_F_3_NO_3_^+^ [M + H]^+^ = 326.0998 *m/z*, found [M + H]^+^ = 326.0989 *m/z*, error = 2.76 ppm.

##### 4.1.7.15 Synthesis of Compound 29

In a solution of aniline **5h** (27.0 mg, 0.078 mmol) in DMF (2 mL), carboxylic acid **6a** (0.310 mmol, 56.8 mg), HBTU (0.310 mmol, 117.6 mg), and triethylamine (0.39 mmol, 39.0 mg, 54 μL) were added. The reaction mixture was stirred at 100 °C and followed general procedure C, affording compound **29** in 30% yield after 114 h.

**(29)** Reddish oil, 12 mg, 30%.**^1^H NMR (400 MHz, CDCl_3_): *δ*** 10.36 (s, 1H), 8.86 (d, *J* = 2.4 Hz, 1H), 8.28 (d, *J* = 8.8 Hz, 1H), 7.35–7.31 (m, 1H), 7.17 (d, *J* = 7.6 Hz, 1H), 6.68 (dd, *J* = 8.8, 2.3 Hz, 1H), 6.56 (d, *J* = 2.3 Hz, 1H), 4.05 (s, 3H), 3.89 (s, 3H), 3.61 (t, *J* = 5.1 Hz, 4H), 2.90 (t, *J* = 5.1 Hz, 4H), 1.49 (s, 9H). **^13^C NMR APT (101 MHz, CDCl_3_):** *δ* 164.2, 163.4, 158.9, 154.8, 145.0, 134.6, 134.1, 127.1 (q, *J* = 32.6 Hz), 125.4, 124.3 (q, *J* = 273.0 Hz), 120.8 (q, *J* = 3.9 Hz), 119.8, 118.8 (q, *J* = 3.9 Hz), 114.9, 106.2, 99.2, 56.7, 55.8, 51.8, 29.8, 28.6. **HRMS (ESI +):** calcd for C_25_H_31_F_3_N_3_O_5_^+^ [M + H]^+^ = 510.2210 *m/z*, found [M + H]^+^ = 510.2191 *m/z*, error = 3.72 ppm.

#### 4.1.8 General Procedure D for Amide Formation via Acyl Chloride

The carboxylic acid (1 eq. of the desired acyl chloride) was kept under an argon atmosphere and then dissolved in anhydrous DCM (2 mL), followed by the addition of oxalyl chloride (5 eq. of the carboxylic acid) and two drops of anhydrous DMF. The reaction was stirred at room temperature under an argon atmosphere for 2 h, and then the solvent and residual oxalyl chloride were evaporated and dried under reduced pressure. In the next step, a solution of acyl chloride in anhydrous DCM (2 mL) was added to a solution of aniline and anhydrous triethylamine in anhydrous DCM (2 mL). The reaction mixture was stirred at room temperature under an argon atmosphere and monitored by TLC. After completion, 25 mL of distilled water was added, and the mixture was transferred to a separatory funnel. Extraction was performed with dichloromethane (15 mL), and the organic layer was washed with a basic NaOH solution (2 M, 25 mL), dried over anhydrous Na_2_SO_4_, filtered, concentrated, and dried. The product was then purified by flash column chromatography using silica gel and a mixture of 70% hexane and 30% ethyl acetate as the eluent.

##### 4.1.8.1 Synthesis of Compound 1

The synthesis of Hit **1** was carried out following general procedure D, using aniline **5a** (30.0 mg, 0.122 mmol), acyl chloride **7a** (0.487 mmol, 97.8 mg), and Et_3_N (0.24 mmol, 24.7 mg, 34 µL) in anhydrous DCM (2 mL). After 114 h at room temperature under an argon atmosphere, compound **1** was obtained with 53% yield.

**(1)** White solid, 26.5 mg, 53%.**^1^H NMR (400 MHz, CDCl_3_): *δ*** 10.36 (s, 1H), 8.86 (d, *J* = 2.1 Hz, 1H), 8.28 (d, *J* = 8.8 Hz, 1H), 7.34 (dd, *J* = 8.3, 2.1 Hz, 1H), 7.20 (d, *J* = 8.3 Hz, 1H), 6.68 (dd, *J* = 8.8, 2.3 Hz, 1H), 6.58 (d, *J* = 2.3 Hz, 1H), 4.10 (s, 3H), 3.91–3.85 (m, 7H), 2.96 (t, *J* = 4.6 Hz, 4H). **^13^C NMR (101 MHz, CDCl_3_): *δ*** 163.5, 162.7, 158.2, 144.3, 134.0, 133.5, 126.5 (q, *J* = 32.4 Hz), 123.6 (q, *J* = 272.0 Hz), 120.2 (q, *J* = 4.0 Hz), 119.0, 118.2 (q, *J* = 4.1 Hz), 114.2, 105.4, 98.5, 66.7, 56.0, 55.1, 51.5. **HRMS (ESI +):** calcd for C_20_H_22_F_3_N_2_O_4_^+^ [M + H]^+^ = 411.1526 *m/z*, found [M + H]^+^ = 411.1516 *m/z*, error = 2.43 ppm.

##### 4.1.8.2 Synthesis of Compound 9

The synthesis of compound **9** was carried out following general procedure D, using aniline **5a** (20.0 mg, 0.081 mmol), commercially available acyl chloride **7b** (0.162 mmol, 23.0 mg), and Et_3_N (0.200 mmol, 20.2 mg, 27 µL) in anhydrous DCM (2 mL). After 150 h at room temperature under an argon atmosphere, compound **9** was obtained with 92% yield.

**(9)** White solid, 26.1 mg, 92%. **^1^H NMR (400 MHz, CDCl_3_): *δ*** 9.38 (s, 1H), 8.92 (d, *J* = 2.1 Hz, 1H), 8.13–8.08 (m, 1H), 7.95–7.90 (m, 2H), 7.63–7.52 (m, 3H), 7.47 (t, *J* = 7.7 Hz, 1H), 7.41–7.36 (m, 1H), 7.30 (d, *J* = 8.3 Hz, 1H), 3.94–3.89 (m, 4H), 2.98–2.94 (m, 4H). **^13^C NMR APT (101 MHz, CDCl_3_): *δ*** 164.9, 143.8, 134.5, 134.0, 132.2, 129.1, 128.2, 127.9, 126.9, 121.0–120.8 (m), 116.8 (q, *J* = 3.9 Hz), 67.6, 52.4. **HRMS (ESI +):** calcd for C_18_H_18_F_3_N_2_O_2_^+^ [M + H]^+^ = 351.1314 *m/z*, found [M + H]^+^ = 351.1304 *m/z*, error = 2.85 ppm.

##### 4.1.8.3 Synthesis of Compound 10

The synthesis of compound **10** was carried out following general procedure D, using aniline **5a** (50.0 mg, 0.200 mmol), acyl chloride **7c** (0.600 mmol, 120.4 mg), and Et_3_N (0.200 mmol, 20.2 mg, 27 µL) in anhydrous DCM (2 mL). After 91 h at room temperature under an argon atmosphere, compound **10** was obtained with 64% yield.

**(10)** White solid, 52 mg, 64%. **^1^H NMR (400 MHz, CDCl_3_):** *δ* 9.33 (s, 1H), 8.90 (d, *J* = 2.2 Hz, 1H), 7.59 (d, *J* = 2.1 Hz, 1H), 7.43 (dd, *J* = 8.4, 2.1 Hz, 1H), 7.37 (dd, *J* = 8.4, 2.1 Hz, 1H), 7.29 (d, *J* = 8.3 Hz, 1H), 6.98 (d, *J* = 8.3 Hz, 1H), 3.98 (s, 3H), 3.98 (s, 3H), 3.92 (t, *J* = 4.6 Hz, 4H), 2.96 (t, *J* = 4.5 Hz, 4H). **^13^C NMR (101 MHz, CDCl_3_):** *δ* 164.4, 152.5, 149.5, 143.7, 134.1, 127.1, 129.3–122.2 (m), 120.8, 120.7–120.4 (m), 119.0, 116.7 (d, *J* = 4.1 Hz), 110.8, 110.6, 67.7, 56.1 (d, *J* = 1.7 Hz), 52.4. **HRMS (ESI +):** calcd for C_20_H_22_F_3_N_2_O_4_^+^ [M + H]^+^ = 411.1526 *m/z*, found [M + H]^+^ = 411.1519 *m/z*, error = 1.70 ppm.

##### 4.1.8.4 Synthesis of Compound 19

The synthesis of compound **19** was carried out following general procedure D, using aniline **5a** (30.0 mg, 0.122 mmol), acyl chloride **7d** (0.487 mmol, 77.7 mg), and Et_3_N (0.365 mmol, 37.0 mg, 50 µL) in anhydrous DCM (2 mL). After 19 h at room temperature under an argon atmosphere, compound **19** was obtained with 89% yield.

**(19)** White solid, 39.9 mg, 89%. **^1^H NMR (400 MHz, CDCl_3_): *δ*** 10.87 (s, 1H), 8.88 (d, *J* = 2.3 Hz, 1H), 8.51 (d, *J* = 2.9 Hz, 1H), 8.34 (dd, *J* = 8.6, 4.6 Hz, 1H), 7.62 (td, J = 8.3, 2.8 Hz, 1H), 7.37 (dd, *J* = 8.2, 2.2 Hz, 1H), 7.23 (d, *J* = 8.2 Hz, 1H), 4.01–3.96 (m, 4H), 3.01–2.95 (m, 4H). **^13^C NMR APT (101 MHz, CDCl_3_): *δ*** 161.6 (d, *J* = 261.9 Hz), 161.2, 146.6 (d, *J* = 4.0 Hz), 144.8, 137.1 (d, *J* = 25.3 Hz), 133.2, 127.4 (q, *J* = 32.6 Hz), 124.6 (d, *J* = 8.8 Hz), 124.4 (d, *J* = 21.6 Hz), 124.2 (q, *J* = 272.2 Hz), 121.2 (q, *J* = 3.9 Hz), 120.3, 116.8 (q, *J* = 4.0 Hz), 67.5, 52.3. **HRMS (ESI +):** calcd for C_17_H_16_F_4_N_3_O_2_^+^ [M + H]^+^ = 370.1173 *m/z*, found [M + H]^+^ = 370.1163 *m/z*, error = 2.70 ppm.

##### 4.1.8.5 Synthesis of Compound 24

The synthesis of compound **24** was carried out following general procedure D, using aniline **5f** (33.0 mg, 0.134 mmol), acyl chloride **7a** (0.536 mmol, 107.5 mg), and Et_3_N (0.268 mmol, 27.1 mg, 37 µL) in anhydrous DCM (2 mL). After 116 h at room temperature under an argon atmosphere, compound **24** was obtained with 24% yield.

**(24)** White solid, 13 mg, 24%. **^1^H NMR (400 MHz, CDCl_3_): *δ*** 9.74 (s, 1H), 8.25 (d, *J* = 8.8 Hz, 1H), 7.95 (dd, *J* = 8.2, 2.7 Hz, 1H), 7.79 (d, *J* = 2.6 Hz, 1H), 7.37 (d, *J* = 8.6 Hz, 1H), 6.66 (dd, *J* = 8.8, 2.4 Hz, 1H), 6.55 (d, *J* = 2.3 Hz, 1H), 4.05 (s, 3H), 3.88 (s, 3H), 3.84–3.81 (m, 4H), 2.90 (t, *J* = 4.6 Hz, 4H). **^13^C NMR APT (101 MHz, CDCl_3_): *δ*** 164.2, 163.4, 158.8, 147.9, 135.9, 134.5, 128.7–127.7 (m), 124.9, 119.7 (m), 119.4, 106.0, 99.0, 67.6, 56.5, 55.8, 54.0. **HRMS (ESI +):** calcd for C_20_H_22_F_3_N_2_O_4_^+^ [M + H]^+^ = 411.1526 *m/z*, found [M + H]^+^ = 411.1512 *m/z*, error = 3.41 ppm.

##### 4.1.8.6 Synthesis of Compound 27

The synthesis of compound **27** was carried out following general procedure D, using aniline **5g** (40.0 mg, 0.153 mmol), acyl chloride **7a** (0.612 mmol, 122.8 mg), and Et_3_N (0.46 mmol, 46.5 mg, 64 µL) in anhydrous DCM (2 mL). After 28 h at room temperature under an argon atmosphere, compound **27** was obtained with 37% yield.

**(27)** Light yellow solid, 24.4 mg, 37%. **^1^H NMR (400 MHz, CDCl_3_): *δ*** 10.33 (s, 1H), 8.88 (d, *J* = 2.3 Hz, 1H), 8.29 (d, *J* = 8.8 Hz, 1H), 7.33 (dd, *J* = 8.3, 2.4 Hz, 1H), 7.19 (d, *J* = 8.3 Hz, 1H), 6.69 (dd, *J* = 8.8, 2.4 Hz, 1H), 6.58 (d, *J* = 2.3 Hz, 1H), 4.10 (s, 3H), 3.89 (s, 3H), 3.22–3.14 (m, 4H), 2.87–2.80 (m, 4H). **^13^C NMR APT (101 MHz, CDCl_3_): *δ*** 164.2, 163.5, 158.8, 146.1, 134.7, 134.4, 127.4 (d, *J* = 32.3 Hz), 120.7 (q, *J* = 4.0 Hz), 120.6, 118.7 (q, *J* = 4.0 Hz), 114.9, 106.1, 99.2, 56.6, 55.8, 54.4, 28.4. **HRMS (ESI +):** calcd for C_20_H_22_F_3_N_2_O_3_S^+^ [M + H]^+^ = 427.1297 *m/z*, found [M + H]^+^ = 427.1292 *m/z*, error = 1.17 ppm.

##### 4.1.8.7 Synthesis of Compound 28

For the synthesis of compound **28**, the general procedure D was followed using aniline **5h** (52.2 mg, 0.201 mmol), acyl chloride **7a** (0.804 mmol, 161.3 mg), and Et_3_N (0.603 mmol, 61.0 mg, 84 µL) in anhydrous DCM (2 mL). After stirring for 45 h at room temperature under an argon atmosphere, compound **28** was obtained in 20% yield.

**(28)** Light brown solid, 17.2 mg, 20%. **^1^H NMR (400 MHz, CDCl_3_): *δ*** 10.34 (s, 1H), 8.85 (d, *J* = 2.6 Hz, 1H), 8.28 (d, *J* = 8.7 Hz, 1H), 7.35–7.30 (m, 1H), 7.21 (d, *J* = 8.6 Hz, 1H), 6.68 (dd, *J* = 8.8, 2.4 Hz, 1H), 6.57 (d, *J* = 2.3 Hz, 1H), 4.08 (s, 3H), 3.89 (s, 3H), 2.99 (t, *J* = 4.8 Hz, 4H), 2.62 (s, 4H), 2.38 (s, 3H). **^13^C NMR APT (101 MHz, CDCl_3_): *δ*** 164.0, 163.3, 158.8, 145.2, 134.5, 134.0, 127.4–126.2 (m), 121.5 (d, *J* = 271.8 Hz), 120.6 (q, *J* = 4.0 Hz), 119.6, 118.6–118.3 (m), 114.9, 105.8, 98.9, 56.6, 55.6, 55.4, 51.6, 46.2. **HRMS (ESI +):** calcd for C_21_H_25_F_3_N_3_O_3_^+^ [M + H]^+^ = 424.1842 *m/z*, found [M + H]^+^ = 424.1836 *m/z*, error = 1.41 ppm.

##### 4.1.8.8 Procedure for the Synthesis of Compound 8

In a solution of aniline **5a** (20.0 mg, 0.0812 mmol) in DCM (2 mL), carboxylic acid **6f** (16.1 mg, 0.106 mmol), EDC (20.3 mg, 0.106 mmol), and DMAP (3.0 mg, 0.025 mmol) were added. The reaction was stirred at room temperature and monitored by TLC. After 16 h, little progress was observed, so the mixture was heated under reflux at 40 °C. After 24 h, product formation slightly increased, but the unreacted amine was still predominant. Additional DMAP (4.0 mg) was then added. After 18 more hours with no significant progress, 4 mL of acetonitrile was added, and the temperature was raised to 70 °C. The reaction was quenched by cooling and adding cold distilled water (20 mL) in an ice bath, with no precipitate formation. The solution was extracted with DCM (5 mL), and the organic phase was washed with a NaOH solution (4 M, 25 mL). The solid obtained was purified by preparative thin‐layer chromatography (PTLC), affording compound **8** in 43% yield.

**(8)** White solid, 13.4 mg, 43%. **^1^H NMR (400 MHz, CDCl_3_): *δ*** 9.28 (s, 1H), 8.90 (d, *J* = 2.1 Hz, 1H), 7.92–7.87 (m, 2H), 7.36 (d, *J* = 2.1 Hz, 1H), 7.29 (s, 1H), 7.03 (d, *J* = 8.8 Hz, 2H), 3.95–3.89 (m, 7H), 2.98–2.93 (m, 4H). **^13^C NMR APT (101 MHz, CDCl_3_):** *δ* 164.4, 162.8, 143.7, 134.2, 128.8, 126.7, 120.7, 120.6 (d, *J* = 4.0 Hz), 116.8 (d, *J* = 4.1 Hz), 114.3, 67.6, 55.5, 52.4. **HRMS (ESI +):** calcd for C_19_H_20_F_3_N_2_O_3_^+^ [M + H]^+^ = 381.1420 *m/z*, found [M + H]^+^ = 381.1408 *m/z*, error = 3.15 ppm.

##### 4.1.8.9 Procedure for the Synthesis of Compound 13

In a flask containing hit **1** (20.0 mg, 0.0487 mmol), 1.0 mL of a 1 M BBr_3_ solution in DCM (approximately 10 eq. per methoxy group, totaling 20 eq.) was added under an argon atmosphere in an ice bath. The reaction was stirred at room temperature and monitored by TLC (6:4 hexane/ethyl acetate, iodine visualization). After completion, the reaction was quenched with distilled water (20 mL) and extracted with DCM (2 × 25 mL). The organic phase was dried over anhydrous Na_2_SO_4_, filtered, concentrated, and dried. The residue was purified by column chromatography (6:4 hexane/ethyl acetate), followed by recrystallization in hexane, yielding compound **13** in 40% yield.

**(13)** 7.3 mg, 40%. **^1^H NMR (400 MHz, CDCl_3_): *δ*** 12.39 (s, 1H), 9.35 (s, 1H), 8.74 (d, *J* = 2.1 Hz, 1H), 7.46–7.37 (m, 2H), 7.30 (d, *J* = 8.3 Hz, 1H), 6.53 (dq, *J* = 5.4, 2.6 Hz, 2H), 3.93 (t, *J* = 4.6 Hz, 4H), 3.86 (s, 3H), 2.96 (t, *J* = 4.6 Hz, 4H) [13]. **C NMR (101 MHz, CDCl**_**3**_ **) *δ*** 168.2, 164.8, 144.3, 133.4, 126.4, 121.3 (q, *J* = 3.8 Hz), 121.1, 117.3 (q, *J* = 3.9 Hz), 108.0, 107.7, 102.2, 67.8, 55.7, 52.5. **HRMS (ESI +):** calcd for C_19_H_20_F_3_N_2_O_4_^+^ [M + H]^+^ = 397.1369 *m/z*, found [M + H]^+^ = 397.1361 *m/z*, error = 2.01 ppm.

##### 4.1.8.10 Procedure for the Synthesis of Compound 15

To a solution of dimethyl sulfate (16.7 mg, 0.132 mmol) in DMF (1 mL) under an argon atmosphere was added a solution of phenol (20.5 mg, 0.056 mmol) in DMF (1 mL) followed by sodium tert‐butoxide (19.4 mg, 0.202 mmol). The reaction was monitored by TLC (7:3 hexane/ethyl acetate) and, after 21 h, was quenched by adding cold distilled water (20 mL) in an ice bath, forming a small amount of precipitate. The solid was filtered, dried, and recrystallized from DCM/hexane (1:3). The aqueous layer from the first filtration was extracted with ethyl acetate (30 mL), and the organic phase was washed with 20 mL of NaOH solution (2 M) and 20 mL of distilled water. The organic phase was dried over anhydrous Na_2_SO_4_, filtered, concentrated, and dried under vacuum, affording compound **15** in quantitative yield.

**(15)** dark red solid, 21.3 mg, 100%. **^1^H NMR (400 MHz, CDCl_3_): *δ*** 10.88 (s, 1H), 8.91 (d, *J* = 2.4 Hz, 1H), 8.32 (d, *J* = 2.9 Hz, 1H), 8.26 (d, *J* = 8.6 Hz, 1H), 7.36 (dt, *J* = 8.3, 2.4 Hz, 2H), 7.21 (d, *J* = 7.7 Hz, 1H), 4.00 (t, *J* = 4.6 Hz, 4H), 3.96 (s, 3H), 2.99 (t, *J* = 4.6 Hz, 4H). **^13^C NMR (101 MHz, CDCl_3_):** *δ* 162.1, 158.3, 144.5, 142.7, 136.6, 133.4, 127.3, 127.0, 123.8, 120.8–120.6 (m), 120.6, 119.9, 116.7–116.5 (m), 67.4, 55.9, 52.1. **HRMS (ESI +):** calcd for C_18_H_18_F_3_N_3_NaO_3_^+^ [M + Na]^+^ = 404.1192 *m/z*, found [M + Na]^+^ = 404.1182 *m/z*, error = 2.72 ppm.

##### 4.1.8.11 Procedure for the Synthesis of Compound 30

To a solution of Boc‐piperazine (7.0 mg, 0.0137 mmol) in DCM (1 mL) was added TFA (70 µL). The reaction was stirred at room temperature for 20 h and monitored by TLC (7:3 hexane/ethyl acetate). After completion, the solvent was removed, and the resulting salt was neutralized by extraction with NaOH solution (2 M, 4 mL) and ethyl acetate (6 mL). The organic phase was dried over anhydrous Na_2_SO_4_, filtered, and evaporated, yielding compound **30** in 77% yield.

**(30)** white solid, 4.3 mg, 77%. **^1^H NMR (400 MHz, CDCl_3_): *δ*** 10.39 (s, 1H), 8.86 (d, *J* = 2.4 Hz, 1H), 8.28 (d, *J* = 8.7 Hz, 1H), 7.33 (ddd, *J* = 8.3, 2.2, 0.8 Hz, 1H), 7.19 (dd, *J* = 8.3, 0.8 Hz, 1H), 6.68 (dd, *J* = 8.9, 2.3 Hz, 1H), 6.57 (d, *J* = 2.3 Hz, 1H), 4.08 (s, 3H), 3.89 (s, 3H), 3.09–3.05 (m, 4H), 2.91 (t, *J* = 4.8 Hz, 4H). **^13^C NMR (101 MHz, CDCl_3_): *δ*** 164.2, 163.5, 158.9, 145.7, 134.6, 134.2, 126.9 (d, *J* = 32.3 Hz), 128.5–120.2 (m), 120.9–120.6 (m), 119.8, 118.7–118.5 (m), 115.0, 106.0, 99.1, 56.7, 55.8, 53.3, 46.5. **HRMS (ESI +):** calcd for C_20_H_23_F_3_N_3_O_3_^+^ [M + H]^+^ = 410.1686 *m/z*, found [M + H]^+^ = 410.1679 *m/z*, error = 1.71 ppm.

### 4.2 In Vitro Biological Assays

#### 4.2.1 Anti‐*T. cruzi* Activity Assays

The in vitro anti‐*T. cruzi* activity of the synthesized compounds was evaluated using different parasite strains (Tulahuen, Y, and CL‐Brener) and host cell models. For assays with the Tulahuen strain, the in vitro anti‐*T. cruzi* activity was evaluated on L929 cells (mouse fibroblasts) infected with the Tulahuen strain of the parasite expressing the Escherichia coli β‐galactosidase as a reporter gene were used. For the bioassay, 4000 L929 cells (4 × 103 cells/well) were added to each well of seeded in a 96‐well microtiter plate. After an overnight incubation, 40,000 trypomastigotes were added to the cells (10:1 ratio) and incubated for 2 h. Then, the medium containing extracellular parasites was replaced with 200 µL of fresh medium, and the plate was incubated for an additional 48 h to establish the infection. Infected cells were subsequently treated with serial concentrations of the compounds. To determine the IC_50_ of the compounds, cells were exposed to each compound at successively decreasing concentrations (100, 50, 25, 12.5, 6.25, and 3.12 µg/mL), and the plate was incubated for 96 h. After this period, 50 µL of 500 µM chlorophenol red beta‐D‐galactopyranoside (CPRG) in 0.5 % Nonidet P40 was added to each well, followed by incubation, the plate was incubated for further 16–20 h, and the absorbance at 570 nm was measured using a microplate reader (SpectraMax M2, Molecular Devices, Sunnyvale, CA). Controls with uninfected cells, untreated infected cells, and infected cells treated with benznidazole at 3.8 µM (positive control) or DMSO 1% were used. The results were expressed as the percentage of *T. cruzi* growth inhibition relative to untreated infected controls in the cells treated with the compound tested compared to that in the infected cells and untreated cells. The IC_50_ values were determined by linear interpolation. Quadruplicates were run on the same plate, and the experiments were repeated at least once.

For assays with the Y and CL‐Brener strains, L6 cells in RPMI supplemented with 10% FCS and 100U PenStrep (Sigma) were plated seeded in 96‐well plates (1 × 104 cells/well) and maintained incubated for 24 h. *T. cruzi* Y or CL‐Brener trypomastigotes were then co‐incubated with the host cells (30:1 ratio) and incubated for 24 h at 37 °C and 5% CO_2_. The wells were then washed three times with fresh RPMI medium to remove noninternalized parasites, and the infected cells were subsequently treated with serial dilutions of the assessed compounds (200 to 0.006 µM) and incubated for 72 h. Cells infected with the Y strain were fixed and stained using a panoptic staining protocol, and infection levels were determined by counting the total number of intracellular amastigotes relative to the total number of host cells per well. For the CL‐Brener strain, parasite infection was assessed by luminescence after the addition of D‐luciferin potassium salt (IVISbrite D‐Luciferin, XenoLight, Revvity) at 3 mg/mL, with readings performed in a SpectraMax M5 microplate reader. The cells were then fixed and stained using either a panoptic staining protocol (for the Y strain) or luminescence labeling (for the CL Brener strain). The percentage of infected cells was determined by counting the total number of intracellular amastigotes relative to the total number of host cells per well (for the Y strain) or by adding IVISbrite D‐Luciferin Potassium Salt (XenoLightTM D‐Luciferin K + salt, Revvity) (3 mg/mL) and reading the luminescence on a SpectraMax M5 microplate reader. The inhibition of parasite replication was assessed by comparing the infection rates in the wells treated with the compounds to those in the untreated controls. IC_50_ was determined using four‐parameter nonlinear regression analysis on GraphPad Prism v8.4 from three independent experiments.

#### 4.2.2 Anti‐*L. infantum* Activity Assays

Cells derived from the human monocytic strain THP‐1 were cultured in complete Rowell Park Memorial Institute (RPMI)‐1640 medium (supplemented with 10% fetal bovine serum, 100 U/mL penicillin, and 100 µg/mL streptomycin). Monocytes were differentiated into macrophages by the addition of 50 ng/mL phorbol myristate acetate (PMA). After 72 h, the macrophages (5 × 10^4^), in black 96‐well microtiter plates (Corning Incorporated, Corning, NY), were infected with promastigotes forms of *Leishmania* (*Leishmania*) *infantum* (MHOM/BR/1974/PP75) transfected with gene *tdTomato*, on the second day of the stationary phase (20 parasites per macrophage) for 4 h. The parasites that failed to infect the macrophages were washed away (three times with 1X PBS), and the infected macrophages were incubated for 72 h in RPMI‐1640 medium containing different compounds concentrations (80, 40, 20, 10, and 5 µg/mL). The compound concentration that inhibits 50% of parasite growth (IC_50_) was determined by the decrease in parasite fluorescence in the absence and presence of the compound, after 72 h of exposure, using a microplate reader (Varioskan LUX plate reader, Thermo Scientific) with excitation and emission values of 554 and 581 nm, respectively. The IC_50_ values were determined by nonlinear regression using a variable slope model (log(concentration) vs. growth inhibition) with the GraphPadPrism v8.2.0 (CA, USA) software. Controls with uninfected cells, untreated infected cells, and infected cells treated with amphotericin B at 0.25 μM (positive control) or DMSO 1% were used. Quadruplicates were run on the same plate, and the experiments were repeated at least once.

### 4.3 In Vitro Cytotoxic Activity

The cytotoxicity (CC_50_) of the synthesized compounds was evaluated in vitro using L929, THP‐1–derived macrophages, and L6 cell lines by a resazurin‐based viability assay. L929 mouse fibroblasts (4 × 10^3^ cells/well), THP‐1 cells differentiated into macrophages (4 × 10^3^ cells/well), or L6 rat skeletal muscle cells (1 × 10^4^ cells/well) were seeded in 96‐well plates and incubated under standard culture conditions. Cells were treated with serial dilutions of the compounds and incubated for 72 h (THP‐1 and L6 cells) or 96 h (L929 cells). Cell viability was assessed by adding resazurin to a final concentration of 45 µM per well, followed by incubation for 4 h at 37 °C under 5% CO_2_. Fluorescence was measured upon excitation at 570 nm and emission at 590 nm. Untreated cells and cells treated with 1% DMSO were included as controls. The CC_50_ was determined by nonlinear regression analysis using GraphPad Prism v8.2.0. Experiments were performed in triplicate or quadruplicate and repeated at least once. The selectivity index (SI) was calculated as the ratio between the CC_50_ in host cells and the IC_50_ (against *T. cruzi* or *L. infantum*), with both values obtained under comparable assay conditions.

### 4.4 ADME Assays

ADME analyzes were performed using LC–MS/MS (liquid chromatography–tandem mass spectrometry). The chromatography system used was Prominence UFLC (Shimadzu Corporation, Kyoto, Japan) and interface with an LCMS‐8045 triple quadrupole mass spectrometer (Shimadzu Corporation, Kyoto, Japan) with an electrospray ionization source (ESI).

### 4.5 Experimental Determination of Distribution Coefficient (eLogD7.4)

For distribution coefficient (eLogD) determination, a methodology based on the retention time of molecules in the stationary phase was used. The chromatogram was achieved on a Supelco Ascentis express RP amide HPLC column (5 cm × 2.1 mm, 2.7 μM) and mobile phases of 5% methanol in 10 mM ammonium acetate pH 7.4 (A) and 100% methanol (B). The mobile phase was eluted in binary gradient mode, as follows: 0 min, 95% A; 0.3 min 100% A; 5.2 min 0% A; 5.6 min 0% A; 5.8 min 100% A; 7.0 min 100% A. The run time was 7 min, and the sample injection volume was 5 μL. Test compounds were prepared at 1.0 mg/mL by adding the stock solution at (1:1) mobile phases A:B (internal standard at 200 nM), and the DMSO concentration was lower than 2%. The lipophilicity of compounds was assessed by injecting individually the test compounds and a series of eight commercial drugs, covering a LogD range of −1.86 to 6.1 (acyclovir −1.86, atenolol 0.16, antipyrine 0.38, fluconazole 0.50, metoprolol 1.88, ketoconazole 3.83, tolnaftate 5.40, and amiodarone 6.10) [34, 35, 36]. The retention time (in minutes) of each of the eight standards was plotted against their LogD values. The resulting equation for the calibration curve (*y* = *m* *x* + *b*) was used to calculate the eLogD values for the compounds.

### 4.6 Mouse Liver Microsomal Stability Assay

The metabolic stability was evaluated in pooled liver CD1 mouse microsomes (20 mg/mL, GIBCO). Compounds were prepared at a concentration of 0.5 μM and incubated with 0.25 mg/mL liver microsomes at phosphate‐buffered saline pH 7.4, and the DMSO concentration was lower than 1%. The reaction started by adding the cofactor NADPH at 0.5 μM. Samples were taken at timepoint 0 (immediately following the addition of the cofactor), 5, 10, 20, 30, and 60 min. The reactions were stopped by addition of a quench solution containing acetonitrile: methanol (1:1) (internal standard at 50 nM). Samples were centrifuged (3500 rpm/30 min) to obtain the pellet of precipitated microsomal protein. The supernatant fractions were quantified by LC–MS/MS. Peak area ratios (analyte/internal standard) were converted to % remaining using the area ratio at time 0 as 100%. The half‐life (*t*1/2 = ln (2)/*k*) in minutes and intrinsic clearance (Clint = *k* × 1000/(0.25)) in μL/min/mg were calculated using a nonlinear regression from % remaining versus incubation time. From this plot, the slope (*k*) was determined. The chromatograms for analysis were achieved the Supelco Ascentis express C18 column (3 cm × 2.1 mm, 5 μM). The mobile phases consisted of water +0.1% formic acid (A) and acetonitrile +0.1% formic acid (B). The mobile phase was eluted in binary gradient mode, and the gradient was as follows: 0 min 95% A; 0.05 min 95% A; 0.3 min: 2% A; 0.7 min: 2% A; 0.8 min: 95% A; 1.15 min 95% A; 2.0 min 95% A. The running time was 2 min, the sample injection volume was 10 μL, and the flow rate was 0.7 mL/min. All microsomal stability tests were performed in triplicate.

### 4.7 Parallel Artificial Membrane Permeability Assays (PAMPA)

The parallel artificial membrane permeability assay was carried out in a 96‐well precoated PAMPA plate system (Corning Gentest # 353015). Solutions of the compounds were prepared by diluting the stock solutions (10 mM) in phosphate‐buffered saline (PBS) pH 6.5 at a final concentration of 10 μM and then added to the donor portion of the plate (300 μL/well), while PBS (pH 7.4 (200 μL/well)) was added to the acceptor portion. The DMSO concentration was lower than 1%. The two portions of the plate were then assembled, and the system was incubated (5 h/37 °C). Samples of the initial donor solution (*T*0) were collected and storage at −20 °C. At the end of the incubation, samples were collected from the donor and acceptor plates and then added to plates containing quench solution (10% water and 90% methanol: acetonitrile (50:50) + 50 nM tolbutamide); the *T*0 samples were treated similarly. The final concentrations of compounds in the donor, acceptor, and *T*0 wells were quantified by LC–MS/MS. The chromatogram for analysis was achieved on a Supelco Ascentis express C18 column (3 cm × 2.1 mm, 5 μM). The mobile phases consisted of water +0.1% formic acid (A) and acetonitrile +0.1% formic acid (B). The mobile phase was eluted in binary gradient mode, and the gradient was as follows: 0 min, 95% A; 0.05 min 95% A; 0.3 min: 2% A; 0.7 min: 2% A; 0.8 min: 95% A; 1.15 min 95% A; 2.0 min 95% A. The run time was 2 min, and the sample injection volume was 10 μL and a flow rate of 0.7 mL/min. The results were 0 min, 95% A; 0.05 min 95% A; 0.3 min: 2% A; 0.7 min: 2% A; 0.8 min: 95% A; 1.15 min 95% A; 2.0 min 95% A. The run time was 2 min, and the sample injection volume was 10 μL and a flow rate of 0.7 mL/min. The results were used to calculate an effective permeability (Pe) value. All PAMPA assays were performed in triplicate.

### 4.8 Kinetic Solubility

To determine kinetic solubility, stock solutions of test compounds and controls (10 mM in DMSO) were transferred to two 96‐well plate (incubation plate) in duplicate; for each sample on the plates, PBS pH 7.4 or 2.0 (final concentration of 250 μM) was added, and DMSO concentration was lower than 2.5%. The plate was sealed and shaken for 24 h (200 rpm/25 °C). The precipitates on the incubation plate were removed by centrifugation (15 min/3000 rpm/25 °C). The supernatant fractions were quantified by LC–MS/MS. From 10 mM standard solution, an intermediate standard dilution at 0.5 mM in 1:1 acetonitrile‐to‐water solution was prepared. A calibration curve was prepared for each test compound and control by diluting several times the intermediate standard to reach the desired concentration of 50, 40, 20, 2, and 1 μM. The resulting equation for the calibration curve (*y* = *m* *x* + *b*) was used to calculate the experimental concentration values for the compounds. The chromatogram for analysis was achieved on a Supelco Ascentis express C18 column (3 cm × 2.1 mm, 5 μM). The mobile phases consisted of water +0.05% formic acid (A) and acetonitrile +0.05% formic acid (B). The mobile phase was eluted in binary gradient mode, and the gradient was as follows: 0 min, 98% A; 1.2 min 2% A; 2.0 min: 2% A; re‐equilibration time: 0.6 min, 98% A. The run time was 2 min, and the sample injection volume was 5 μL and a flow rate of 0.6 mL/min.

## Funding

This study was supported by the Fundação de Amparo à Pesquisa do Estado de Minas Gerais (FAPEMIG, grants RED‐00110‐23 and APD‐01401‐25), the Conselho Nacional de Desenvolvimento Científico e Tecnológico (CNPq, grants 309994‐2023‐3, 316892/2023‐8, 300079/2025‐7, and 408678/2024‐0), the Fundação de Amparo à Pesquisa do Estado de São Paulo (FAPESP‐CIBFar, grant 13/07600‐3), and the Fundação de Apoio à Pesquisa do Distrito Federal (FAPDF, grant 00193‐00002136/2023‐01).

## Conflicts of Interest

The authors declare no conflicts of interest.

## Supporting information

NMR and HRMS spectra of compounds hit **1** and **4**–**30** can be found in the Supporting Information.

## Data Availability

Research data are not shared.
