# Supplementary material for: Synthesis, Antiparasitic Activity, and ADME Profile of Morpholinephenylbenzamides: A Pharmacological Study Against Chagas Disease and Visceral Leishmaniasis
Source: ChemMedChem. 2026 Oct 8;21(19):e70518. doi: 10.1002/cmdc.70518 (PMC13650381; doi:10.1002/cmdc.70518)
Supplement: Supplementary file 1 [file CMDC-21-e70518-s001.pdf]

Supporting Information for

Synthesis, antiparasitic activity, and ADME profile of morpholinephenylbenzamides: a pharmacological study against Chagas disease and visceral leishmaniasis

Danilo Nascimento Farago[a], Daniela de Melo Resende[b], Mariza Gabriela Faleiro de Moura Lodi Cruz[b], Rafaela Marçal Sousa[a], Rafael Consolin Chelucci[c], Simone Michelan-Duarte[c], Felipe da Silva Mendonça de Melo[d], Douglas Davison da Silva Oliveira[a], Paula Derksen Macruz[e], Alexandra Maria dos Santos Carvalho[d], Eduardo Jorge Pilau[e], Izabela Marques Dourado Bastos[d], Adriano D. Andricopulo[c], Leonardo L. G. Ferreira[c], Silvane Maria Fonseca Murta[b*], Celso de Oliveira Rezende Júnior[a*]

[*] Corresponding author: silvane.murta@fiocruz.br and celso@ufu.br

1. NMR spectra of compounds hit 1 and 4 - 30

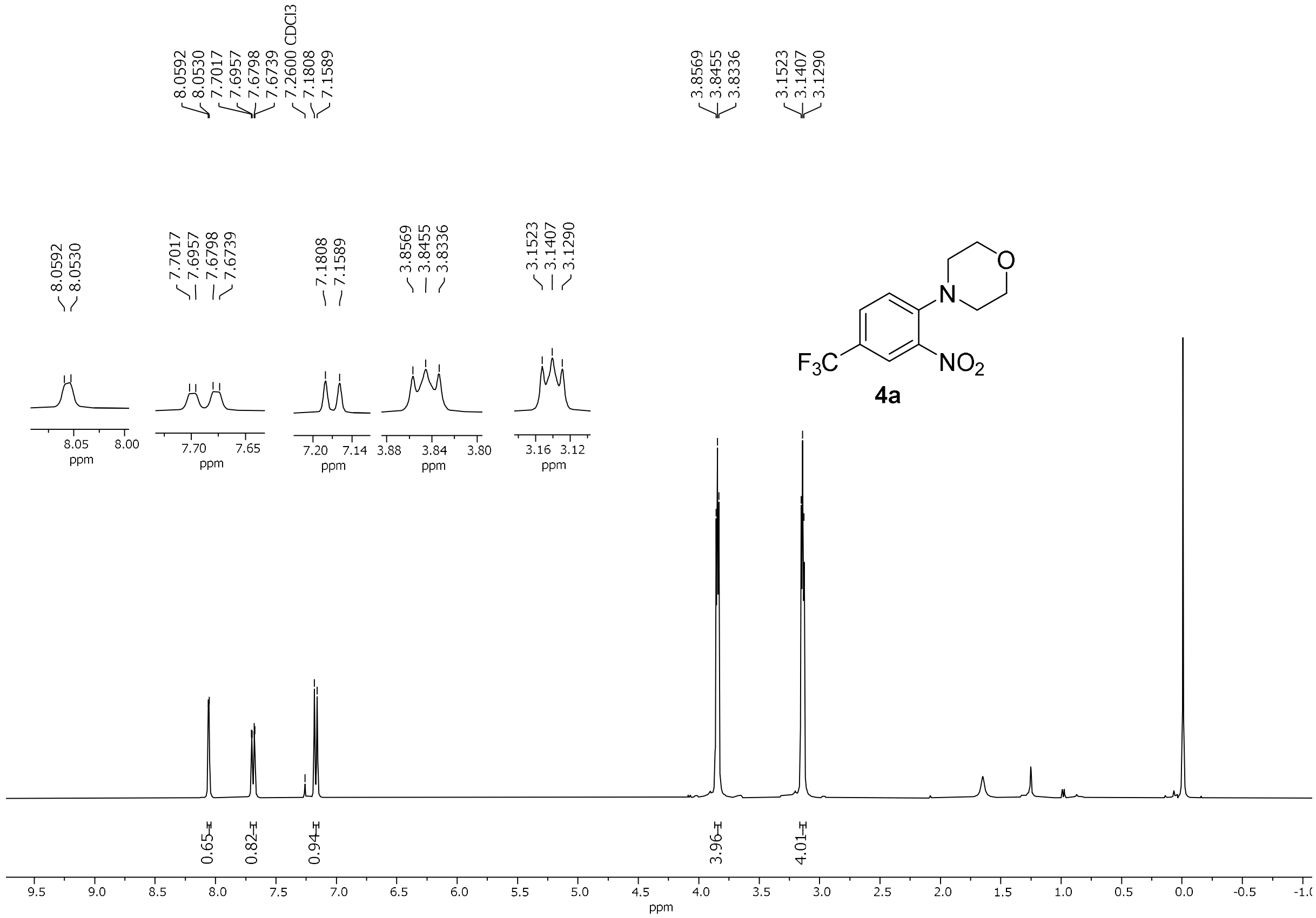

Figure S1. ^{1}H NMR spectrum of **4a** (400 MHz, $CDCl_3$)

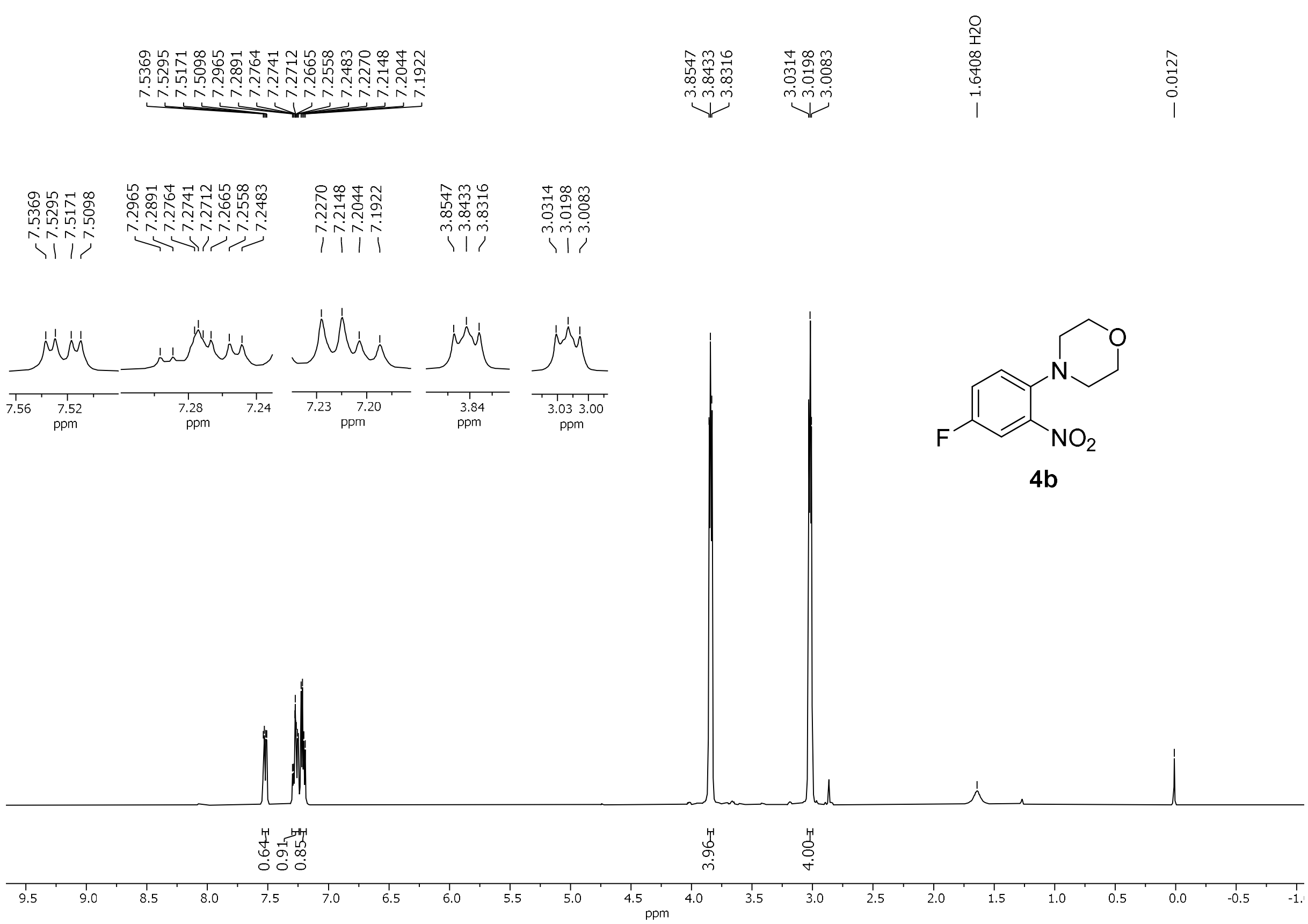

Figure S2. ^{1}H NMR spectrum of **4b** (101 MHz, $CDCl_3$)

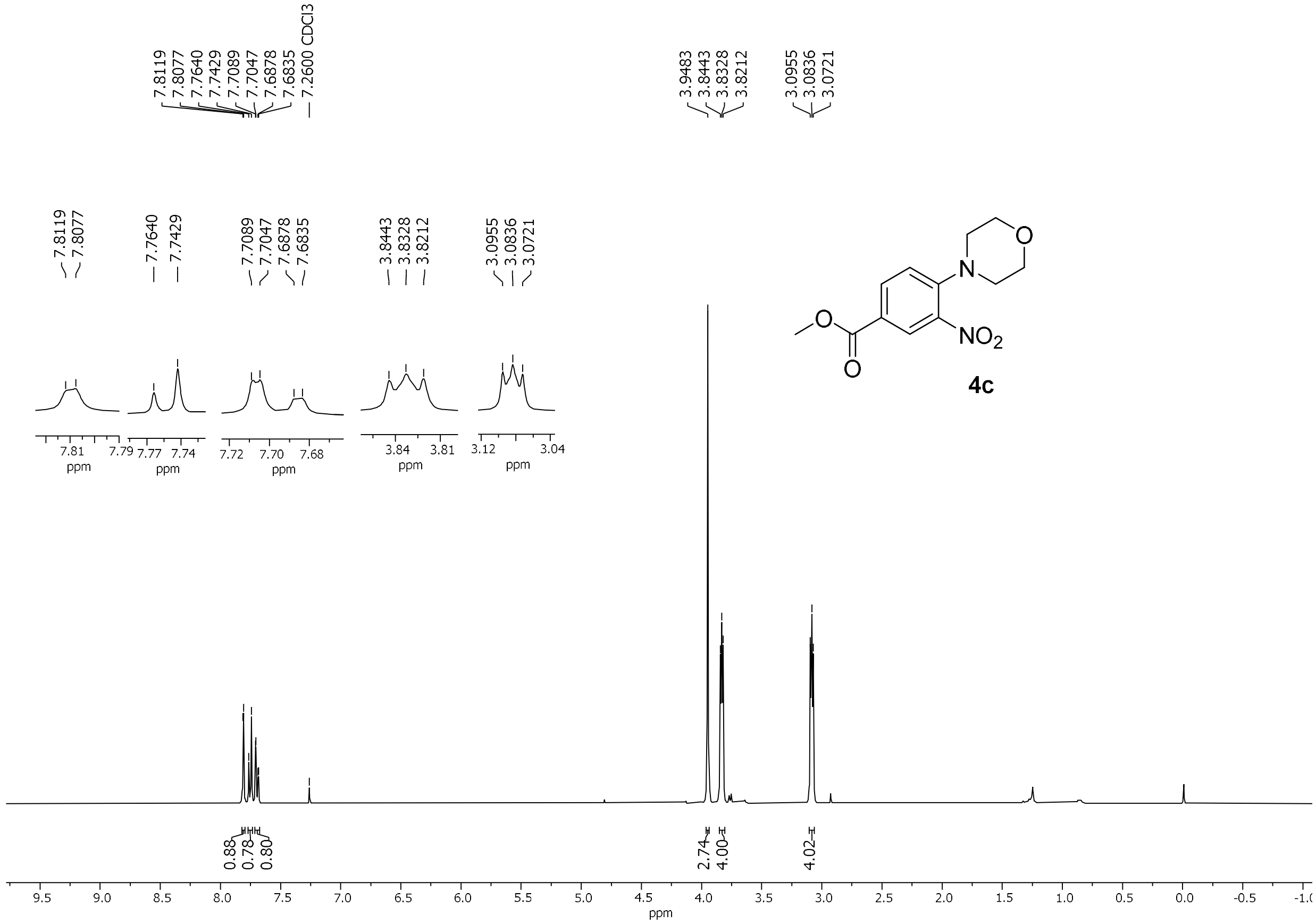

Figure S3. ^{1}H NMR spectrum of **4c** (400 MHz, $CDCl_3$)

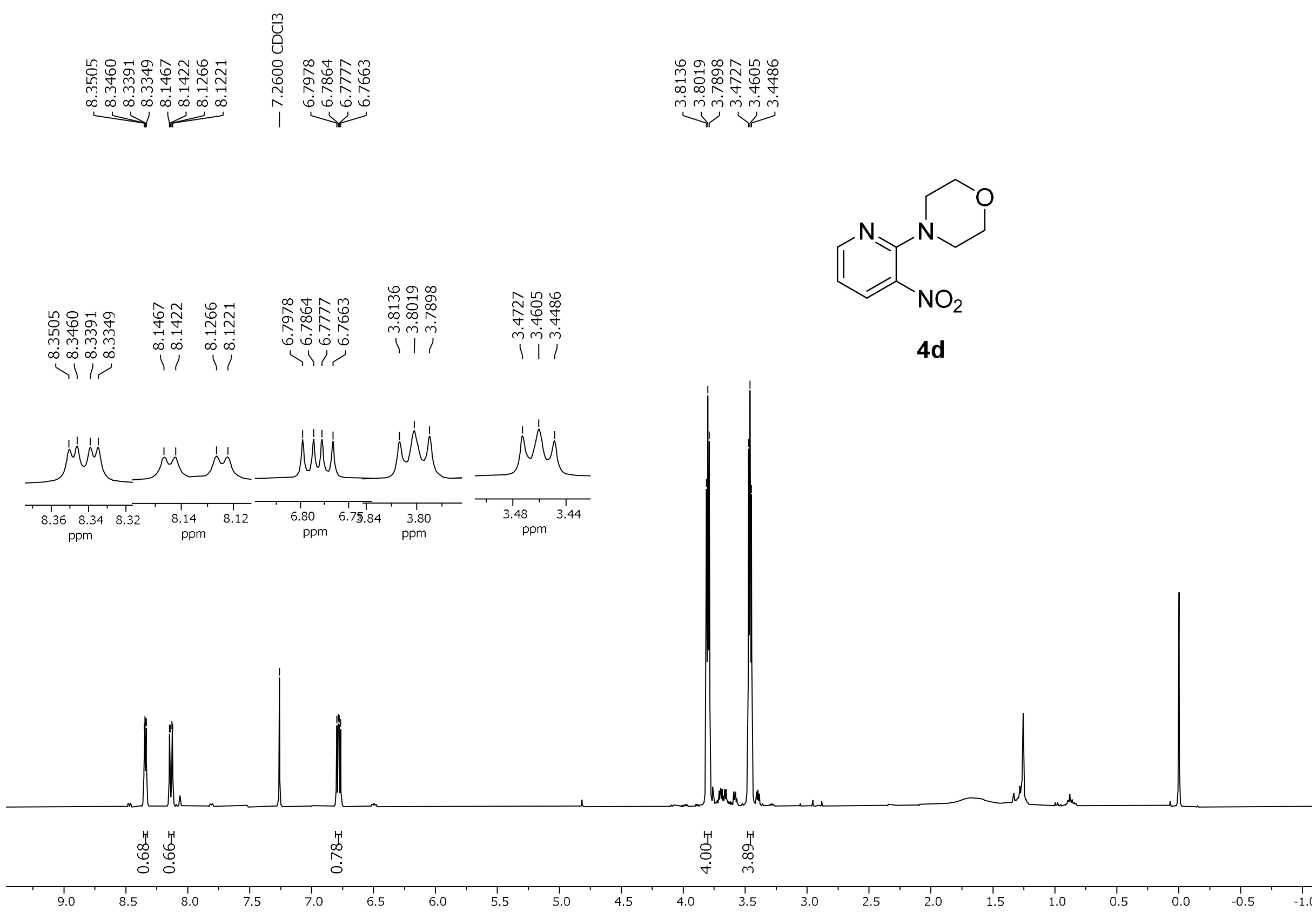

Figure S4. ^{1}H NMR spectrum of **4d** (400 MHz, $CDCl_3$)

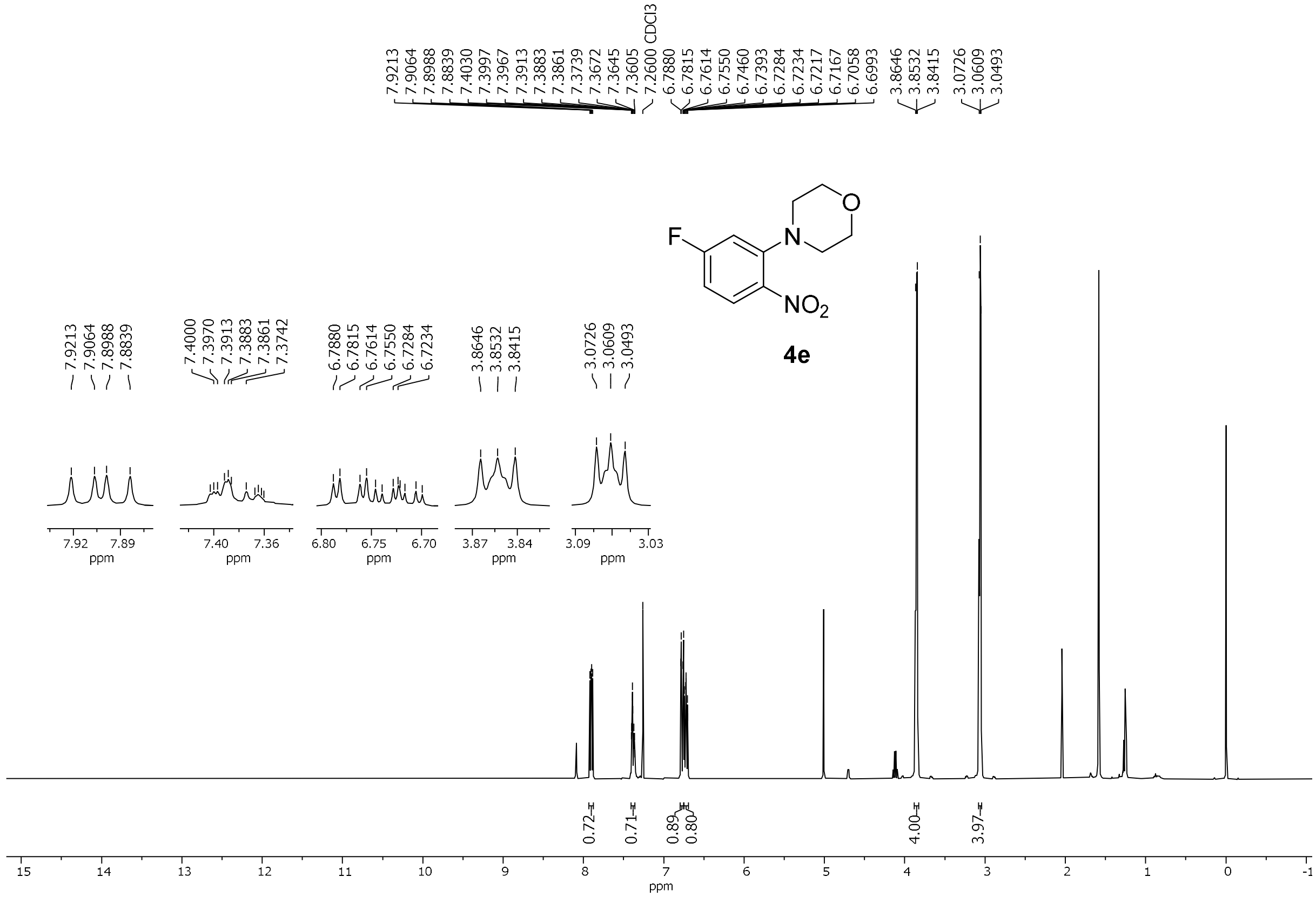

Figure S5. ^{1}H NMR spectrum of **4e** (400 MHz, $CDCl_3$)

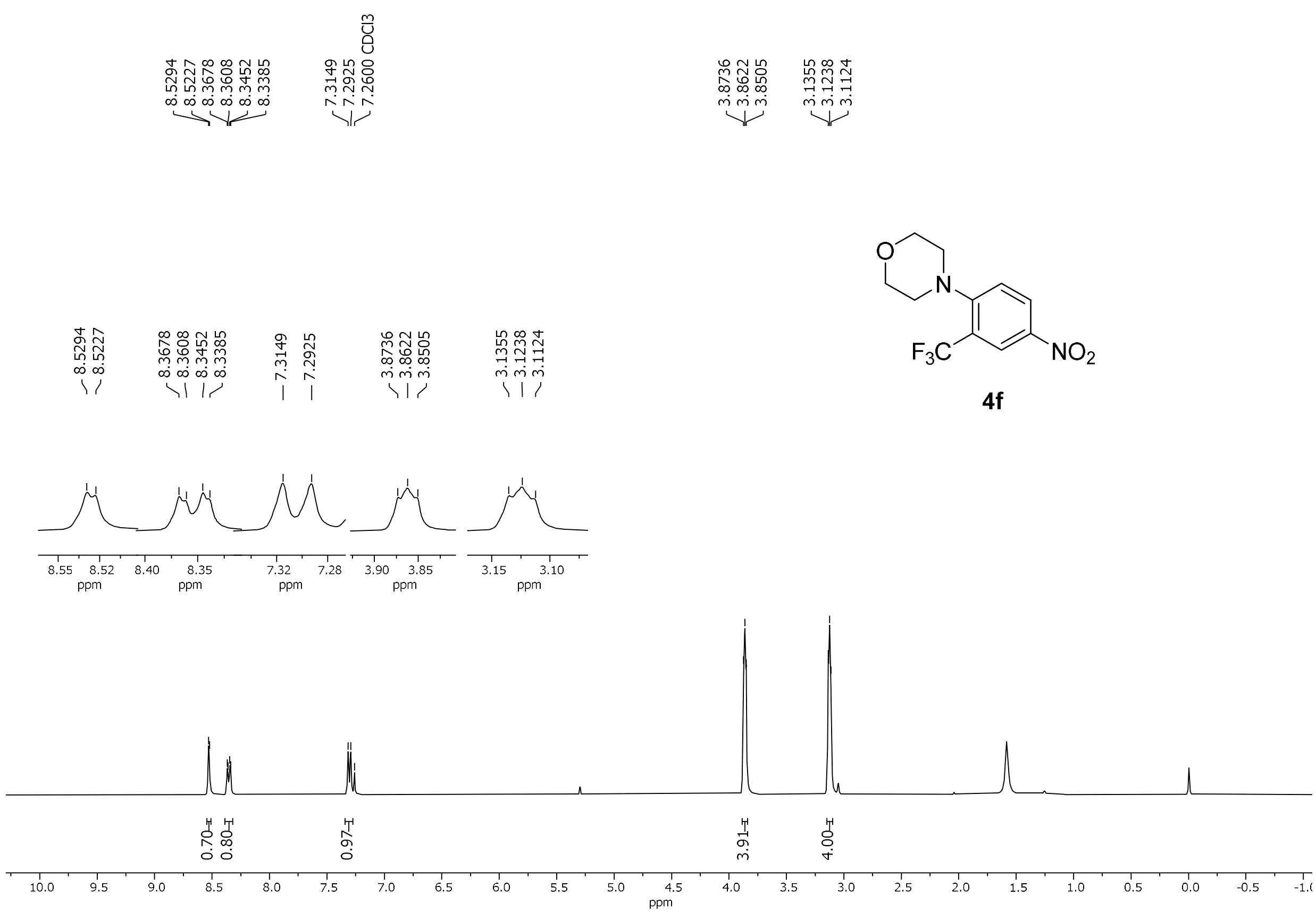

Figure S6. ^{1}H NMR spectrum of **4f** (400 MHz, $CDCl_3$)

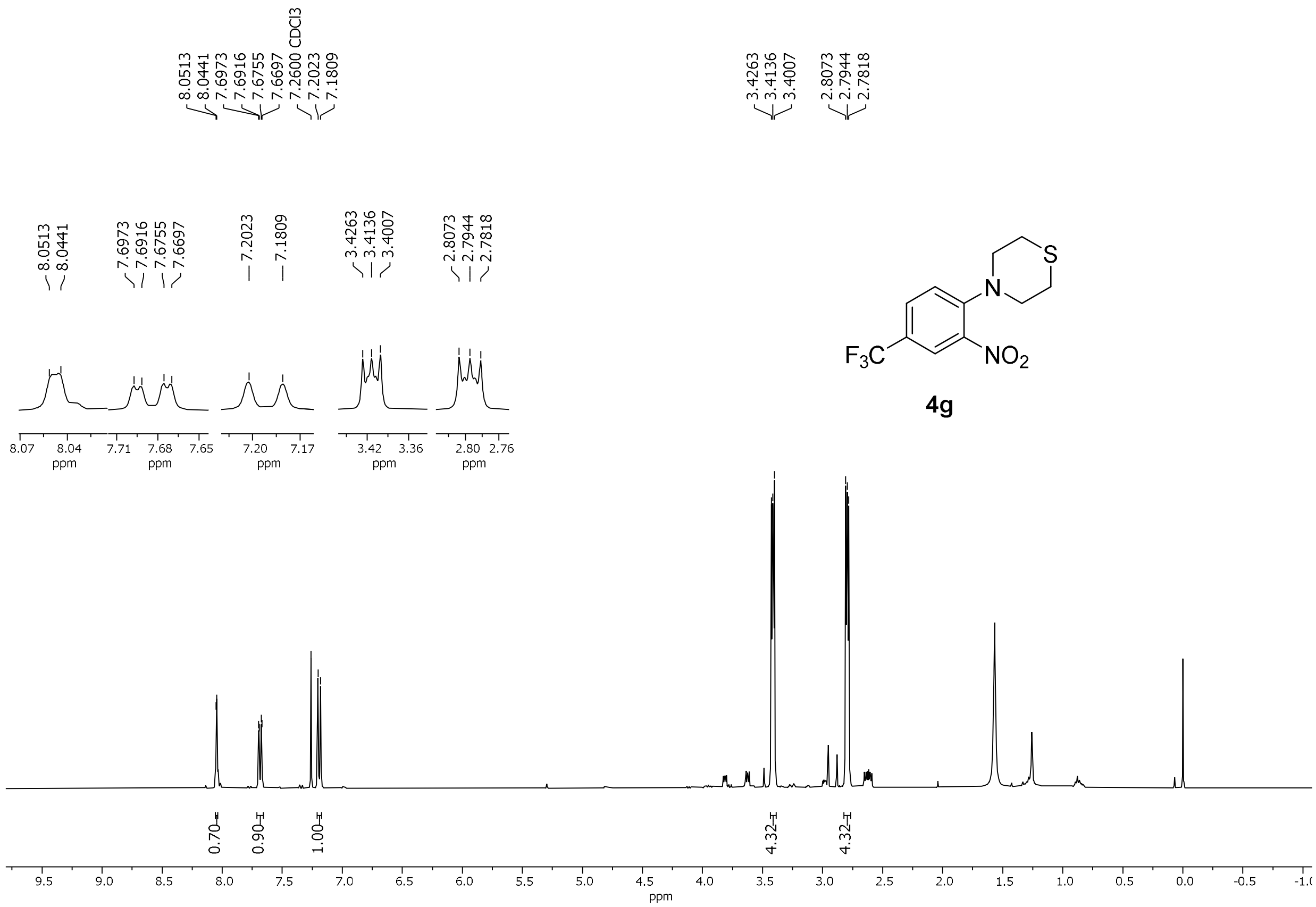

Figure S7. ^{1}H NMR spectrum of **4g** (400 MHz, $CDCl_3$)

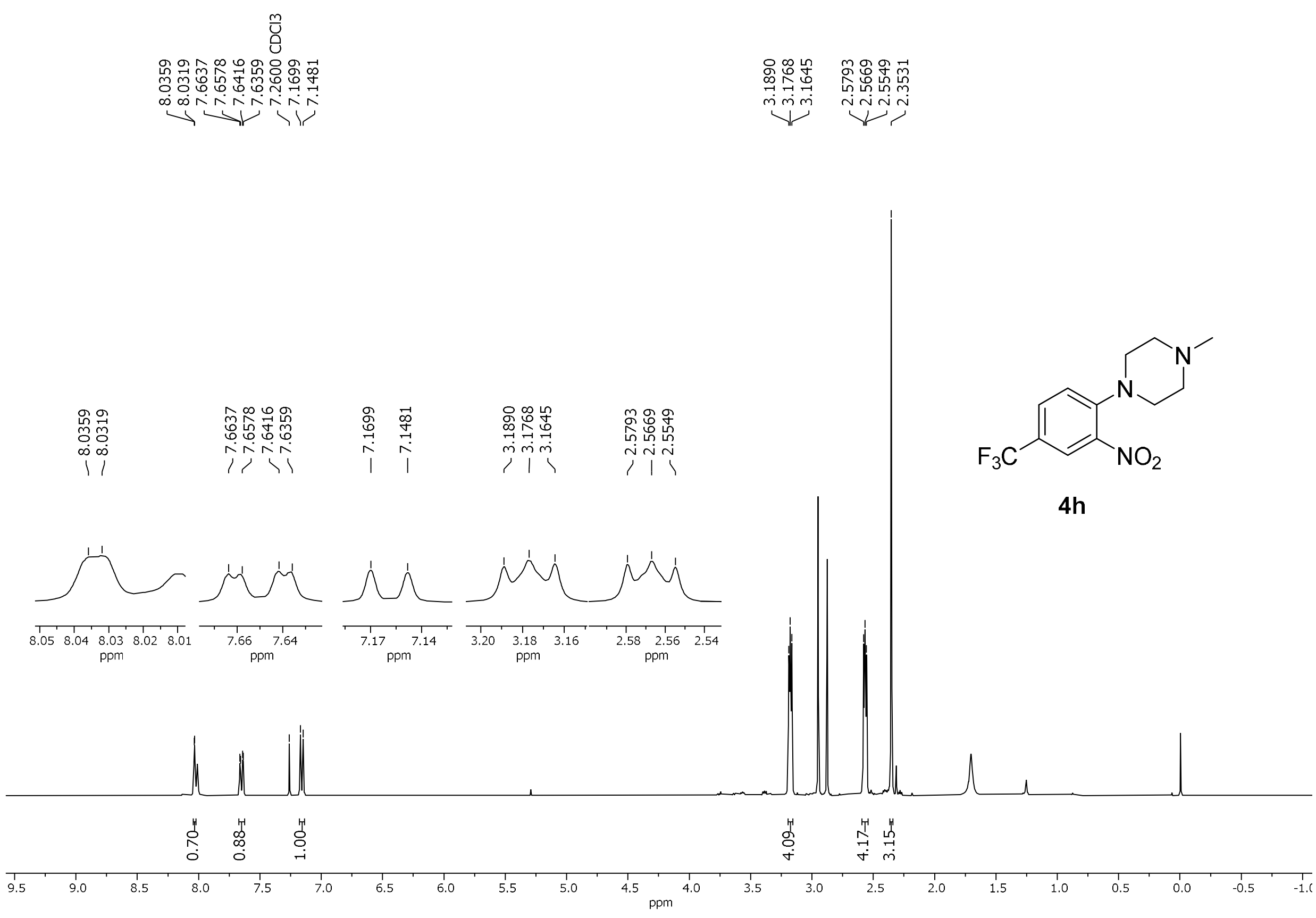

Figure S8. ^{1}H NMR spectrum of **4h** (400 MHz, $CDCl_3$)

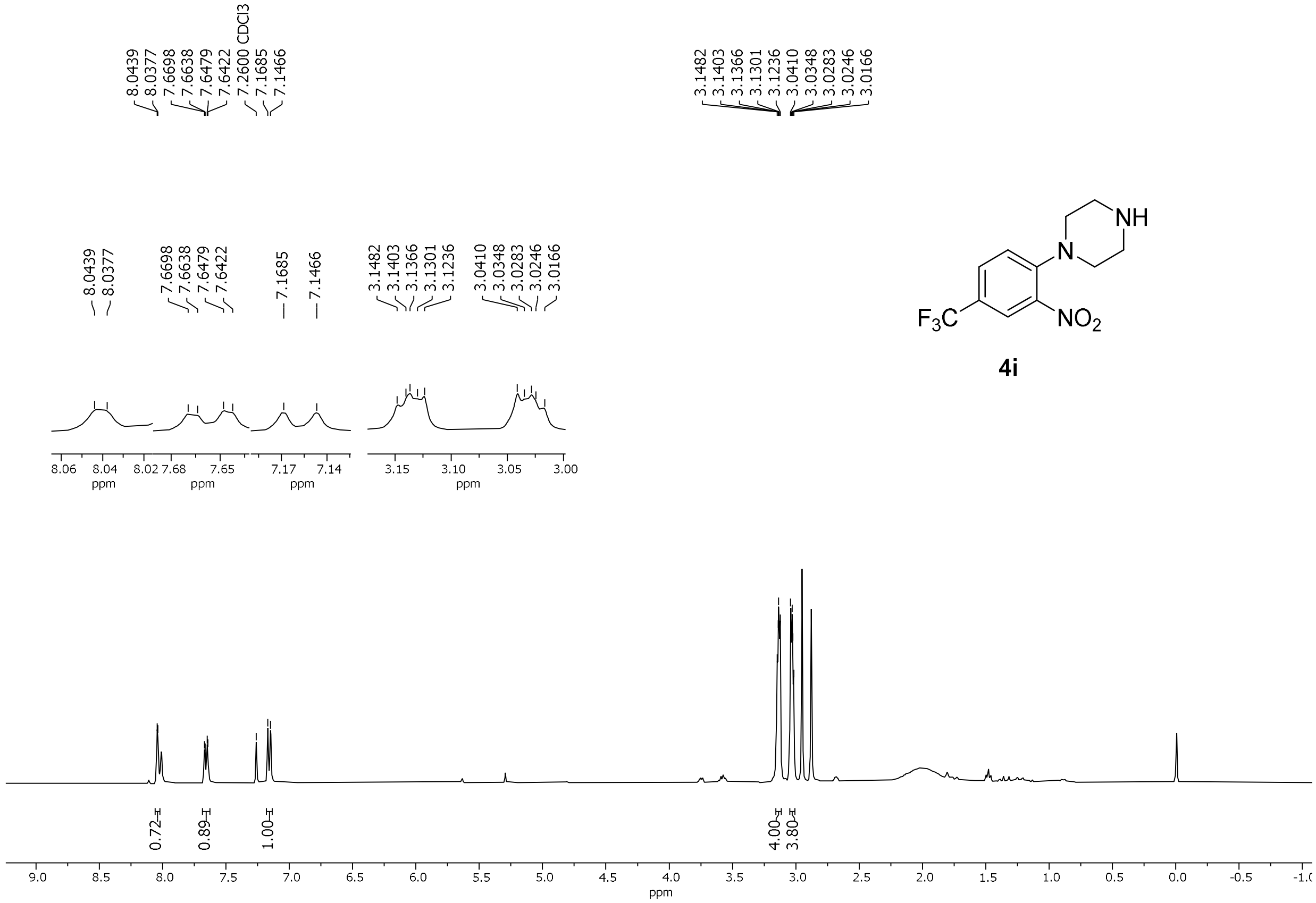

Figure S9. ^{1}H NMR spectrum of **4i** (400 MHz, $CDCl_3$)

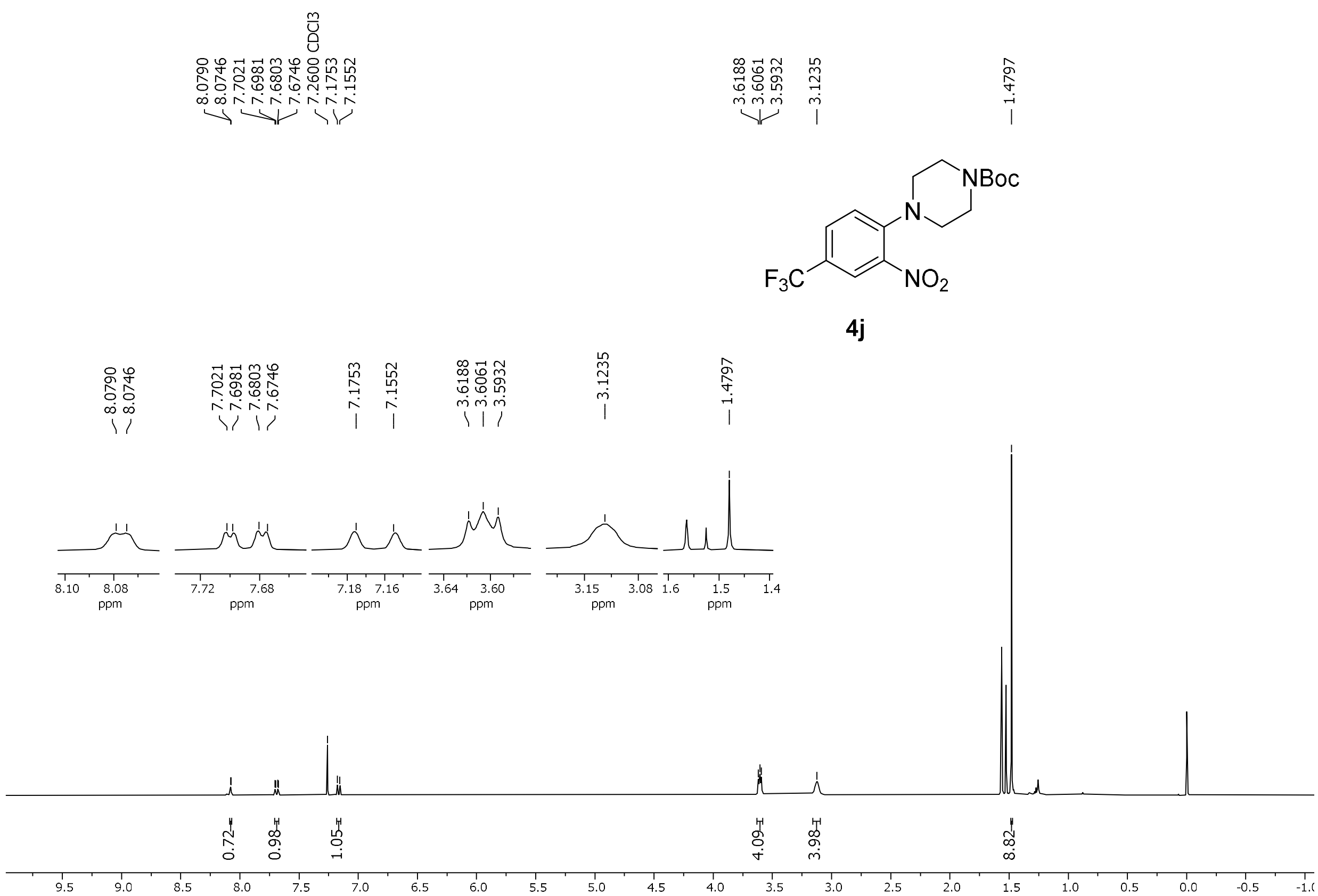

Figure S10. ^{1}H NMR spectrum of **4j** (400 MHz, $CDCl_3$)

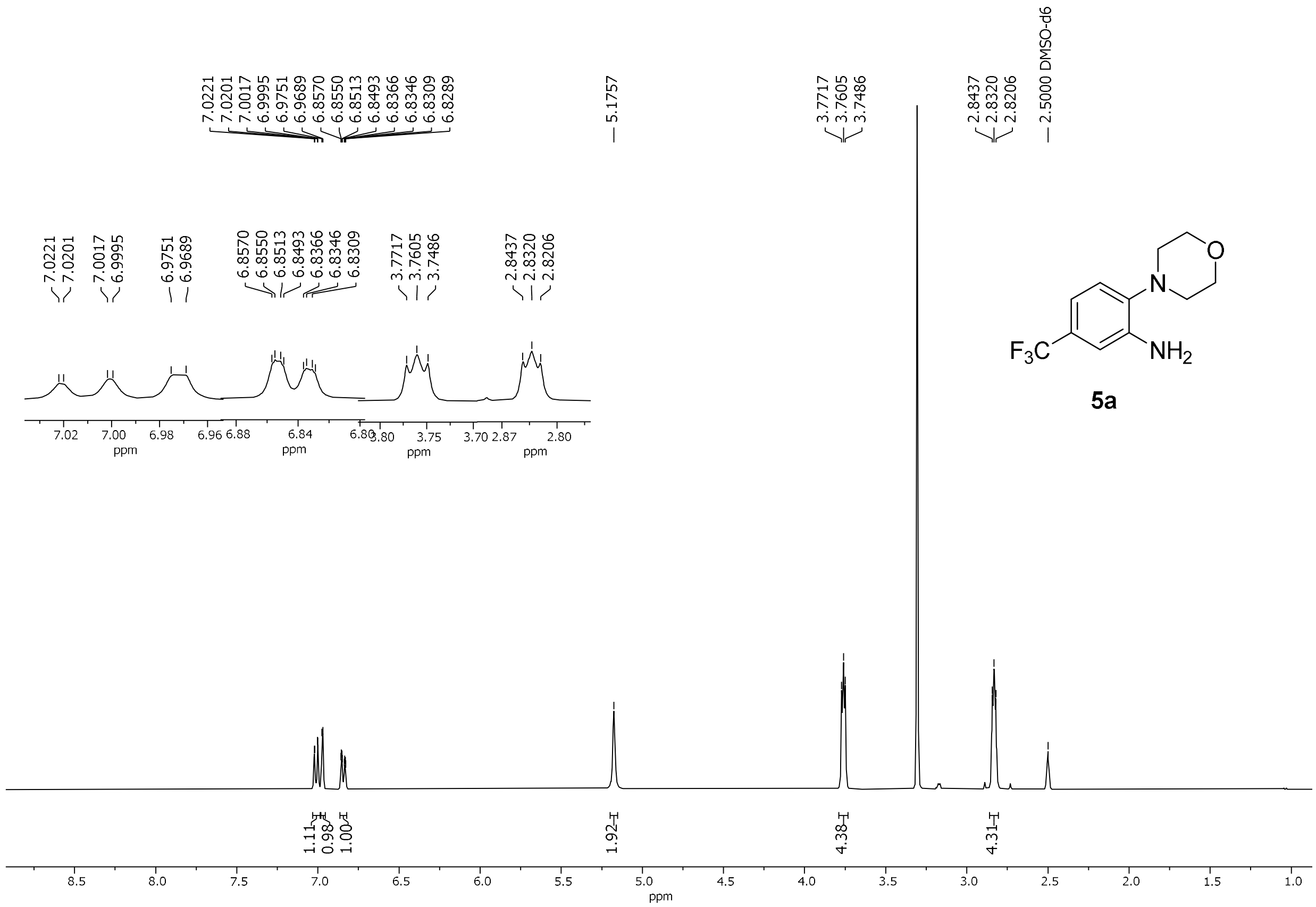

Figure S11. ^{1}H NMR spectrum of **5a** (400 MHz, $CDCl_3$)

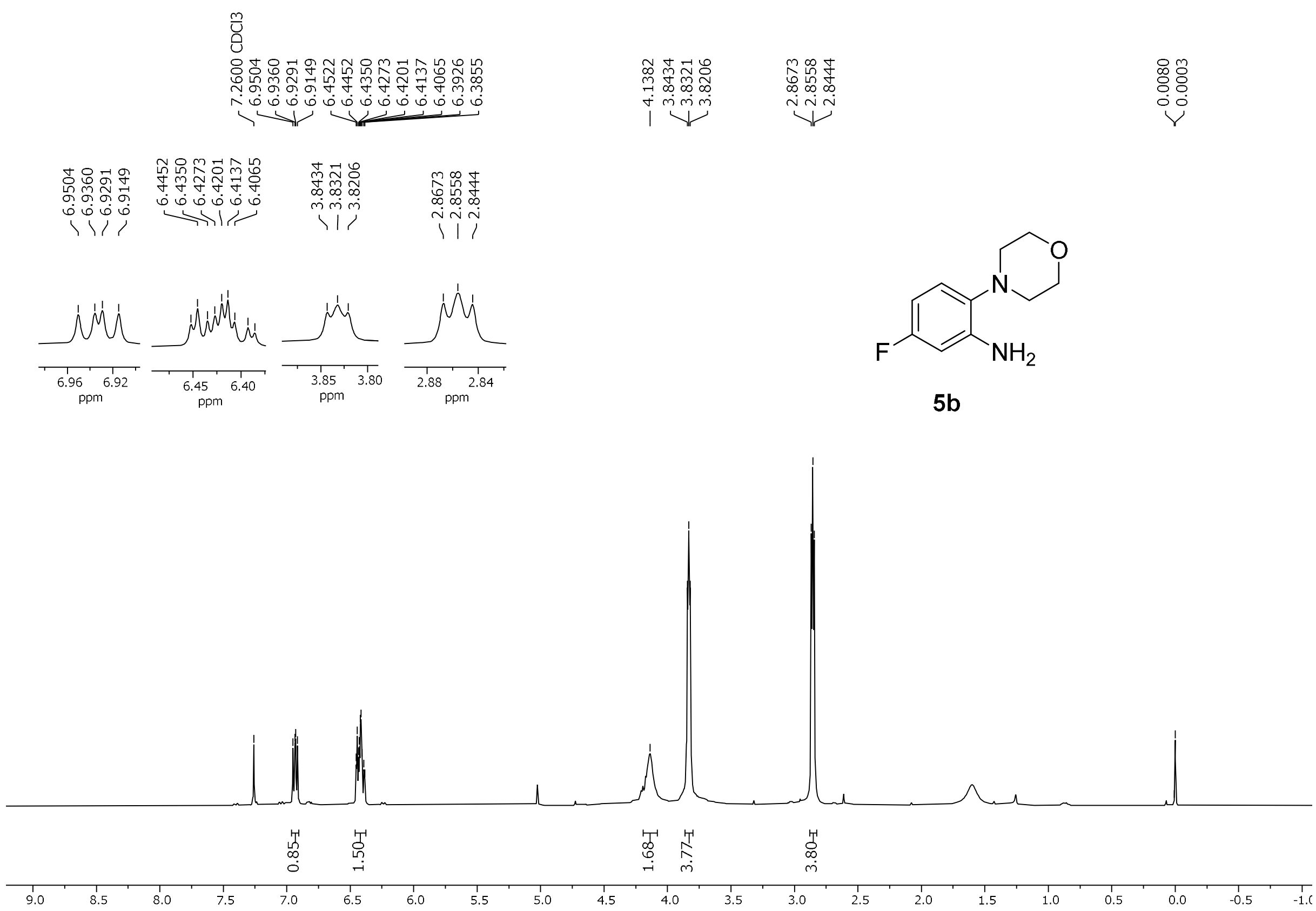

Figure S12. ^{1}H NMR spectrum of **5b** (400 MHz, $CDCl_3$)

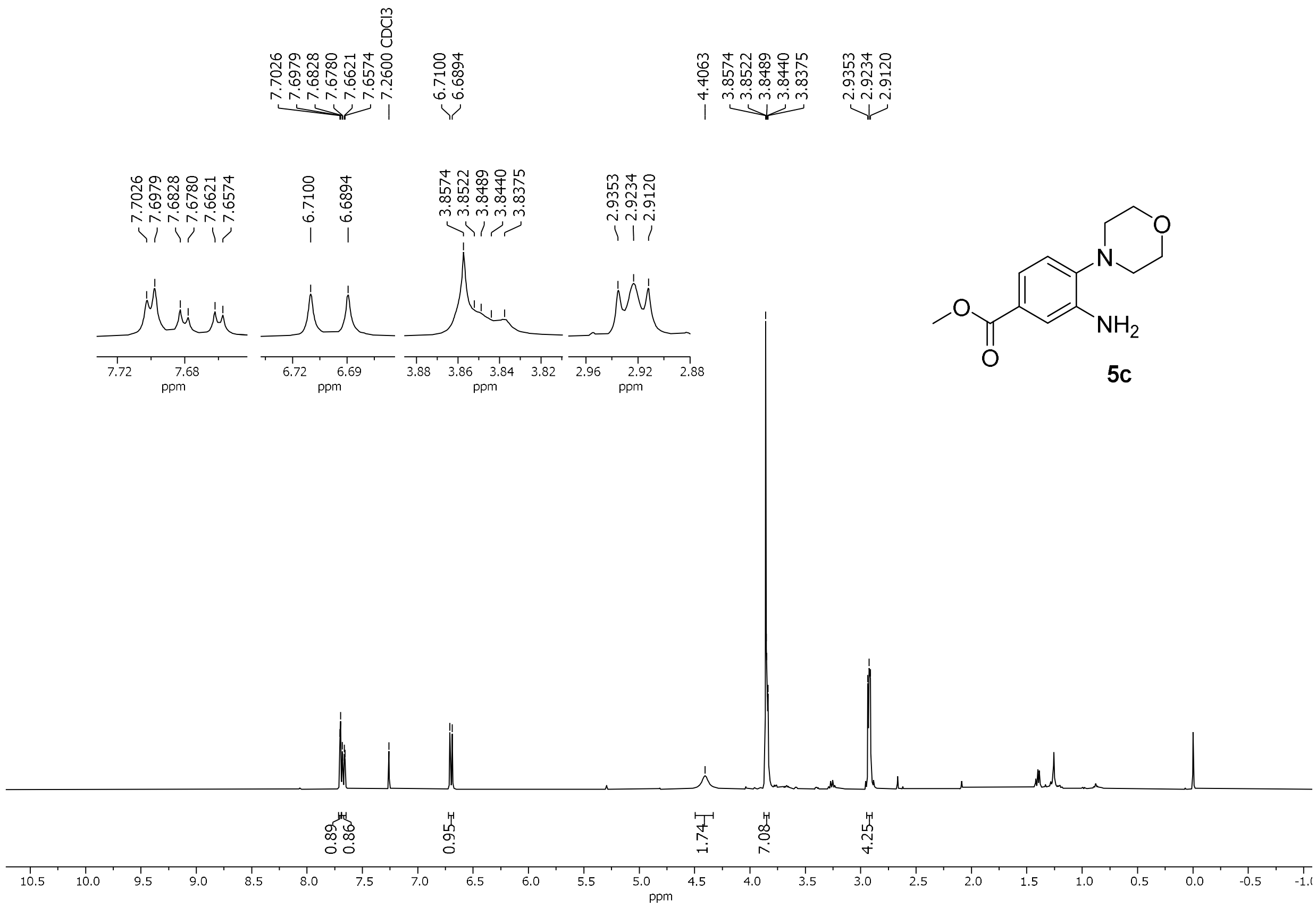

Figure S13. ^{1}H NMR spectrum of **5c** (400 MHz, $CDCl_3$)

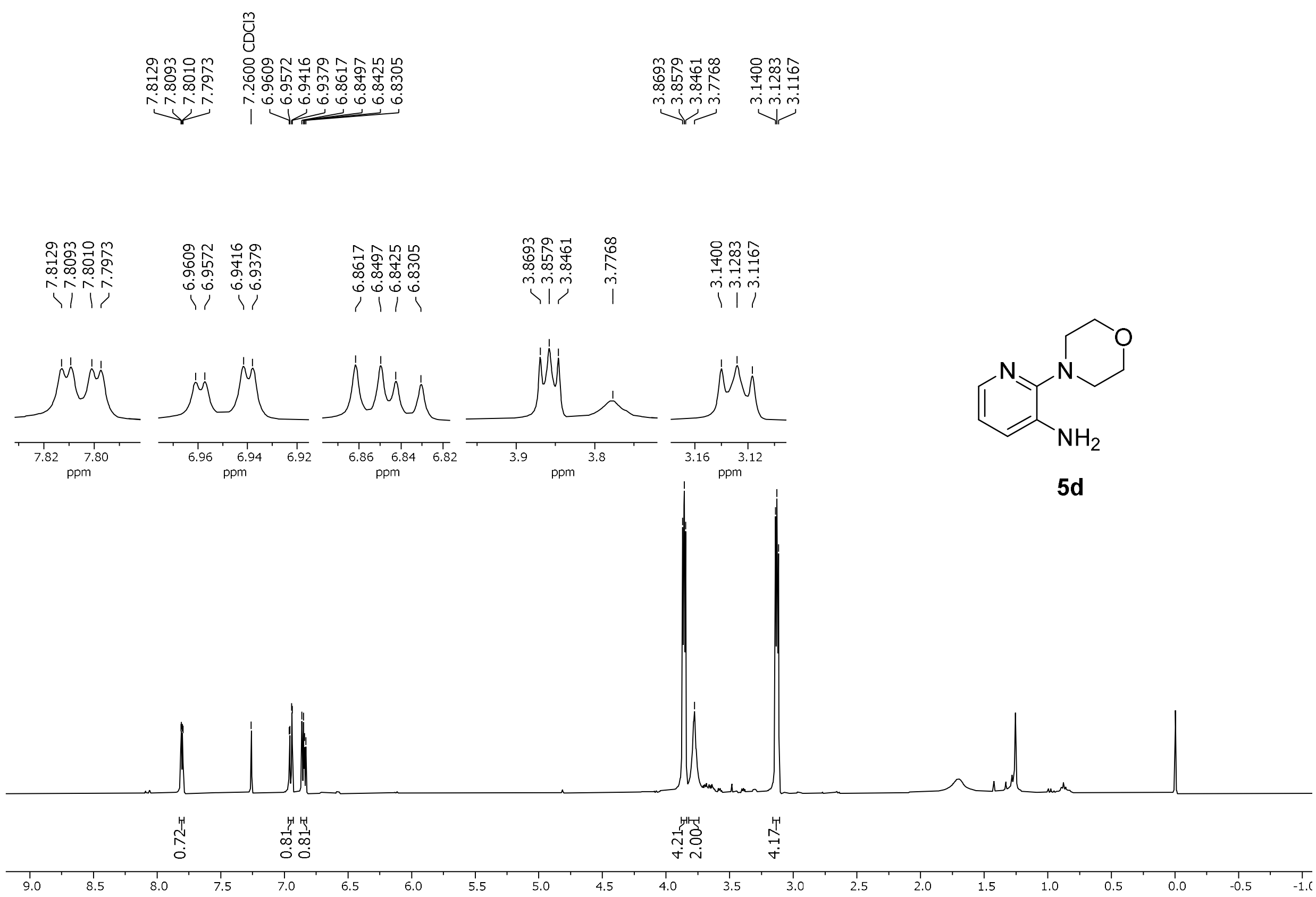

Figure S14. ^{1}H NMR spectrum of **5d** (400 MHz, $CDCl_3$)

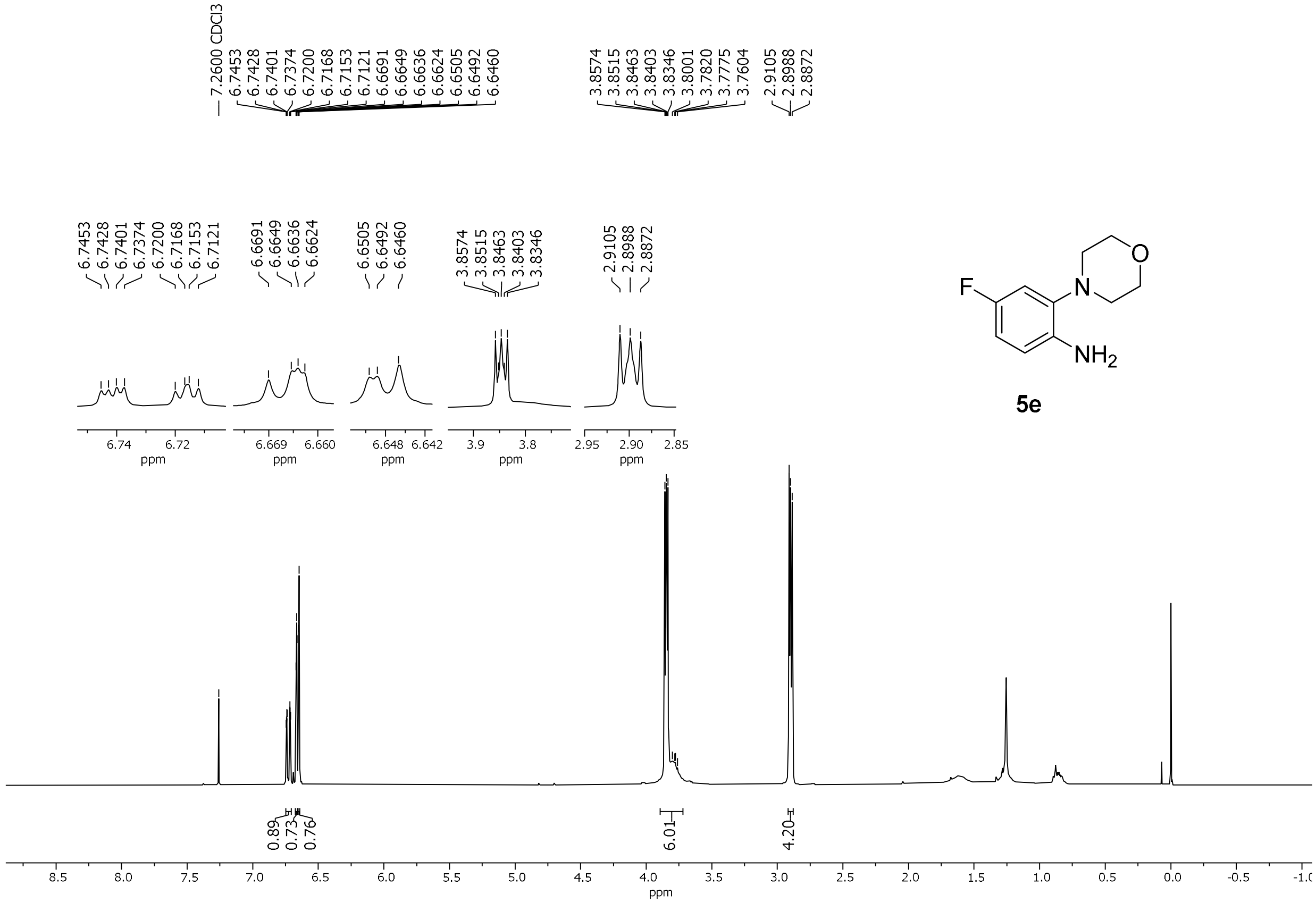

Figure S15. ^{1}H NMR spectrum of **5e** (400 MHz, $CDCl_3$)

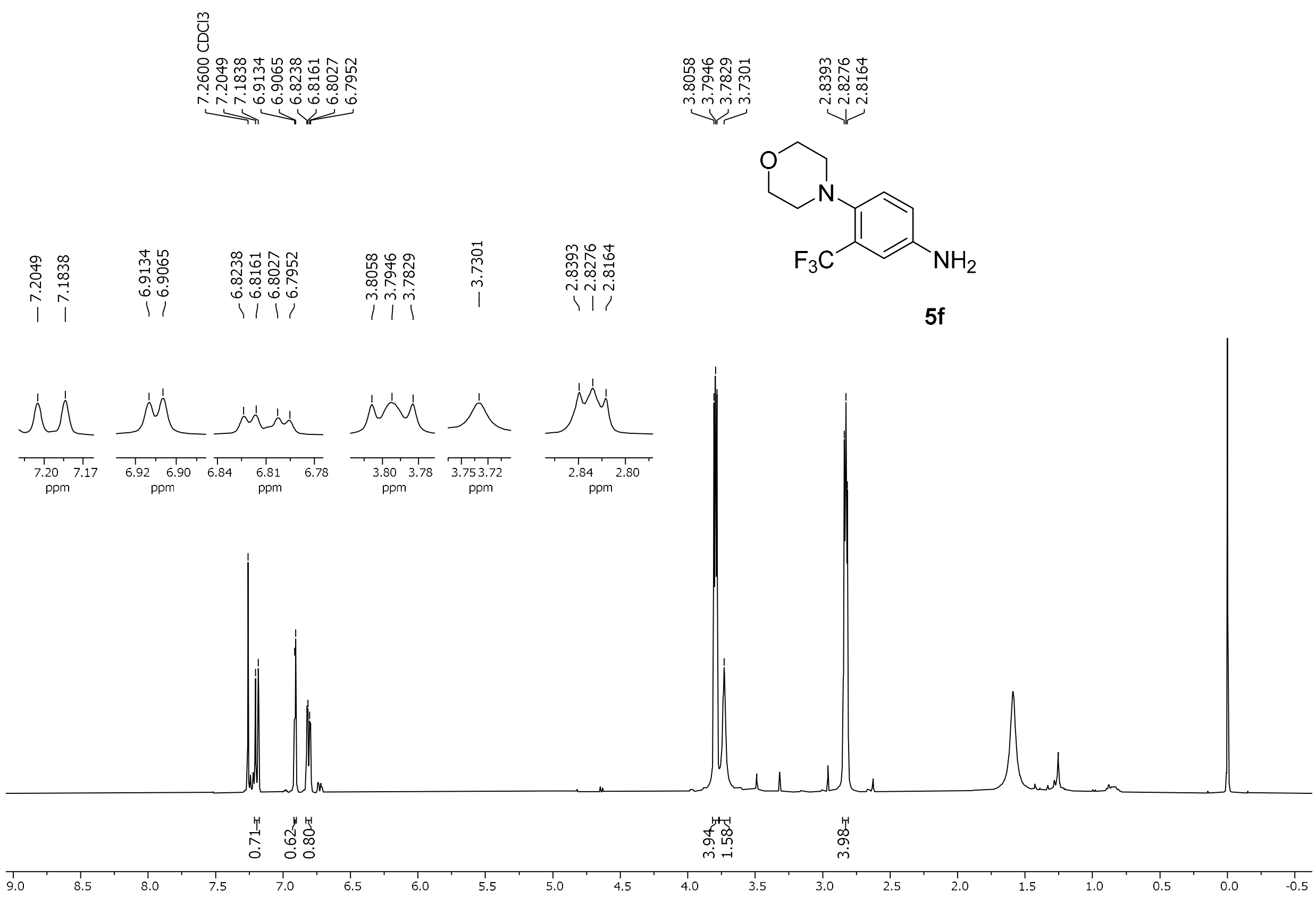

Figure S16. ^{1}H NMR spectrum of **5f** (400 MHz, $CDCl_3$)

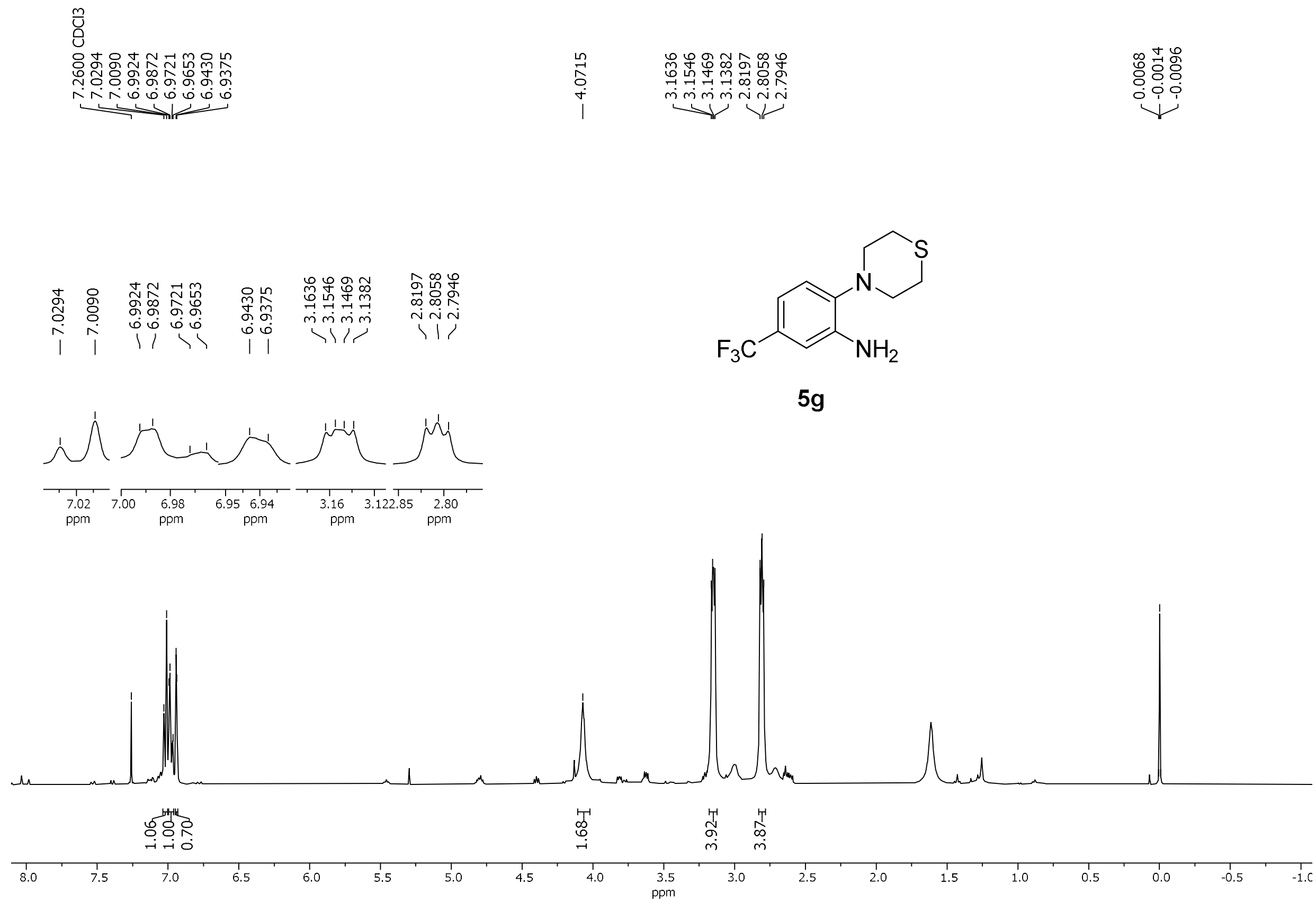

Figure S17. ^{1}H NMR spectrum of **5g** (400 MHz, $CDCl_3$)

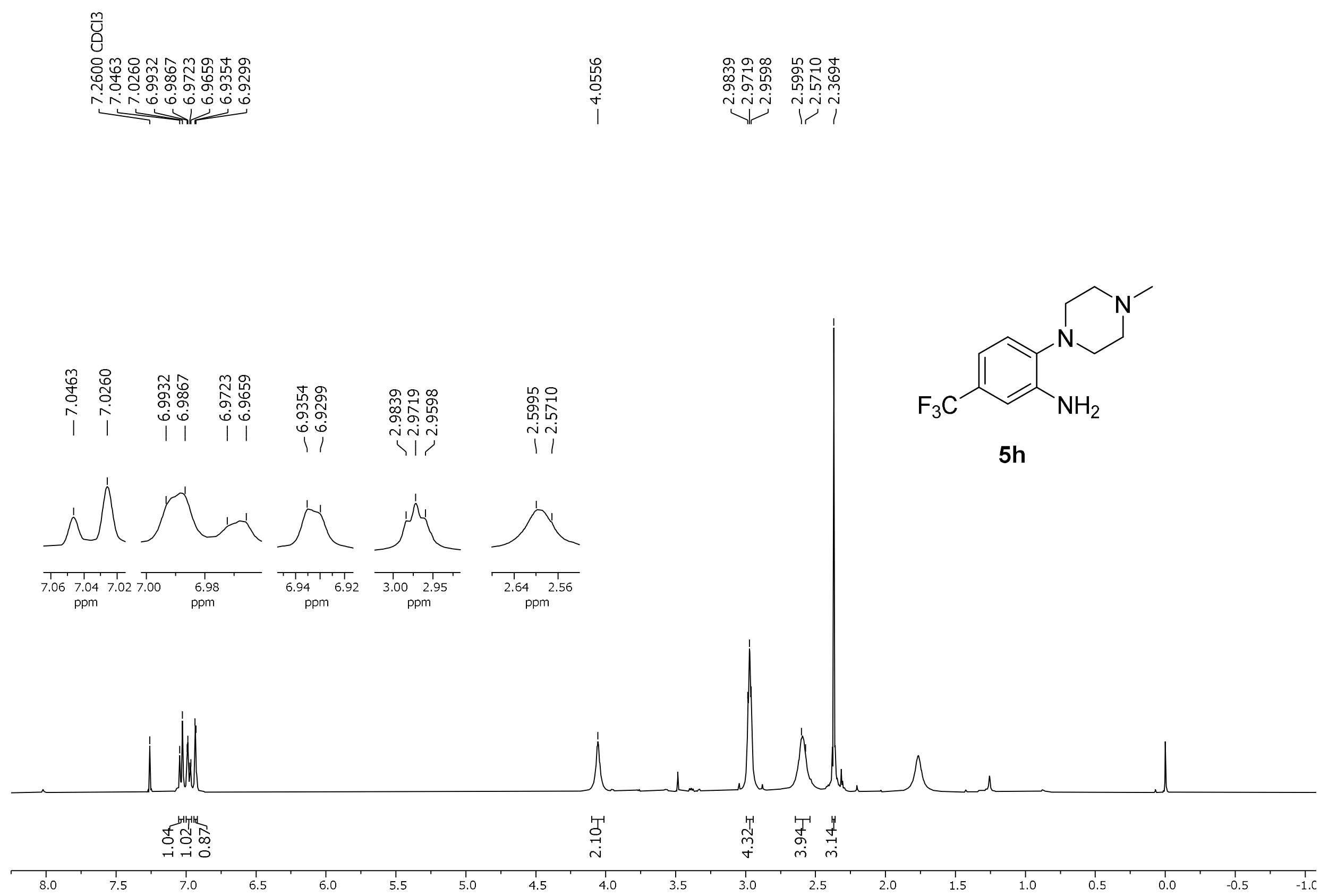

Figure S18. ^{1}H NMR spectrum of **5h** (400 MHz, $CDCl_3$)

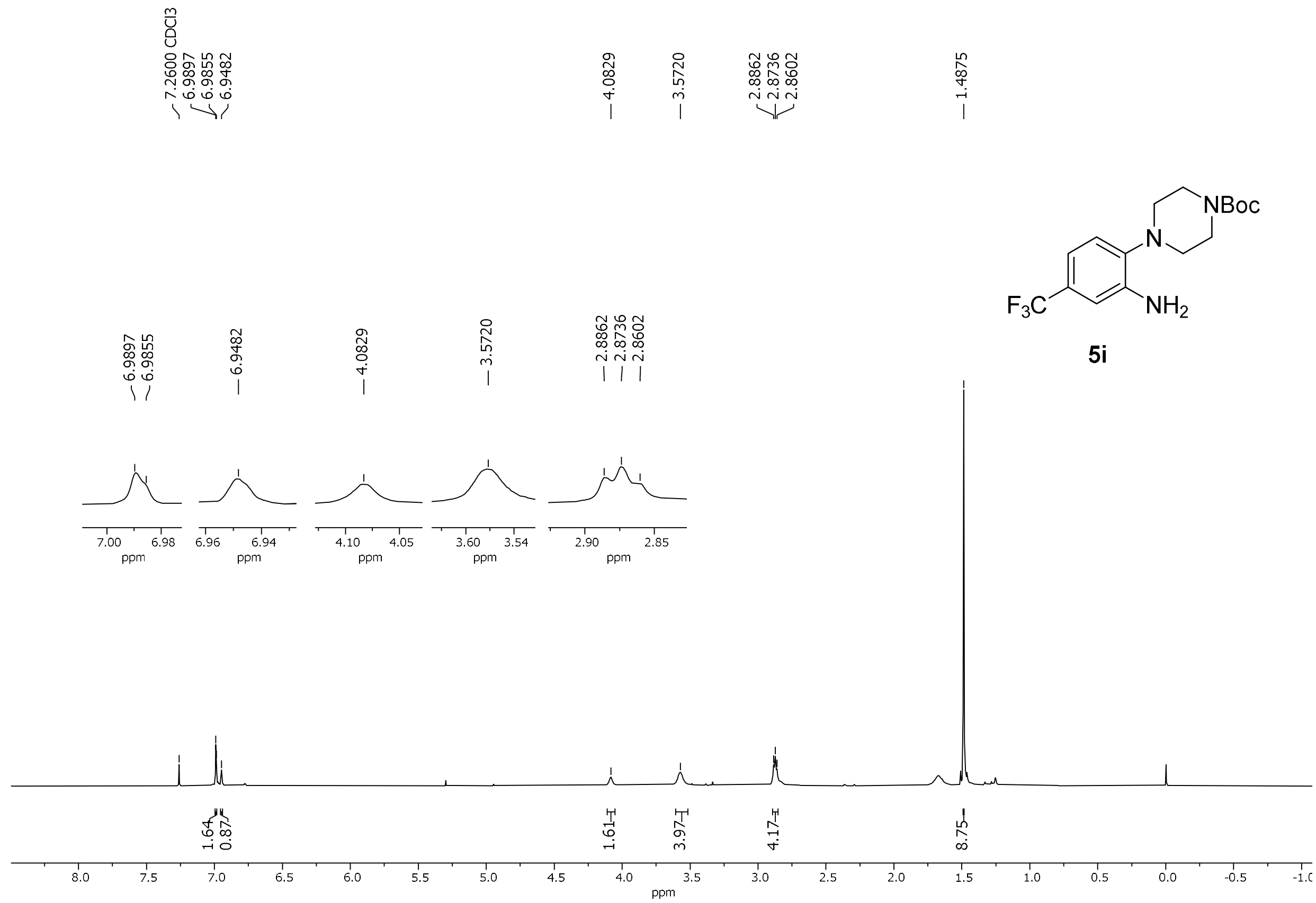

Figure S19. ^{1}H NMR spectrum of **5i** (400 MHz, $CDCl_3$)

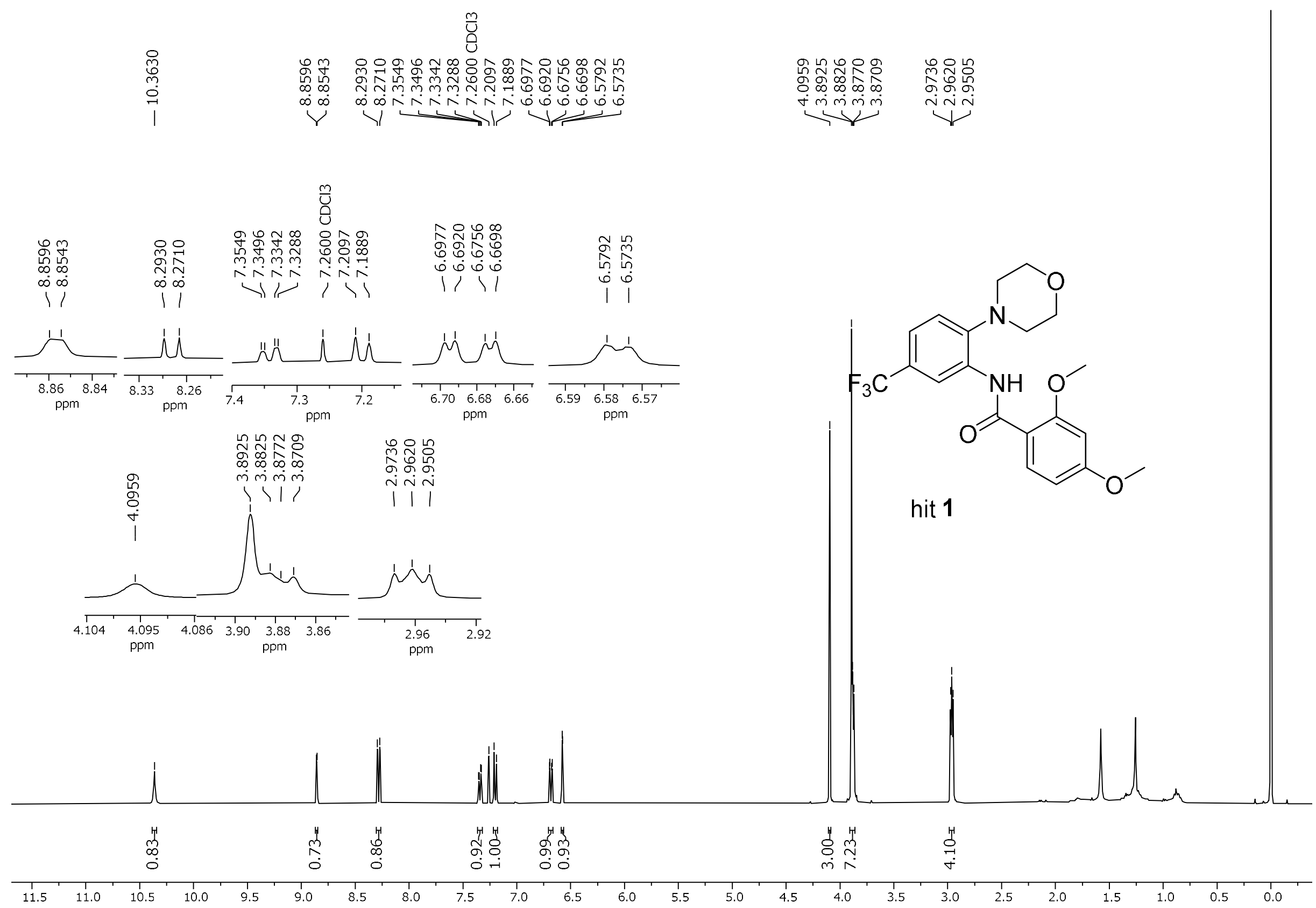

Figure S20. ^{1}H NMR spectrum of hit **1** (400 MHz, $CDCl_3$)

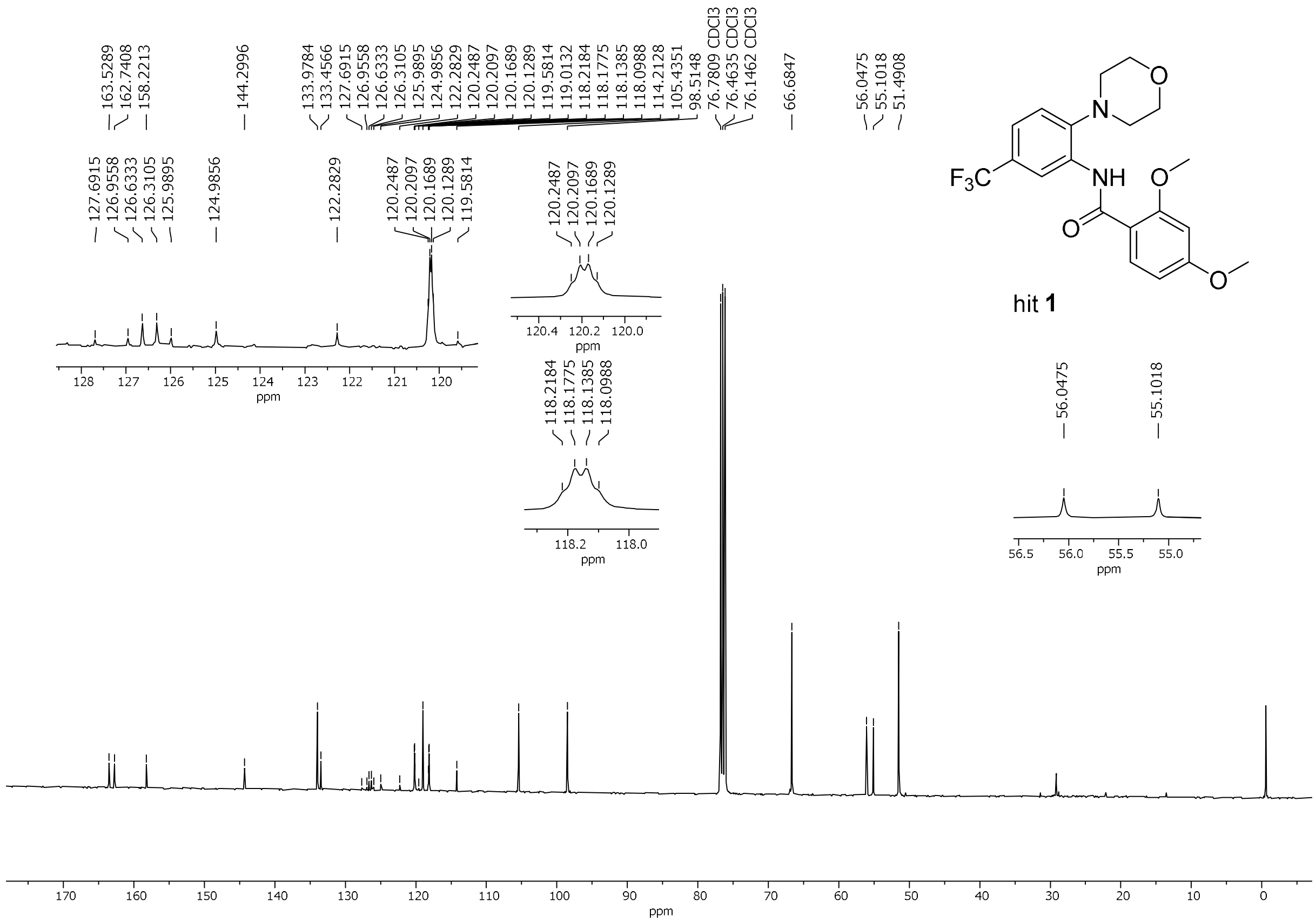

Figure S21. ^{13}C NMR spectrum of hit **1** (101 MHz, $CDCl_3$)

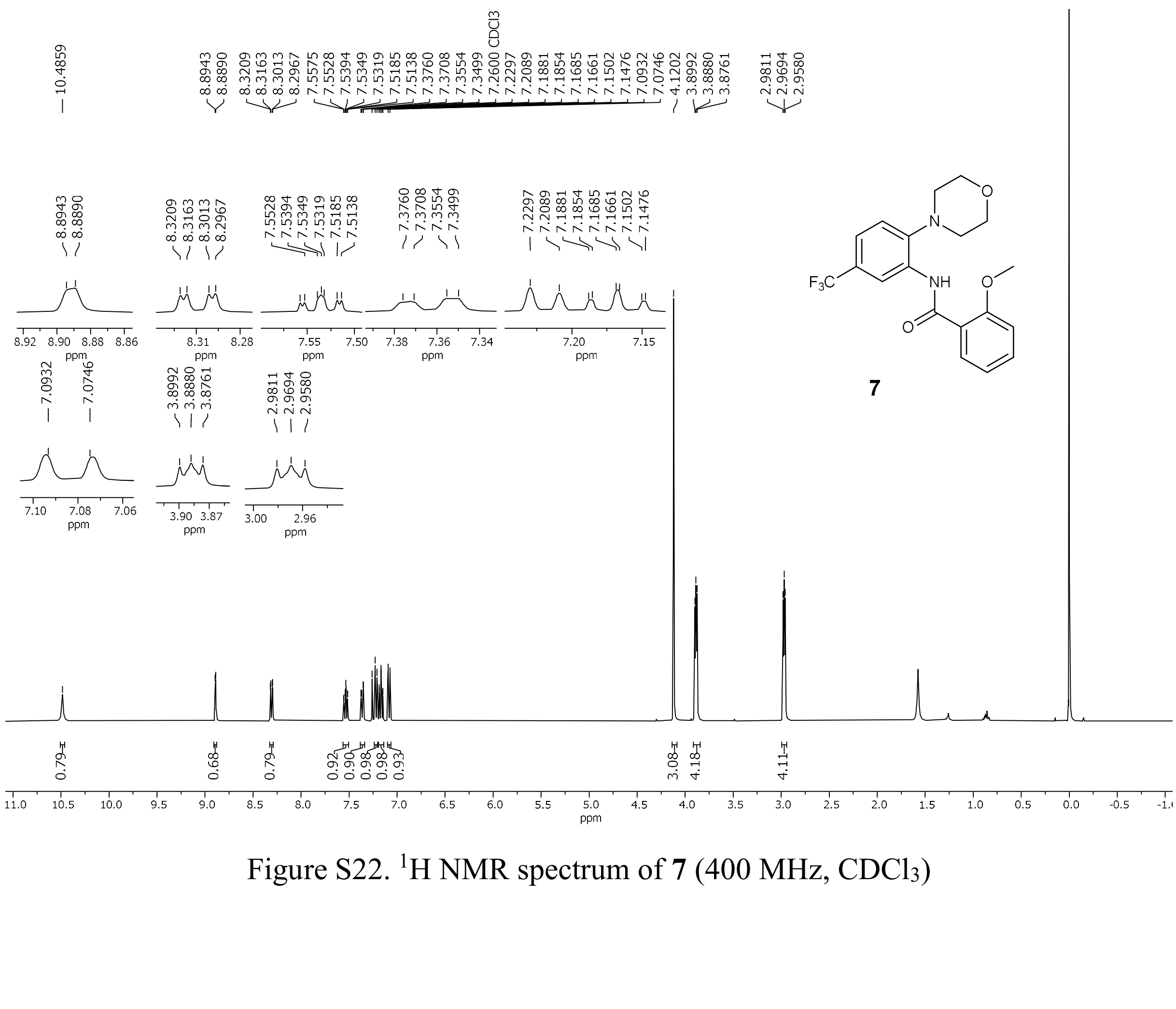

Figure S22. ^{1}H NMR spectrum of **7** (400 MHz, $CDCl_3$)

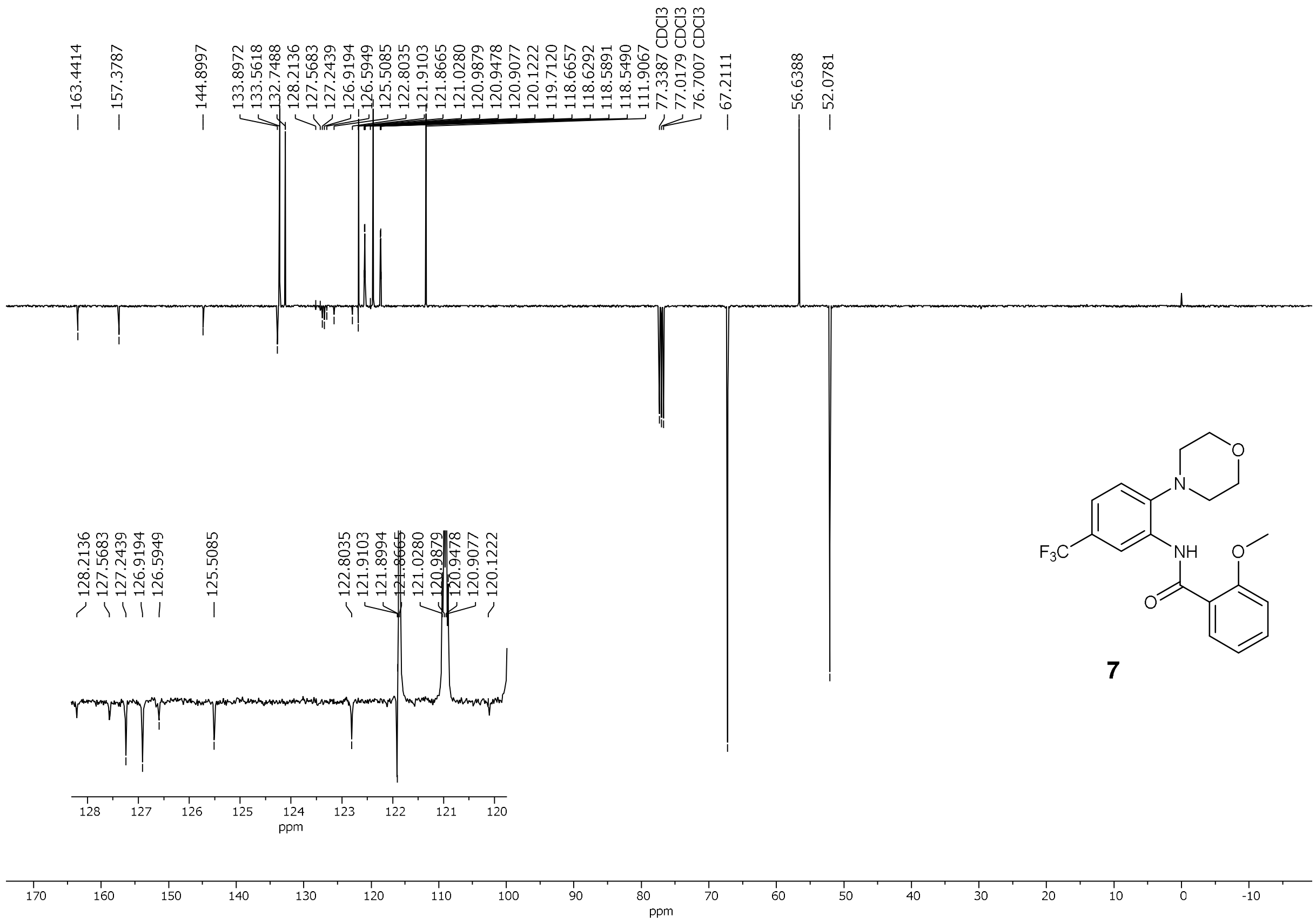

Figure S23. ^{13}C APT NMR spectrum of **7** (101 MHz, CDCl$_3$)

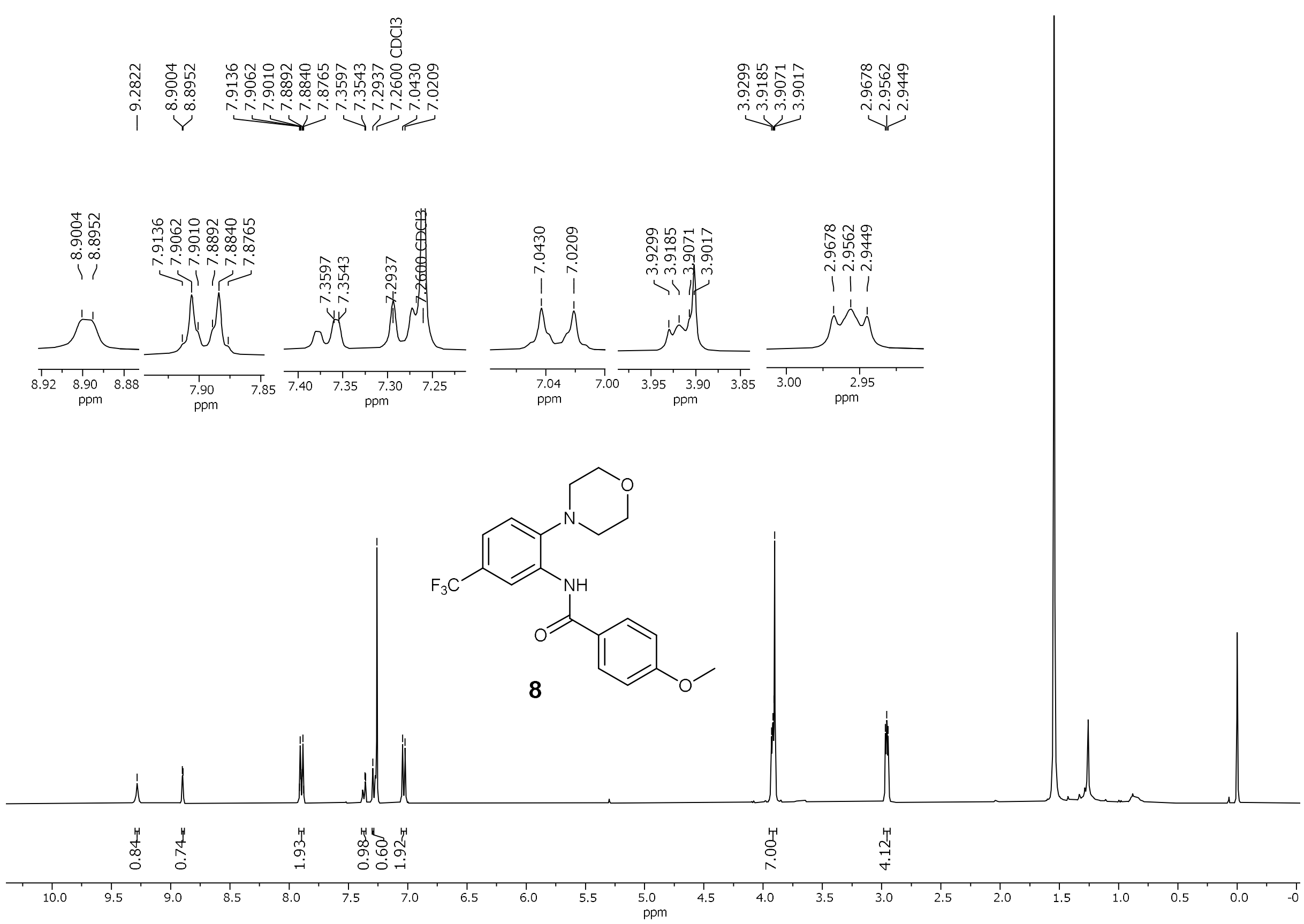

Figure S24. ^{1}H NMR spectrum of **8** (400 MHz, CDCl$_3$)

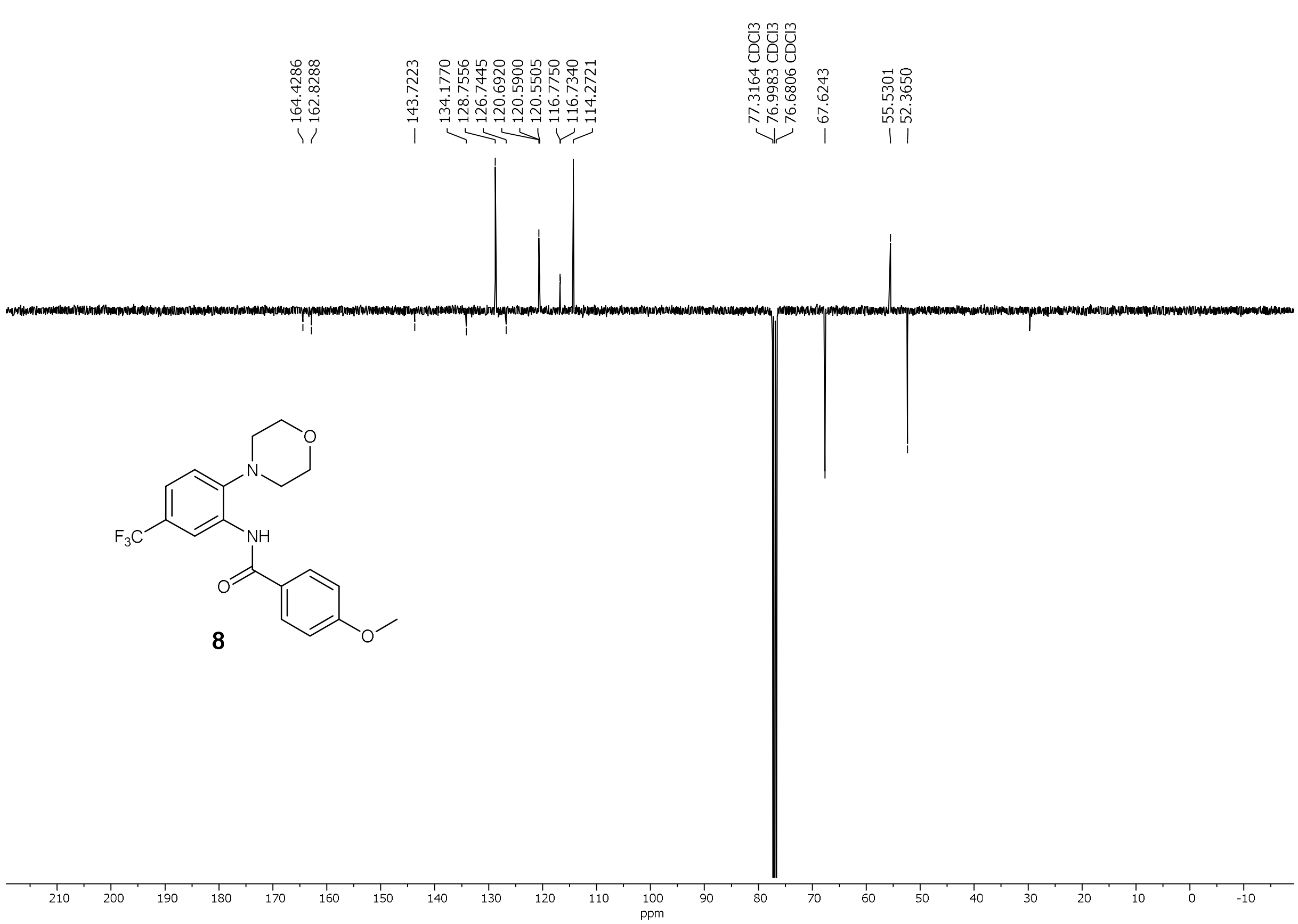

Figure S25. ^{13}C APT NMR spectrum of **8** (101 MHz, $CDCl_3$)

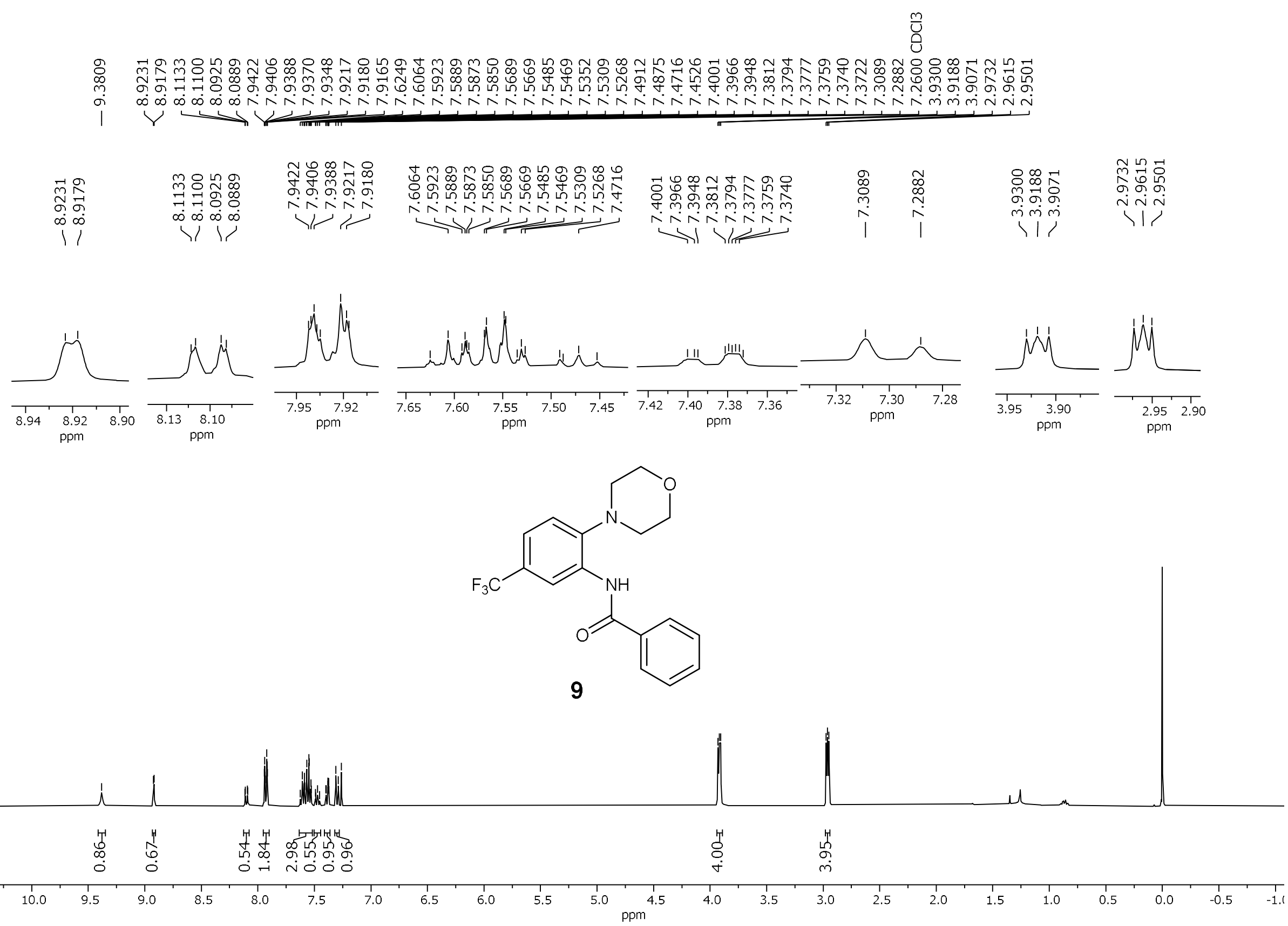

Figure S26. ^{1}H NMR spectrum of **9** (400 MHz, $CDCl_3$)

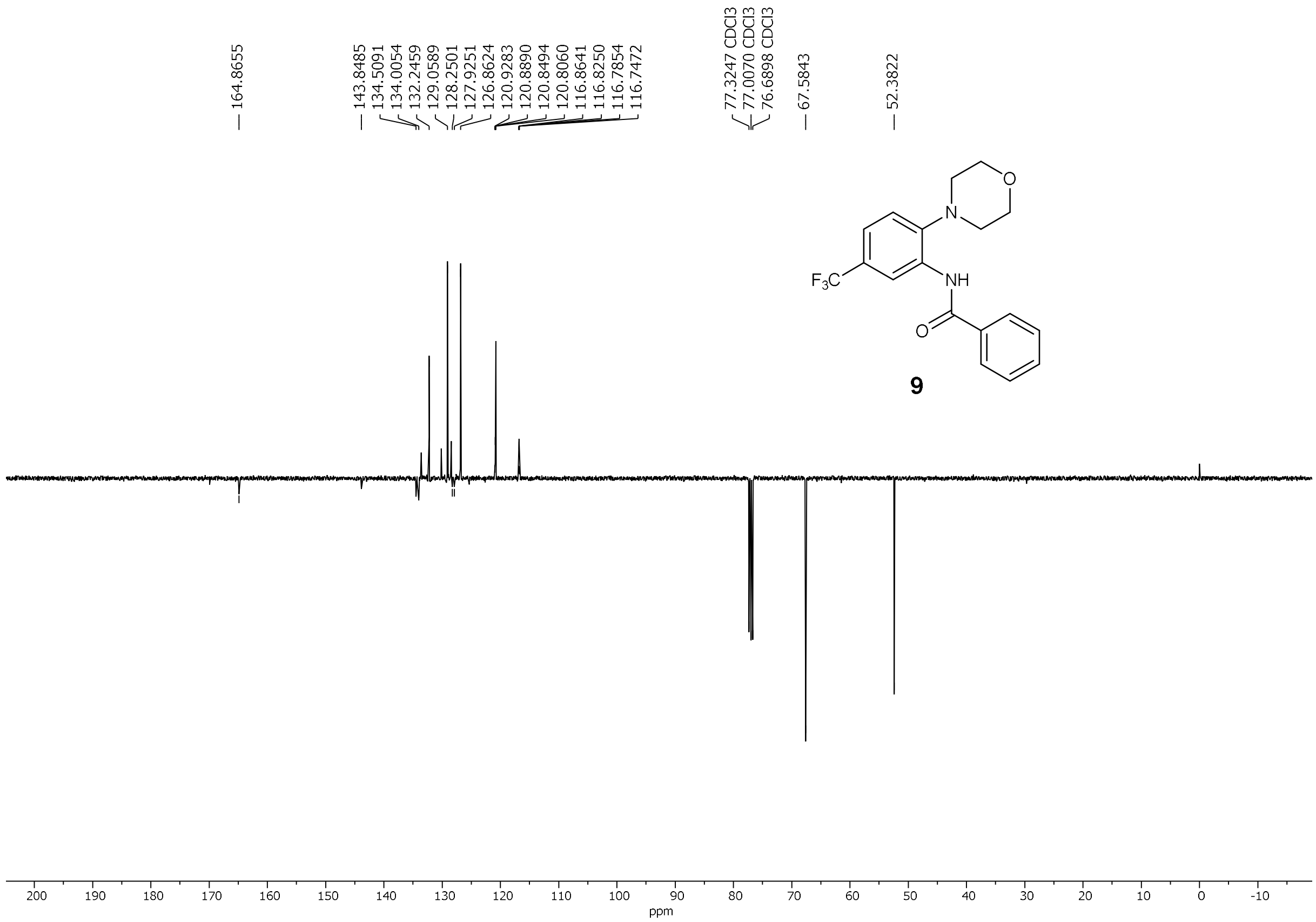

Figure S27. ^{13}C APT NMR spectrum of **9** (101 MHz, $CDCl_3$)

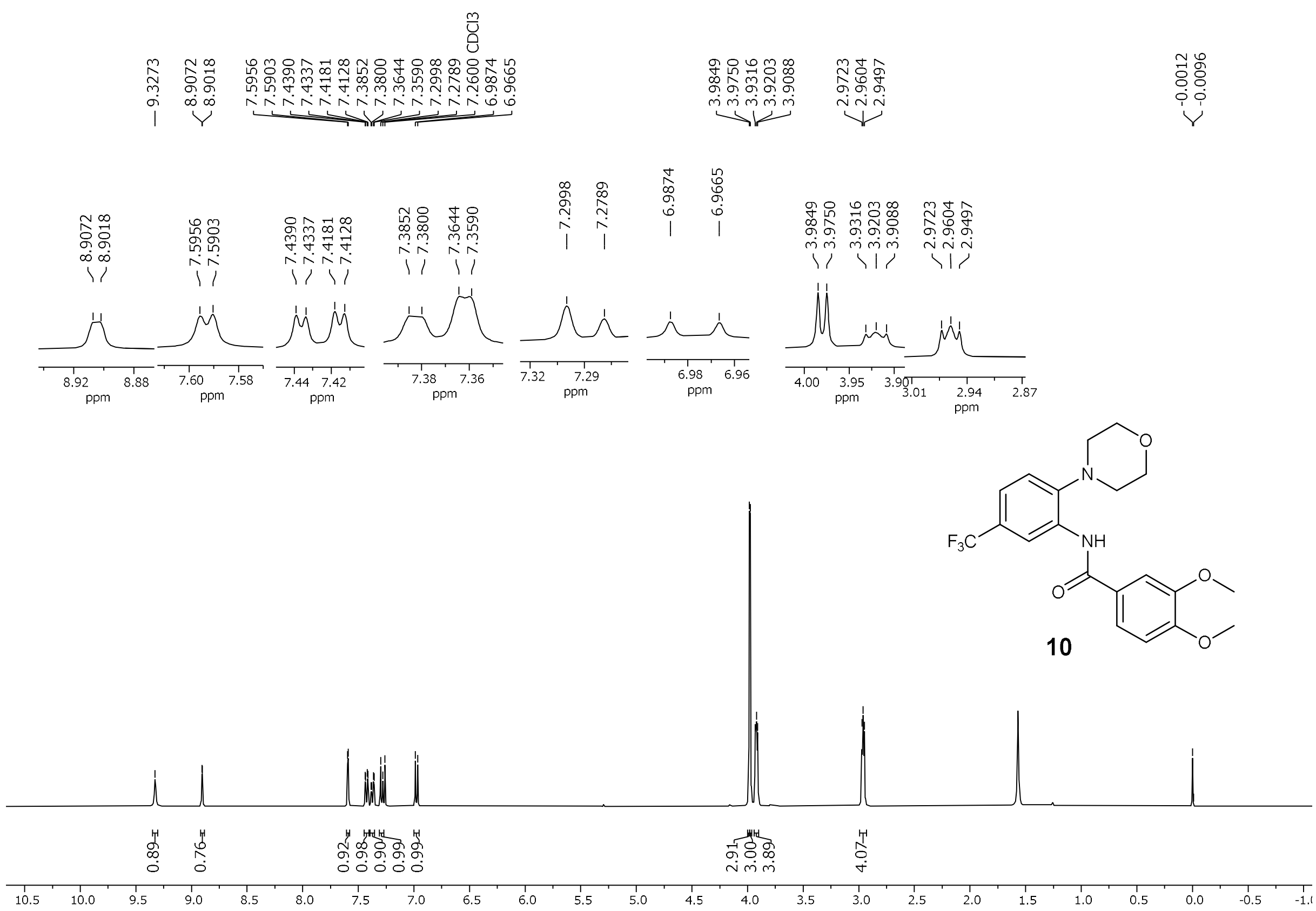

Figure S28. ^{1}H NMR spectrum of **10** (400 MHz, $CDCl_3$)

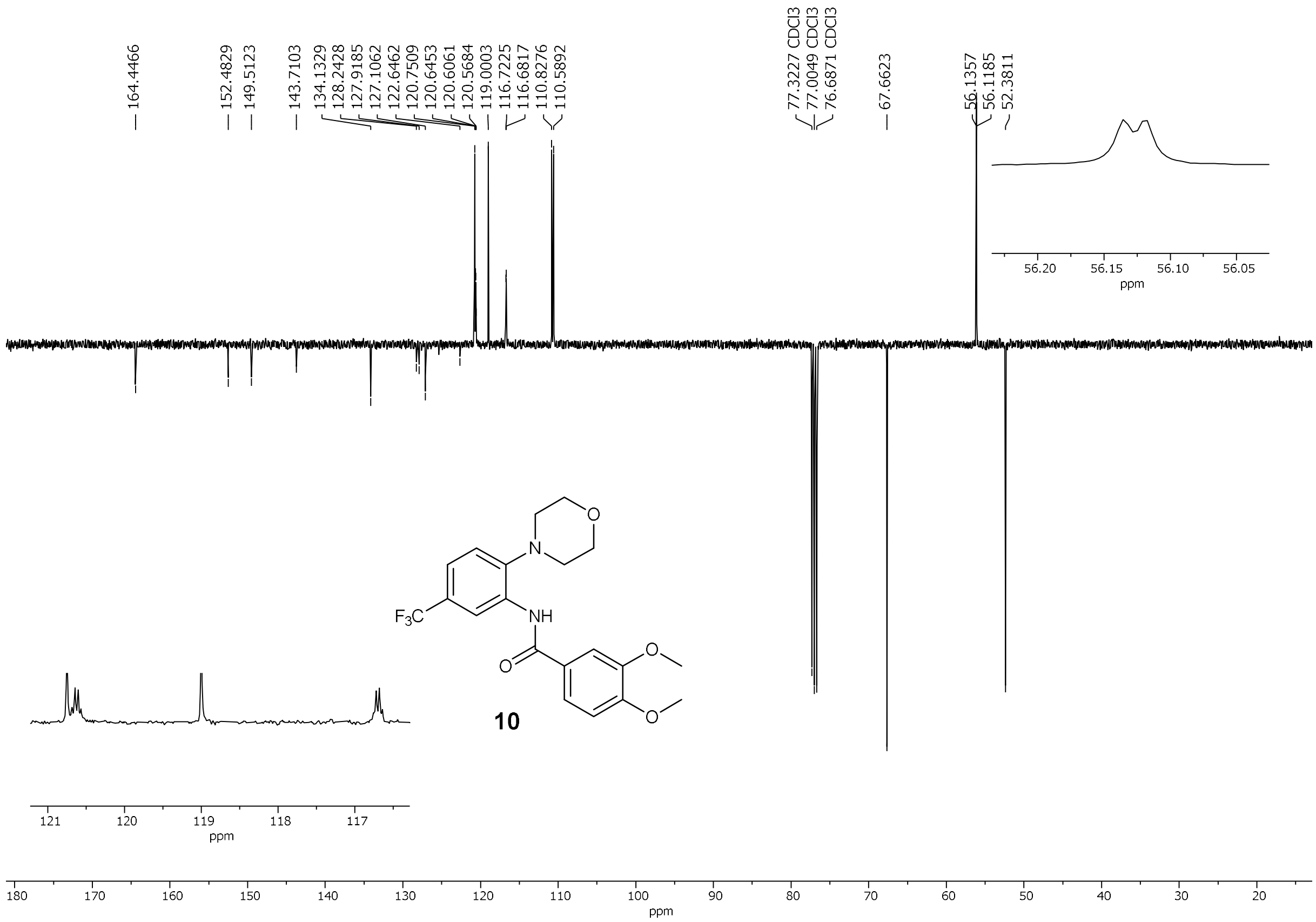

Figure S29. [11]C APT NMR spectrum of **10** (101 MHz, $CDCl_3$)

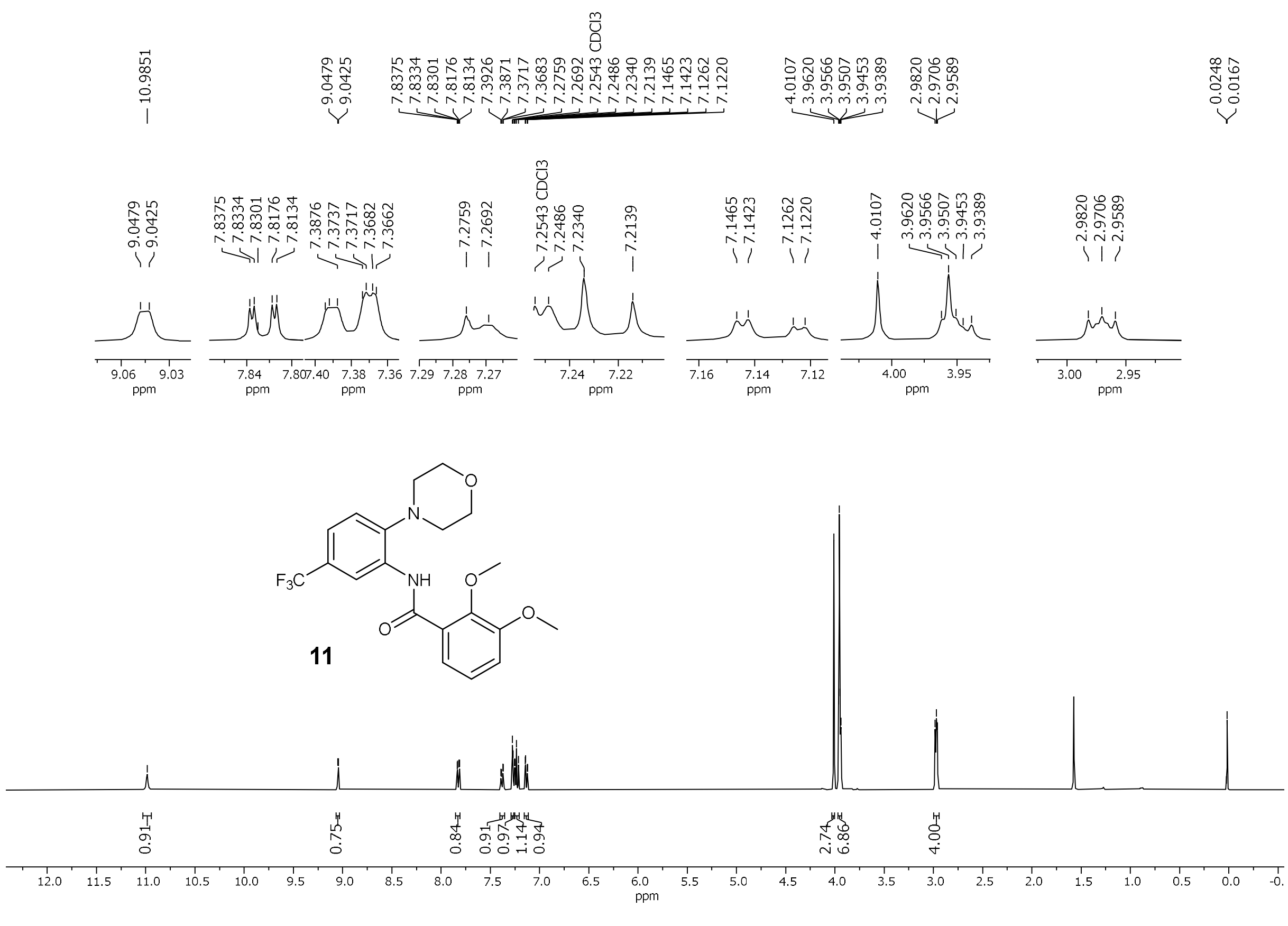

Figure S30. ^{1}H NMR spectrum of **11** (400 MHz, $CDCl_3$)

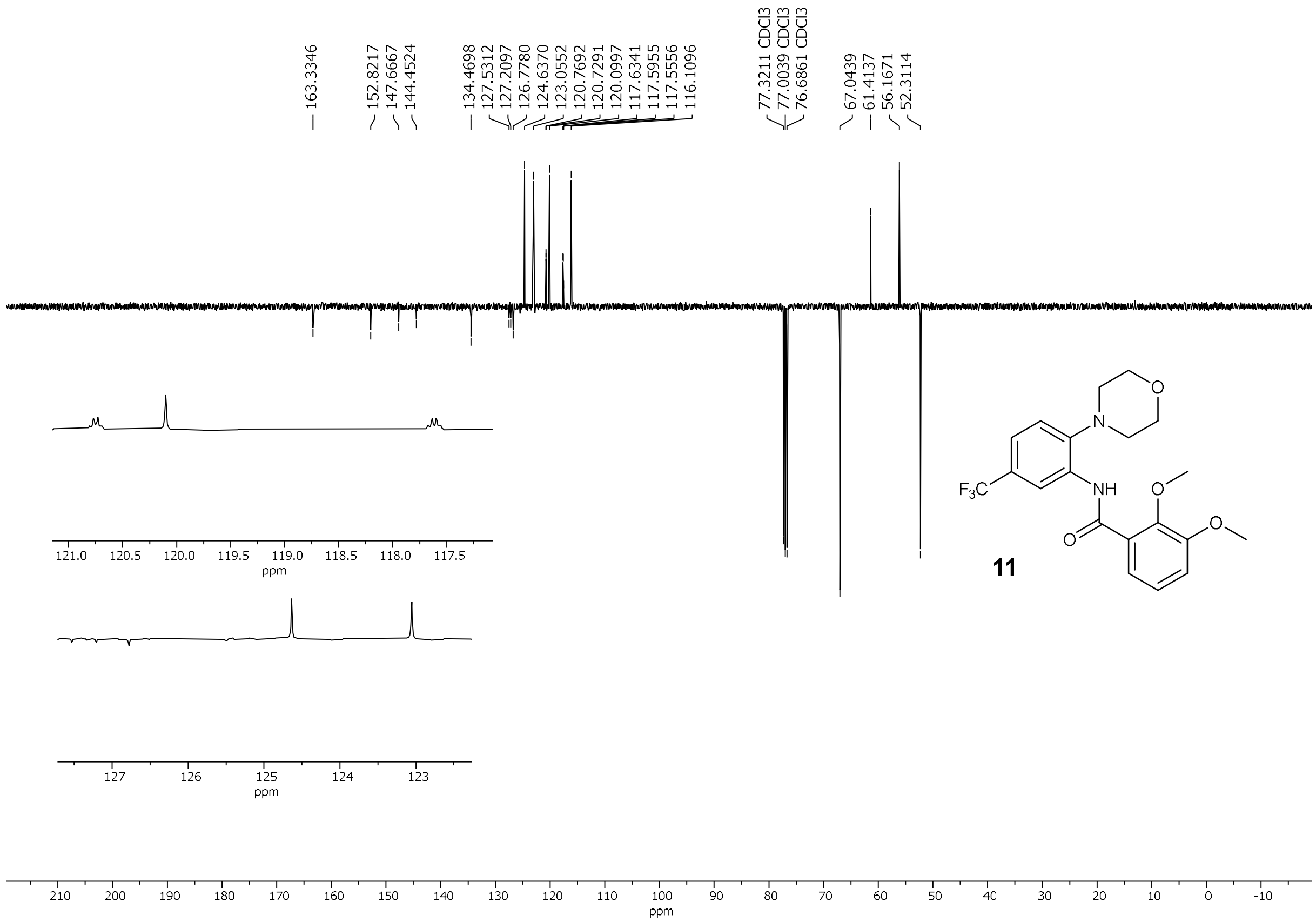

Figure S31. ^{13}C APT NMR spectrum of **11** (101 MHz, $CDCl_3$)

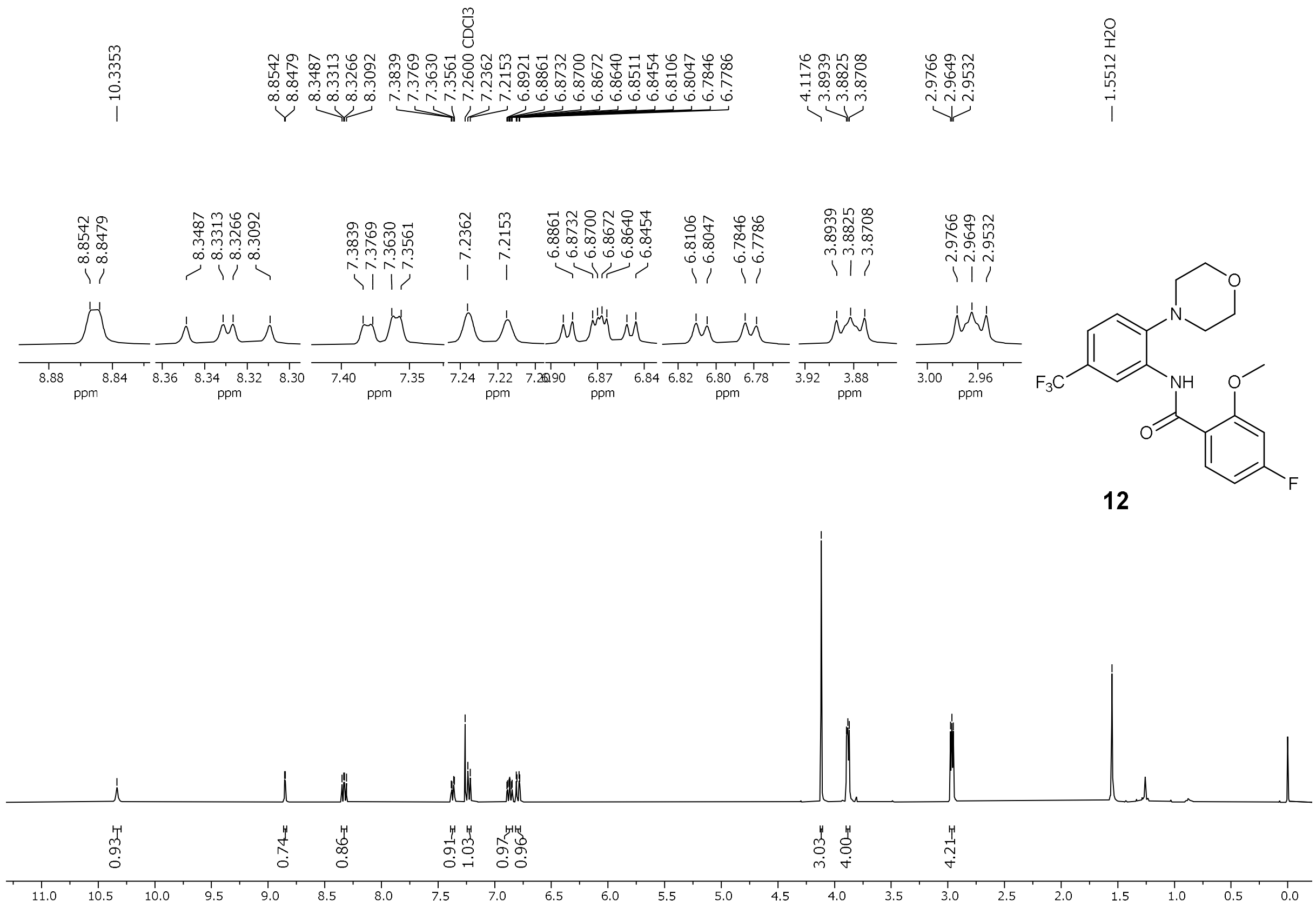

Figure S32. ^{1}H NMR spectrum of **12** (400 MHz, $CDCl_3$)

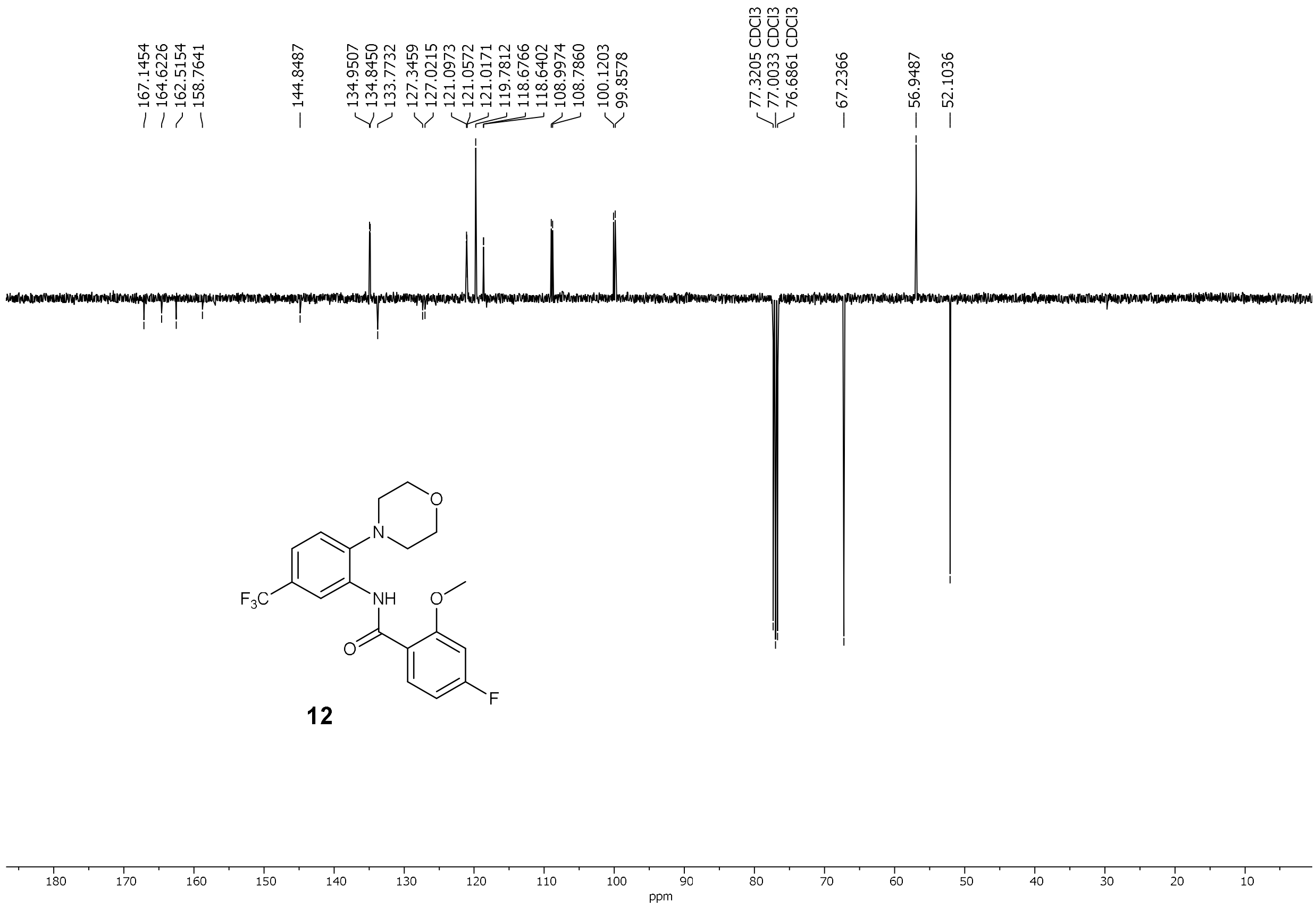

Figure S33. ^{13}C APT NMR spectrum of **12** (101 MHz, $CDCl_3$)

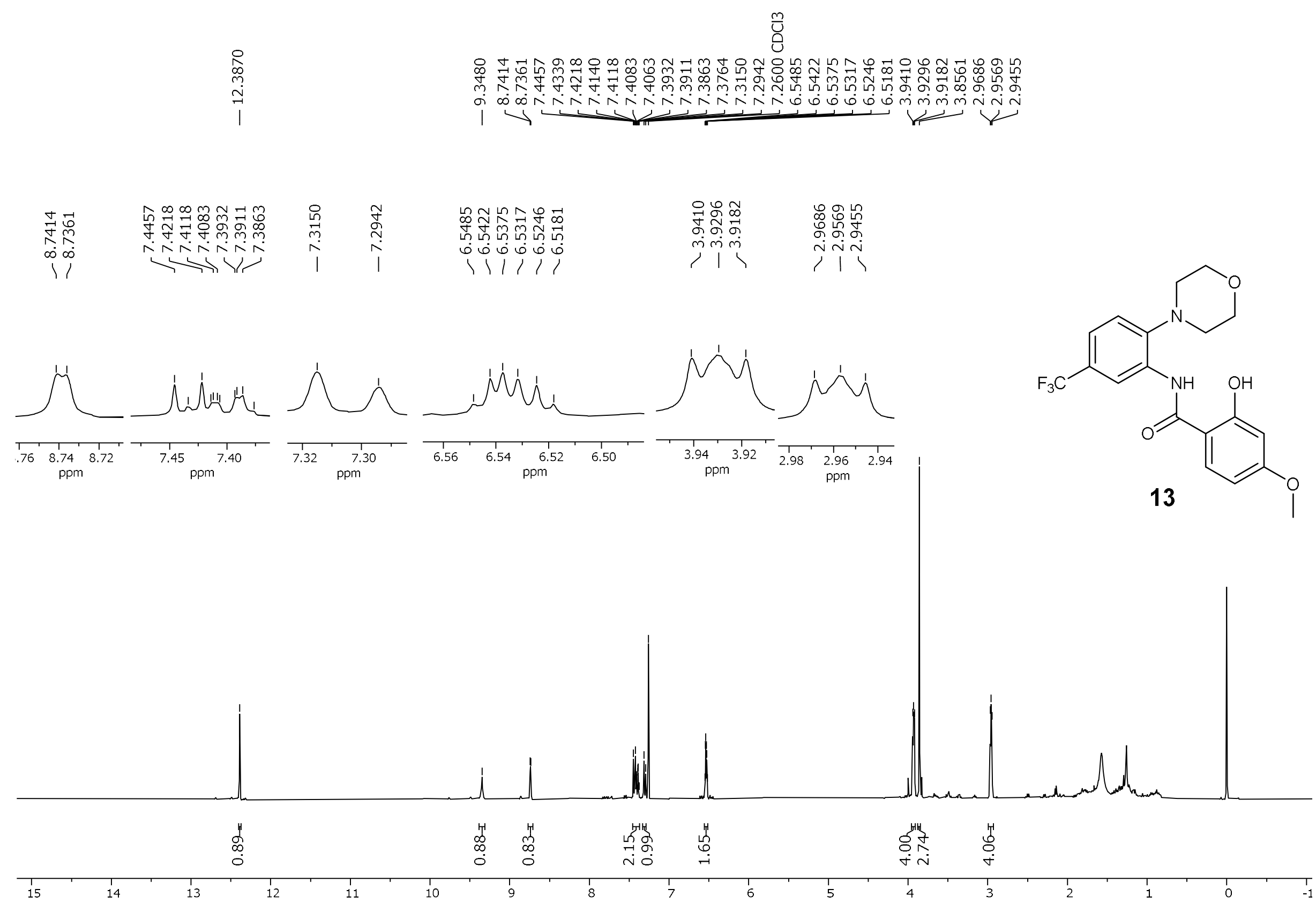

Figure S34. ^{1}H NMR spectrum of **13** (400 MHz, $CDCl_3$)

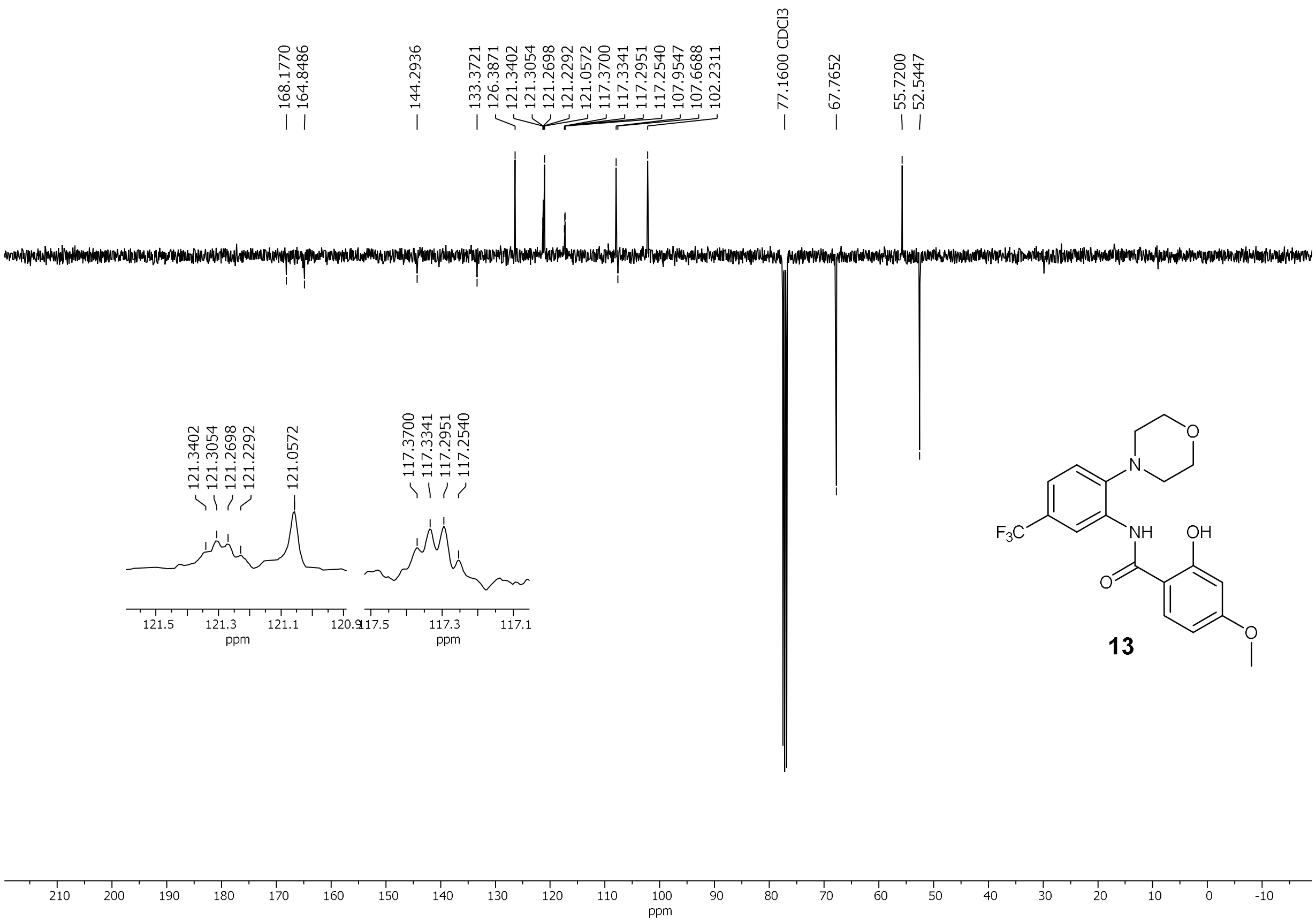

Figure S35. ^{13}C APT NMR spectrum of **13** (101 MHz, $CDCl_3$)

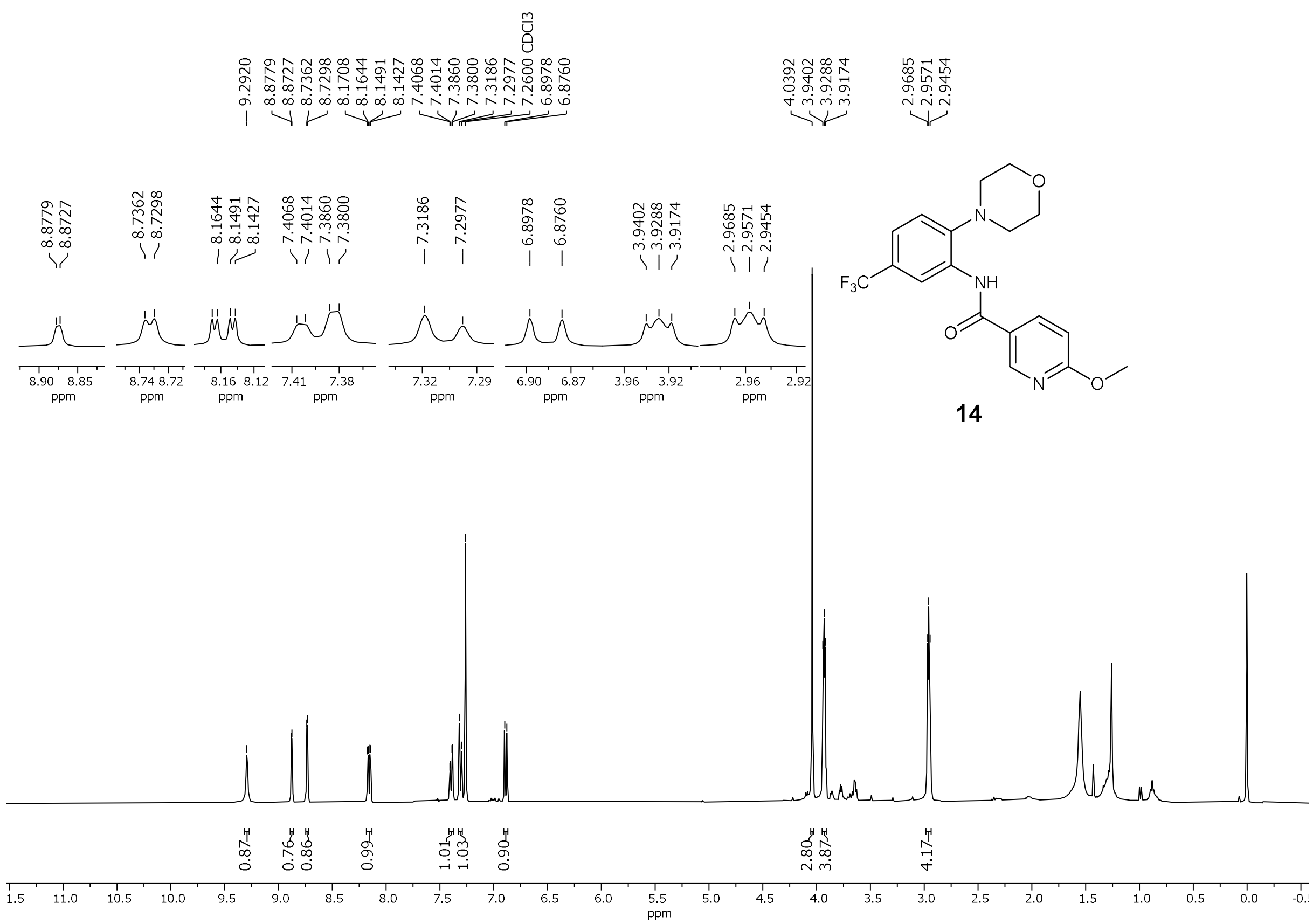

Figure S36. ^{1}H NMR spectrum of **14** (400 MHz, $CDCl_3$)

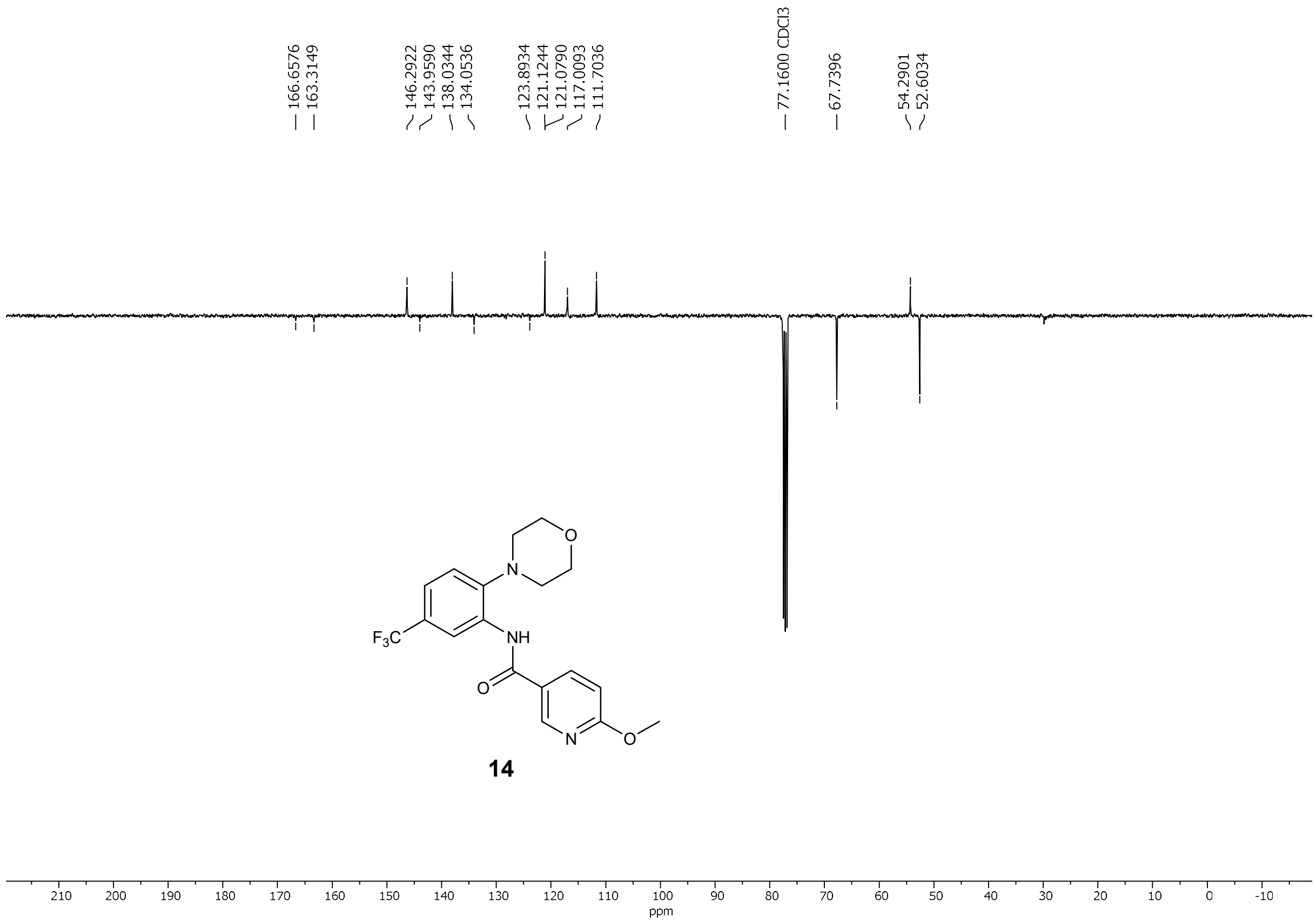

Figure S37. ^{13}C APT NMR spectrum of **14** (101 MHz, $CDCl_3$)

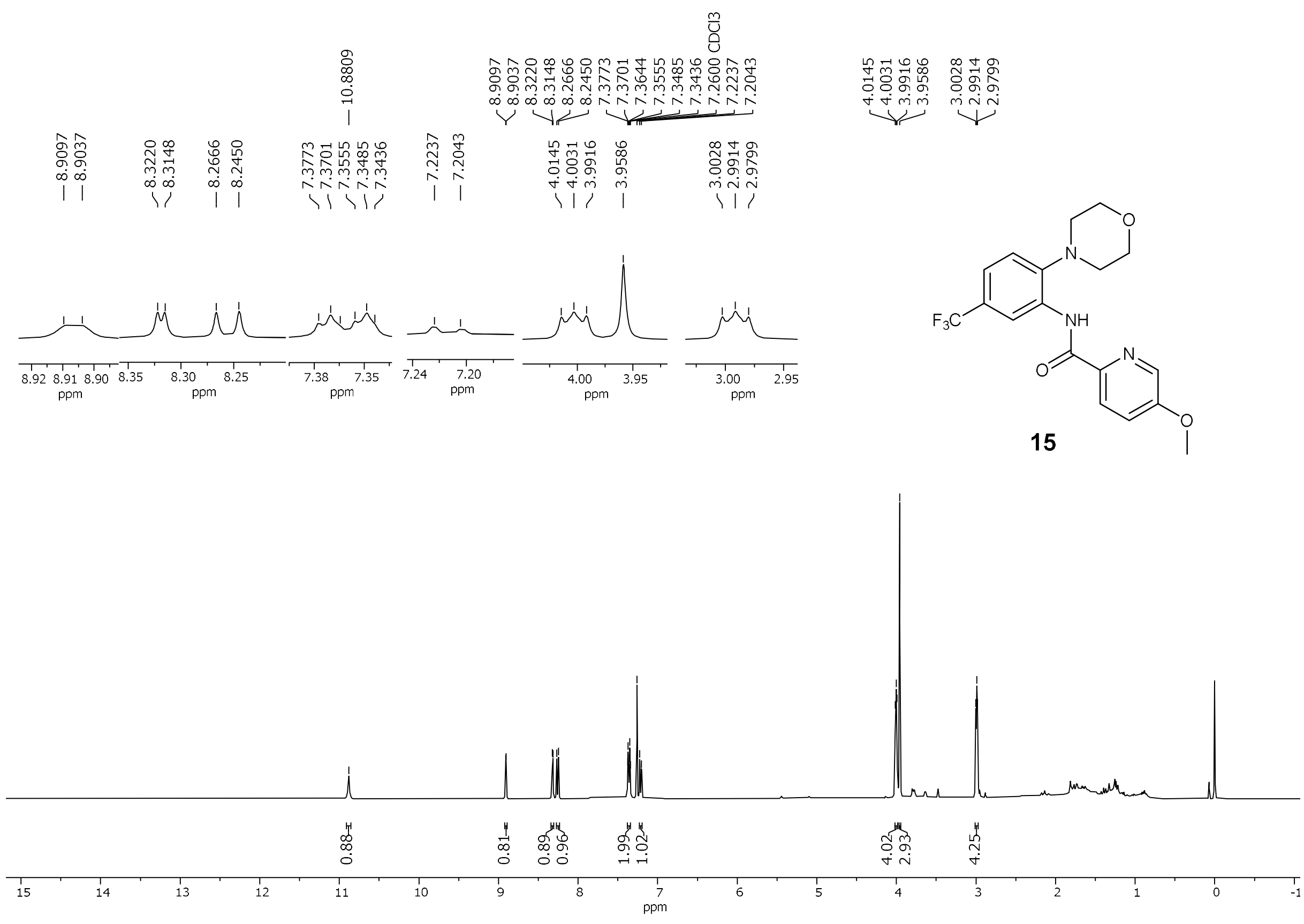

Figure S38. ^{1}H NMR spectrum of **15** (400 MHz, CDCl3)

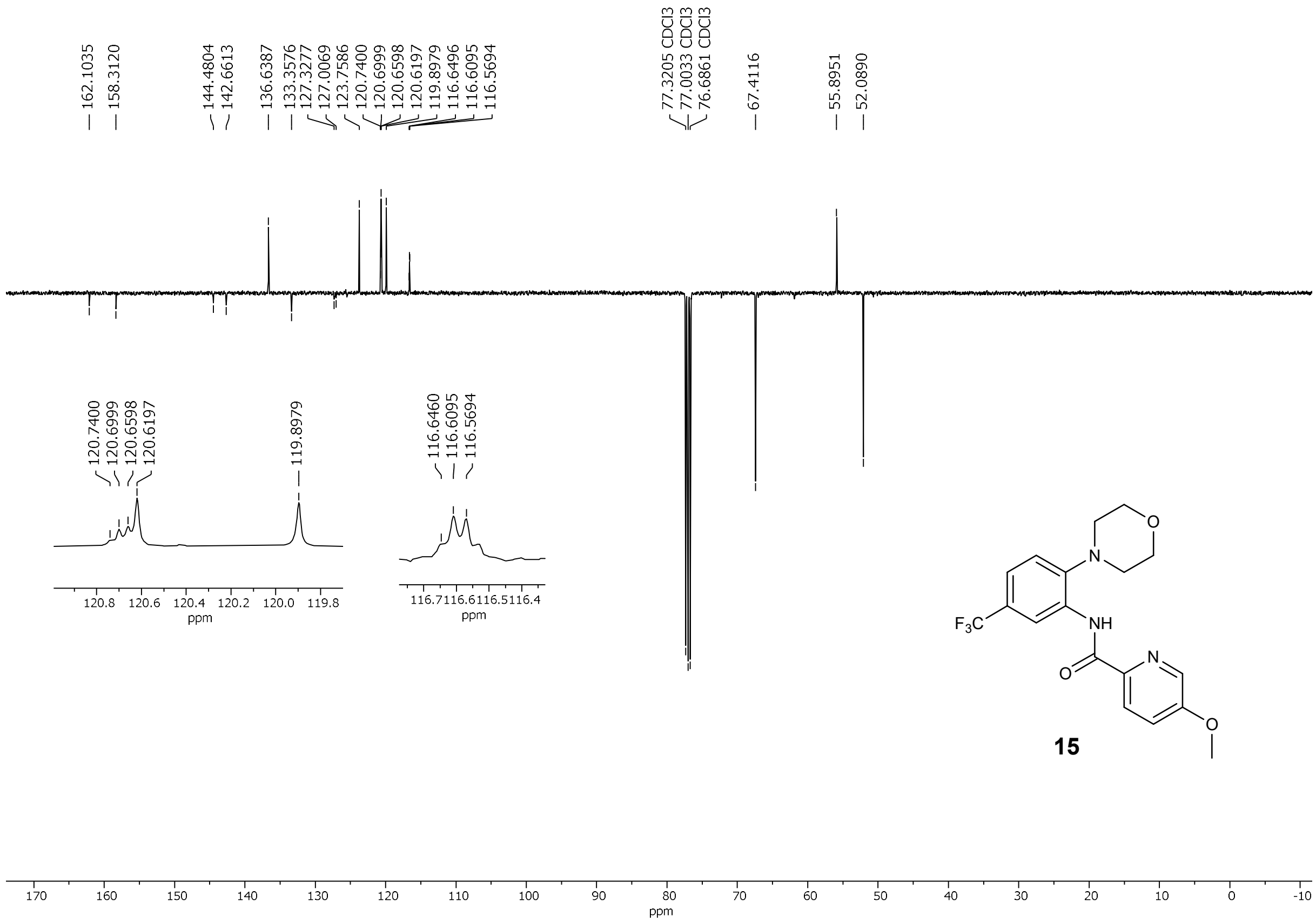

Figure S39. ^{13}C APT NMR spectrum of **15** (101 MHz, $CDCl_3$)

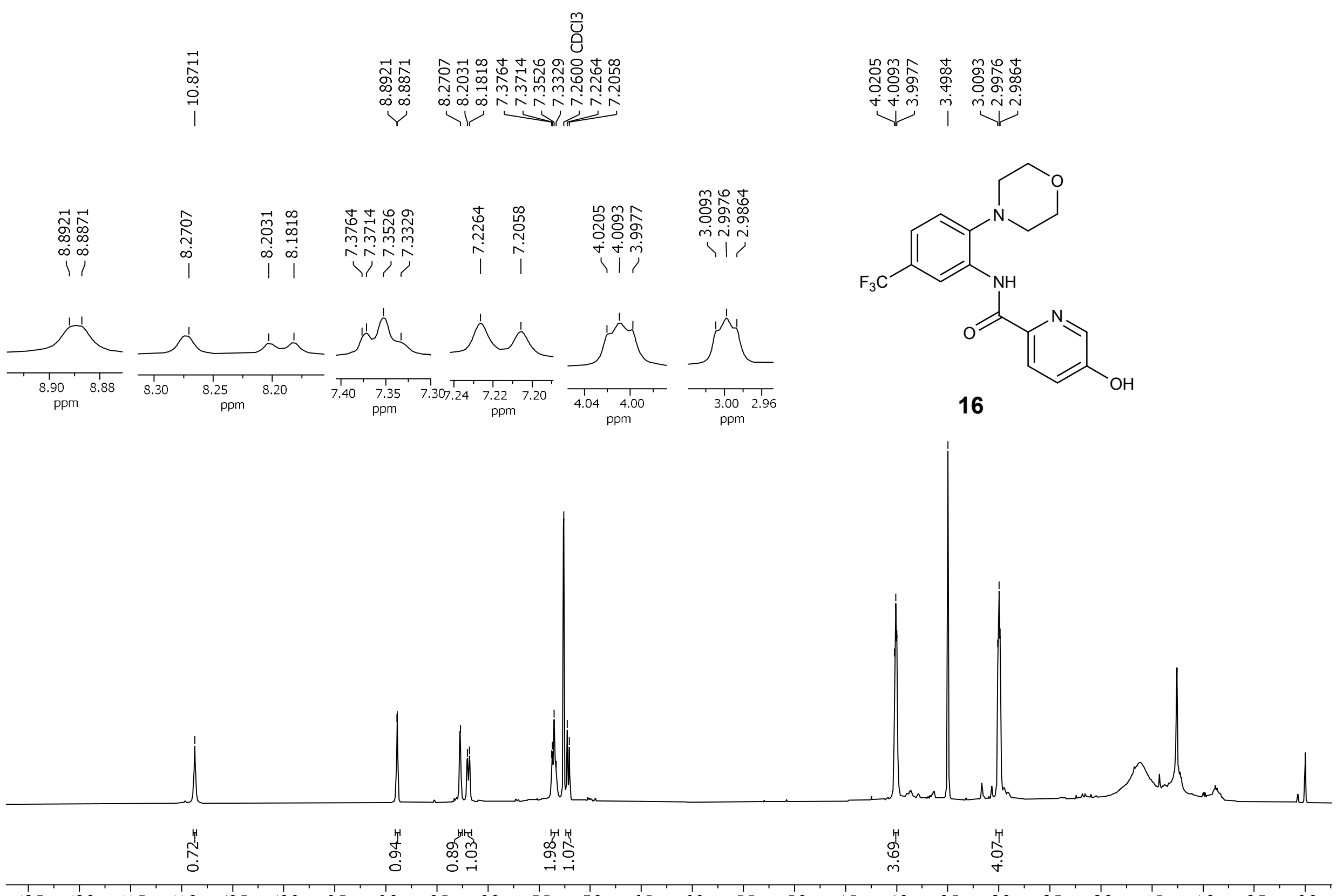

Figure S40. ^{1}H NMR spectrum of **16** (400 MHz, $CDCl_3$)

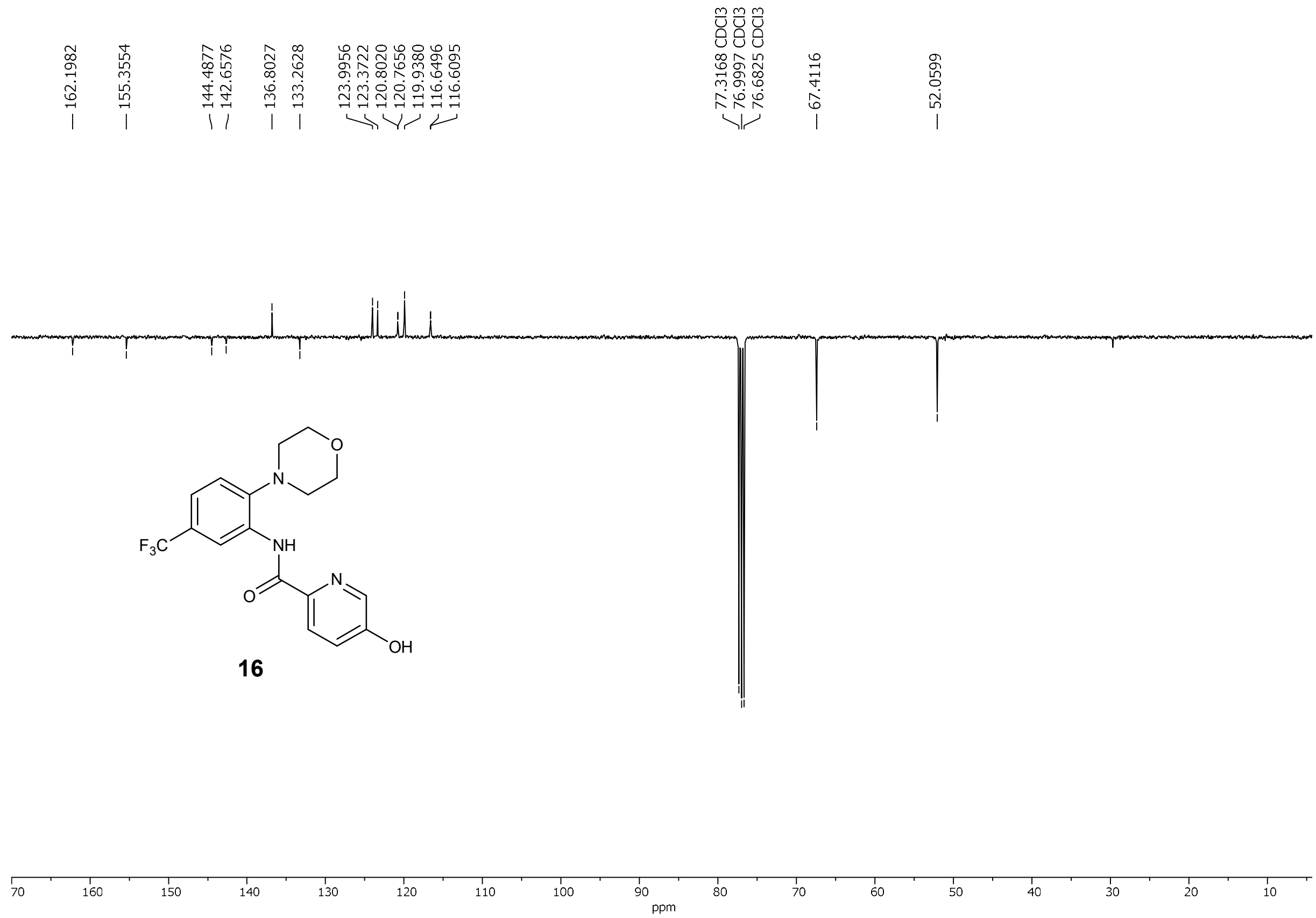

Figure S41. ^{13}C APT NMR spectrum of **16** (101 MHz, $CDCl_3$)

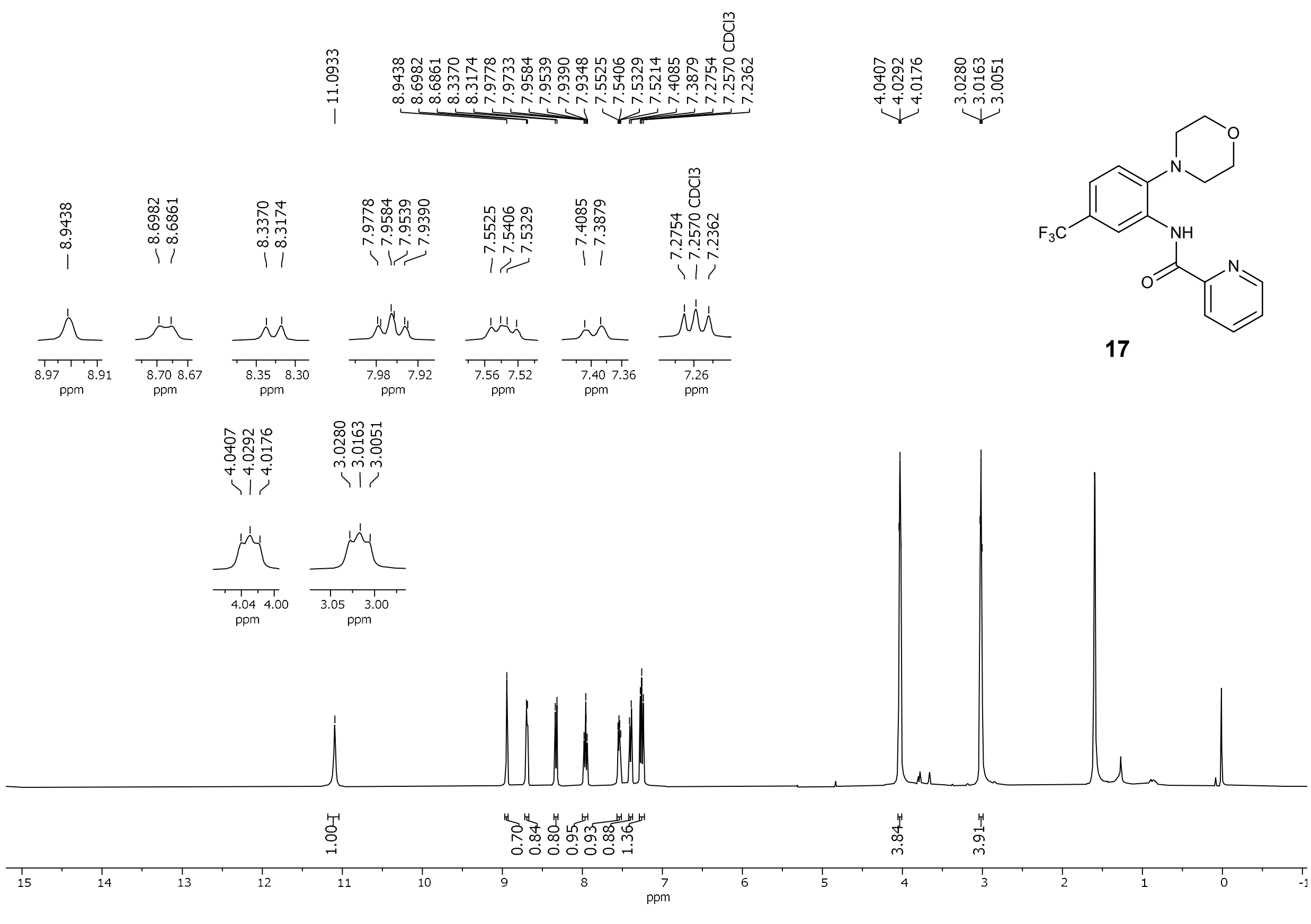

Figure S42. ^{1}H NMR spectrum of **17** (400 MHz, $CDCl_3$)

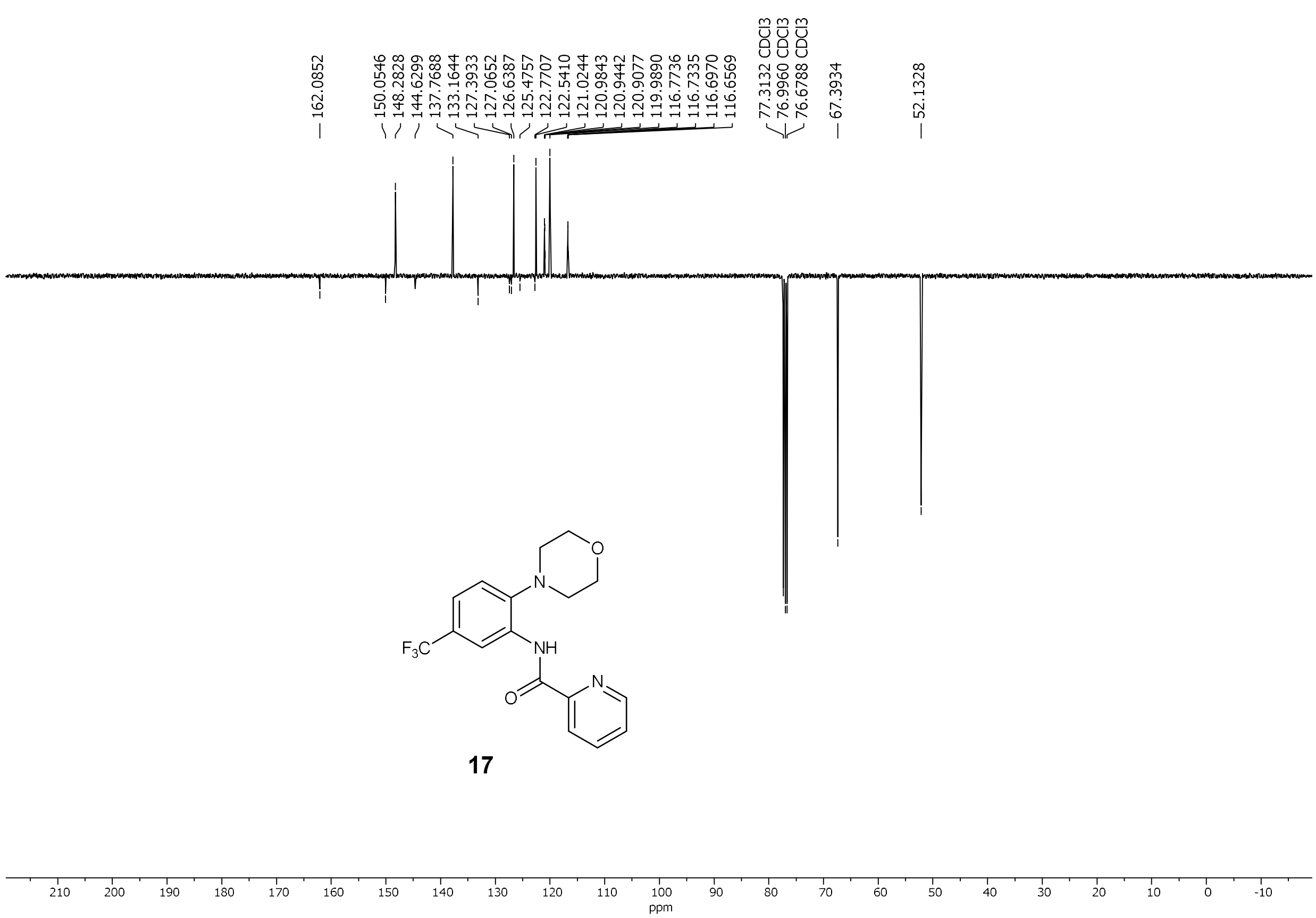

Figure S43. ^{13}C APT NMR spectrum of **17** (101 MHz, $CDCl_3$)

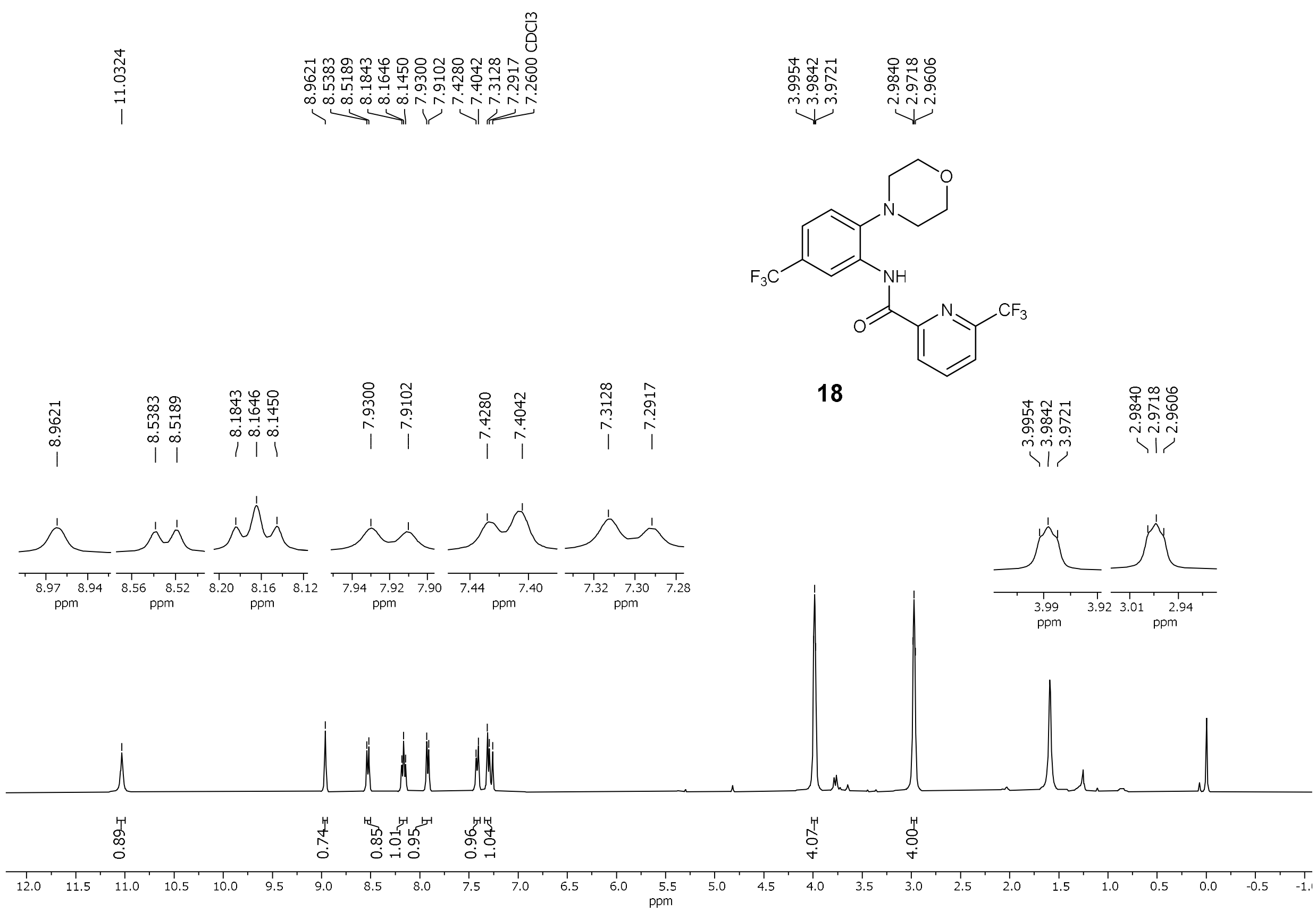

Figure S44. ^{1}H NMR spectrum of **18** (400 MHz, $CDCl_3$)

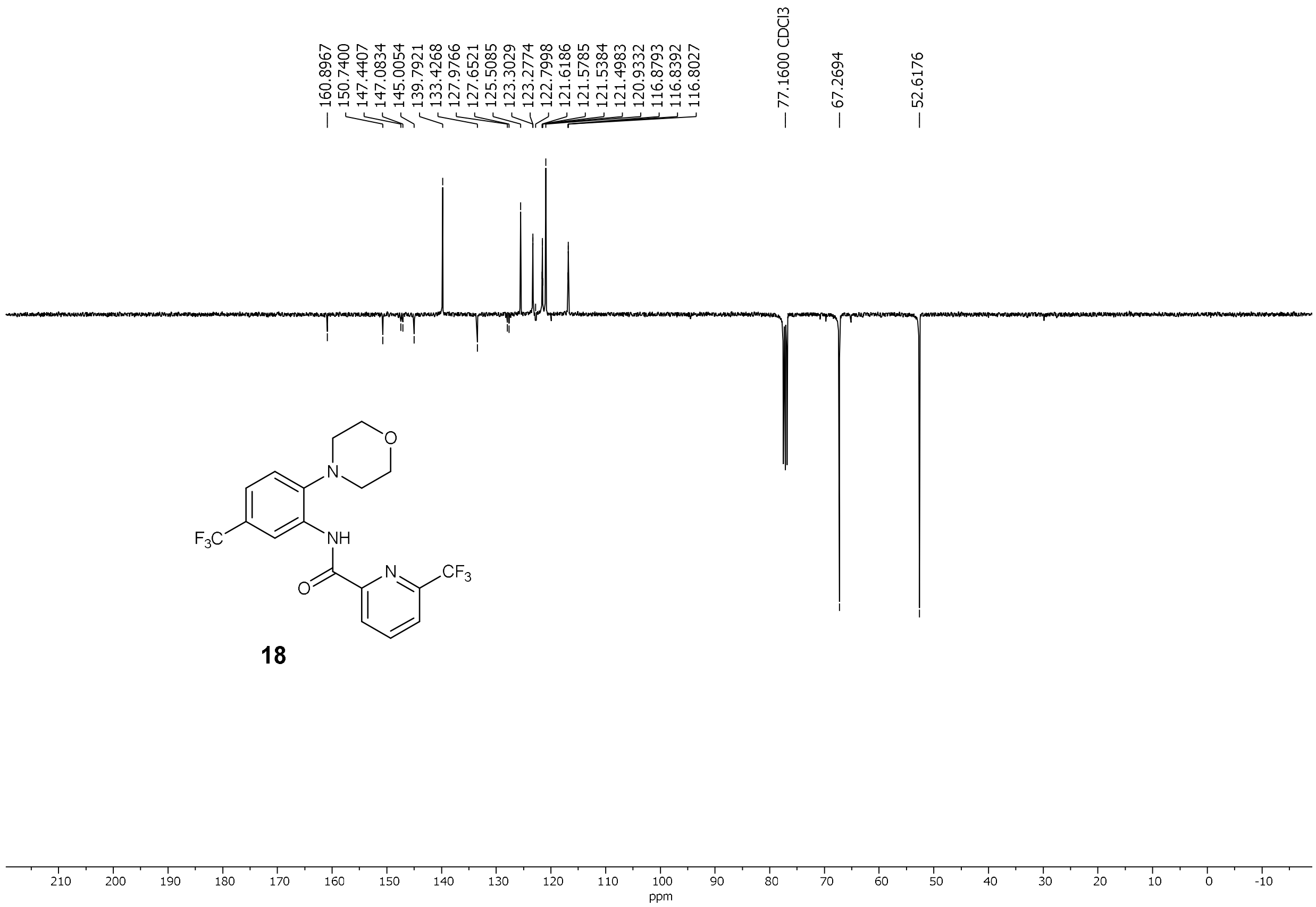

Figure S45. ^{13}C APT NMR spectrum of **18** (101 MHz, CDCl$_3$)

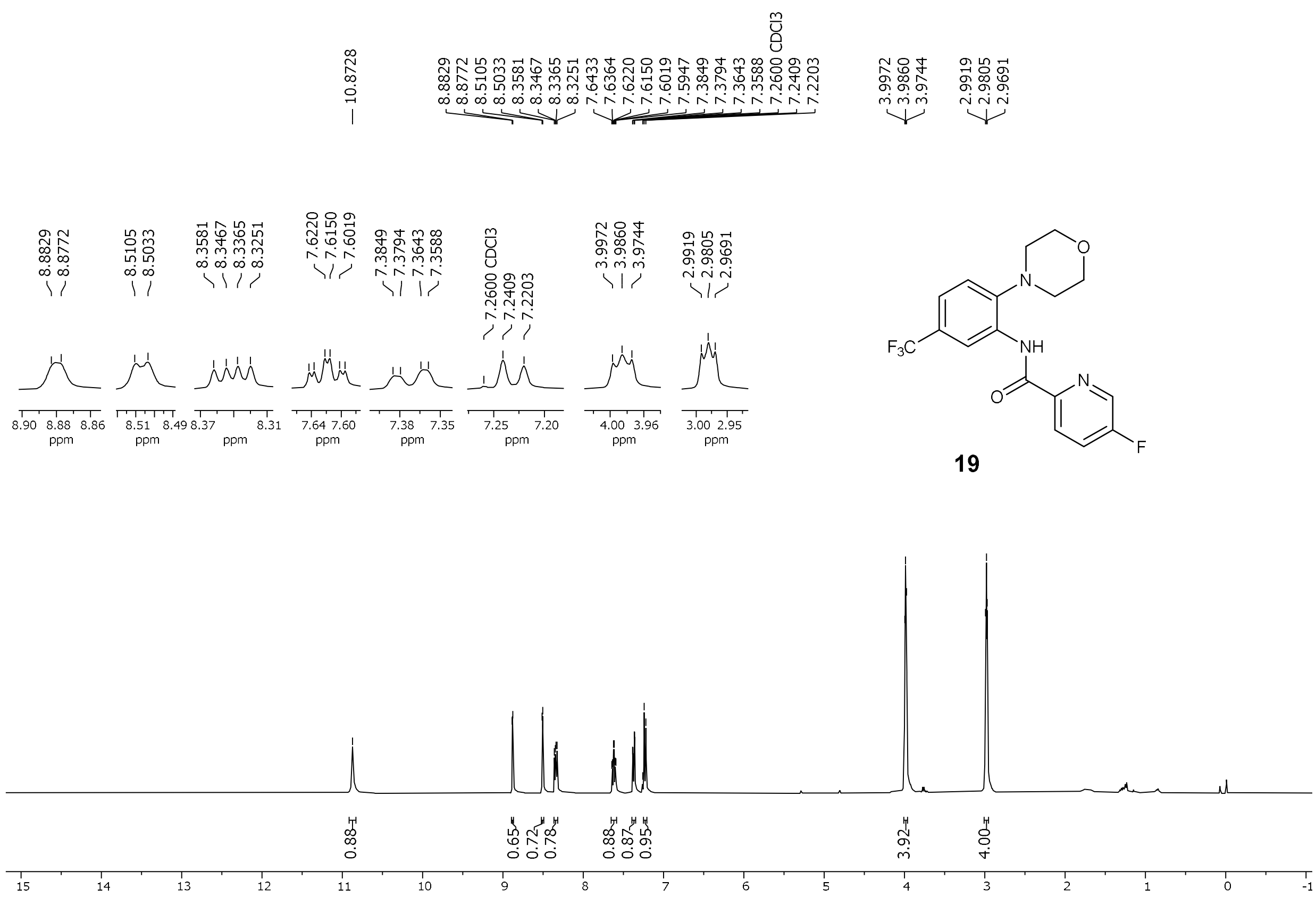

Figure S46. ^{1}H NMR spectrum of **19** (400 MHz, CDCl$_3$)

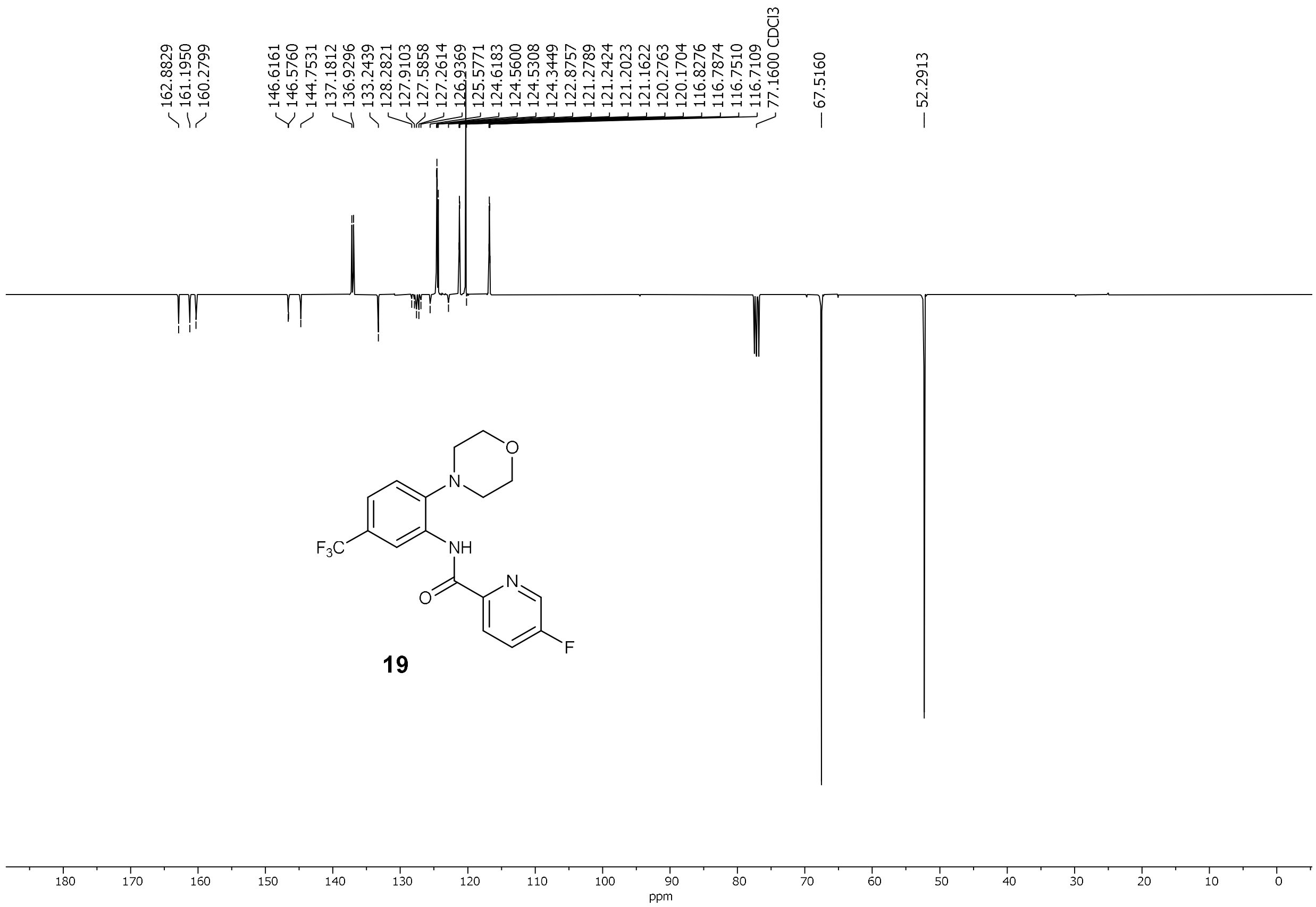

Figure S47. ^{13}C APT NMR spectrum of **19** (101 MHz, $CDCl_3$)

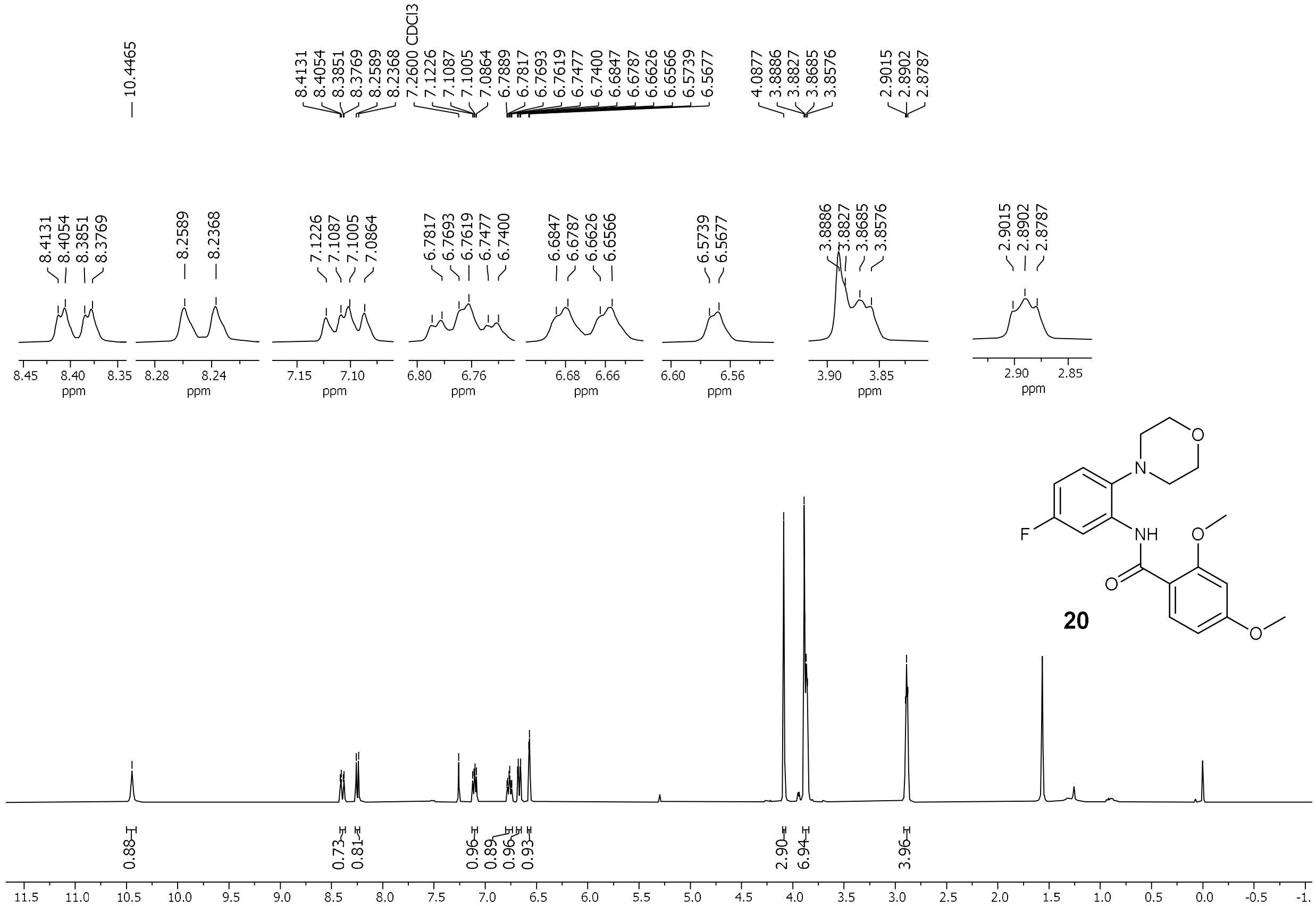

Figure S48. ^{1}H NMR spectrum of **20** (400 MHz, $CDCl_3$)

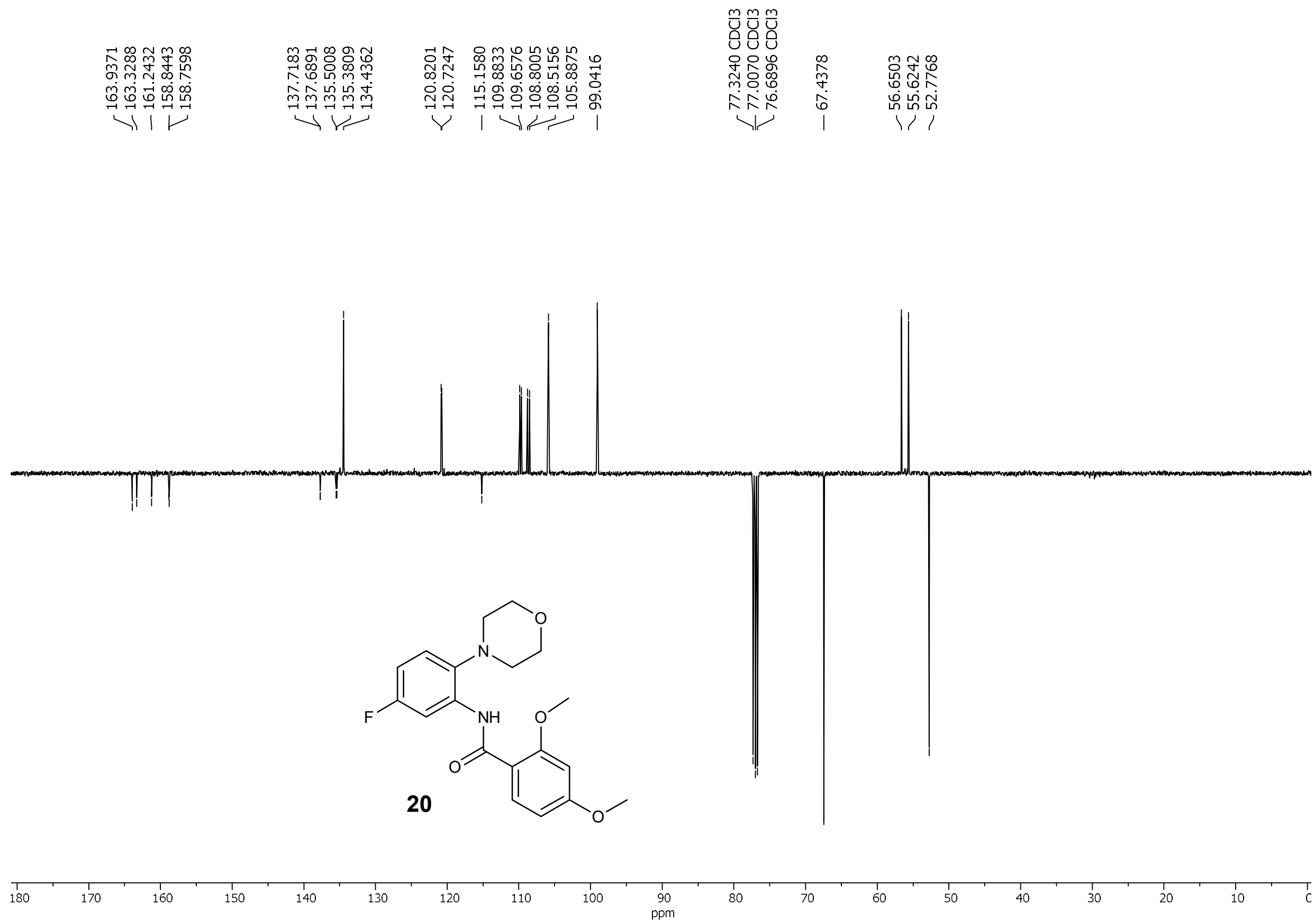

Figure S49. ^{13}C APT NMR spectrum of **20** (101 MHz, $CDCl_3$)

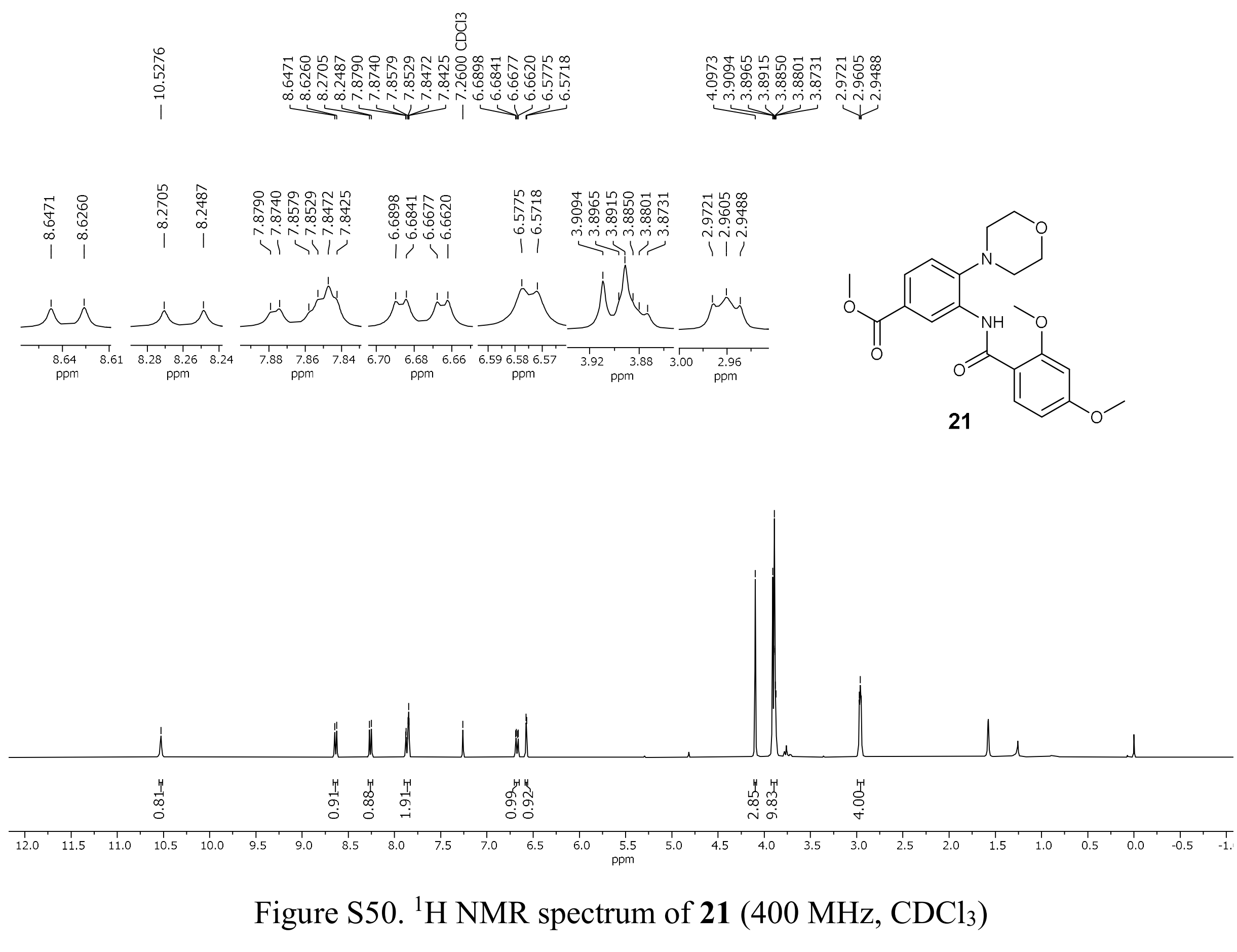

Figure S50. ^{1}H NMR spectrum of **21** (400 MHz, $CDCl_3$)

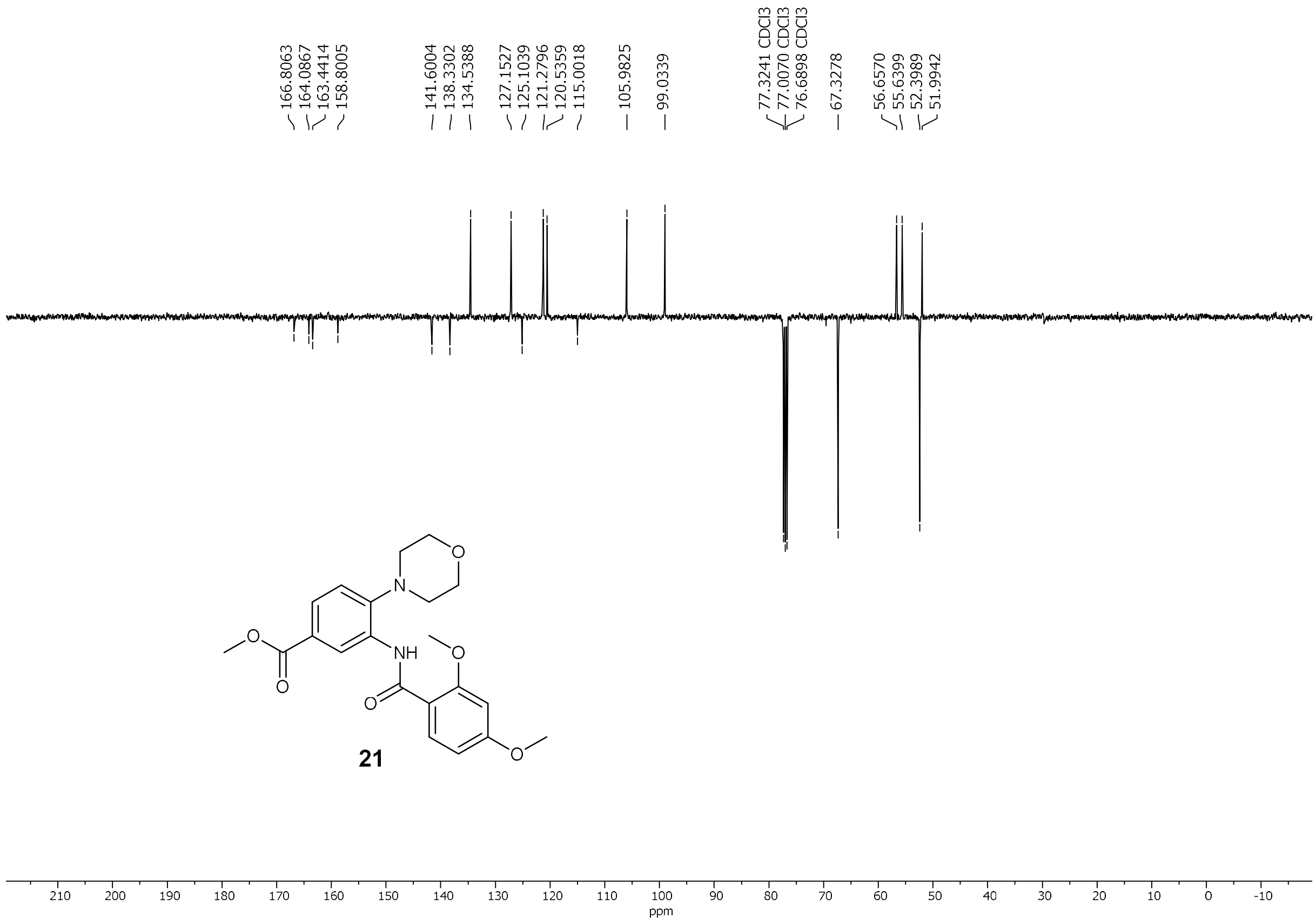

Figure S51. ^{13}C APT NMR spectrum of **21** (101 MHz, $CDCl_3$)

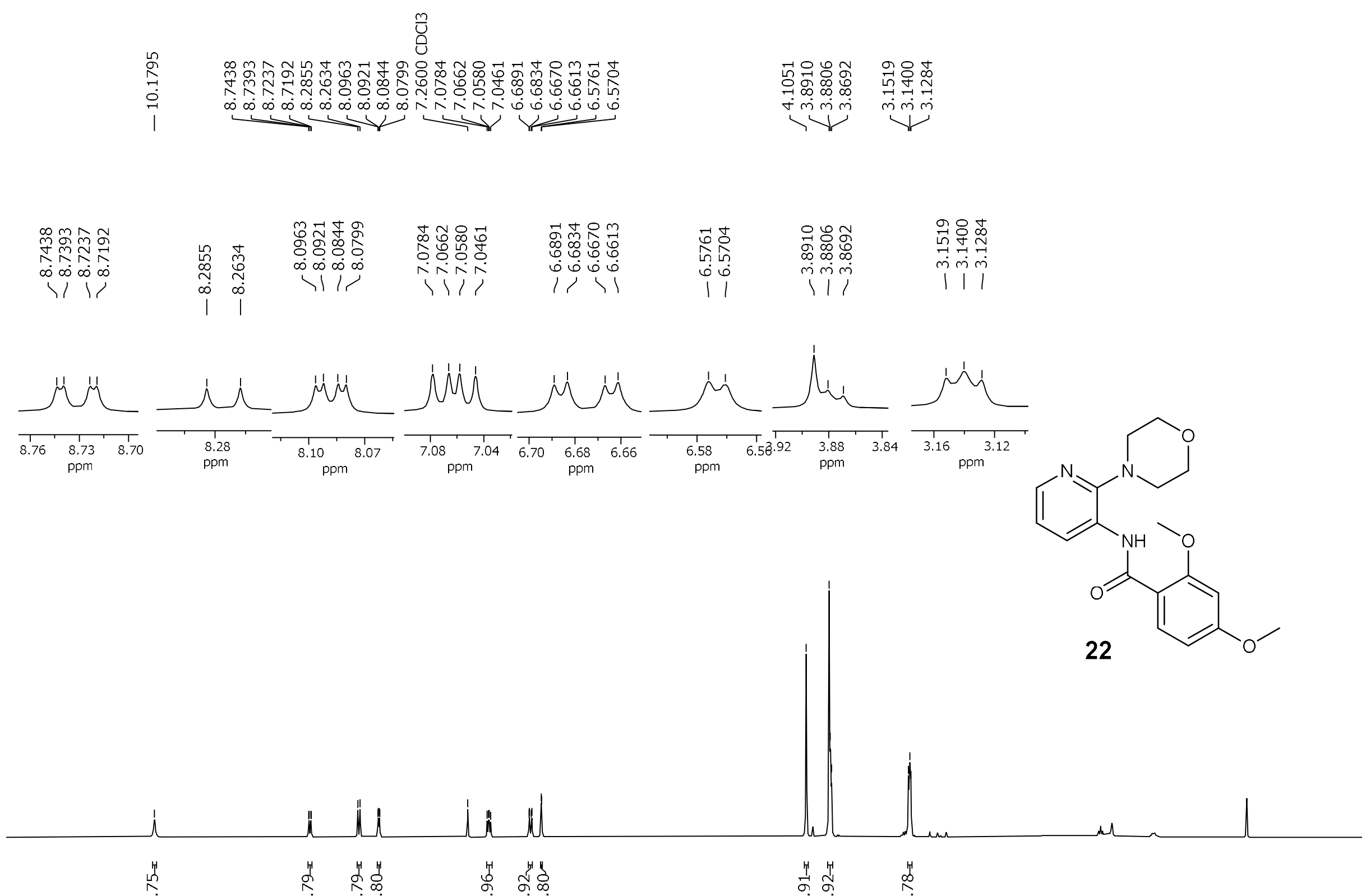

Figure S52. ^{1}H NMR spectrum of **22** (400 MHz, $CDCl_3$)

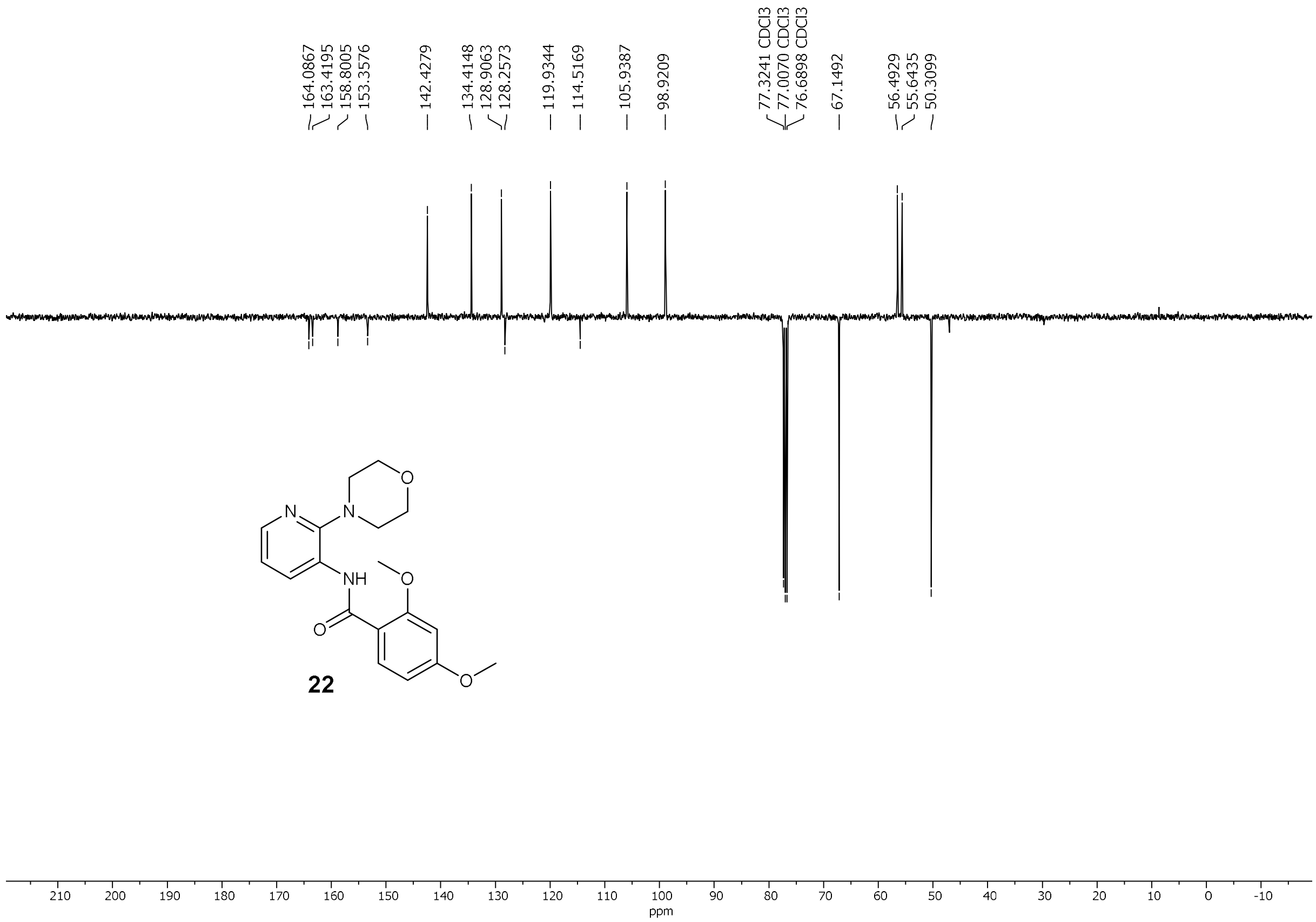

Figure S53. ^{13}C APT NMR spectrum of **22** (101 MHz, CDCl$_3$)

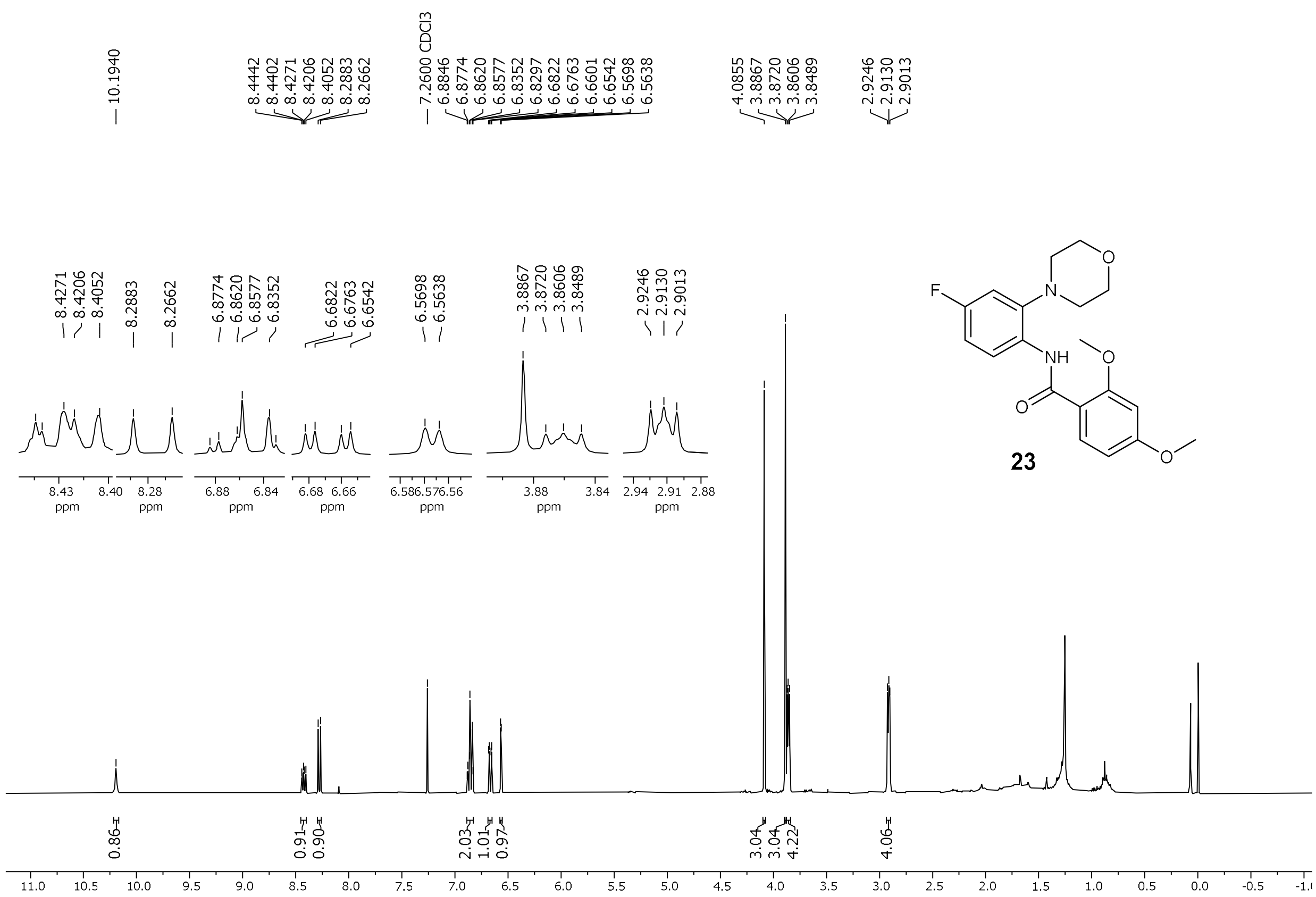

Figure S54. ^{1}H NMR spectrum of **23** (400 MHz, CDCl$_3$)

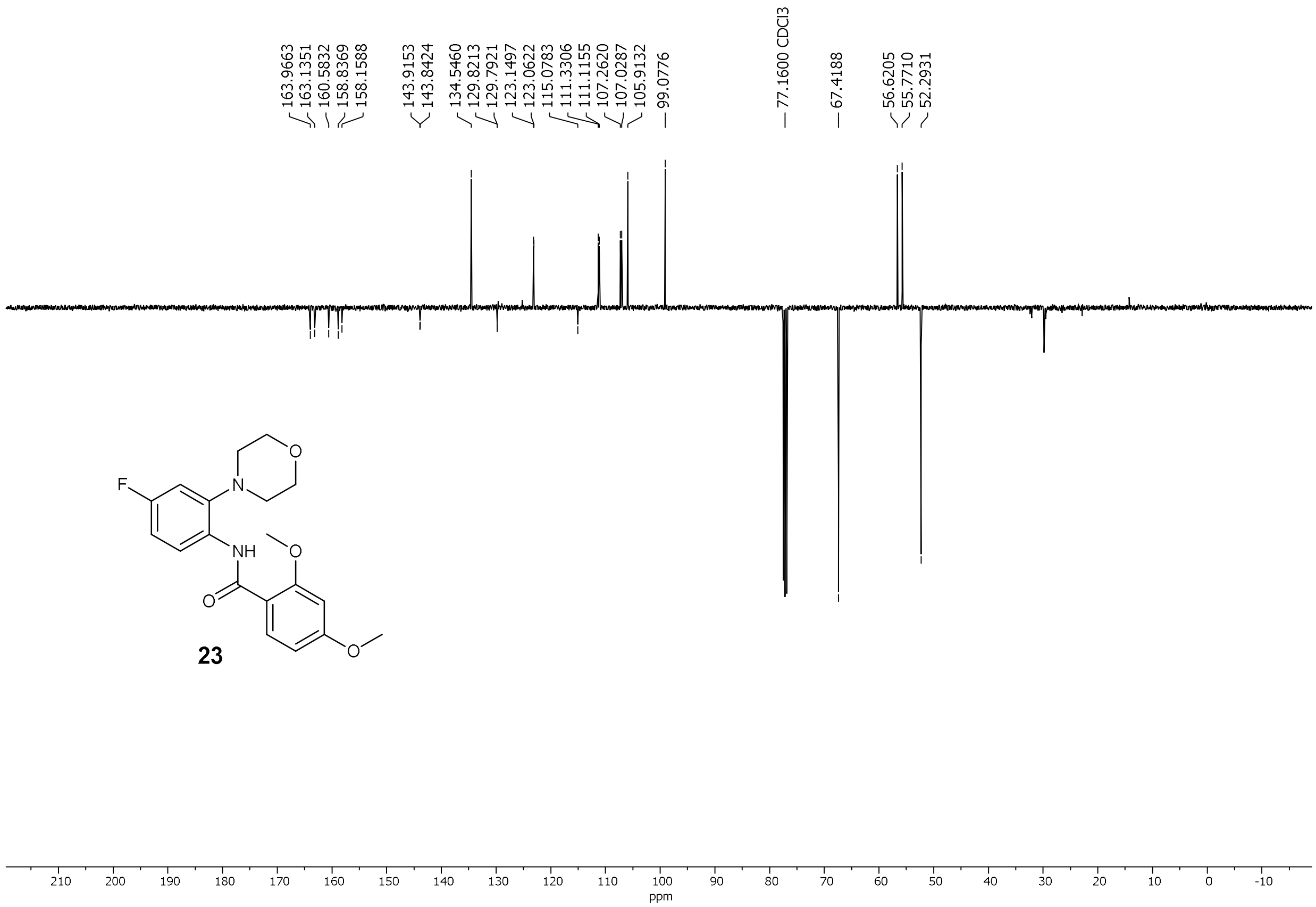

23

Figure S55. ^{13}C APT NMR spectrum of **23** (101 MHz, $CDCl_3$)

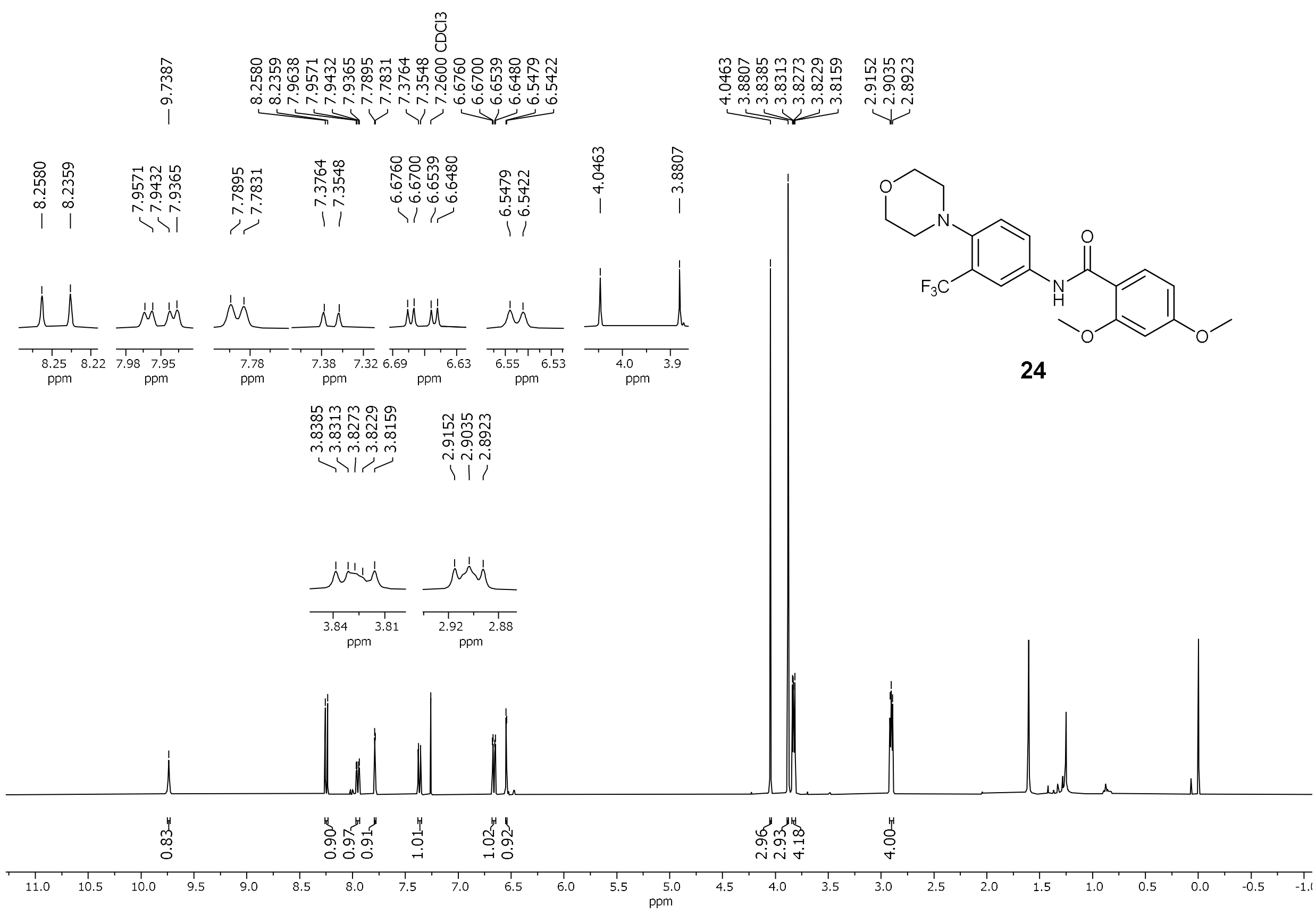

24

Figure S56. ^{1}H NMR spectrum of **24** (400 MHz, $CDCl_3$)

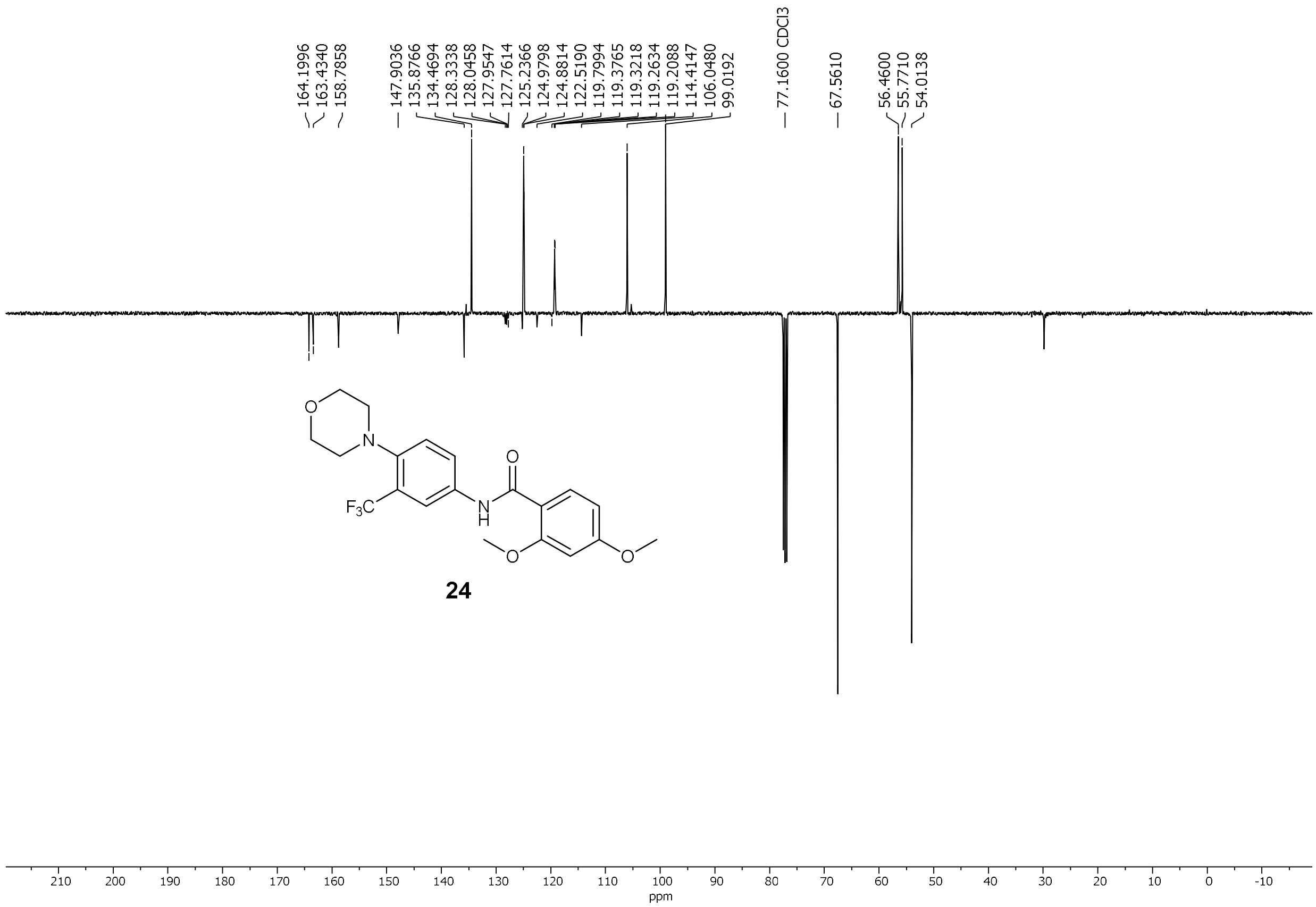

Figure S57. ^{13}C APT NMR spectrum of **24** (101 MHz, $CDCl_3$)

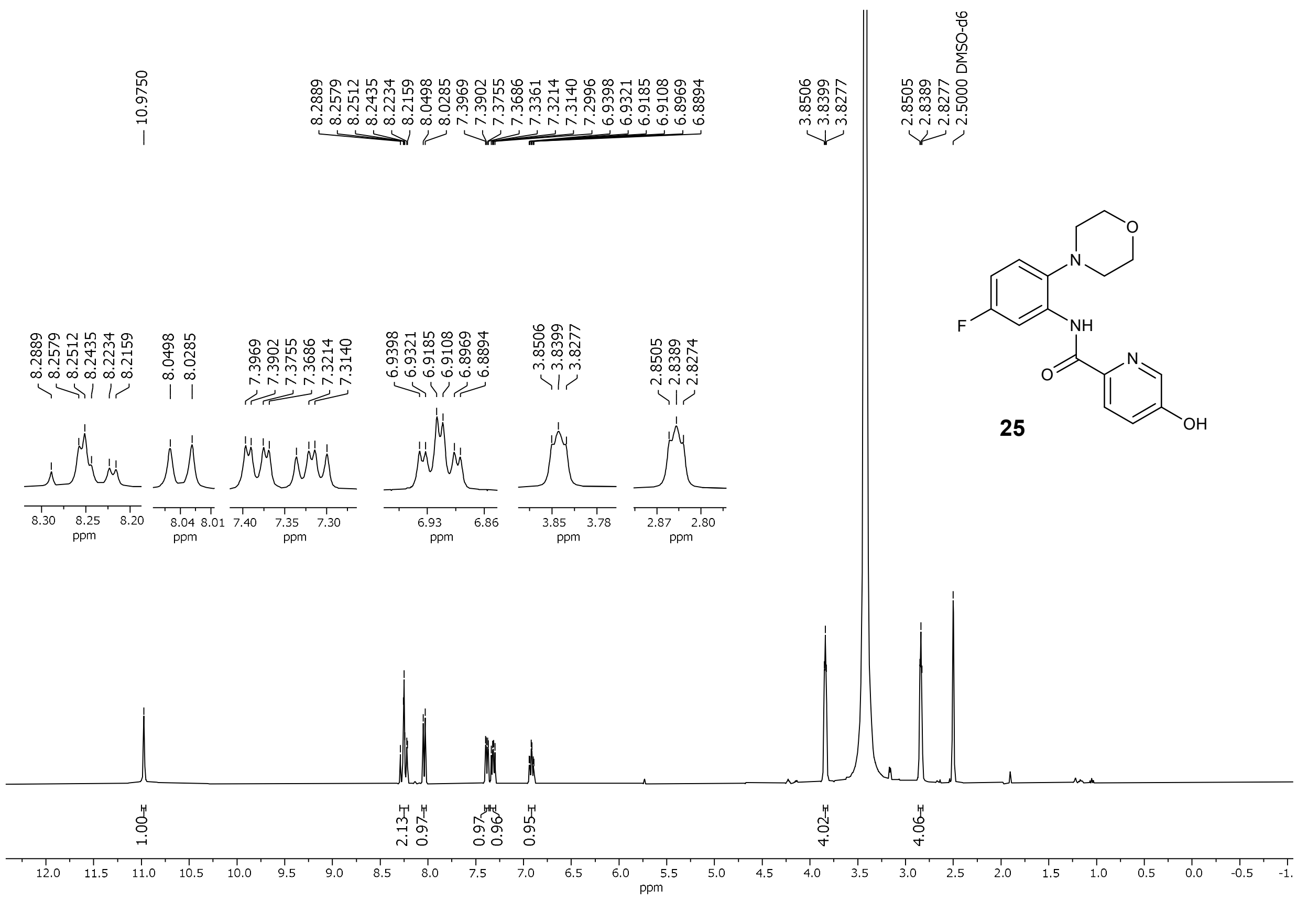

Figure S58. ^{1}H NMR spectrum of **25** (400 MHz, $CDCl_3$)

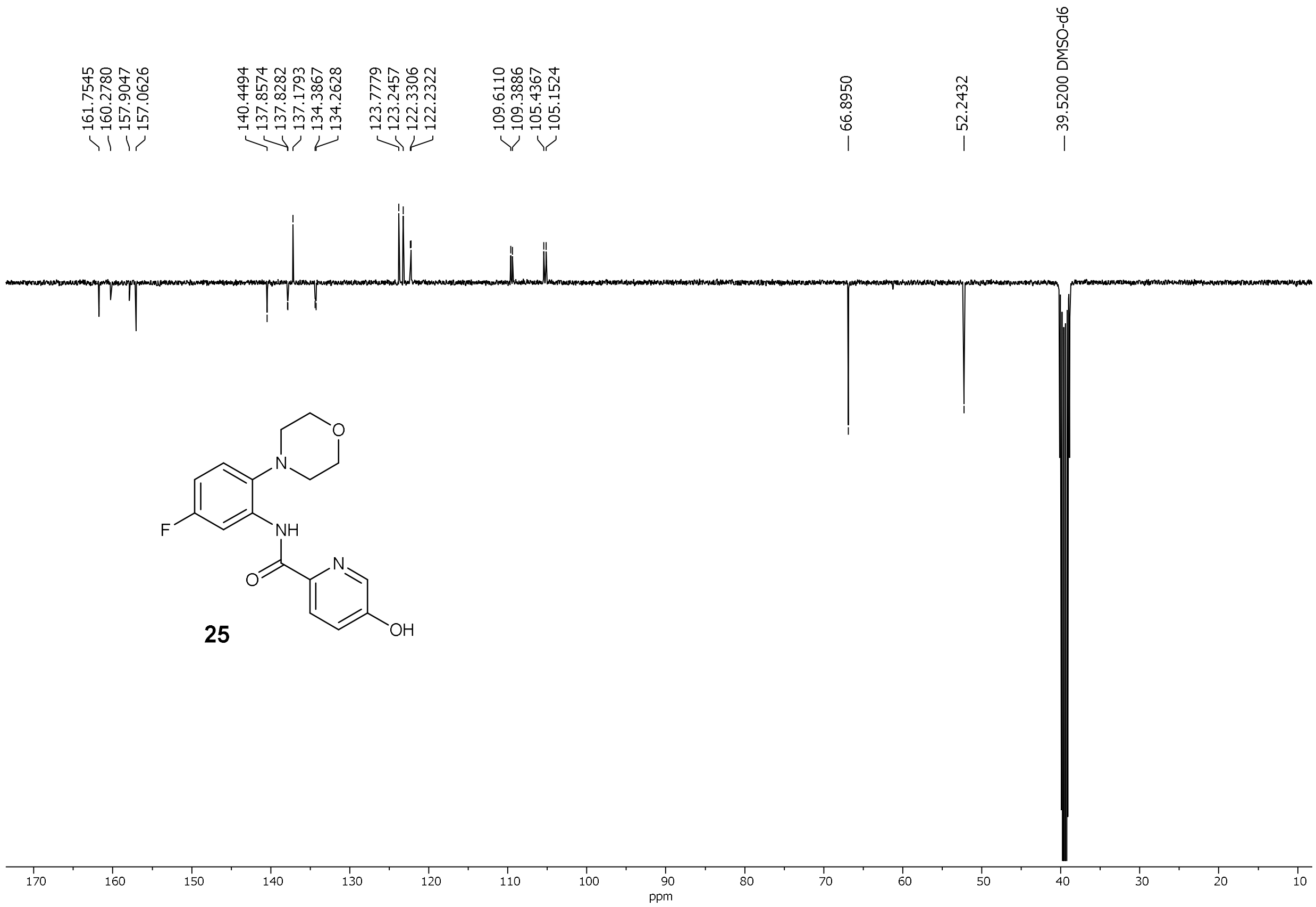

Figure S59. ^{13}C APT NMR spectrum of **25** (101 MHz, $CDCl_3$)

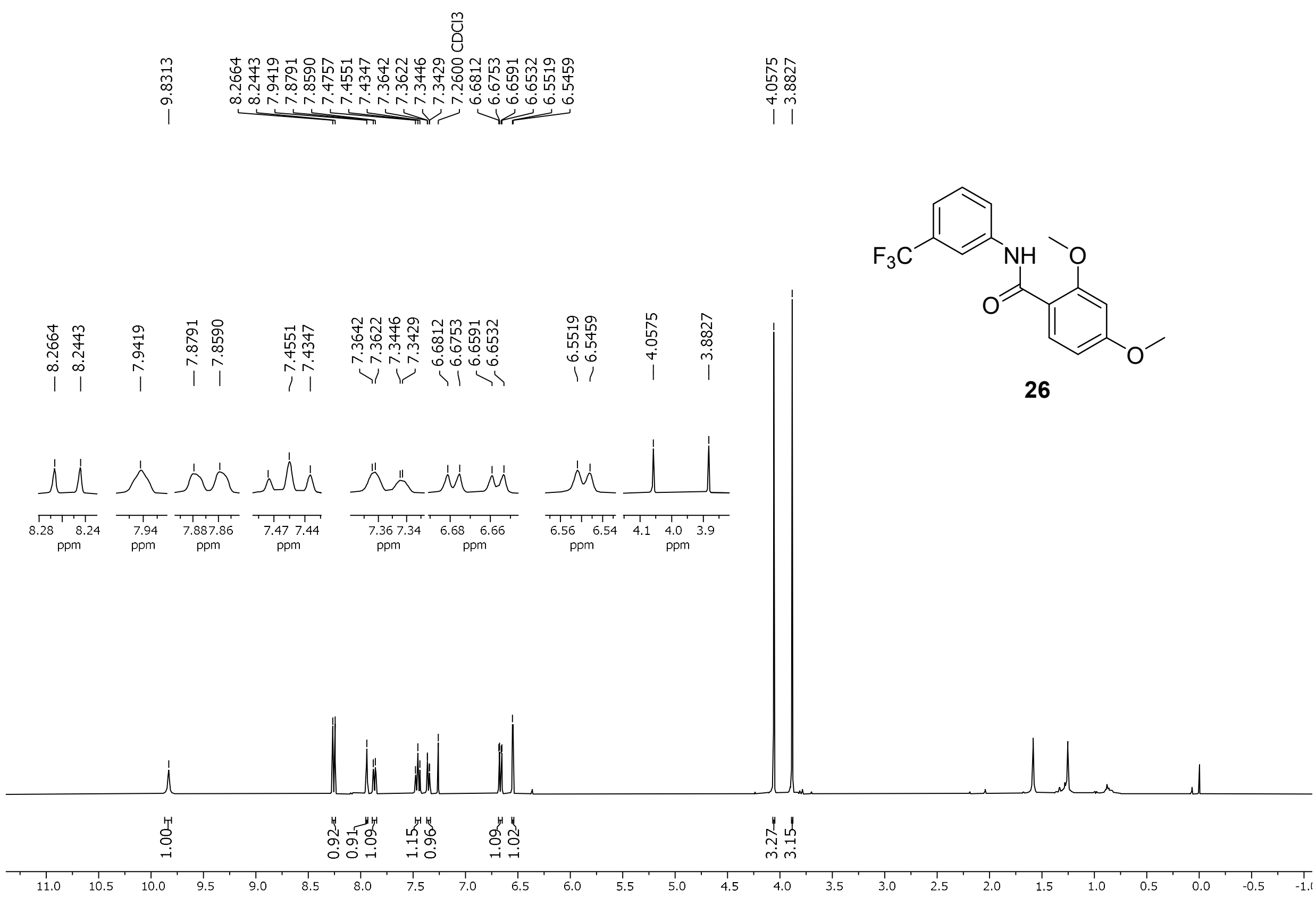

Figure S60. ^1H NMR spectrum of **26** (400 MHz, $CDCl_3$)

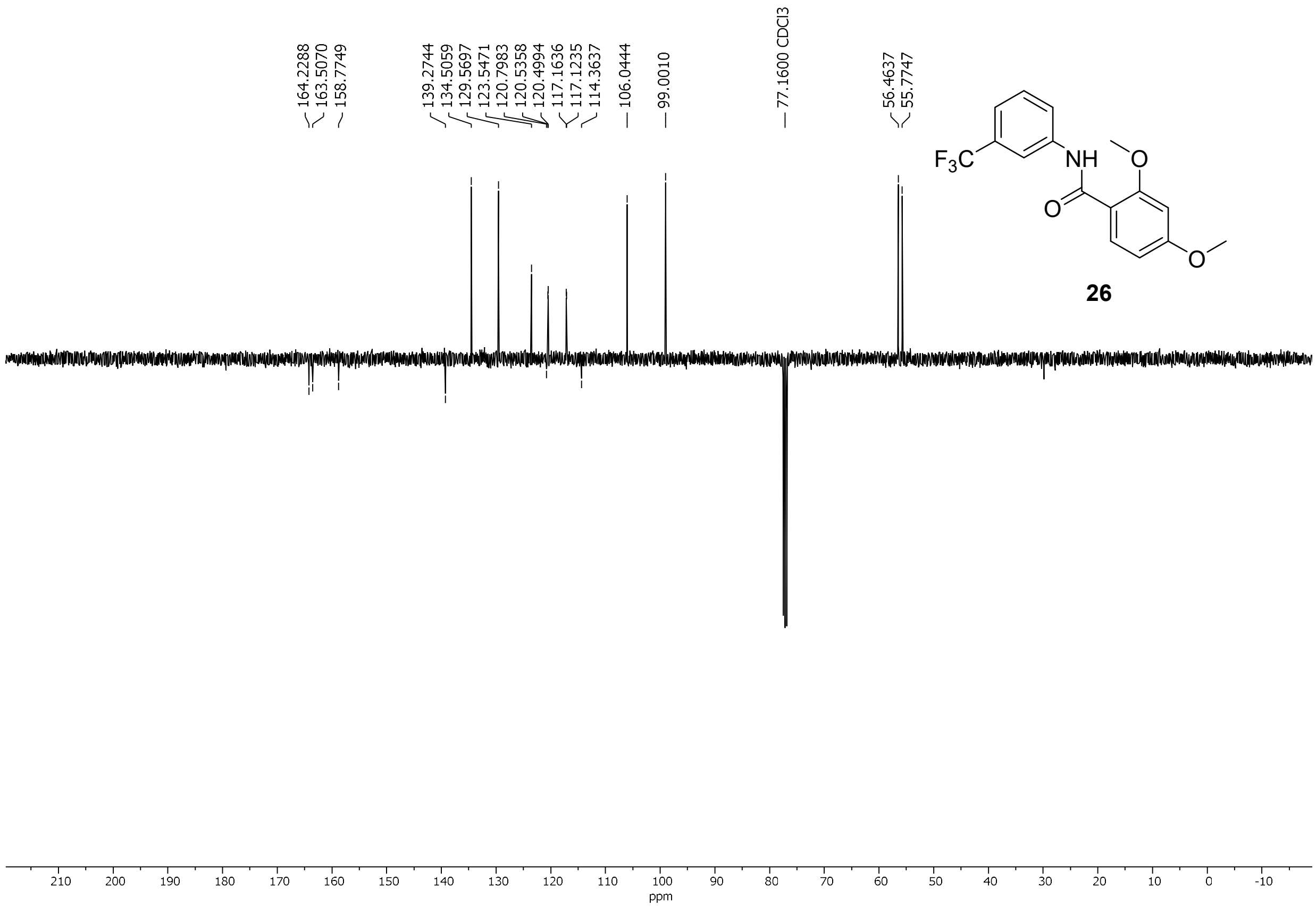

Figure S61. ^{13}C APT NMR spectrum of **26** (101 MHz, $CDCl_3$)

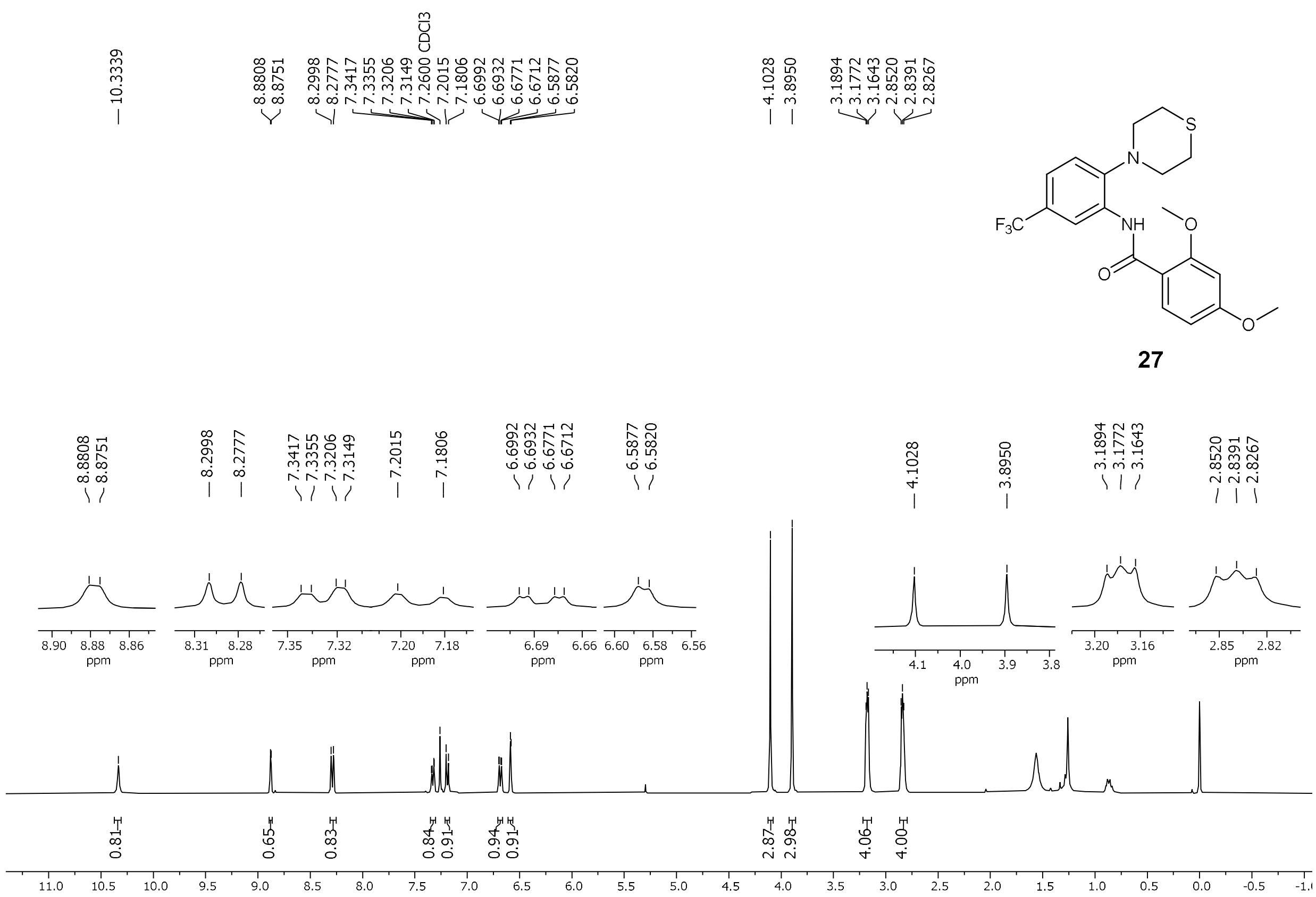

Figure S62. ^{1}H NMR spectrum of **27** (400 MHz, $CDCl_3$)

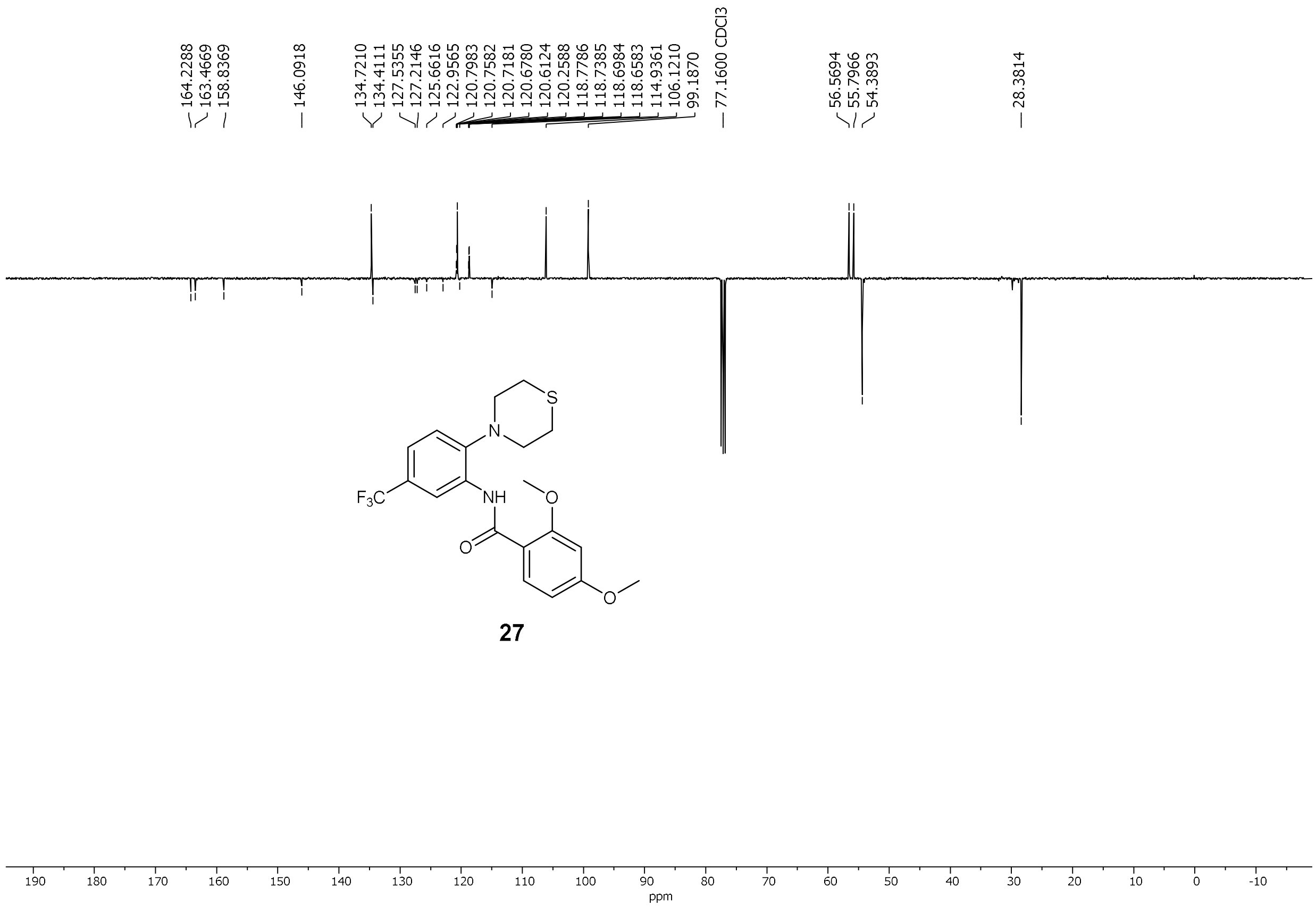

Figure S63. ^{13}C APT NMR spectrum of **27** (101 MHz, $CDCl_3$)

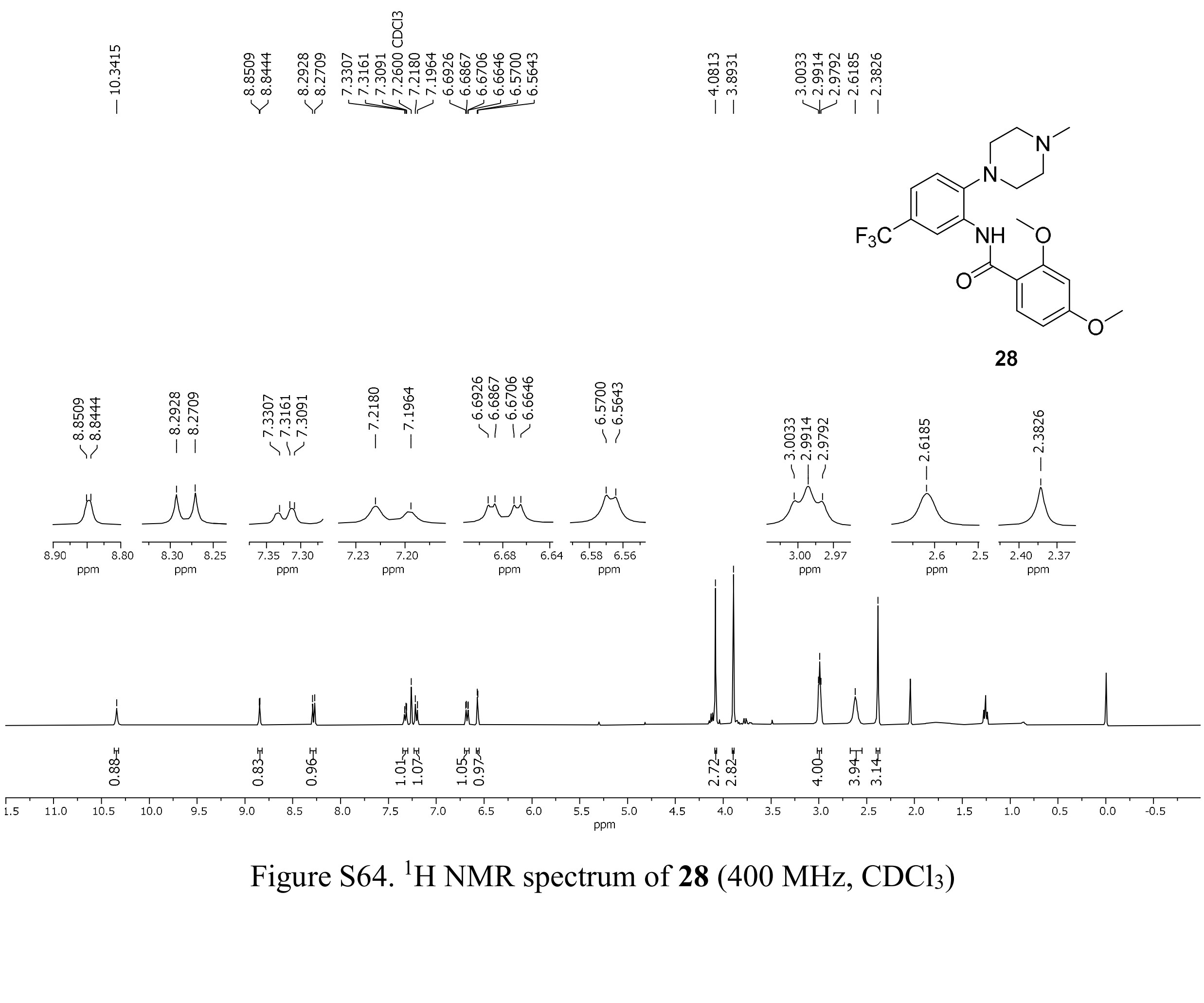

Figure S64. ^{1}H NMR spectrum of **28** (400 MHz, $CDCl_3$)

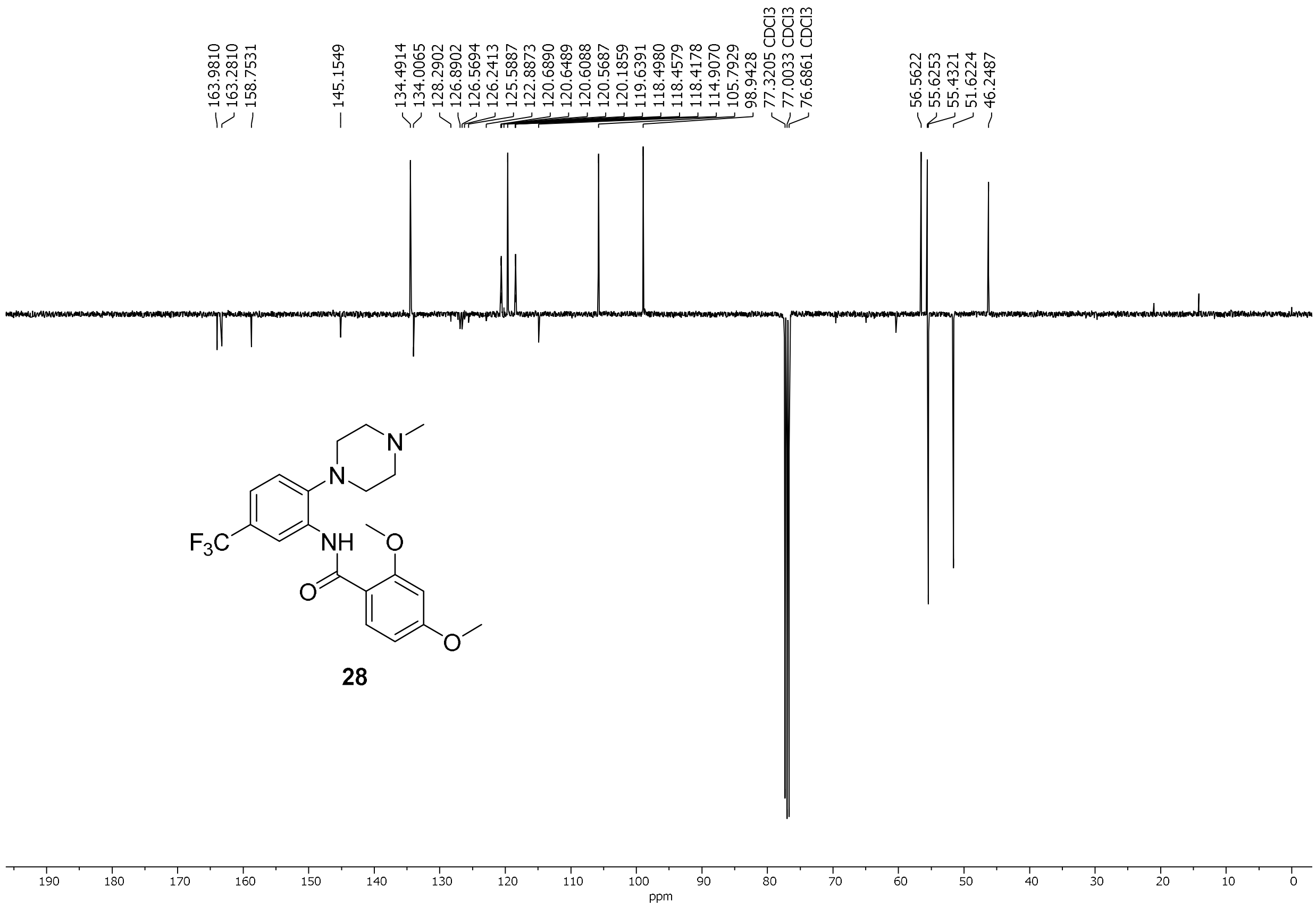

Figure S65. ^{13}C APT NMR spectrum of **28** (101 MHz, $CDCl_3$)

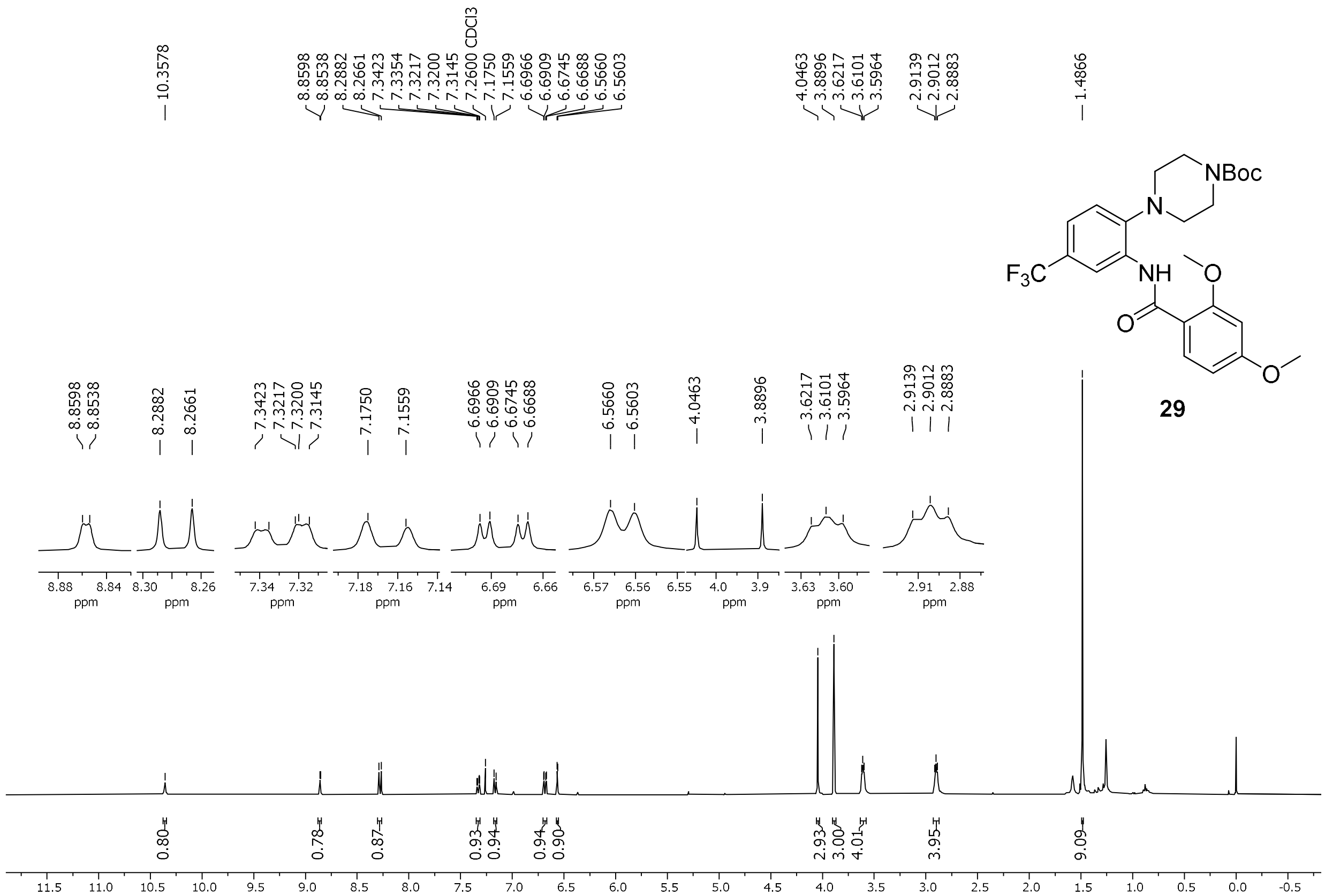

Figure S66. ^{1}H NMR spectrum of **29** (400 MHz, $CDCl_3$)

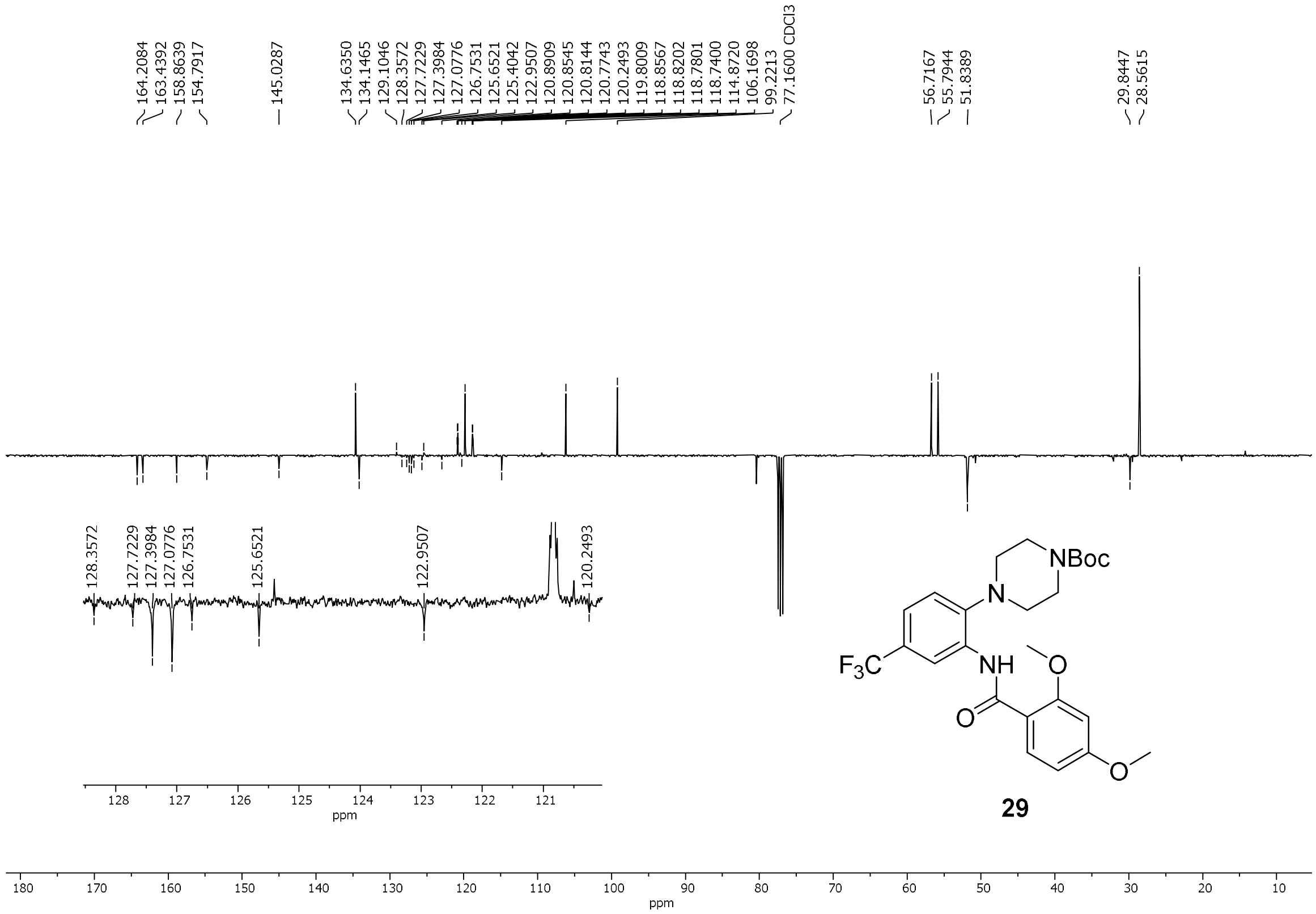

Figure S67. ^{13}C APT NMR spectrum of **29** (101 MHz, $CDCl_3$)

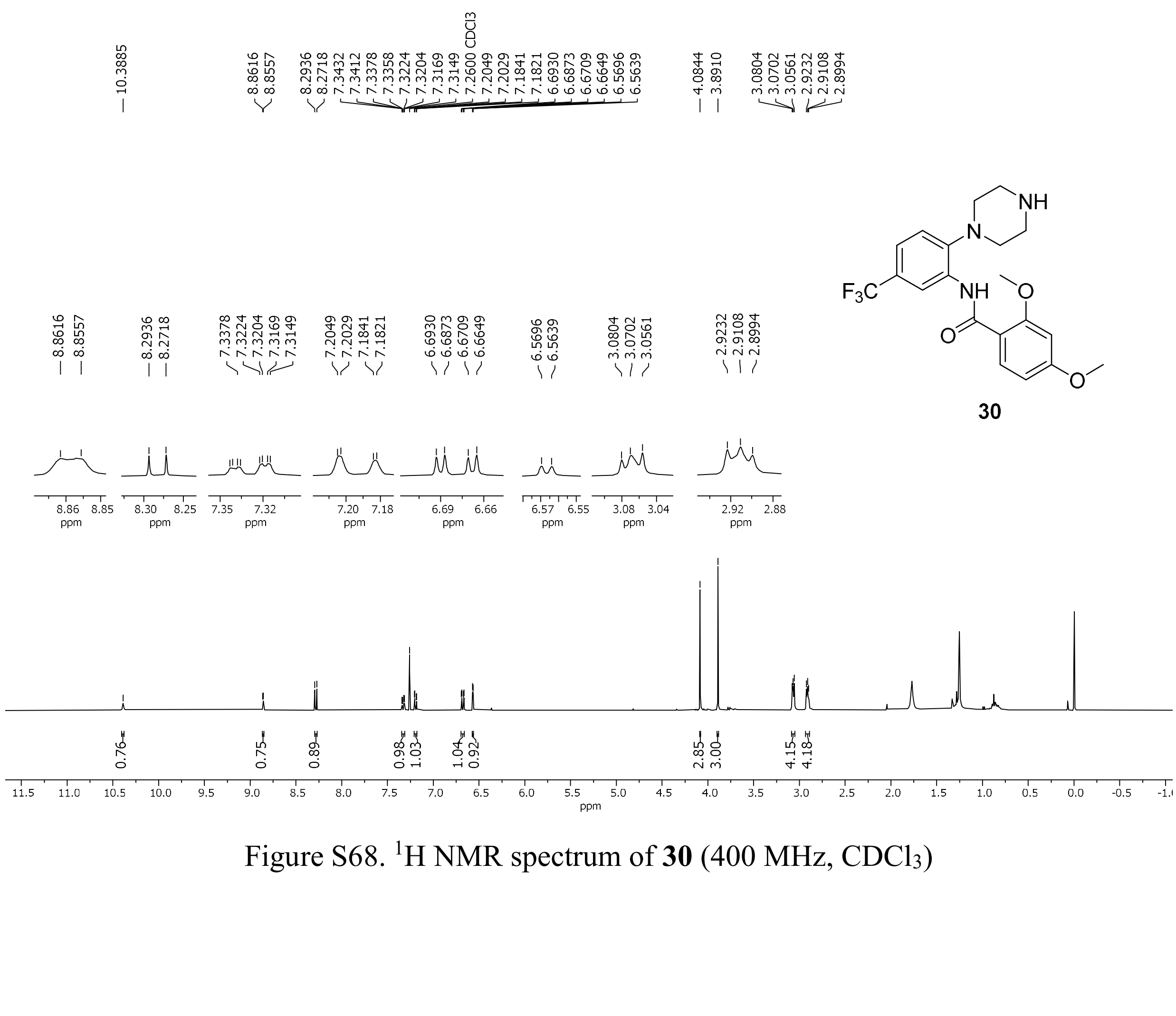

Figure S68. ^{1}H NMR spectrum of **30** (400 MHz, $CDCl_3$)

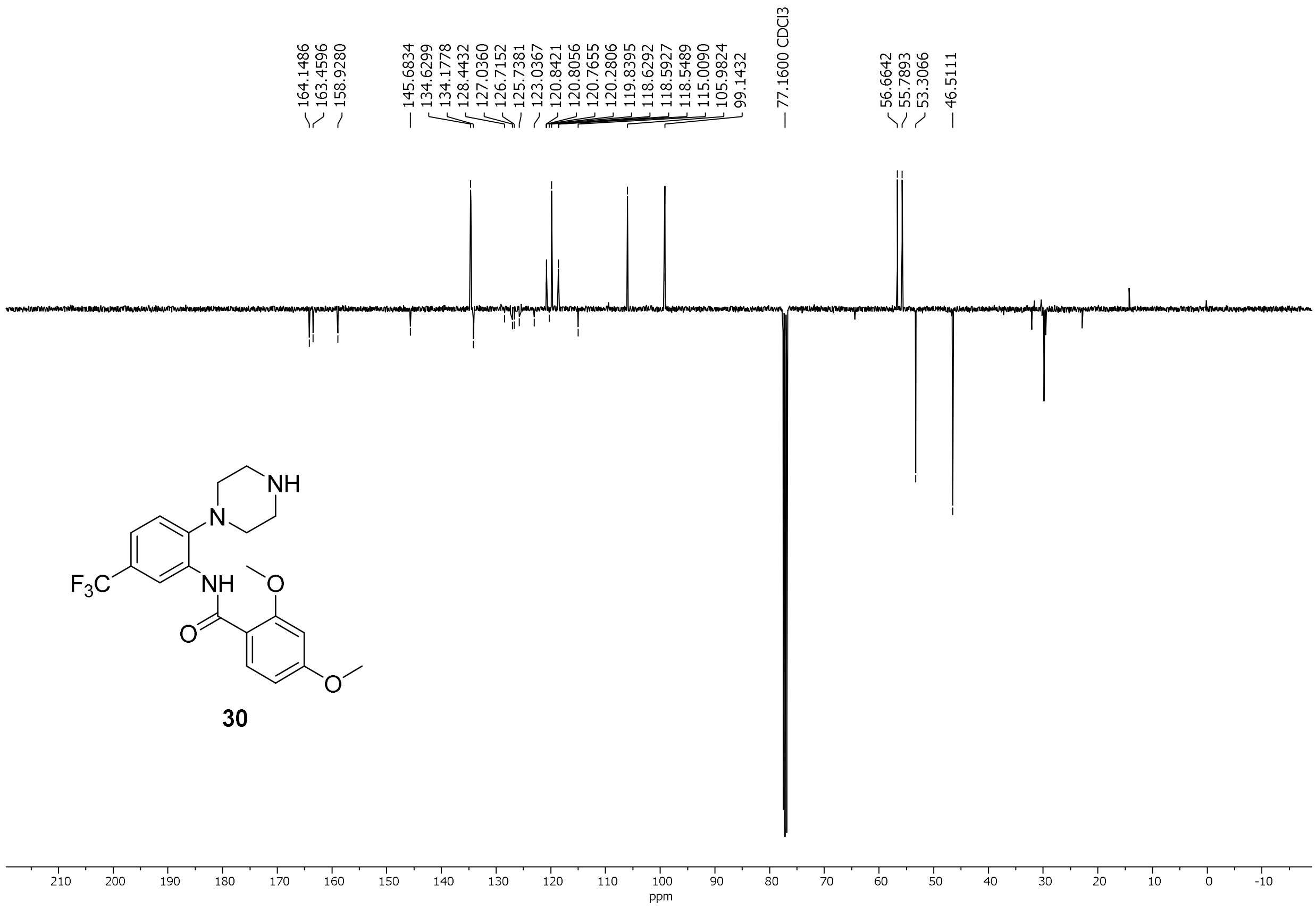

Figure S69. ^{1}C APT NMR spectrum of **30** (101 MHz, $CDCl_3$)

2. HRMS spectra of compounds hit 1 and 7 - 30

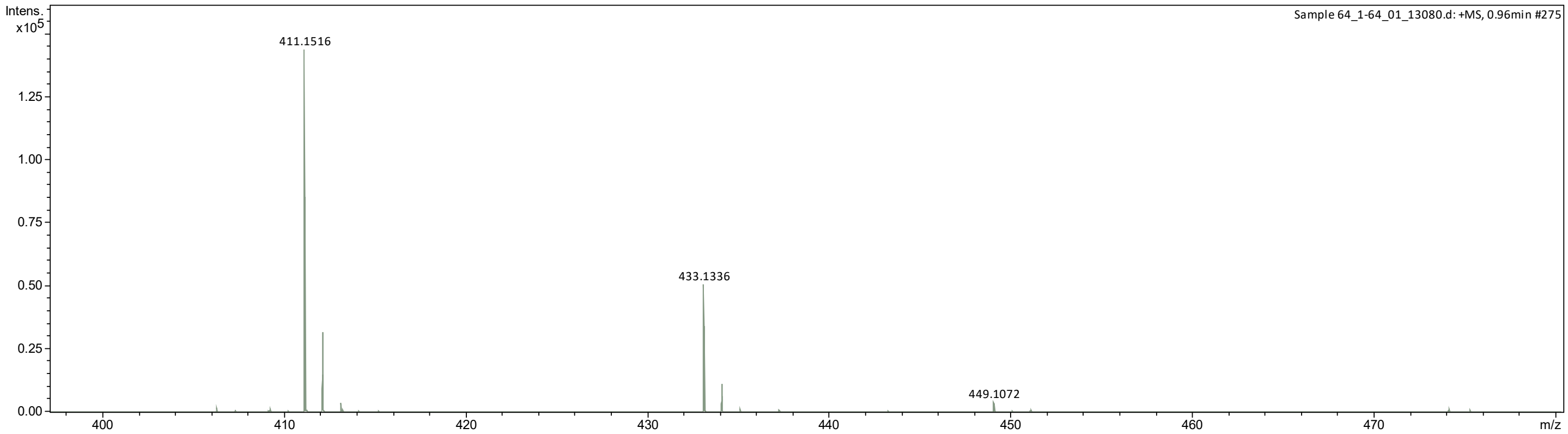

Figure S70. HRMS (ESI +) spectrum of hit **1**

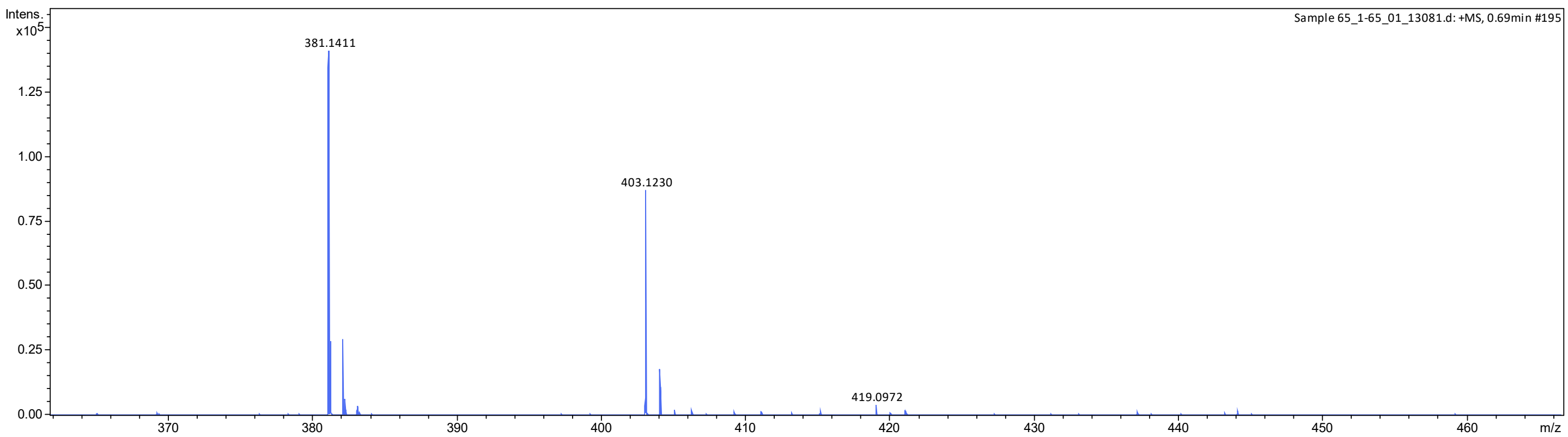

Figure S71. (ESI +) spectrum of **7**

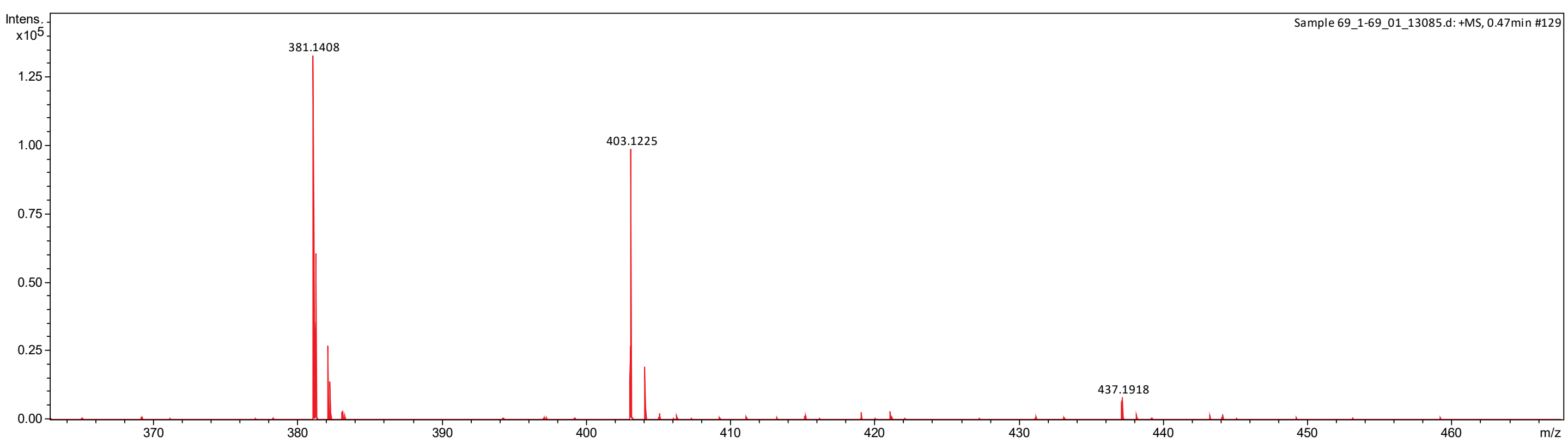

Figure S72. (ESI +) spectrum of **8**

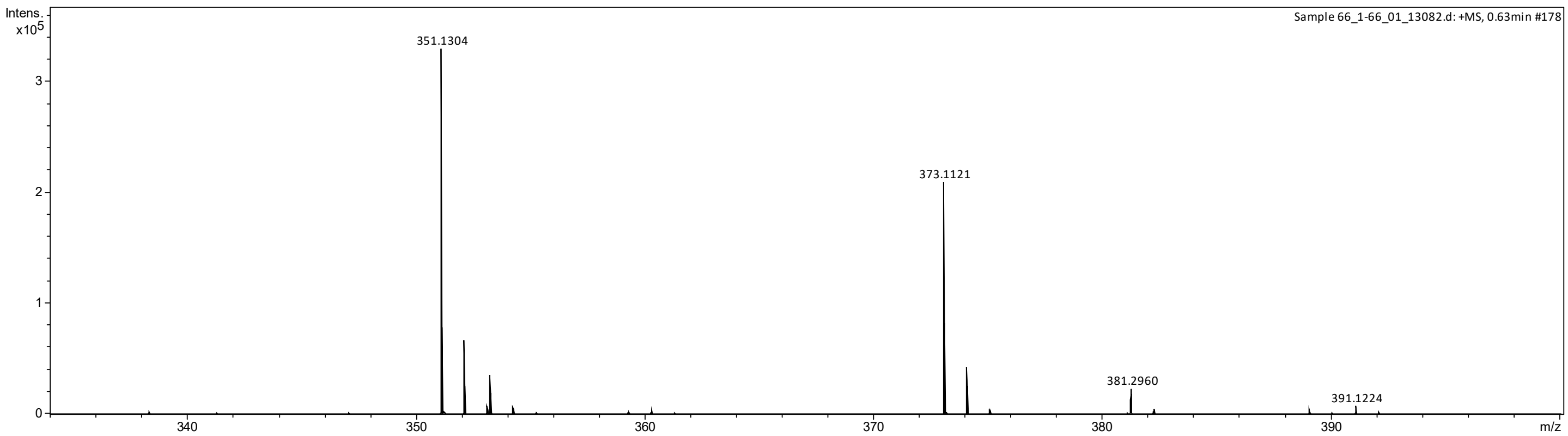

Figure S73. HRMS (ESI +) spectrum of **9**

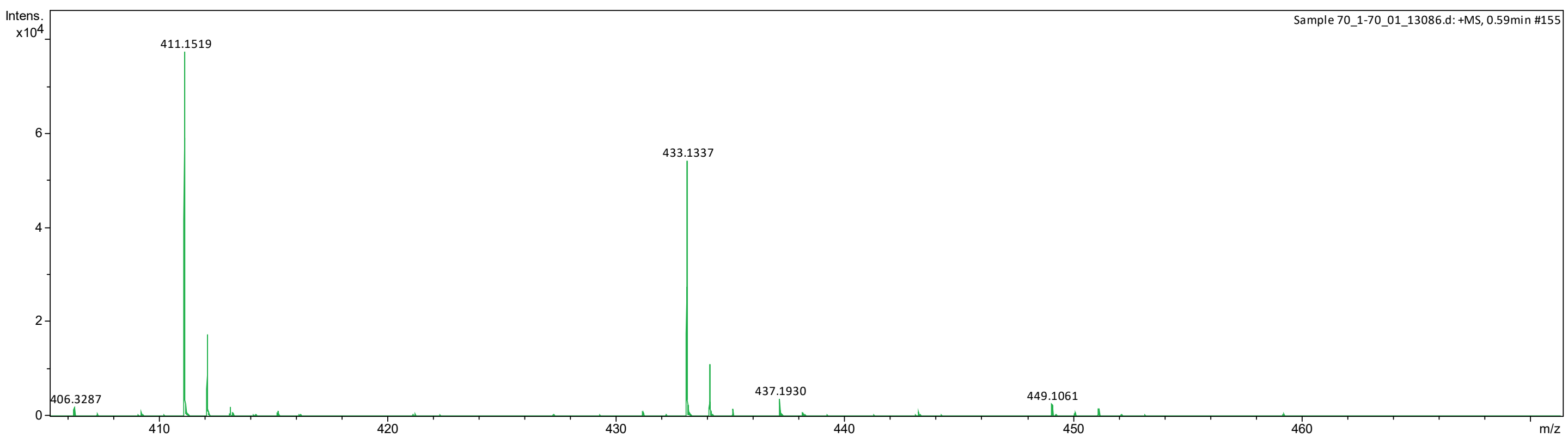

Figure S74. HRMS (ESI +) spectrum of **10**

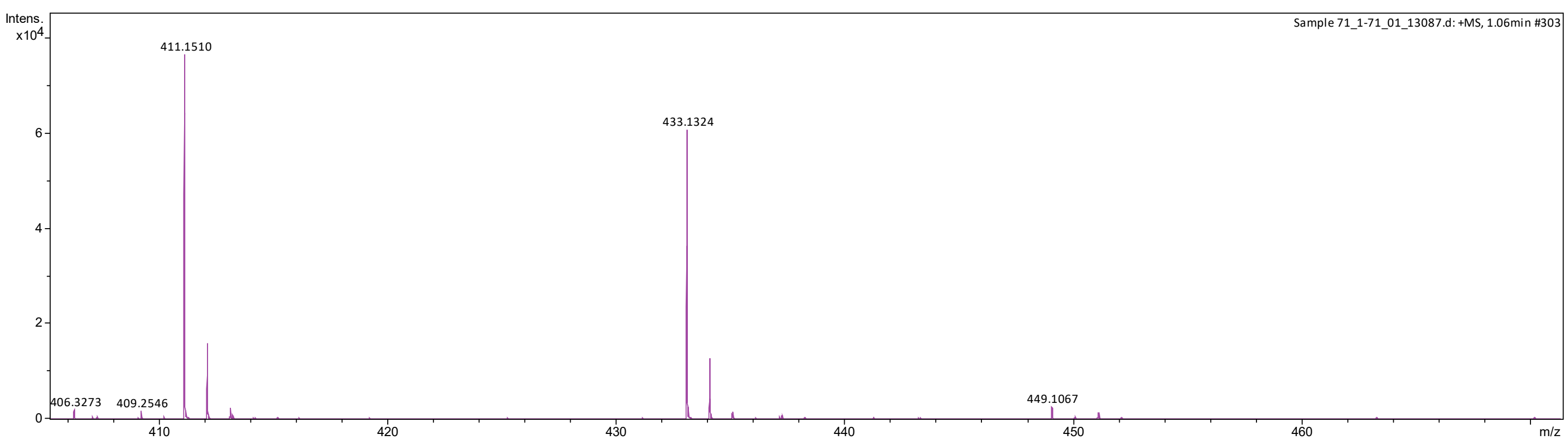

Figure S75. HRMS (ESI +) spectrum of **11**

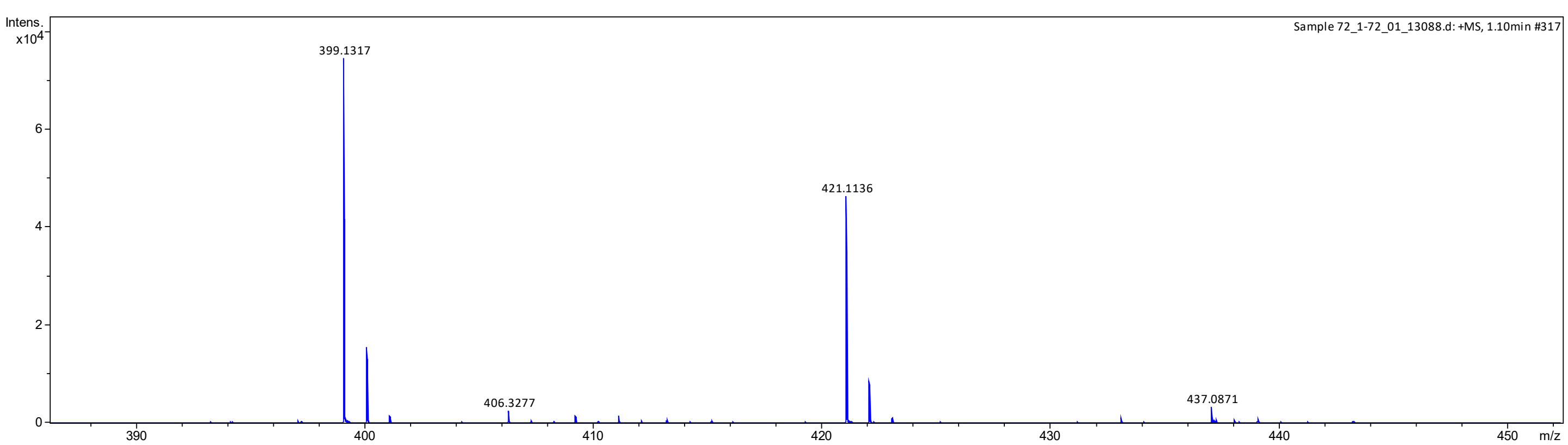

Figure S76. HRMS (ESI +) spectrum of **12**

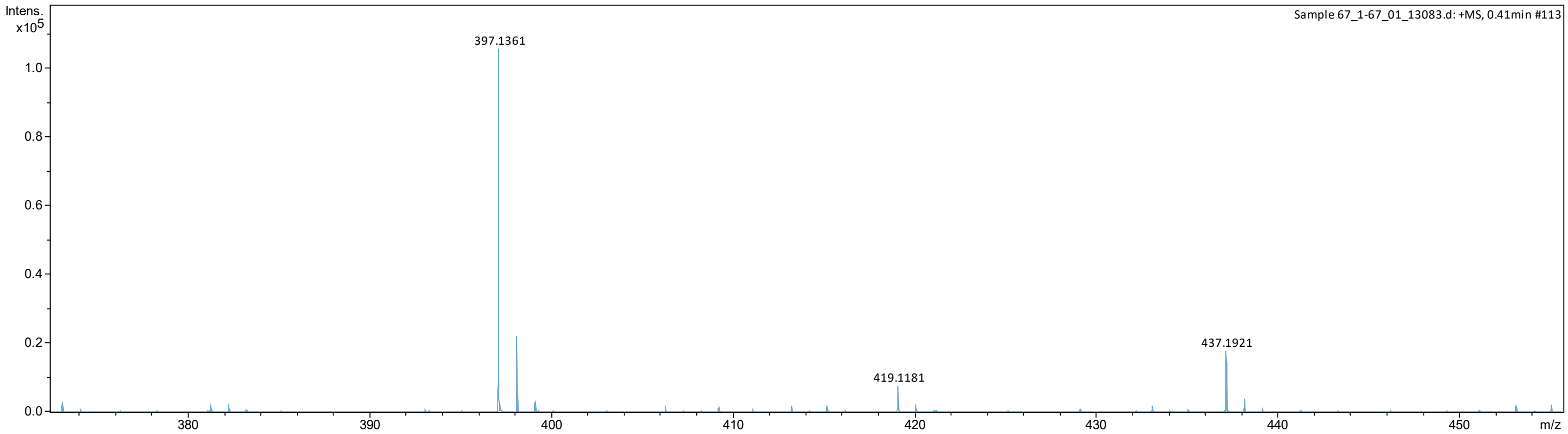

Figure S77. HRMS (ESI +) spectrum of **13**

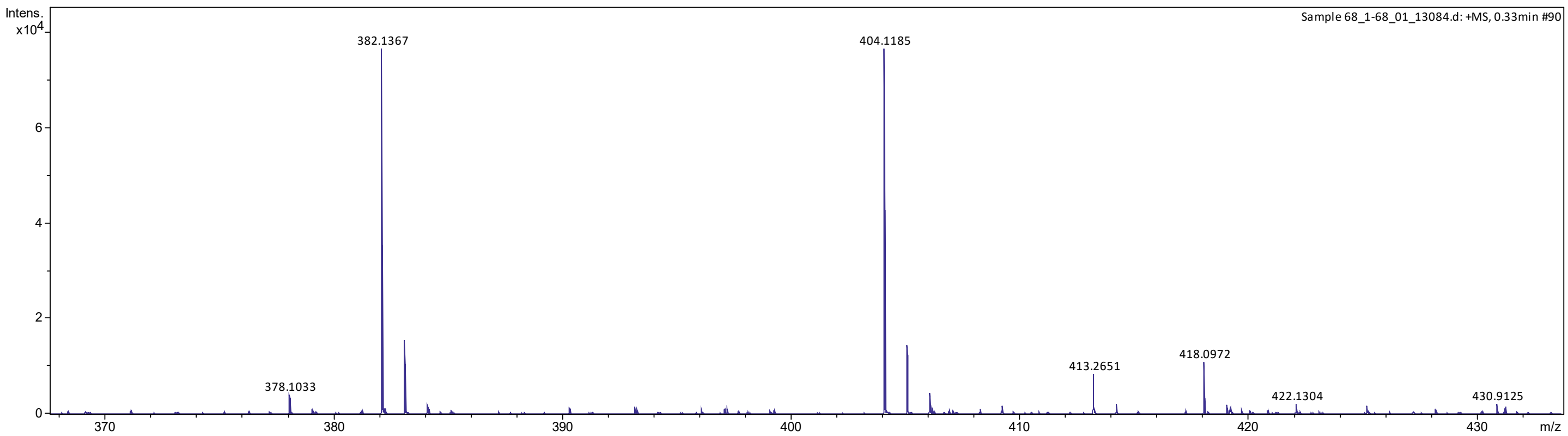

Figure S78. HRMS (ESI +) spectrum of **14**

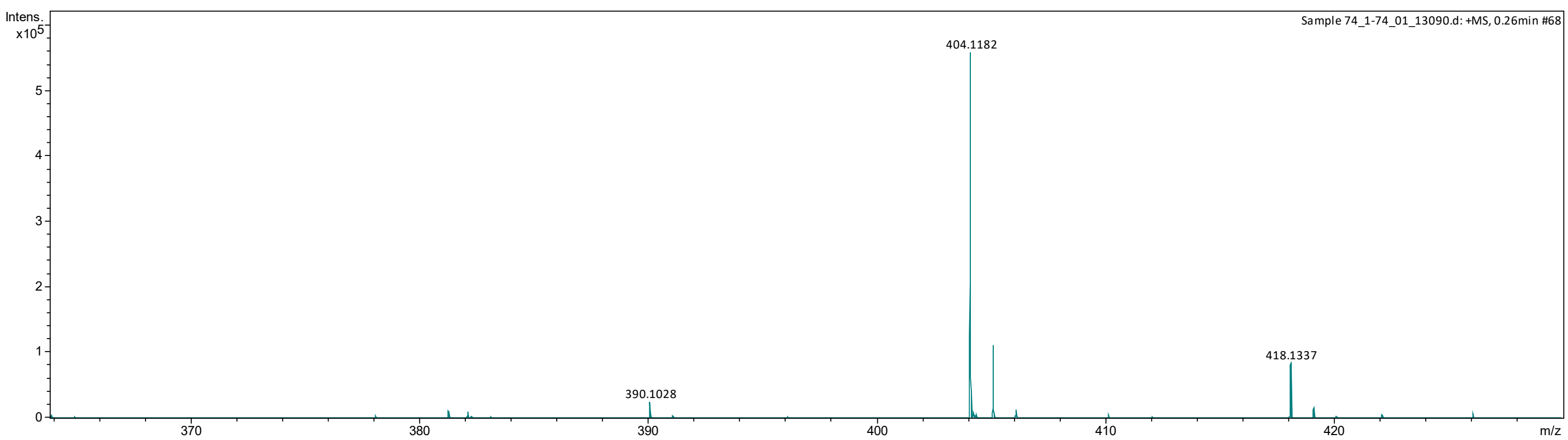

Figure S79. HRMS (ESI +) spectrum of **15**

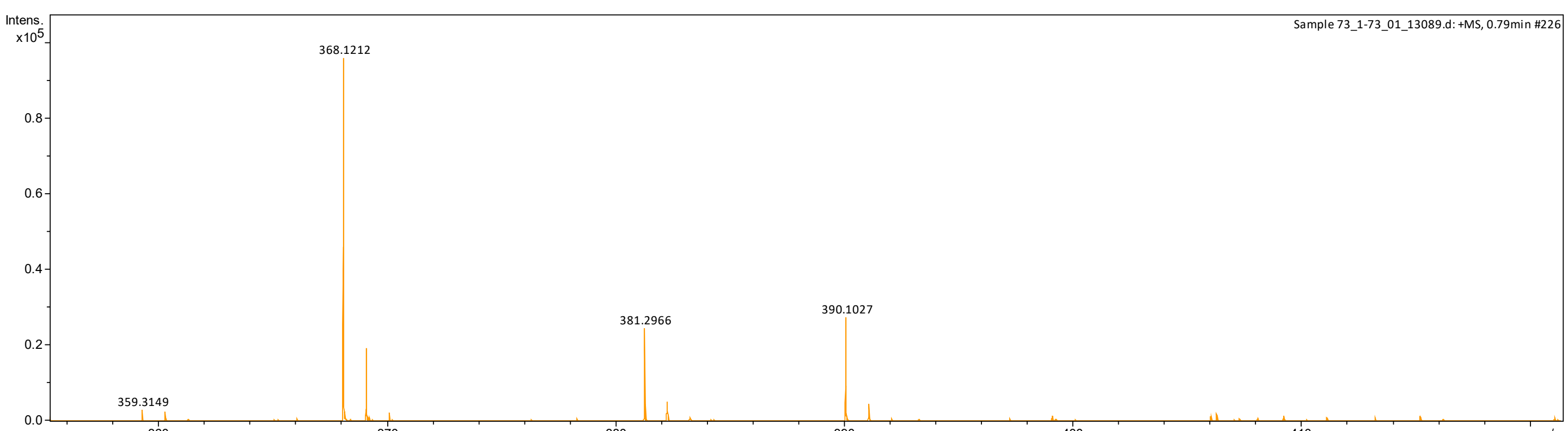

Figure S80. HRMS (ESI +) spectrum of **16**

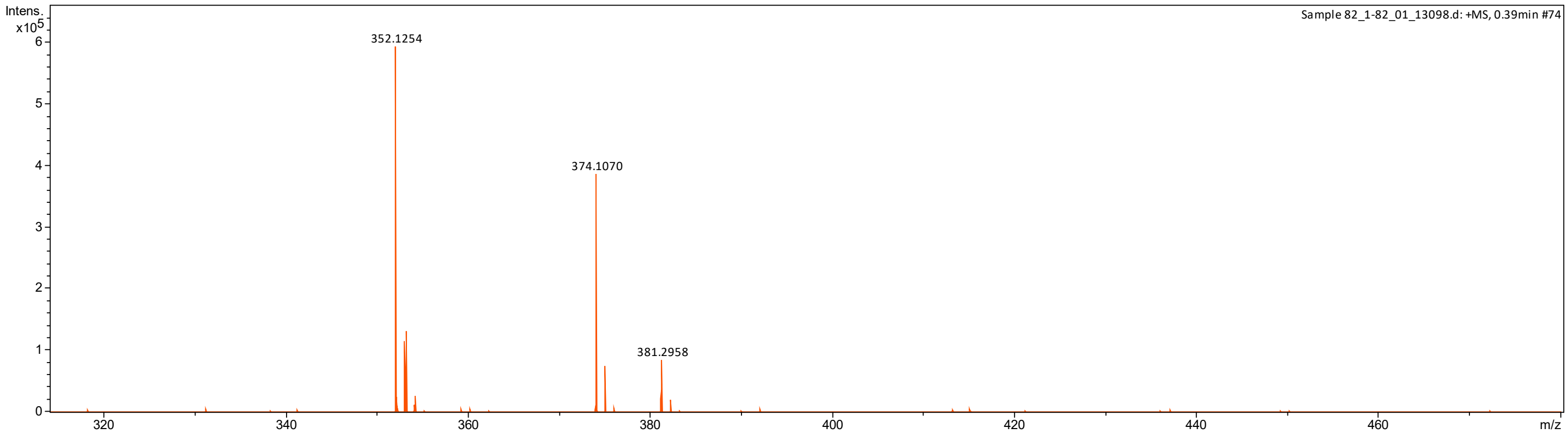

Figure S81. HRMS (ESI +) spectrum of **17**

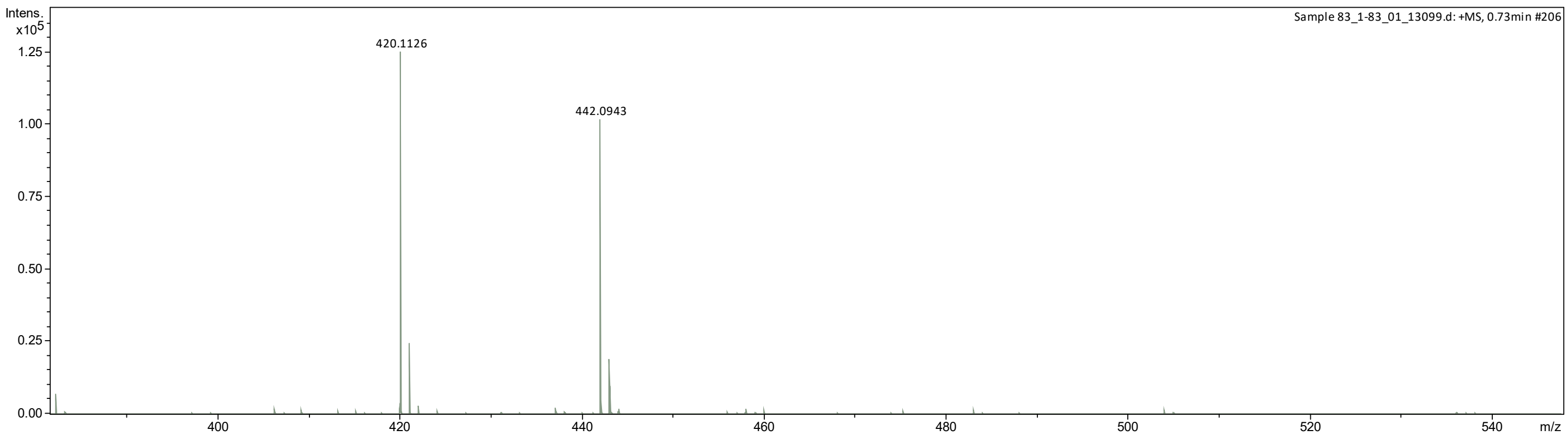

Figure S82. HRMS (ESI +) spectrum of **18**

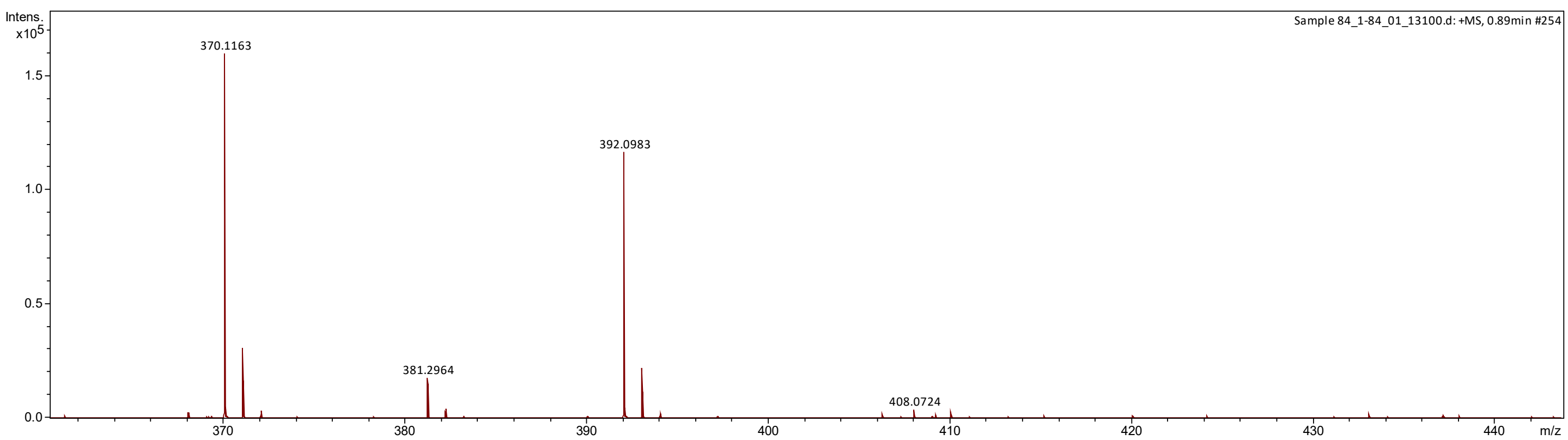

Figure S83. HRMS (ESI +) spectrum of **19**

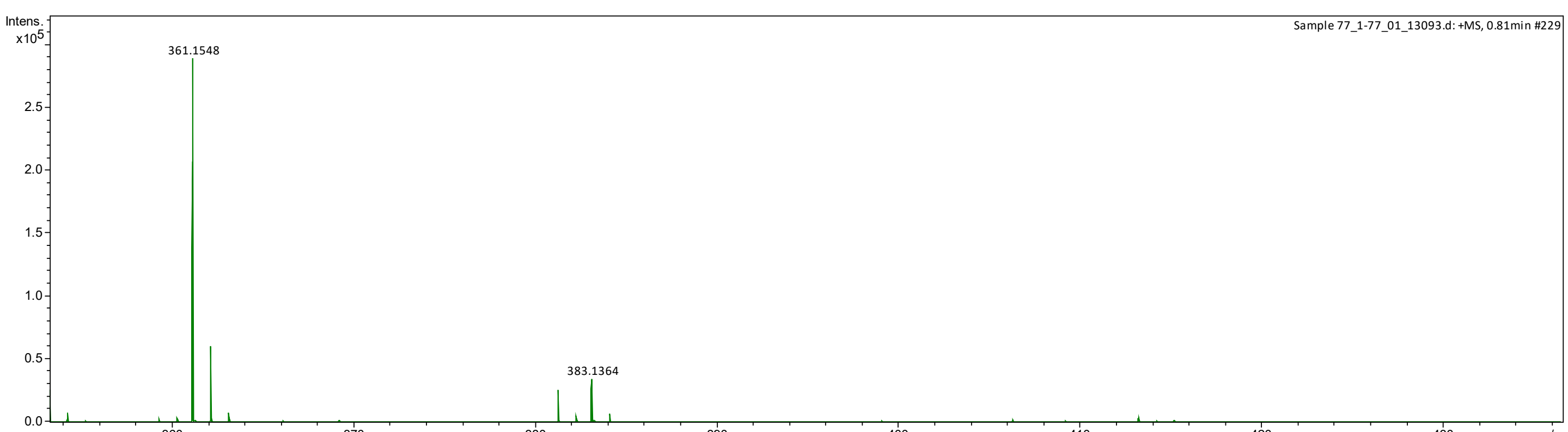

Figure S84. HRMS (ESI +) spectrum of **20**

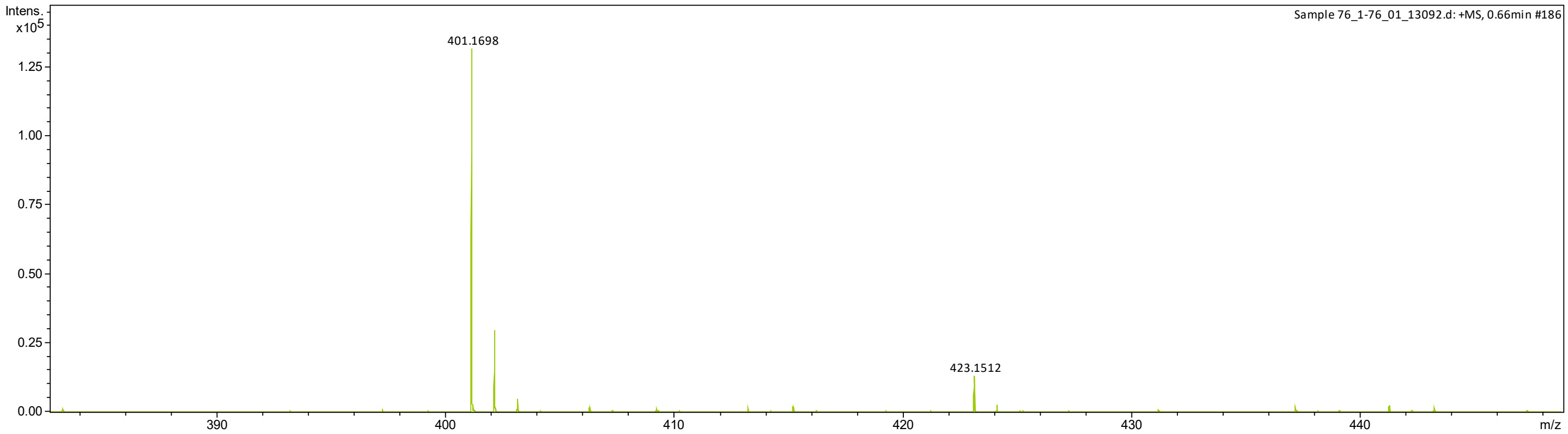

Figure S85. HRMS (ESI +) spectrum of **21**

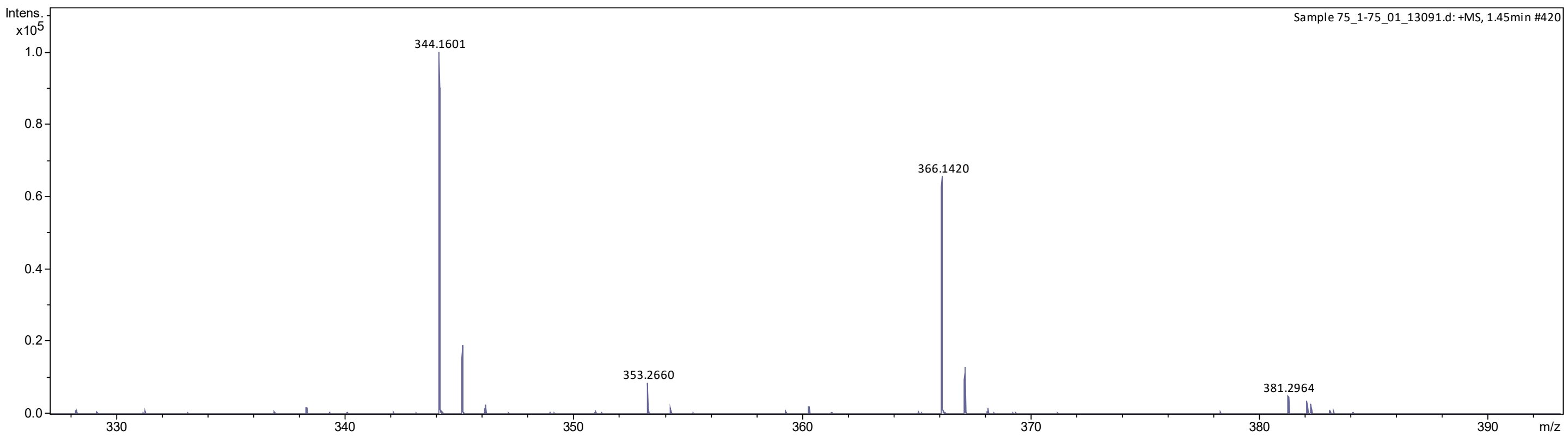

Figure S86. HRMS (ESI +) spectrum of **22**

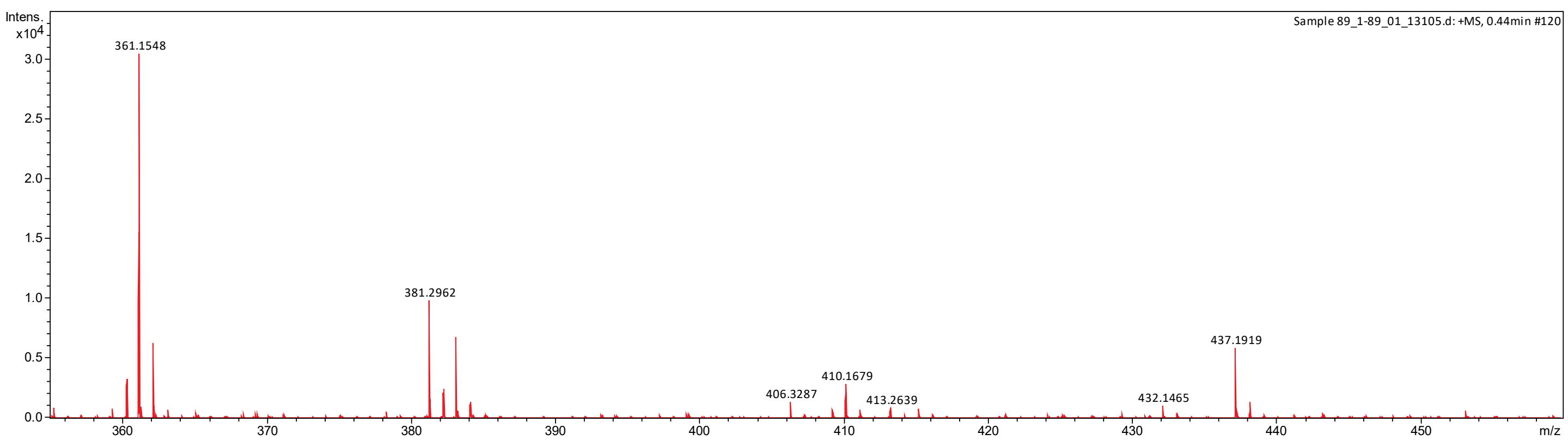

Figure S87. HRMS (ESI +) spectrum of **23**

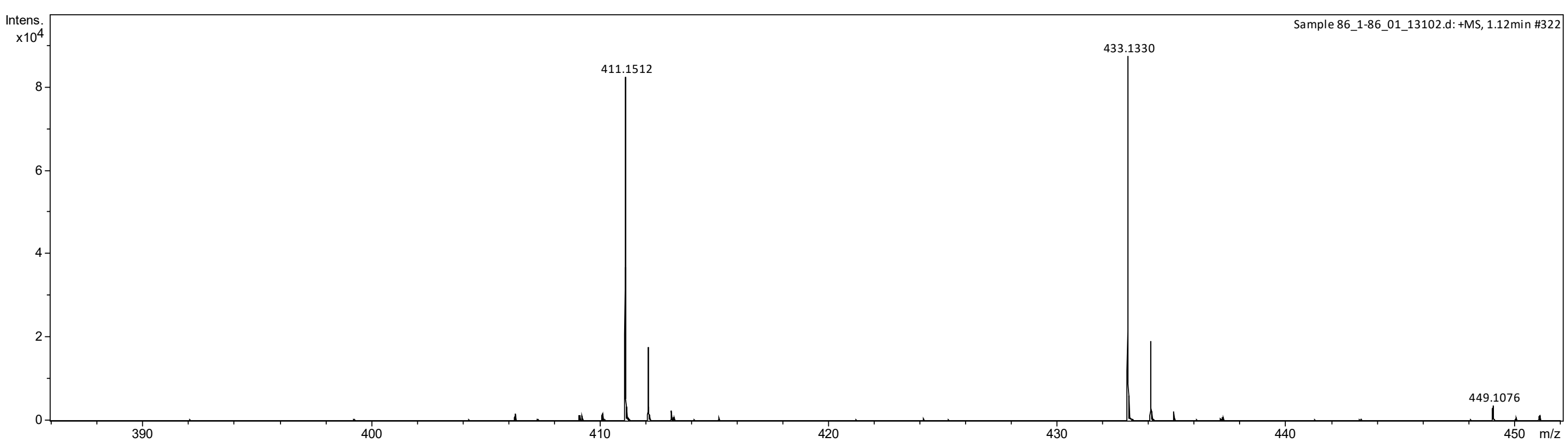

Figure S88. HRMS (ESI +) spectrum of **24**

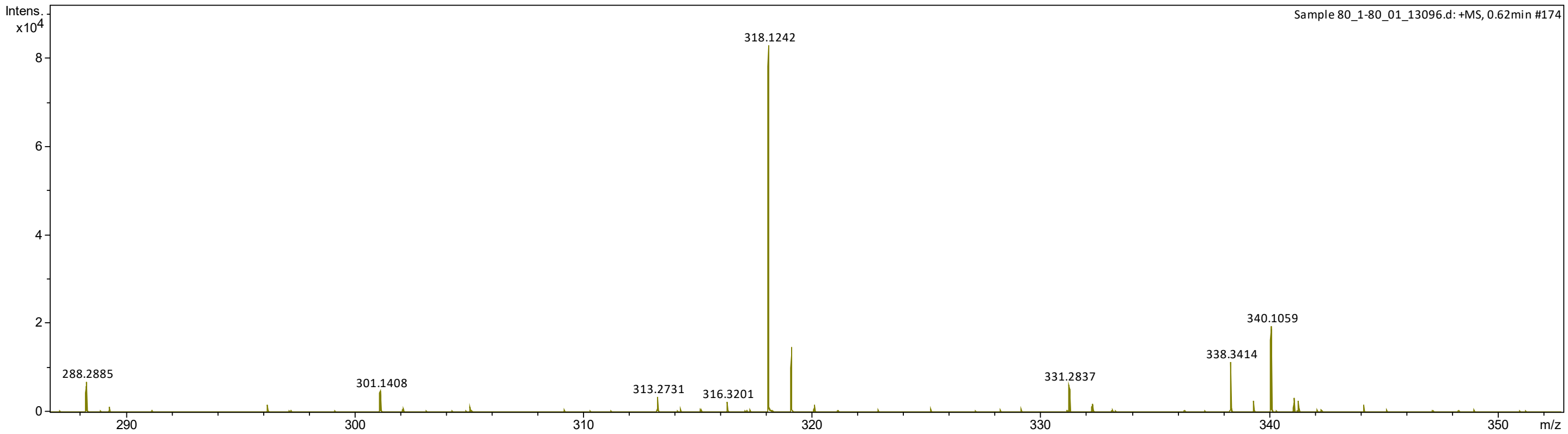

Figure S89. HRMS (ESI +) spectrum of **25**

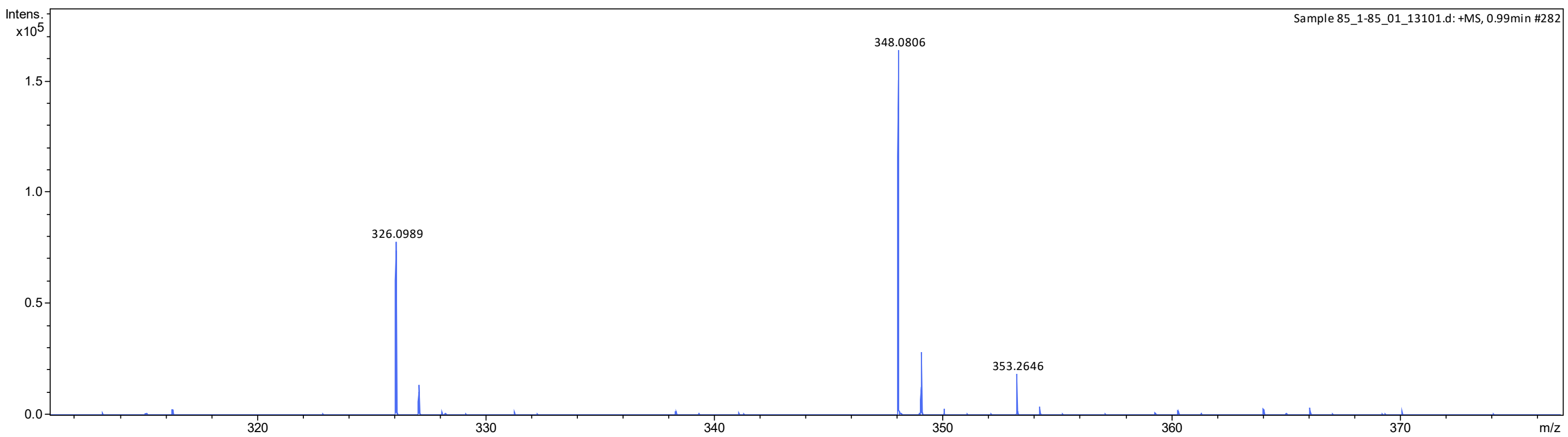

Figure S90. HRMS (ESI +) spectrum of **26**

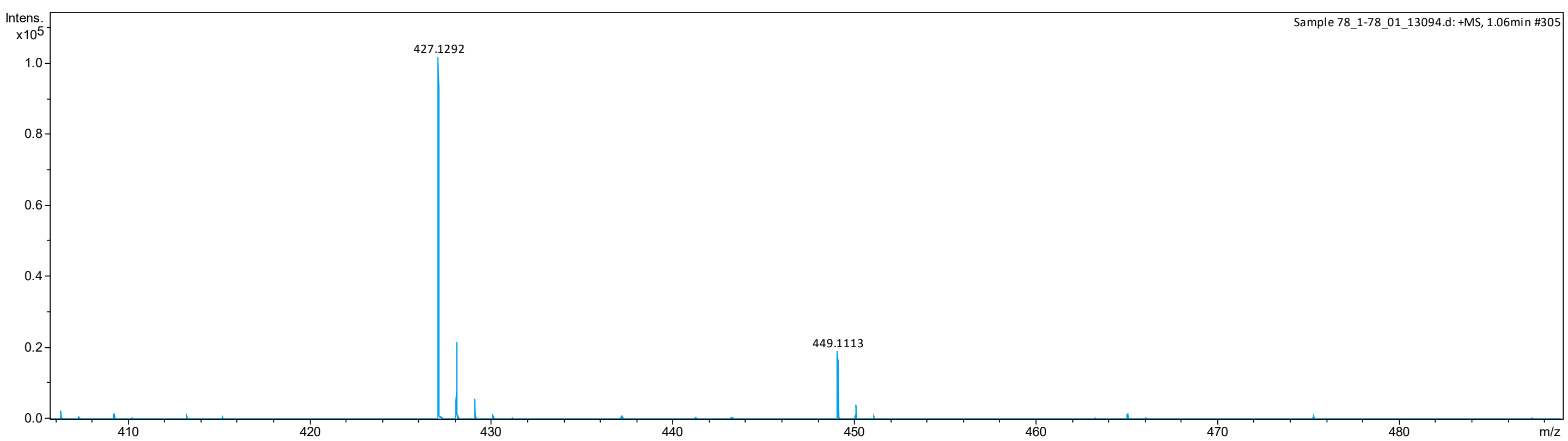

Figure S91. HRMS (ESI +) spectrum of **27**

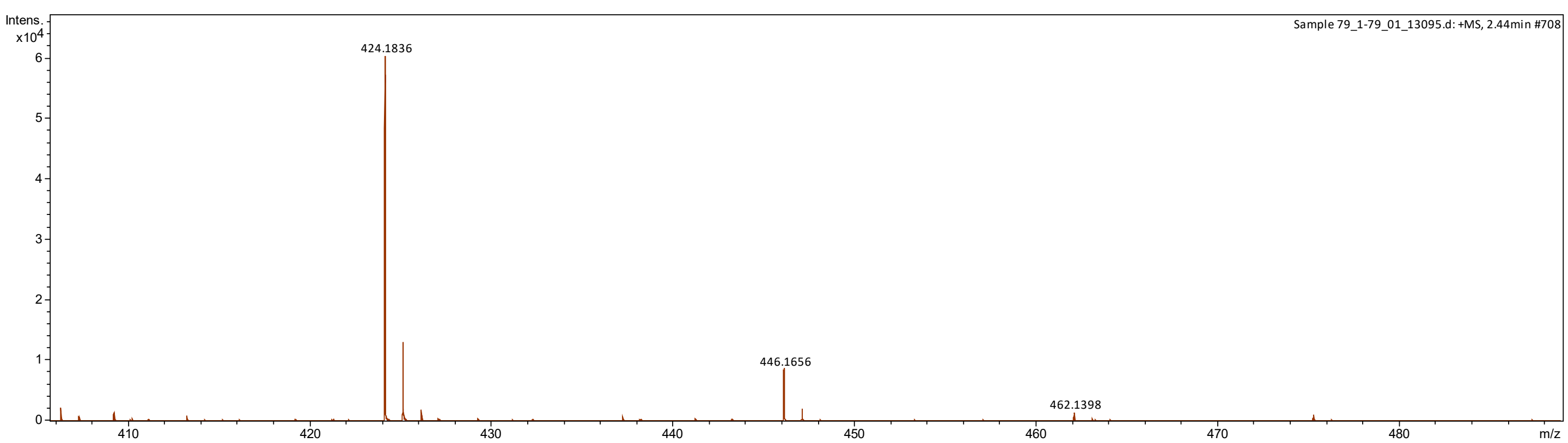

Figure S92. HRMS (ESI +) spectrum of **28**

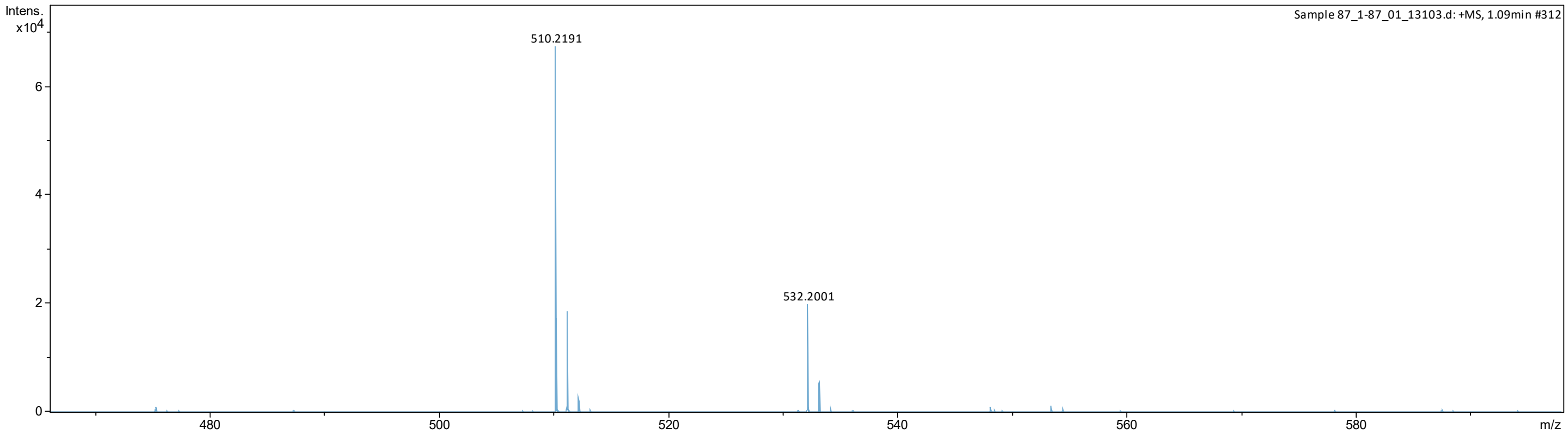

Figure S93. HRMS (ESI +) spectrum of **29**

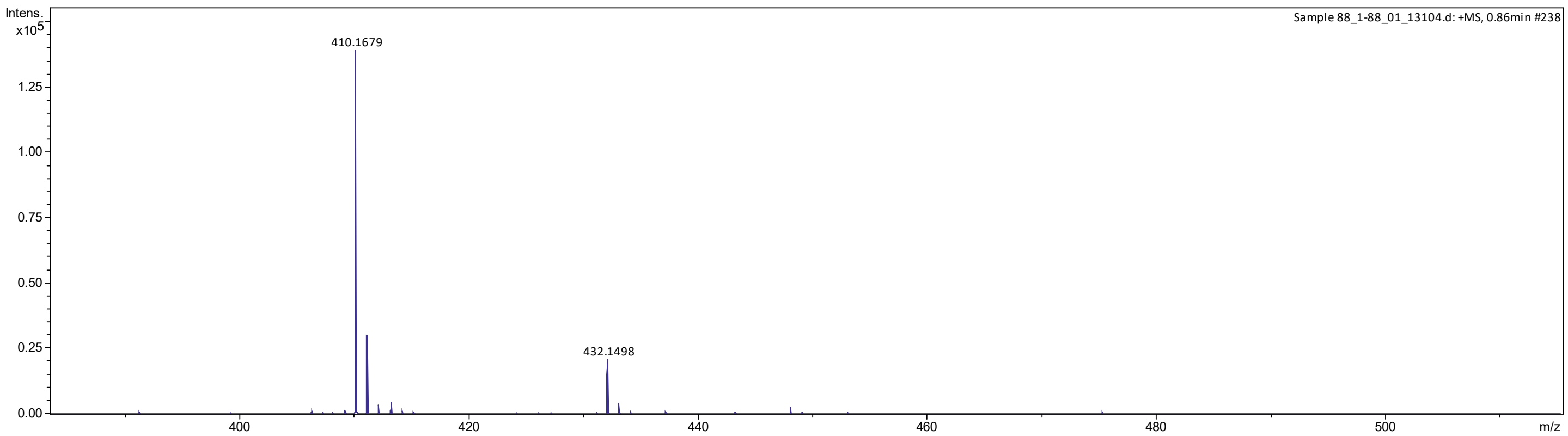

Figure S94. HRMS (ESI +) spectrum of **30**
